# Supplementary material for: Clinical efficacy and effectiveness of 3D printing: a systematic review
Source: BMJ Open. 2017 Dec 21;7(12):e016891. doi: 10.1136/bmjopen-2017-016891 (PMC5778284; doi:10.1136/bmjopen-2017-016891)
Supplement: Supplementary material 1 [file bmjopen-2017-016891supp001.pdf]

1. Database search *IEEE Xplore and Google Scholar required a simplified search.*

Database	PubMed (All article types)	ISI Web of Science (Article, review, case report, clinical trial, book or report)	OVID (Journal OR book)	IEEE Xplore (All article types)	Google Scholar (Exclude patents and citations)
Search Term	"**health care evaluation mechanisms**" OR "randomized controlled trial" OR "randomised controlled trial" OR "clinical trial"<br>OR (efficacy OR effectiveness OR validation OR validity OR feasibility OR cohort OR observational OR longitudinal OR retrospective OR comparative OR "exploratory study" OR "case-control study" OR "case study"<br>AND (clinical OR medical OR hospital OR biomedical OR assistive OR "health care" OR patients OR patient))<br>AND ("**Printing, Three-Dimensional**" OR "Three Dimensional Printing" OR "Three-Dimensional Printing" OR "3 Dimensional Printing" OR "3D Printing" OR "3-D Printing" OR stereolithography OR "laser melting" OR "electron beam melting" OR "fused deposition modeling" OR "fused deposition modelling" OR "rapid manufacturing" OR "rapid prototyping" OR "layered manufacturing" OR "digital manufacturing" OR bioprinting OR "3D prototyping" OR "3D fabrication" OR "rapid fabrication" OR "freeform fabrication")			randomized controlled trial OR "clinical trial" OR (efficacy OR validation OR feasibility OR "case study" AND (clinical OR medical OR hospital OR assistive OR "health care")) AND ("Three Dimensional Printing" OR "3D Printing" OR "rapid prototyping" OR "additive manufacturing")	"randomized controlled" OR "clinical trial" efficacy OR validation OR feasibility OR "case study" clinical OR medical OR hospital OR assistive OR "health care" "Three Dimensional Printing" OR "3D Printing" OR "rapid prototyping" OR "additive manufacturing"
**Number of studies**	913	700	1158	754	980

Number of studies: 4505

2. Duplicate removal *The first four databases were combined, then relevant additional results from Google Scholar were added.*

Number of studies: 3084
