# Supplementary material for: Clinical efficacy and effectiveness of 3D printing: a systematic review
Source: BMJ Open. 2017 Dec 21;7(12):e016891. doi: 10.1136/bmjopen-2017-016891 (PMC5778284; doi:10.1136/bmjopen-2017-016891)
Supplement: Author's manuscript [file bmjopen-2017-016891.draft_revisions.pdf]

BMJ Open is committed to open peer review. As part of this commitment we make the peer review history of every article we publish publicly available.

When an article is published we post the peer reviewers' comments and the authors' responses online. We also post the versions of the paper that were used during peer review. These are the versions that the peer review comments apply to.

The versions of the paper that follow are the versions that were submitted during the peer review process. They are not the versions of record or the final published versions. They should not be cited or distributed as the published version of this manuscript.

BMJ Open is an open access journal and the full, final, typeset and author-corrected version of record of the manuscript is available on our site with no access controls, subscription charges or pay-per-view fees (http://bmjopen.bmj.com).

If you have any questions on BMJ Open's open peer review process please email editorial.bmjopen@bmj.com

BMJ Open

Clincal efficacy and effectiveness of additive manufacturing: A systematic review

Journal:	*BMJ Open*
Manuscript ID	bmjopen-2017-016891
Article Type:	Research
Date Submitted by the Author:	16-Mar-2017
Complete List of Authors:	Diment, Laura; University of Oxford, Department of Engineering, Institute of Biomedical Engineering<br>Thompson, Mark; University of Oxford, Department of Engineering, Institute of Biomedical Engineering<br>Bergmann, Jeroen; University of Oxford, Department of Engineering, Institute of Biomedical Engineering
**Primary Subject Heading**:	Patient-centred medicine
Secondary Subject Heading:	Evidence based practice
Keywords:	Printing, Three-Dimensional, Health Care Evaluation Mechanisms, Medical devices, Personalised healthcare, Fabrication, Surgical tool

SCHOLARONE™
Manuscripts

For peer review only

Title page

Clinical efficacy and effectiveness of additive manufacturing: A systematic review

Corresponding Author:

Laura E. Diment
DPhil student
Department of Engineering Science
University of Oxford
Parks Road, Oxford, UK
OX1 3PJ
laura.diment@eng.ox.ac.uk
Ph. 01865 227374

Co-authors:

Mark S. Thompson
Director, Oxford Mechanobiology Group
Department of Engineering Science
University of Oxford
Parks Road, Oxford, UK
OX1 3PJ

Jeroen HM. Bergmann
Associate Director, Oxford Biodesign Programme
Department of Engineering Science
University of Oxford
Parks Road, Oxford, UK
OX1 3PJ

Word count: 2899

For peer review only

ABSTRACT

Objective
To evaluate the clinical efficacy and effectiveness of using additive manufacturing (AM) to develop medical devices across all medical fields.

Design
PRISMA-compliant systematic review

Data sources
PubMed, Web of Science, OVID, IEEE Xplore and Google Scholar.

Methods
A double-blinded review method was used to select all abstracts that reported on clinical trials of an AM medical device. While reviewing the full papers, additional referenced studies that met the criteria were included. The studies were ranked according to their level of evidence, divided into medical fields based on the International Classification of Diseases chapter divisions, and categorised into whether they were used for preoperative planning, aiding surgery or therapy. The Downs and Black Quality Index critical appraisal tool was used to assess the quality of reporting, external validity, risk of bias, risk of confounding and power of each study. Finally, the outcomes and quality of studies in each medical field were summarised.

Results
Of the 3084 abstracts screened, 350 studies met the inclusion criteria, including 21 randomised controlled trials. Oral and Maxillofacial Surgery contained 58.3% of studies, and 23.7% covered the Musculoskeletal System. The medical fields that assessed AM devices concluded that they were clinically effective. However, there were few high-quality controlled studies performed in areas other than oral and maxillofacial surgery and musculoskeletal surgery.

Conclusions
The efficacy and effectiveness of AM devices remains undetermined for the majority of medical fields. More high-quality randomised controlled trials (RCTs) are needed to determine if AM devices are clinically relevant. The few RCTs that were performed showed results in favour of using the AM devices in musculoskeletal surgery, and oral and maxillofacial surgery. AM devices can have an important role to play in healthcare, but more rigorous and long-term assessments are needed before these devices can become part of standard clinical practice.

Strengths and limitations of the study

Previous reviews have documented the uses of additive manufacturing (AM) for developing patient-specific medical devices. However, there has been limited research into assessing the efficacy and effectiveness of these devices. This review demonstrates that Oral and Maxillofacial Surgery and Musculoskeletal Surgery are the leading medical fields in clinically validating AM devices, but all fields require more rigorous and long-term assessments into the efficacy and effectiveness of AM devices before they are introduced into standard clinical practice.

Because of the breadth of uses for anatomical models, surgical guides and therapeutic devices, few of the studies had comparable hypotheses. No meta-analysis could be performed due to the broad scope of this review and the limited number of high-quality studies published.

For peer review only - http://bmjopen.bmj.com/site/about/guidelines.xhtml

INTRODUCTION

Additive manufacturing (AM) is likely to play a pivotal role in transforming healthcare and clinical practice because it provides the opportunity to create customised devices designed for the complexity and individual variances of the patient populations.[1]

AM can be applied across many medical fields for manufacturing custom surgical tools, guides, dose delivery devices, implants, external prosthetics or orthotics, and devices for preoperative planning or education.[2–4] Tailoring devices and procedures to the patient is expected to reduce the time required for surgery, treatment or recovery, while increasing the accuracy and success of the outcome.[1]

AM enables a 3D digital model to be converted into a physical model by printing it layer by layer. For decades AM has been used for rapid prototyping, but the recent advances in the available materials, speed, resolution, accuracy, reliability, cost and repeatability of AM technologies have broadened the possibilities for clinical uses.[3,5] The worldwide 3D printing industry's revenue from products and services is over $4 billion and fast-growing, with 13.1% of the industry attributed to the medical sector.[6]

In most medical fields, AM applications are still in the research and development stage or have only just entered clinical practice within the last decade, and hence there has been a lack of research into the clinical efficacy, effectiveness and long-term follow-up in comparison to traditional technologies.[7] *Efficacy* refers to the performance of the device under ideal and controlled conditions, and *effectiveness* refers to its performance under typical clinical conditions.[8] The Food and Drug Administration (FDA) states that AM medical devices are required to meet the same regulations as their non-AM counterparts, demonstrating evidence of efficacy and effectiveness through randomised clinical trials.[9] There is currently ambiguity in how non-traditional manufacturers, such as hospitals and clinics, must adhere to these regulations and how these sites will be regulated but as further clarity is provided on these issues, it is expected that these manufacturers will also be required to demonstrate proof of device efficacy and effectiveness before prescribing AM devices for patients. Providing a critical appraisal of the efficacy and effectiveness of AM medical devices gives healthcare professionals a resource to assess the validity of the devices, and provides researchers with an overview of areas that require further research and validation.

Previous systematic reviews summarise AM medical devices being used in specific medical fields, such as plastic and reconstructive surgery,[10] preoperative planning for liver resections,[11] and increasingly available AM processes used in dentistry and mandibular reconstructions.[12–14] Reviews on the advantages of AM devices over conventional methods in surgery have found improved clinical outcomes, and reductions in operating times and manufacturing costs.[4,15] However, the reviews do not critically appraise the studies, and are therefore subject to bias. The only systematic review that did asses bias for a very specific field found no difference in patient outcomes when AM instrumentation for total knee replacements was compared to conventional instrumentation.[16] This highlights the need for a systematic review that incorporates a critical appraisal of the studies that are included.

This review aims to assess the clinical efficacy and effectiveness of AM devices through performing a systematic literature search, categorising reports of AM device usage by medical field and purpose, and assessing the reports' scientific quality using published tools and indices.

METHODS

Search strategy

The applied protocol (not previously registered) used systematic methods to search for relevant studies, screen them for eligibility, and assess their quality. The review follows the Preferred Reporting Items for Systematic Reviews and Meta-Analyses (PRISMA) guidelines.[17]

A literature search was performed using PubMed, Web of Science, OVID, IEEE Xplore and Google Scholar. A combination of 45 relevant keywords were used to collect all studies that included an AM device and a clinical trial. Due to the search restrictions in IEEE Xplore and Google Scholar, a narrower search of 15 terms was performed for these databases. The search includes all publications up to January 2017. The complete search strategy is provided in Appendix 1.

Study selection

After removing all duplicates from the databases, the title and abstract of each publication was reviewed using a double-blinded method to determine eligibility for inclusion. Where the results of the reviewers (LD and JB) conflicted, the reviewers discussed their reasoning for inclusion/exclusion, and where required for

For peer review only - http://bmjopen.bmj.com/site/about/guidelines.xhtml

clarification of the paper's relevance, the paper's introduction and methods were assessed. Consensus on eligibility was achieved for all papers.

The inclusion criteria were:

i. **Relevance -** papers were required to report on a clinical study using an AM device.
ii. **Language -** only papers written or translated into English were included in the review.
iii. **Peer-review -** records that had no peer-review or where the level of peer-review could not be traced were excluded from the review.

Included articles underwent a full-text review and references cited in these studies were also examined for relevance using the same inclusion criteria.

Quality assessment

The studies were then rated according to their level of evidence, based on the Oxford Centre for Evidence-based Medicine (CEBM) Levels of Evidence,[18] as summarised in Table 1. Systematic reviews and opinion papers were excluded because they cannot provide the original data in a suitable format to be assessed using the quality assessment method used in this review. Where studies used AM devices but the hypothesis was focused on an outcome other than the effect of the AM device, the paper has been graded as the level of evidence shown for the AM device.

Table 1. CEBM Levels of Evidence

Level	Types of study
1	- Randomised controlled trials (RCTs)
2	- Cohort studies
3	- Case-control studies<br>- Poor quality estimates of data that include sensitivity analyses incorporating clinically sensible variations
4	- Case-series studies
5	- Case reports<br>- Studies that do not provide quantitative evidence to support a hypothesis

Studies were then placed in medical fields based on the International Classification of Diseases (ICD) chapter divisions[19] and categorised by the purpose of the device. The three categories were; devices used for preoperative planning, devices to aid surgery, and therapeutic devices.

To analyse the quality of the studies under review, critical appraisal tools were assessed to find one that met the following criteria:

- Suitable for assessing the quality of both randomised and non-randomised studies,
- Well-regarded and commonly used for quality assessments in systematic reviews.
- Demonstrates internal consistency, test-retest reliability and criterion validity.
- Assesses the risk of bias, the participant selection methods, the study protocol, and the validity, reliability and responsiveness of the study's results.
- Simple and intuitive to interpret.
- Does not assign weights to the items in the scale where there is a lack of empirical evidence to support the assignment.[20]

The Downs and Black Quality Index[21] meets all but the final criterion on this list. To overcome this limitation, responses have been left in their raw form (yes/no/unable to determine/not applicable) and presented in a table to enable the reader to visualise the category trends, as recommended by the Cochrane Handbook for Systematic Reviews of Interventions.[20] The study details (intervention, details of participants and primary outcomes) were also tabulated.

For peer review only - http://bmjopen.bmj.com/site/about/guidelines.xhtml

For peer review only

RESULTS

The search yielded 4505 records and, after removing duplicates, 3084 abstracts were screened. A total of 350 studies met the inclusion criteria and were included in the review (Figure 1). The studies covered 9 medical fields, with the majority (58.3%) of studies falling into the Oral and Maxillofacial Surgery field, which included dentistry and orthopaedic surgery of the jaw, face and skull, and those covering the Musculoskeletal System (23.7%) making up the second group. Some studies spanned multiple medical fields, such as neoplasms that occur in the circulatory system. In these cases, the authors selected the most prominent field of the paper.

Figure 1. Flow chart of the selection and sorting method

Only 14.0% of included studies used a control group (Levels 1 to 3 in Figure 2), whereas 41.4% were level 5 studies. The average number of participants per controlled trial was 41 (36 for RCTs), compared to 17 for all studies.

Figure 2. Number of studies per level of evidence given by medical field. Levels of evidence are colour coded and fields are visually separated by alternating between white and grey columns.

A wide range of AM devices have been developed and clinically trialled. The most common device types were anatomical models for preplanning surgeries (40.9%), followed by guides to aid in surgery (37.1%). A summary of all RCTs is provided in Table 2. Information for all studies included in the review is summarised in Appendix 2.

For peer review only

Table 2. Summary of RCTs (Type: P = preoperative planning, S = surgical tool or guide, T = therapeutic device. + = significant results in favour of the AM device)

Sector	First author	Date	Device	Type	Size (n)	Participant details	Aim	Outcomes
Neoplasms	de Farias[22]	2014	Mandible model	P	37 (Test=17, Control=20)	Age: 9 to 74 years (Test mean = 39.57 years, Control mean = 53.11 years). Test = 14M/3F, Control = 15M/5F	Aim: to evaluate the efficacy of using AM models to plan head and neck surgeries.	+ The average reconstruction time decreased; Test=43.7 minutes, Control=127.7 minutes, p=0.001). Though significance was not given, the size of the bone flap taken for reconstruction was typically smaller, and the aesthetic results better in the test group.
Oral and Maxillofacial	Ahrberg[23]	2015	All-ceramic zirconia crowns	T	25 (Same participants used for test and control)	Age: >18 years 10M/15F	Aim: to evaluate the marginal and internal fit of AM fabricated zirconia crowns and three-unit fixed dental prostheses resulting from direct versus indirect digitalization.	+ The mean for the marginal gap was significantly (p<0.05) smaller 61.08 µm (±24.77 µm) for the test group compared with the control 70.40 µm (±28.87 µm). The test group showed significantly lower values of internal fit at the centro-occlusal site.
Oral and Maxillofacial	D'Urso[24]	1999	Cranio-maxillofacial model	P	30 (Test=15, Control=15)	No information provided	Aim: to assess whether biomodels in addition to standard imaging have greater utility in patient education, diagnosis and operative planning than the standard imaging alone.	+ AM models improved operative planning (p<0.01) and diagnosis (p<0.01) based on images, and measurement accuracy improved (p<0.05). Operating time was also reduced by an estimated 17.63%.
Oral and Maxillofacial	Al-Ahmad[25]	2013	Surgical guide for sagittal splitting ramus osteotomy	S	8 (One side used as test, the other as control)	Age: 18-30 years (mean=23 years)	Aim: to evaluate the effectiveness of SSRO in reducing the incidence and severity of neurosensory alterations, using an AM surgical guide.	+ The test group had lower postoperative abnormal thresholds for the Semmes–Weinstein monofilaments on lower lip and chin (p<0.05 at 3 months) and for the two-point discrimination on lower lip (p<0.05 at 1 week) and chin (p<0.05 at 6 months), with fewer abnormal self-reported changes in lower lip sensation (p<0.05 at 1 week).
Oral and Maxillofacial	Ayoub[26]	2014	Guide for mandibular reconstruction	S	20 (Test=10, Control=10)	Age: Test=52.3 ± 19.2 years, Control=54.7 ± 14.2 years). Test=4M/6F, Control=6M/4F	Aim: to evaluate the benefits of computer-assisted mandibular reconstruction with iliac crest bone grafts regarding the intraoperative time for transplant shaping, ischemia, duration of surgery, amount of bone removed and the change in postoperative condyle position compared to conventional surgery.	+ Computer-assisted surgery shortened the time of transplant ischemia (p<0.005) and defect reconstruction (p<0.001) compared to conventional surgery. There was less bone harvested in the test group (p<0.001), and the intercondylar distance before compared to after surgery was less affected in the computer-assisted than in the conventional group (p<0.001).
Oral and Maxillofacial	Goh[27]	2015	Polycaprolactone scaffold for ridge preservation	T	13 (Test=6, Control=7)	Age: 29–60 years (mean=46.8 years). 7M/7F (1 lost to follow-up, so excluded from results)	Aim: to evaluate the feasibility and effectiveness of using a polycaprolactone scaffold in fresh extraction sockets for ridge preservation.	+ The test group showed less vertical ridge resorption (significant in the mesio-buccal aspect: p = 0.008), with better maintenance of ridge height after 6 months.
Oral and Maxillofacial	Van de Velde[28]	2010	Maxilla surgical guide	S	13 (Test=36 tooth implants, Control=34 tooth implants)	Age: 39-75 years (mean =55.7 years), 4M/9F	Aim: to compare the outcome of dental implants placed using a flapless protocol with an AM guide and immediate loading with a conventional protocol and loading.	+ Implants can successfully integrate in the posterior maxilla using a flapless approach with immediate loading. The mucosal tissues around implants changed significantly less compared with controls (p<0.05). Marginal bone levels were not significantly different between test and control implants. Opinion about speech, function, aesthetics and self-confidence was significantly better for the test side after 1 week (p<0.05).
Oral and Maxillofacial	Vercruyssen[29]	2014	Surgical guides for edentulous jaws	S	59 (72 jaws, 12 in each group)	Age:31-78 years 31 male jaws, 41 female jaws	Aim: To assess the accuracy of guided surgery compared to mental navigation or the use of a surgical template, in fully edentulous jaws.	+ Lower deviation at the entry point, at the apex and angular deviation when using the AM guiding systems than when using mental navigation or a conventional surgical template (all p<0.05). There were negligible differences between bone and mucosa support or type of guidance.
Musculoskeletal	Chareancholvanich[30]	2013	Cutting guides for total knee replacement	S	80 (Test=40, Control=40)	Age: 55-84 years (mean = 69.5 years) 6M/34F	Aim: to compare patient-specific cutting guides with conventional instrumentation in total knee replacement.	+ The tibial component in the test group was closer to neutral alignment (p=0.03), but the size of the difference was small (89.8°±1.2° vs 90.5°±1.6°). The AM guides also shortened the bone-cutting time by a 3.6 minutes (p<0.001) and the operating time by 5.1 minutes (p=0.019), without differences in post-operative blood loss (p=0.528) or need for blood transfusion (p=0.789). These minimal advantages of the AM guides are unlikely to be clinically relevant.

For peer review only - http://bmjopen.bmj.com/site/about/guidelines.xhtml

Musculoskeletal	Chen[31]	2015	Guide plate for pedicle screw fixation	S	43 (Test=20, Control=23)	Age: Test=35-70 years (mean=52.3 years) Control=37-72 years (mean=55.4 years) Test=9M/11F, Control=12M/11F	Aim: to evaluate the clinical efficacy of use of a 3D printing guide plate in posterior lumbar pedicle screw fixation.	+ Placement time for each screw was shorter, the amount of hemorrhage was less, and the fluoroscopy times of each screw placement was lower in the test group than in the control group (all p<0.05). The excellent and good screw placement rate was 100% in the test group and 98.4% in the control group (p>0.05). No obvious complications were reported.
Musculoskeletal	Du[32]	2013	Templates to aid in pin placement in hip resurfacing arthroplasty	S	34 (Test-16, Control=18)	Ages: 37-55 years	Aim: To assess whether AM patient-specific templates aid in accurate intraoperative pin placement.	+ The prosthesis stem shaft angle was greater for the test group (p=0.001). The locating template can provide precise and dependable location for hip resurfacing femoral components during arthroplasty and ensure the valgus stem placement necessary for optimal outcomes.
Musculoskeletal	Gan[33]	2015	Navigational template for total knee arthroplasty	S	70 (Test=35, Control=35)	Age: Test=63-75 years (mean=68 years), Control=64-72 years (mean=67.8 years) Test=10M/25F, Control=9M/26F	Aim: to validates a novel patient-specific navigational template for total knee arthroplasty.	+ The AM navigational template reduced errors, operation time and blood loss (all p<0.05).
Musculoskeletal	Hendel[34]	2012	Guide for determining glenoid component position	S	31 (Test=15, Control=16)	No information provided	Aim: to compare patient-specific instruments with standard surgical instruments in determining glenoid component position.	+ The AM technology decreased the average deviation of implant position for inclination and medial-lateral offset (p<0.05). The greatest benefit was observed in patients with retroversion >16˚ (p<0.001).
Musculoskeletal	Maini[35]	2016	Plate for acetabulum fracture fixation	P	21 (Test=10, Control=11)	Age: 18-60 years (mean=38.7 years) Test=9M/1F, Control=9M/2F	Aim: to evaluate the accuracy of patient-specific pre-contoured plates.	+ Reduction achieved, as evaluated by CT scan, was more in the test group than the control group (p<0.05). Reduced blood loss (100 ml less) and surgical time (12 min less) were observed in the test group but the results were not significant (p>0.05). All plates fitted well to the pelvis intra-operatively.
Musculoskeletal	Merc[36]	2013	drill guide for screw placement	S	19 (Test=9 (54 screws), Control=10(54 screws))	Age: Test=59 ± 5 years, Control=62 ±12 years Test=4M/5F, Control=5M/5F	Aim: to evaluate the accuracy of a multi-level drill guide template for lumbar and first sacral pedicle screw placement and to compare it with the freehand technique under fluoroscopy supervision.	+ The incidence of cortex perforation was significantly reduced in the test group (p<0.05). So was the deviation and displacement level of screws in the sagittal plane but not in the transverse plane.
Musculoskeletal	Shuang[37]	2016	Osteosynthesis Plates	T	13 (Test=6, Control=7)	Age: Test=31-62 years (mean=46.2 years) Control=27-59 years (mean=40.3 years) Test= 4M/2F, Control=6M/1F	Aim: to evaluate the efficacy custom AM osteosynthesis plates in the treatment of intercondylar humeral fractures.	+ Operative time was shorter for the test group than the control group (p=0.026). The mean time to bone union was 3.4 months. No significant difference was found in the rate of patients with good elbow function, or in the ranges of elbow flexion/extension and pronation/supination (all p>0.05).
Musculoskeletal	Stephens[38]	2016	Bone models	P	58 (Test=29, Control=29)	Age: >60 years (mean=73 years) 5M/53F	Aim: to investigate the efficacy of AM bone models as a tool to facilitate initiation of bisphosphonate treatment among individuals who are newly diagnosed with osteoporosis.	+ The test group were more emotionally affected by osteoporosis immediately after the interview (p=0.04) and reported a greater understanding of osteoporosis at follow-up (p=0.04), than the control group.
Musculoskeletal	Wu[39]	2011	Spine model	P	62 (Test=34, Control=28) 677 screws inserted	Age: 4-22 years (mean=11 years)	Aim: to compare the accuracy and safety of pedicle screw placement in congenital scoliosis using the AM technique versus conventional fluoroscopy.	+ Compared with the fluoroscopy method, the AM assisted technique had a shorter operation time and higher scoliosis correction rate (both p<0.05). Accuracy of placement was 89.7% (84.7% in the control group; 93.5% in the test group).
Musculoskeletal	Yang[40]	2016	Model of trimalleolar fracture	P	30 (Test=15, Control=15)	Age: 31-42 years (mean=36.5 years) 16M/14F	Aim: to evaluate the effectiveness of using AM models for surgical planning in treating trimalleolar fractures and in physician-patient communication.	+ Both the operation time and the intraoperative blood loss were less in the test group than in the control group (both p<0.05). Patient satisfaction with using the AM device for communication was scored highly.
Musculoskeletal	You[41]	2016	Proximal humeral fracture model	P	66 (Test=34, Control=32)	61-76 years (Test = 66.09±4.09 years, Control = 66.28± 4.10 years) 27M/39F	Aim: to investigate the feasibility and clinical potential of using AM models versus the conventional thin-layer CT scan for the treatment of proximal humeral fractures in old people.	+ Reduced surgery duration, less blood loss during surgery, and lower number of fluoroscopy was observed in the test group compared with the control group (all p<0.05). The results showed no significant difference in time to union between the groups (p>0.05).
Musculoskeletal	Zhang[42]	2011	Acetabular navigational template	S	22 (Test=11, Control=11)	Age: Test=48.6 ± 6.8 years, Control=49.3 ± 4.9 years Test=7M/4F, Control=5M/6F	Aim: to compare a customised AM acetabular navigational template for total hip arthroplasty (THA) to conventional THA in adult single development dysplasia of the hip.	+ The test group showed smaller differences from the predetermined angles than the control group (p<0.05).

For peer review only - http://bmjopen.bmj.com/site/about/guidelines.xhtml

A critical appraisal of all comparative studies is provided in Appendix 2. Critical appraisal of the RCTs from each medical field are shown in Table 3. The table includes the abridged quality index questions. Studies with higher levels of evidence typically rated better across all areas assessed using the quality index. However, many of the RCTs did not demonstrate external validity, and did not blind participants or those measuring the outcomes, or assess confounding factors between groups. Only 57% of the RCTs included follow-up.

Table 3. Critical appraisal of studies with the highest level of evidence from each medical field.

+ Yes
- No
? Unable to determine
x Not applicable

Medical sector	First author	Date	Reporting										External validity			Internal validity - bias							Internal validity - confounding						Power
			1. Clear hypothesis/aim/objective	2. Clear outcome measures	3. Patient characteristics described	4. Interventions clearly described	5. Distributions of confounders described	6. Findings clearly described	7. Estimates given of random variability	8. Adverse events reported	9. Patients lost to follow-up described	10. Probability values reported	11. Recruitment pool represents population	12. Participants represent population	13. Staff/places/facilities match std. treatment	14. Participants blinded to intervention	15. Those measuring outcomes blinded	16. Data dredging reported	17. Adjusted for different lengths of follow-up	18. Appropriate statistical tests ecruitment	19. Reliable compliance with intervention	20. Accurate/reliable outcome measures	21. Groups recruited from same population	22. Groups recruited over same timeframe	23. Subjects randomised into intervention	24. Randomised intervention concealed	25. Adjustment for confounding	26. Losses to follow-up accounted for	27. Sufficient power
Neoplasms	de Farias	2014	+	+	+	+	+	+	+	+	x	+	?	?	+	+	+	+	x	+	+	+	+	+	+	+	?	x	+
Oral and Maxillofacial	Ahrberg	2015	+	+	+	+	+	+	+	-	x	+	?	?	+	+	+	+	x	+	+	+	+	+	+	+	+	x	+
Oral and Maxillofacial	D'Urso	1999	+	+	+	+	+	+	+	-	x	-	?	?	+	-	-	+	x	+	+	+	+	+	+	?	-	x	+
Oral and Maxillofacial	Al-Ahmad	2013	+	+	+	+	x	+	+	-	+	+	?	?	+	+	+	+	+	+	+	+	+	+	+	+	+	+	+
Oral and Maxillofacial	Ayoub	2014	+	+	+	+	-	+	+	-	x	-	?	?	+	-	-	+	x	+	+	+	+	+	+	?	-	x	+
Oral and Maxillofacial	Goh	2015	+	+	+	+	-	+	+	-	+	+	?	?	+	-	+	+	+	+	+	+	+	+	+	?	-	+	+
Oral and Maxillofacial	Van de Velde	2010	+	+	+	+	-	+	+	+	+	+	?	?	+	-	+	+	+	+	+	+	+	+	+	?	-	+	+
Oral and Maxillofacial	Vercruyssen	2014	+	+	+	+	-	+	+	+	+	-	?	?	+	-	+	+	+	+	+	+	+	+	+	?	-	+	+
Musculoskeletal	Chareancholvanich	2013	+	+	+	+	-	+	+	+	+	+	?	?	+	-	+	+	-	+	+	+	+	+	+	?	-	+	+
Musculoskeletal	Chen	2015	+	+	+	+	+	+	+	+	+	-	?	?	+	+	-	+	+	+	+	+	+	+	+	+	+	+	+
Musculoskeletal	Du	2012	+	+	-	-	+	+	+	-	+	+	?	?	+	-	-	+	-	+	+	+	+	+	+	?	-	+	+
Musculoskeletal	Gan	2015	+	+	+	+	+	+	+	+	x	+	?	?	+	-	+	+	x	+	+	+	+	+	+	?	-	x	+
Musculoskeletal	Hendel	2012	+	+	-	+	-	+	+	+	-	+	+	+	+	-	+	+	?	+	+	+	+	+	+	-	-	?	+
Musculoskeletal	Maini	2016	+	+	+	+	-	+	+	+	x	+	?	?	+	-	-	+	x	+	+	+	+	+	+	?	-	x	+
Musculoskeletal	Merc	2013	+	+	+	+	+	+	+	+	x	+	+	+	+	-	-	+	x	+	+	+	+	+	+	?	-	x	+
Musculoskeletal	Shuang	2016	+	+	-	+	+	+	+	+	+	+	?	?	+	-	-	+	?	+	+	+	+	+	+	+	+	+	+
Musculoskeletal	Stephens	2016	+	+	+	+	-	+	+	-	+	+	?	?	?	+	+	+	+	+	+	+	+	+	+	+	-	+	+
Musculoskeletal	Wu	2011	+	+	-	+	-	+	+	+	+	+	+	+	+	-	-	+	-	+	+	+	+	+	+	?	-	+	+
Musculoskeletal	Yang	2016	+	+	-	+	-	+	+	+	x	+	?	?	+	-	-	+	x	+	+	+	+	+	+	?	-	x	+
Musculoskeletal	You	2016	+	+	-	+	+	+	+	+	+	+	+	+	+	-	-	+	-	+	+	+	+	+	+	?	+	+	+
Musculoskeletal	Zhang	2011	+	+	-	+	+	+	+	-	+	+	?	?	+	-	-	+	-	+	+	+	+	+	+	?	+	+	+

DISCUSSION

The results show that very few papers have rigorously assessed the efficacy or effectiveness of clinical AM devices. The majority of RCTs assessed surgical guides that aid cutting or navigating in oral and maxillofacial surgery or musculoskeletal surgery, or assessed anatomical models to aid in surgery preplanning. There were also two RCTs that tested therapeutic devices; one evaluated dental crowns and one assessed scaffolds. The RCT listed under Neoplasms was an anatomical model used for planning mandibular surgery, but with a focus on tumour removal, so no RCTs were found outside the oral and maxillofacial surgery or musculoskeletal surgery fields. The AM devices tested were mainly designed for planning surgeries, with little attention dedicated to interventions. Therapeutic devices such as implants, prosthetic limbs and orthotics offer the promise of revolutionising the medical industry because of their ability to be custom-made for the patient, but most therapeutic devices are still early-stage, with little research into their efficacy and effectiveness in a clinical setting.

For peer review only - http://bmjopen.bmj.com/site/about/guidelines.xhtml

The RCTs all demonstrated significant results in favour of using the AM device over the current clinical practice to which it was compared. The main conclusions drawn from these trials were that AM devices were able to reduce operating time, with less blood-loss and more accuracy, and the patients experienced less swelling post-surgery. However, one RCT[30] concluded that the improvement with AM was too small to provide a clinical advantage. There might also be a publication bias towards positive trials.

Most excluded AM device studies had uses in medical training, or as moulds for manufacturing and therefore did not meet the inclusion requirement. A few other studies were excluded because they had not been translated into English.

The majority of studies reviewed were poor quality. Most did not use a control group or include follow-up, and few addressed the external validity, bias or confounding factors. The vast majority of papers reported positive outcomes for the patients, but few studies demonstrated clinically significant findings. Until independent groups start validating AM devices, it is difficult to avoid researcher bias.

While this review demonstrates that AM has been used for a broad range of applications, the 41.4% of papers that reported on individual case studies or provided only qualitative results do not clinically validate the devices that they report on, and therefore add little value to their fields. Without endeavouring to avoid bias, many of the controlled trials also provide limited value.

All the RCTs were single site trials, and even the largest RCT only compared 40 participants to 40 controls, providing limited evidence to support the device's adoption into standard clinical practice. Despite these limitations, the RCTs demonstrated significant results in support of integrating AM into clinical practice in the areas they assessed.

The fields of Oral and Maxillofacial Surgery and Musculoskeletal Surgery are leading the way in validating AM devices for clinical use. Multiple high-quality studies have been performed on surgical guides for maxillofacial, hip and knee surgeries. This growing body of comparable high-quality research sets an example for other fields to emulate in order to demonstrate the efficacy and effectiveness required to integrate these AM devices into clinical practice.

Because of the breadth of uses for anatomical models, surgical guides and therapeutic devices, few of the studies had comparable hypotheses. No meta-analysis could be performed due to the broad scope of this review and the limited number of high-quality studies published.

A critical appraisal of the efficacy and effectiveness of AM devices across medical fields provides clinicians with an evidence-based approach to determine the applicability of AM within their field. It also gives researchers an overview of areas that require further research and validation. It encourages investigators to discover what methods have been successfully validated in other medical fields and promotes potential collaborations between fields.

AM provides a way of customising devices to improve patient outcomes. Available techniques and materials will increase, as AM technology continues to be developed. There is therefore a growing need for validation of new devices, materials and techniques to ensure best patient outcomes. Much research has already gone into developing AM devices for medical purposes. However, this drive for new technology development has not yet been matched with a drive for critical appraisal of the devices to demonstrate their efficacy and effectiveness. Even the fields that are leading the way in critically evaluating new AM devices will be required to increase their output as AM continues to grow in popularity and functionality.

The early research covered by this review shows that AM use in medicine can be valuable. The next important step to take is growing the body of research that focuses on validating AM devices. Funders can take an active role in promoting not only early technological development, but also the subsequent clinical trials. Clinical efficacy and effectiveness will become increasingly important as regulatory bodies improve their ways of enforcing unconventional manufacturers, such as hospitals and clinics, to adhere to the regulations that require evidence of efficacy and effectiveness for all medical devices.

Fields such as Oral and Maxillofacial surgery, who were early in the uptake of AM, are beginning to stabilise, with a steady number of studies being published each year, but other fields, such as Musculoskeletal and Circulatory Systems, are more recently gaining traction, with increasing numbers of studies performed each year. AM drug delivery devices and biological AM technologies for printing tissue show huge promise, but have not been clinically trialled and so are not included here.[43] This is likely to

For peer review only - http://bmjopen.bmj.com/site/about/guidelines.xhtml

For peer review only

change within the near future. It is therefore recommended that in this fast-growing and dynamic environment, this review is updated every few years.

CONCLUSION

This review demonstrates that despite the large number of studies on AM devices used in the clinic, there is need for further validation of the efficacy and effectiveness of these devices. In all medical fields that have tested AM devices, there have been positive outcomes, but few studies have rigorously assessed the efficacy and effectiveness of AM devices for medical applications in areas other than Oral and Maxillofacial Surgery and Musculoskeletal Surgery. More rigorous assessment of AM devices in comparison to current devices and procedures is required, with long-term follow-up, before introducing AM devices into standard clinical practice.

Data sharing: All additional data is available in the supplementary material.

Funding sources: The researchers are financially supported by the General Sir John Monash Foundation and the Wellcome Trust.

For peer review only

For peer review only - http://bmjopen.bmj.com/site/about/guidelines.xhtml

REFERENCES

1. Zadpoor AA, Malda J. Additive Manufacturing of Biomaterials, Tissues, and Organs. Ann Biomed Eng. 2017;45(1):1–11.
2. Lee Ventola C. Medical applications for 3D printing: Current and projected uses. P T. 2014;39(10):704–11.
3. Banks J. Adding value in additive manufacturing: Researchers in the United Kingdom and Europe look to 3D printing for customization. IEEE Pulse. 2013;4(6):22–6.
4. Tack P, Victor J, Gemmel P, Annemans L. 3D-printing techniques in a medical setting: a systematic literature review. Biomed Eng Online. 2016;15(1):115.
5. Royal Academy of Engineering. Additive Manufacturing : Opportunities and Constraints [Internet]. Royal Academy of Engineering. 2013. Available from: http://www.raeng.org.uk/publications/reports/additive-manufacturing
6. Caffrey T, Wohlers T. Additive manufacturing state of the industry. Manuf Eng. 2015;154(5):67–78.
7. Mulford JS, Babazadeh S, Mackay N. Three-dimensional printing in orthopaedic surgery: Review of current and future applications. ANZ J Surg. 2016;
8. Revicki DA, Frank L. Pharmacoeconomic evaluation in the real world: Effectiveness versus efficacy studies. Vol. 15, PharmacoEconomics. 1999. p. 423–34.
9. FDA. Technical Considerations for Additive Manufactured Devices - Draft Guidance for Industry and Food and Drug Administration Staff. 2016.
10. Bauermeister AJ, Zuriarrain A, Newman MI. Three-Dimensional Printing in Plastic and Reconstructive Surgery: A Systematic Review. Ann Plast Surg. 2015;
11. Soon DSC, Chae MP, Pilgrim CHC, Rozen WM, Spychal RT, Hunter-Smith DJ. 3D haptic modelling for preoperative planning of hepatic resection: A systematic review. Vol. 10, Annals of Medicine and Surgery. 2016. p. 1–7.
12. Patzelt SBM, Spies BC, Kohal RJ. CAD/CAM-fabricated implant-supported restorations: A systematic review. Clin Oral Implants Res. 2015;26:77–85.
13. Bidra AS, Taylor TD, Agar JR. Computer-aided technology for fabricating complete dentures: Systematic review of historical background, current status, and future perspectives. Vol. 109, Journal of Prosthetic Dentistry. 2013. p. 361–6.
14. Tarsitano A, Del Corso G, Ciocca L, Scotti R, Marchetti C. Mandibular reconstructions using computer-aided design/computer-aided manufacturing: A systematic review of a defect-based reconstructive algorithm. J Cranio-Maxillofacial Surg. 2015;43(9):1785–91.
15. Martelli N, Serrano C, Van Den Brink H, Pineau J, Prognon P, Borget I, et al. Advantages and disadvantages of 3-dimensional printing in surgery: A systematic review. Vol. 159, Surgery (United States). 2016. p. 1485–500.
16. Zhang QM, Chen JY, Li H, Chai W, Ni M, Zhang ZD, et al. No Evidence of Superiority in Reducing Outliers of Component Alignment for Patient-specific Instrumentation for Total Knee Arthroplasty: A Systematic Review. Vol. 7, Orthopaedic Surgery. 2015. p. 19–25.
17. Liberati A, Altman DG, Tetzlaff J, Mulrow C, Gøtzsche PC, Ioannidis JPA, et al. The PRISMA statement for reporting systematic reviews and meta-analyses of studies that evaluate health care interventions: explanation and elaboration. In: Journal of clinical epidemiology. 2009. p. e1-34.
18. Oxford Centre for Evidence-based Medicine. Levels of Evidence. Univ Oxford [Internet]. 2009;4–5. Available from: www.cebm.net
19. World Health Organization. International Statistical Classification of Diseases and Related Health Problems (ICD) 10th Revision - Version:2010 [Internet]. Occupational Health. 2010. Available from: http://apps.who.int/classifications/icd10/browse/2010/en
20. Higgins JPT, Green S. Cochrane Handbook for Systematic Reviews of Interventions Version 5.1.0

For peer review only - http://bmjopen.bmj.com/site/about/guidelines.xhtml

[updated March 2011]. Vol. 4, The Cochrane Collaboration. 2011.

21. Downs SH, Black N. The feasibility of creating a checklist for the assessment of the methodological quality both of randomised and non-randomised studies of health care interventions. J Epidemiol Community Health. 1998;52(6):377–84.

22. de Farias TP, Dias FL, Galvão MS, Boasquevisque E, Pastl AC, Albuquerque Sousa B. Use of prototyping in preoperative planning for patients with head and neck tumors. Head Neck. 2013;1–31.

23. Ahrberg D, Lauer HC, Ahrberg M, Weigl P. Evaluation of fit and efficiency of CAD/CAM fabricated all-ceramic restorations based on direct and indirect digitalization: a double-blinded, randomized clinical trial. Clin Oral Investig. 2015;

24. D'Urso PS, Barker TM, Earwaker WJ, Bruce LJ, Atkinson RL, Lanigan MW, et al. Stereolithographic biomodelling in cranio-maxillofacial surgery: a prospective trial. J Craniomaxillofac Surg. 1999;27(1):30–7.

25. Al-Ahmad HT, M Saleh MW, Hussein AM. Evaluation of an innovative computer-assisted sagittal split ramus osteotomy to reduce neurosensory alterations following orthognathic surgery: a pilot study. Int J Med Robot. 2013;9(2):134–41.

26. Ayoub N, Ghassemi A, Rana M, Gerressen M, Riediger D, Hölzle F, et al. Evaluation of computer-assisted mandibular reconstruction with vascularized iliac crest bone graft compared to conventional surgery: a randomized prospective clinical trial. Trials. 2014;15:114.

27. Goh BT, Teh LY, Tan DBP, Zhang Z, Teoh SH. Novel 3D polycaprolactone scaffold for ridge preservation - a pilot randomised controlled clinical trial. Clin Oral Implants Res. 2015;26(3):271–7.

28. Van de Velde T, Sennerby L, De Bruyn H. The clinical and radiographic outcome of implants placed in the posterior maxilla with a guided flapless approach and immediately restored with a provisional rehabilitation: A randomized clinical trial. Clin Oral Implants Res. 2010;21(11):1223–33.

29. Vercruyssen M, Cox C, Coucke W, Naert I, Jacobs R, Quirynen M. A randomized clinical trial comparing guided implant surgery (bone- or mucosa-supported) with mental navigation or the use of a pilot-drill template. J Clin Periodontol. 2014;41(7):717–23.

30. Chareancholvanich K, Narkbunnam R, Pornrattanamaneewong C. A prospective randomised controlled study of patient-specific cutting guides compared with conventional instrumentation in total knee replacement. Bone Joint J. 2013;95–B:354–9.

31. Chen HL, Wu DY, Yang HL, Guo KJ. Clinical Use of 3D Printing Guide Plate in Posterior Lumbar Pedicle Screw Fixation. Med Sci Monit. 2015;21:3948–54.

32. Du H, Tian XX, Li T Sen, Yang JS, Li KH, Pei GX, et al. Use of patient-specific templates in hip resurfacing arthroplasty: Experience from sixteen cases. Int Orthop. 2013;37(5):777–82.

33. Gan Y, Ding J, Xu Y, Hou C. Accuracy and efficacy of osteotomy in total knee arthroplasty with patient-specific navigational template. Int J Clin Exp Med. 2015;8(8):12192–201.

34. Hendel MD, Bryan J a, Barsoum WK, Rodriguez EJ, Brems JJ, Evans PJ, et al. Comparison of patient-specific instruments with standard surgical instruments in determining glenoid component position: a randomized prospective clinical trial. J Bone Joint Surg Am. 2012;94(23):2167–75.

35. Maini L, Sharma A, Jha S, Sharma A, Tiwari A. Three-dimensional printing and patient-specific pre-contoured plate: future of acetabulum fracture fixation? Eur J Trauma Emerg Surg. 2016;1–10.

36. Merc M, Drstvensek I, Vogrin M, Brajlih T, Recnik G. A multi-level rapid prototyping drill guide template reduces the perforation risk of pedicle screw placement in the lumbar and sacral spine. Arch Orthop Trauma Surg. 2013;133(7):893–9.

37. Shuang F, Hu W, Shao Y, Li H, Zou H. Treatment of Intercondylar Humeral Fractures With 3D-Printed Osteosynthesis Plates. Medicine (Baltimore). 2016;95(3):e2461.

38. Stephens MH, Grey A, Fernandez J, Kalluru R, Faasse K, Horne A, et al. 3-D bone models to improve treatment initiation among patients with osteoporosis: A randomised controlled pilot trial. Psychol Health. 2016;31(4):487–97.

For peer review only - http://bmjopen.bmj.com/site/about/guidelines.xhtml

39. Wu Z-X, Huang L-Y, Sang H-X, Ma Z-S, Wan S-Y, Cui G, et al. Accuracy and safety assessment of pedicle screw placement using the rapid prototyping technique in severe congenital scoliosis. J Spinal Disord Tech. 2011;24(7):444–50.

40. Yang L, Shang XW, Fan JN, He ZX, Wang JJ, Liu M, et al. Application of 3D Printing in the Surgical Planning of Trimalleolar Fracture and Doctor-Patient Communication. Biomed Res Int. 2016;2016.

41. You W, Liu LJ, Chen HX, Xiong JY, Wang DM, Huang JH, et al. Application of 3D printing technology on the treatment of complex proximal humeral fractures (Neer3-part and 4-part) in old people. Orthop Traumatol Surg Res. 2016;

42. Zhang YZ, Chen B, Lu S, Yang Y, Zhao JM, Liu R, et al. Preliminary application of computer-assisted patient-specific acetabular navigational template for total hip arthroplasty in adult single development dysplasia of the hip. Int J Med Robot Comput Assist Surg. 2011;7(4):469–74.

43. Shafiee A, Atala A. Printing Technologies for Medical Applications. Trends Mol Med. 2016;22(3):254–65.

For peer review only

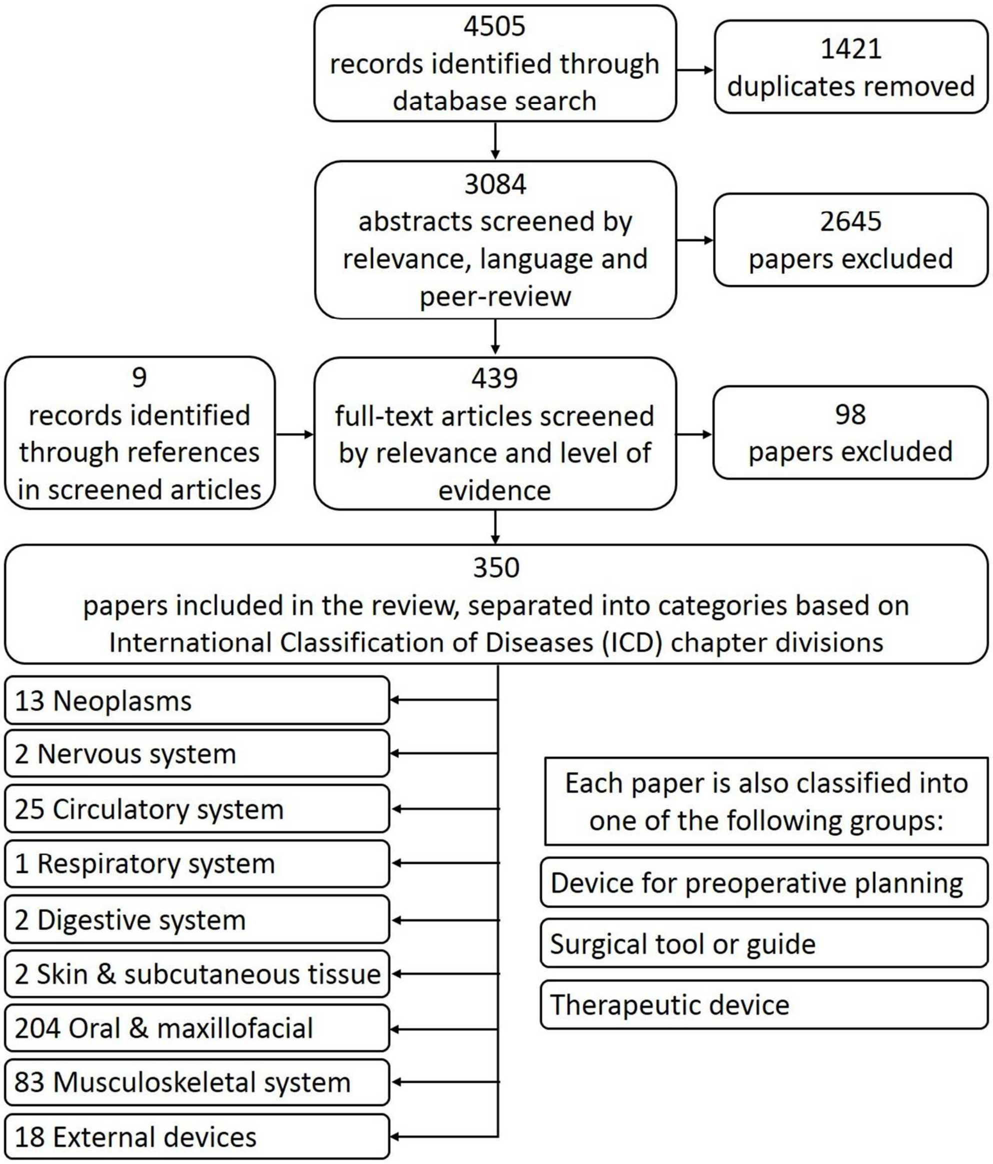

Figure 1. Flow chart of the selection and sorting method.

226x266mm (150 x 150 DPI)

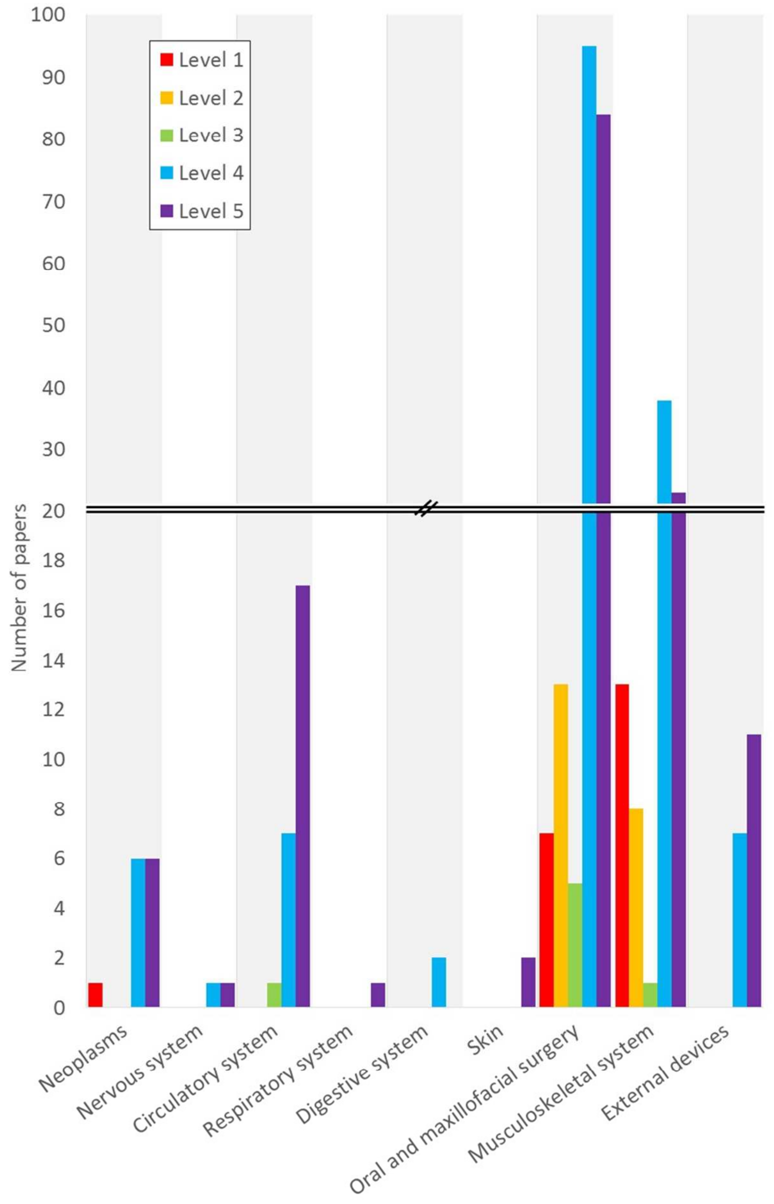

Figure 2. Number of studies per level of evidence given by medical field. Levels of evidence are colour coded and fields are visually separated by alternating between white and grey columns.

163x256mm (150 x 150 DPI)

1. Database search

IEEE Xplore and Google Scholar required a simplified search.

Database	PubMed (All article types)	ISI Web of Science (Article, review, case report, clinical trial, book or report)	OVID (Journal OR book)	IEEE Xplore (All article types)	Google Scholar (Exclude patents and citations)
Search Term	"**health care evaluation mechanisms**" OR "randomized controlled trial" OR "randomised controlled trial" OR "clinical trial"<br>OR (efficacy OR effectiveness OR validation OR validity OR feasibility OR cohort OR observational OR longitudinal OR retrospective OR comparative OR "exploratory study" OR "case-control study" OR "case study"<br>AND (clinical OR medical OR hospital OR biomedical OR assistive OR "health care" OR patients OR patient))<br>AND ("**Printing, Three-Dimensional**" OR "Three Dimensional Printing" OR "Three-Dimensional Printing" OR "3 Dimensional Printing" OR "3D Printing" OR "3-D Printing" OR stereolithography OR "laser melting" OR "electron beam melting" OR "fused deposition modeling" OR "fused deposition modelling" OR "rapid manufacturing" OR "rapid prototyping" OR "layered manufacturing" OR "digital manufacturing" OR bioprinting OR "3D prototyping" OR "3D fabrication" OR "rapid fabrication" OR "freeform fabrication")			randomized controlled trial OR "clinical trial" OR (efficacy OR validation OR feasibility OR "case study" AND (clinical OR medical OR hospital OR assistive OR "health care")) AND ("Three Dimensional Printing" OR "3D Printing" OR "rapid prototyping" OR "additive manufacturing")	"randomized controlled" OR "clinical trial" efficacy OR validation OR feasibility OR "case study" clinical OR medical OR hospital OR assistive OR "health care" "Three Dimensional Printing" OR "3D Printing" OR "rapid prototyping" OR "additive manufacturing"
**Number of studies**	913	700	1158	754	980

Number of studies: 4505

2. Duplicate removal

The first four databases were combined, then relevant additional results from Google Scholar were added.

For peer review only - http://bmjopen.bmj.com/site/about/guidelines.xhtml

Number of studies: 3084

Medical sector	Level of evidence	First author	Date	Device	Category	No. of participants	Participant details	Title	Aim	Outcomes
Neoplasms	1	de Farias	2014	Mandible model	P	37 (Test=17, Control=20)	Age: 9-74 years (Test mean=39.57 years, Control mean=53.11 years) Test=14 male, 3 female, Control=15 male, 5 female	Use of prototyping in preoperative planning for patients with head and neck tumors	Aim: to evaluate the efficacy of using AM models to plan head and neck surgeries.	+ The average reconstruction time decreased; Test=43.7 minutes, Control=127.7 minutes, p=0.001). Though significance was not given, the size of the bone flap taken for reconstruction was typically smaller, and the aesthetic results better in the test group.
Oral and Maxillofacial	1	Ahrberg	2015	All-ceramic zirconia crowns	T	25 (Same participants used for test and control)	Age: >18 years 10 male, 15 female	Evaluation of fit and efficiency of CAD/CAM fabricated all-ceramic restorations based on direct and indirect digitalization: a double-blinded, randomized clinical trial.	Aim: to evaluate the marginal and internal fit of AM fabricated zirconia crowns and three-unit fixed dental prostheses resulting from direct versus indirect digitalization.	+ The mean for the marginal gap was significantly (p<0.05) smaller 61.08 μm (±24.77 μm) for the test group compared with the control 70.40 μm (±28.87 μm). The test group showed significantly lower values of internal fit at the centro-occlusal site.
Oral and Maxillofacial	1	D'Urso	1999a	Cranio-maxillofacial model	P	30 (Test=15, Control=15)	No information provided	Stereolithographic biomodelling in cranio-maxillofacial surgery: a prospective trial	Aim: Assess whether biomodels in addition to standard imaging have greater utility in the surgery performed than the standard imaging alone.	+ Biomodels significantly improved operative planning, diagnosis and measurement accuracy. Operating time was also reduced by an estimated 17.63%.
Oral and Maxillofacial	1	Al-Ahmad	2013	Surgical guide for sagittal splitting ramus osteotomy	S	8 (One side used as test, the other as control)	Age: 18-30 years (mean=23 years)	Evaluation of an innovative computer-assisted sagittal split ramus osteotomy to reduce neurosensory alterations following orthognathic surgery: a pilot study	Aim: to evaluate the effectiveness of SSRO in reducing the incidence and severity of neurosensory alterations, using an AM surgical guide.	+ The test patients had lower postoperative abnormal thresholds for the Semmes–Weinstein monofilaments on lower lip and chin (p<0.05 at 3 months) and for the two-point discrimination on lower lip (p<0.05 at 1week) and chin (p<0.05 at 6 months), with fewer abnormal self-reported changes in lower lip sensation (p<0.05 at 1week) after surgery.
Oral and Maxillofacial	1	Ayoub	2014	Guide for mandibular reconstruction	S	20 (Test=10, Control=10)	Age: Test=52.3 ± 19.2 years, Control=54.7 ± 14.2 years) Test=4 male, 6 female, Control=6 male, 4 female	Evaluation of computer-assisted mandibular reconstruction with vascularized iliac crest bone graft compared to conventional surgery: a randomized prospective clinical trial	Aim: to evaluate the benefits of computer-assisted mandibular reconstruction with iliac crest bone grafts regarding the intraoperative time for transplant shaping, ischemia, duration of surgery, amount of bone removed and the hange in postoperative condyle position compared to conventional surgery.	+ Computer-assisted surgery shortened the time of transplant ischemia (p<0.005) and defect reconstruction (p<0.001) compared to conventional surgery. There was less bone harvested in the test group (p<0.001), and the intercondylar distance before compared to after surgery was less affected in the computer-assisted than in the conventional group (p<0.001).
Oral and Maxillofacial	1	Goh	2015	Polycaprolactone scaffold for ridge preservation	T	13 (Test=6, Control=7)	Age: 29–60 years (mean=46.8 years) 7 male, 7 female (1 was lost to follow-up, so was excluded from results)	Novel 3D polycaprolactone scaffold for ridge preservation – a pilot randomised controlled clinical trial	Aim: to evaluate the feasibility and effectiveness of using a polycaprolactone scaffold in fresh extraction sockets for ridge preservation.	+ The test group showed less vertical ridge resorption (significant in the mesio-buccal aspect: p = 0.008), with better maintenance of ridge height after 6 months.
Oral and Maxillofacial	1	Van de Velde	2010	Maxilla surgical guide	S	13 (Test=36 tooth implants, Control=34 tooth implants)	Age: 39-75 years (mean =55.7 years) 4 male, 9 female	The clinical and radiographic outcome of implants placed in the posterior maxilla with a guided flapless approach and immediately restored with a provisional rehabilitation: a randomized clinical trial	Aim: to compare the outcome of dental implants placed using a flapless protocol with an AM guide and immediate loading with a conventional protocol and loading.	+ Implants can successfully integrate in the posterior maxilla using a flapless approach with immediate loading. The mucosal tissues around implants changed significantly less compared with controls (p<0.05). Marginal bone levels were not significantly different between test and control implants. Opinion about speech, function, aesthetics and self-confidence was significantly better for the test side after 1 week (p<0.05).
Oral and Maxillofacial	1	Vercruyssen	2014	Surgical guides for edentulous jaws	S	59 (72 jaws)	Age:31-78 years 31 male jaws, 41 female jaws	A randomized clinical trial comparing guided implant surgery (bone- or mucosasupported) with mental navigation or the use of a pilot-drill template	Aim: To assess the accuracy of guided surgery compared to mental navigation or the use of a surgical template, in fully edentulous jaws.	+ Significantly lower mean deviation at the entry point, at the apex and angular deviation when using the AM guiding systems than when using mental navigation or a conventional surgical template (p<0.05). Differences between bone and mucosa support or type of guidance were negligible.
Musculoskeletal	1	Chareancholvanich	2013	Cutting guides for total knee replacement	S	80 (Test=40, Control=40)	Age: 55-84 years (mean: 69.5 years) 6 male, 34 female	A prospective randomised controlled study of patient-specific cutting guides compared with conventional instrumentation in total knee replacement	Aim: to compare patient-specific cutting guides with conventional instrumentation in total knee replacement.	+ The tibial component in the test group was closer to neutral alignment (p = 0.030), but the size of the difference was small (89.8°±1.2° vs 90.5°±1.6°). The AM guides also shortened the bone-cutting time by a 3.6 minutes (p < 0.001) and the operating time by 5.1 minutes (p = 0.019), without differences in post-operative blood loss (p = 0.528) or need for blood transfusion (p = 0.789). These minimal advantages of the AM guides are unlikely to be clinically relevant.
Musculoskeletal	1	Chen	2015	Guide plate for pedicle screw fixation	S	43 (Test=20, Control=23)	Age: Test=35–70 years (mean=52.3 years) Control=37–72 years (mean=55.4 years) Test=9 male, 11 female Control=12 male, 11 female	Clinical Use of 3D Printing Guide Plate in Posterior Lumbar Pedicle Screw Fixation.	Aim: to evaluate the clinical efficacy of use of a 3D printing guide plate in posterior lumbar pedicle screw fixation.	+ The time of placement for each screw was shorter, the amount of hemorrhage was less, and the fluoroscopy times of each screw placement was lower in the test group than in the control group (all p<0.05). The excellent and good screw placement rate was 100% in the test group and 98.4% in the control group, without any statistical difference (p>0.05). No obvious complications were reported in either group.
Musculoskeletal	1	Du	2013	Templates to aid in pin placement in hip resurfacing arthroplasty	S	34 (Test-16, Control=18)	Ages: 37-55 years	Use of patient-specific templates in hip resurfacing arthroplasty: experience from sixteen cases	Aim: To assess whether AM patient-specific templates aid in accurate intraoperative pin placement.	+ The prosthesis stem shaft angle was greater for the test group (p=0.001). The locating template can provide precise and dependable location for hip resurfacing femoral components during arthroplasty and ensure the valgus stem placement necessary for optimal outcomes.
Musculoskeletal	1	Gan	2015	Navigational template for total knee arthroplasty	S	70 (Test=35, Control=35)	Age: Test= 63-75 years (mean=68.5±4.8 years), Control=64-72 years (mean=67.8±3.4 years) Test=10 male, 25 female, Control=9 male, 26 female	Accuracy and efficacy of osteotomy in total knee arthroplasty with patient-specific navigational template	Aim: to validates a novel patient-specific navigational template for total knee arthroplasty.	+ The AM navigational template significantly reduced errors, operation time and blood loss (p<0.05).
Musculoskeletal	1	Hendel	2012	Guide for determining glenoid component position	S	31 (Test=15, Control=16)	No information provided	Comparison of Patient-Specific Instruments with Standard Surgical Instruments in Determining Glenoid Cmponent Position	Aim: to compare patient-specific instruments with standard surgical instruments in determining glenoid component position.	+ The AM technology significantly decreased (p < 0.05) the average deviation of implant position for inclination and medial-lateral offset. The greatest benefit was observed in patients with retroversion >16˚ (p < 0.001).
Musculoskeletal	1	Maini	2016	Plate for acetabulum fracture fixation	P	21 (Test=10, Control=11)	Age: 18-60 years (mean=38.7 years) Test = 9 male, 1 female Control = 9 male, 2 female	Three-dimensional printing and patient-specific pre-contoured plate: future of acetabulum fracture fixation?	Aim: to evaluate the accuracy of patient-specific pre-contoured plates.	+ Reduction achieved, as evaluated by CT scan, was more in the test group than the control group (p<0.05). Reduced blood loss (100 ml less) and surgical time (12 min less) were observed in the test group but the results were not significant (p>0.05). All the pre-contoured plates fitted well to the pelvis intra-operatively.
Musculoskeletal	1	Merc	2013	drill guide for screw placement	S	19 (Test=9 (54 screws), Control=10(54 screws))	Age: Test=59 ± 5 years, Control=62 ±12 years Test=4 male, 5 female, Control=5 male, 5 female	A multi-level rapid prototyping drill guide template reduces the perforation risk of pedicle screw placement in the lumbar and sacral spine	Aim: to evaluate the accuracy of a multi-level drill guide template for lumbar and first sacral pedicle screw placement and to compare it with the freehand technique under fluoroscopy supervision.	+ The incidence of cortex perforation was significantly reduced in the test group. So was the deviation and displacement level of screws in the sagital plane but not in the transverse plane.
Musculoskeletal	1	Shuang	2016	Osteosynthesis Plates	T	13 (Test=6, Control=7)	Age: Test=31-62 years (mean=46.2 years) Control=27-59 years (mean=40.3 years) Test= 4 male, 2 female, Control=6 male, 1 female	Treatment of Intercondylar Humeral Fractures With 3D-Printed Osteosynthesis Plates.	Aim: to evaluate the efficacy custom AM osteosynthesis plates in the treatment of intercondylar humeral fractures.	+ Operative time was shorter for the test group than the control group (p=0.026). The mean time to bone union was 3.4 months. The 2 groups showed no significant difference in the rate of patients with excellent or good elbow function, or in the ranges of elbow flexion/extension and pronation/supination (p>0.05).
Musculoskeletal	1	Stephens	2016	Bone models	P	58 (Test=29, Control=29)	Age: >60 years (mean=73 years) 5 male, 53 female	3-D bone models to improve treatment initiation among patients with osteoporosis: A randomised controlled pilot trial.	Aim: to investigate the efficacy of AM bone models as a tool to facilitate initiation of bisphosphonate treatment among individuals who are newly diagnosed with osteoporosis.	+ The test group were more emotionally affected by osteoporosis immediately after the interview (p=0.04) and reported a greater understanding of osteoporosis at follow-up (p=0.04), than the control group.
Musculoskeletal	1	Wu	2011	Spine model	P	62 (Test=34, Control=28) 677 screws inserted	Age: 4-22 years (mean=11 years)	Accuracy and Safety Assessment of Pedicle Screw Placement Using the Rapid Prototyping Technique in Severe Congenital Scoliosis	Aim: to compare the accuracy and safety of pedicle screw placement in congenital scoliosis using the AM technique versus conventional fluoroscopy.	+ Accuracy of placement = 89.7% (84.7% in control group; 93.5% in test group). Compared with the fluoroscopy method, the AM assisted technique had a shorter operation time and higher scoliosis correction rate (p<0.05).
Musculoskeletal	1	Yang	2016	Model of trimalleolar fracture	P	30 (Test=15, Control=15)	Age: 31-42 years (mean=36.5 years) 16 male, 14 female	Application of 3D Printing in the Surgical Planning of Trimalleolar Fracture and Doctor-Patient Communication.	Aim: to evaluate the effectiveness of using AM models for surgical planning in treating trimalleolar fractures and in physician-patient communication.	+ Both the operation time and the intraoperative blood loss were less in the test group than in the control group (p<0.05). Patient satisfaction with using the AM device for communication was scored highly.
Musculoskeletal	1	You	2016	Proximal humeral fracture model	P	66 (Test=34, Control=32)	61-76 years (mean for Test = 66.09 ± 4.09 years, mean for Control = 66.28 ± 4.10 years) 27 male, 39 female	Application of 3D printing technology on the treatment of complex proximal humeral fractures (Neer3-part and 4-part) in old people.	Aim: to investigate the feasibility and clinical potential of using AM models versus the conventional strategy (thin-layer CT scan) for the treatment of complicated proximal humeral fractures in old people.	+ Reduced surgery duration, less blood loss during surgery, and lower number of fluoroscopy can be observed in the test group compared with the control group (P<0.05). The results showed no significant difference in time to union between the two groups (P>0.05).
Musculoskeletal	1	Zhang	2011a	Acetabular navigational template	S	22 (Test=11, Control=11)	Age: Test=48.6 ± 6.8 years, Control=49.3 ± 4.9 years Test=7 male, 4 female, Control=5 male, 6 female	Preliminary application of computer-assisted patient-specific acetabular navigational template for total hip arthroplasty in adult single development dysplasia of the hip	Aim: to compare a patient-specific AM acetabular navigational template for total hip arthroplasty (THA) to conventional THA in adult single development dysplasia of the hip.	+ The test group showed smaller differences from the predetermined angles than the control group (p<0.05).
Oral and Maxillofacial	2	Arisan	2013	Bone and mucosa supported guides for dental implants	S	54 (Mucosa-supported=12, Bone-supported=15, Control=27)	Age: 28-74 years (mean=52.5 years) 23 male, 31 female	Implant Positioning Errors in Freehand and Computer-Aided Placement Methods: A Single-Blind Clinical Comparative Study	Aim: to compare the dental implant positioning errors related to the use of freehand and computer-aided treatment methods.	+ Interproximal emergence, insufficient interimplant distance, and improper parallelism errors were higher in implants placed using the freehand method (p <0.0001, p <.00001, and p <0.001 respectively).
Oral and Maxillofacial	2	Azuma	2014	Mandible model	S	28 (Test=12, Control=16)	Age: Test=34-91, Control=55-82 18 male, 10 female	Mandibular reconstruction using plates prebent to fit rapid prototyping 3-dimensional printing models ameliorates contour deformity	Aim: To evaluate the clinical efficacy and esthetic outcome of using preoperatively manufactured reconstruction plates, produced using AM models, in mandibular reconstruction.	+ The mandibular symmetry in patients receiving the AM-model-based prebent plates was better than that in patients receiving conventional reconstructive surgery (p<0.05).
Oral and Maxillofacial	2	Brown	2015	Orthodontic bracket system	T	96 (G1: Test=31, G2: Direct bonded conventional=33, G3: Indirect bonded conventional=32)	Age: G1=13.42 years, G2=3.58 years, G3=13.42 years G1 (AM) =17 male, 15 female G2=16 male, 15 female G3=17 male, 16 female	Effectiveness and efficiency of a CAD/CAM orthodontic bracket system.	Aim to retrospectively compare: patients who were indirectly bonded with CAD/CAM self-ligating appliances with those direct bonded with conventional self-ligating appliances, and those indirectly bonded with conventional self-ligating appliances.	+ No statistically significant difference was found on ABO Cast-Radiograph Evaluation scores among the 3 treatment groups (p=0.13). The mean treatment times were different (p<0.05, G1: 13.8 ± 3.4 months, G2: 21.9 ± 5.0 months; G3: 16.9 ± 4.1 months.
Oral and Maxillofacial	2	Calvo-Guirado	2011	Composite powder molding for bone grafts	P	20 (Test=10, Control=10)	No information provided	Calculation of bone graft volume using 3D reconstruction system	Aim: to validate the use of AM bioreplicas for calculating bone volumes prior to planning bone regeneration surgery.	NS No differences were recorded for measurements of bone defect between patient bone and the biological models (p>0.05) but there was also no differences between the test group and control group arising from post-surgical complications observed at the post-operatory fifteen and thirty day examinations.
Oral and Maxillofacial	2	Hanasono	2012	Guide for mandibular reconstruction	S	76 (Test=38, Control=38 defect-matched)	Age:Test=51.0 ± 14.4 years, Control=56.4 ± 15.1 years Test=26 male, 12 female Control=28 male, 10 female	Computer-Assisted Design and Rapid Prototype Modeling in Microvascular Mandible Reconstruction	Aim: to evaluate the use of AM modeling to improve the speed and accuracy of mandibular reconstruction.	+ The operative time for the test group was faster than the operative time defect-matched control group (p=0.0006). The change in position of selected bony landmarks was less in the test group than in the control group (p=0.001).
Oral and Maxillofacial	2	Inokoshi	2012	AM complete dentures	T	10 (Same participants used for test and control)	Age: 78.1 ± 5.6 years	Evaluation of a complete denture trial method applying rapid prototyping	Aim: to compare an AM method for complete dentures with the conventional method.	NS Prosthotontists rated esthetics and stability lower for the AM method (p<0.05), but chair time as faster (p<0.01). No significant differences were found between the two methods in regards to patient scores.
Oral and Maxillofacial	2	Kozakiewicz	2011	Model for developing patient-specific orbital wall implants	P	24 (Test=12, Control=12)	Age: Test=36 ± 10 years, Control=40 ± 20 years Test=9 male, 3 female, Control=9 male, 3 female	Treatment with individual orbital wall implants in humans - 1-Year ophthalmologic evaluation	Aim: to compare the functional results, 1 year after treatment, of using an AM reconstruction method for orbital wall repair with a conventional reconstruction method.	+ At 1 year follow up, the test group had better outcomes than the control group (diplopia: p=0.021, upgaze vertical visual disparity reduction: p=0.007)
Oral and Maxillofacial	2	Lee	2009	AM prefabricated prostheses for cranioplasty	T	131 (Autograft (G1)=91, conventional prosthesis (G2)=23, AM prefabricated prosthesis (G3)=17)	Age: G1=43.84 ± 17.91 years, G2=40.96 ± 15.48 years, G3=40.24 ± 11.97 years G1=62 male, 29 female, G2=14 male, 9 female, G3=12 male, 5 female	Cranioplasty using polymethyl methacrylate prostheses	Aim: to assess whether prefabricated AM prostheses outperform intra-operatively moulded prostheses and perform comparably to the gold-standard of autograft bone flaps in reducing operating time, blood loss and surgical complications in elective delayed cranioplasty operations, after decompressive craniectomy, to repair large (> 100 cm2) cranial defects.	+ G3 patients required less operating time than G2 patients (p<0.001), and operating time did not differ significantly between G1 and G3 (p>0.05). Intra-operative blood loss was lower for G3 than G2 (p=0.015) and did not differ significantly between G1 and G3 (p>0.05). The infection rate for G3 was lower than for G2 and was comparable to G1.
Oral and Maxillofacial	2	Modabber	2012a	Guide for jaw reconstruction	S	10 (Test=5, Control=5)	Age: 37-74 years (mean=52.4 years) 8 male, 2 female	Evaluation of computer-assisted jaw reconstruction with free vascularized fibular flap compared to conventional surgery: a clinical pilot study	Aim: to assess whether the AM method has an influence on the duration of the surgery and the amount of bone removed in fibular reconstruction of maxillary or mandibular defects.	+ Shaping procedure and ischaemic time was shorter for the test group than the control (p=0.014). There was no change between the defect size of the fibula and the necessary transplant size for the test group, but a mean change of 1.92cm occured for the control (p=0.001).
Oral and Maxillofacial	2	Resnick	2016	Surgical splint for orthognathic surgery	T	43 - All had Le Fort I osteotomy and bilateral sagittal split osteotomy (19 in G1: facial symmetry, single-segment, 17 in G2: facial asymmetry, single-segment, and 7 in G3: multi-segment).	All groups compared AM to manual splint fabrication. No information provided on age or gender.	Is There a Difference in Cost Between Standard and Virtual Surgical Planning for Orthognathic Surgery?	Aim: to retrospectively compare time and cost differences between virtual surgical planning and 3D printed orthognathic splints with conventional planning and manufacture techniques.	+ The average times and costs were 194 ± 14.1 minutes and $2,765.94, respectively, for VSP and 540.9 ± 99.5 minutes and $3,519.18, respectively, for standard planning. All time and cost differences were statistically significant (p<0.001).
Oral and Maxillofacial	2	Rogers-Vizena	2016	Craniofacial model	P	29 (Test=9, Control=20)	Age: Test=11.4 ± 3.0 years, Control=11.1 ± 4.7 years Test=4 male, 5 female Control=11 male, 9 female	Cost-Benefit Analysis of Three-Dimensional Craniofacial Models for Midfacial Distraction: A Pilot Study.	Aim: to retrospectively assess the benefit of AM models used in planning surgeries in regards to safety, operative time and cost.	NS 7 complications occurred in 6 of the control group, whereas none occurred in the test group. The test group had a 31.3 minute (7.8%) reduction in operative time (not significant), and time-based cost savings were $1036.
Oral and Maxillofacial	2	Sumida	2015	membranes for bone augmentation in implant treatment	T	26 (Test=13, Control=13)	Age: Test=48.6 ± 9.4 years, Control= 47.8 ± 8.8 years Test=4 male, 9 female, Control=3 male, 10 female	Custom-made titanium devices as membranes for bone augmentation in implant treatment: Clinical application and the comparison with conventional titanium mesh	Aim: to compare titanium AM devices with conventional methods for reconstructing alveolar bone for implantation.	+ There was no significant difference between groups in occurance of mucosal rupture (p=0.27). However, the operation time was shorter for the test group (p<0.01), and fewer screws were used to retain the membrane than the control group (p<0.01).
Oral and Maxillofacial	2	Zhang	2011b	Titanium plate to guide temporomandibular joint reconstruction	S	35 (Test=11, Control=24)	Age: Test=14-65 years (mean=42.3 years), Control=24-56 years (mean=34.9) Test=5 male, 6 female, Control=14 male, 10 female	Application of Rapid Prototyping for Temporomandibular Joint Reconstruction	Aim: to compare the operative time between temporomandibular joint reconstruction surgery using AM guides and conventional surgery.	+ Operative time was shorter for the test group than the control (p<0.05). The outcome of the surgeries was good, with the condyle-incisor, condyle–mental foramen, and condyle-angle from the postoperative CT scan showing no significant difference between the 2 sides (p>0.05).
Musculoskeletal	2	Hirao	2014	Guide for total ankle arthroplasty	S	38 (Test=24, Control=14)	No information provided	Use of a Custom-made Surgical Guide in Total Ankle Arthroplasty in Rheumatoid Arthritis Cases	Aim: to retrospectively compare the accuracy and stability of bone cutting for total ankle arthroplasty when using AM surgical guides to using conventional guides.	+ The error angle of the tibial bone cut was smaller (p = 0.02), and the tilting angle between components was smaller in the test group than the control group (P = 0.01).
Musculoskeletal	2	Iannotti	2015	Template for glenoid implant positioning	P	46 (Test=25, Control=21)	No information provided	Three-Dimensional Imaging and Templating Improve Glenoid Implant Positioning	Aim: to assess whether 3D imaging and the use of patient-specific instrumentation results in more accurate placement of the glenoid component than 3D imaging and standard instrumentation.	NS There was no significant difference between the test group and the control group in achieving the desired implant position, but both performed better than a retrospective sample that used 2D imaging with standard instrumentation.
Musculoskeletal	2	Karlin	2016	Spine model	P	17 (Test=7, Control =10)	Age: Test = 5-14 years (mean=10) Control = 11-18 years (mean=13) Test = 3 male, 4 female Control = 4 male, 6 female	The surgical treatment of spinal deformity in children with myelomeningocele: the role of personalized three-dimensional printed models.	Aim: to retrospectively review children for whom AM spine models were used for surgical planning (Test) and compare them through subjective and objective criteria to a similar group that had no models (Control).	NS The models improved planning and performance of patient-specific spinal instrumentation that was secure and low profile. The efficiency of the surgery as measured by intraoperative fluoroscopy time and blood loss and the extent of the deformity correction was comparable or superior in the test group.
Musculoskeletal	2	Li	2015a	Lumbar vertebral model	P	37 (Test=15, Control=22)	Age: Test=52.36 ± 4.57 years, Control= 50.83 ± 3.60 years Test=9 male, 6 female, Control= 15 male, 7 female	Application of the polystyrene model made by 3-D printing rapid prototyping technology for operation planning in revision lumbar discectomy	Aim: to evaluate the effectiveness of 3-D rapid prototyping technology in revision lumbar discectomy.	+ The test group had a shorter operation time (p<0.001) and less blood loss (p<0.001) than the control group. There was no difference between groups for complication rate or clinical metrics.
Musculoskeletal	2	Li	2016a	Model of pelvis and pelvic fracture	P	157 (Test=81, Control=76)	Age: Test=34.0 ± 8.2 years, Control = 32.2 ± 9.4 years Test=55 male, 26 female, Control=51 male, 25 female	Comparative use of the computer-aided angiography and rapid prototyping technology versus conventional imaging in the management of the Tile C pelvic fractures	Aim to compare the two groups on length of surgical procedure, intra-operative complications, intra- and postoperative blood loss, postoperative pain, postoperative nausea and vomiting (PONV), length of stay, and type of discharge.	+ The test group experienced shorter surgery time (p=0.035), less blood loss (31.0±8.2 g/L vs. 36.2±7.4 g/L), less pain (2.5±2.3 NRS score vs. 2.8±2.0 NRS score), and shorter recoveries (7.8±2.0 days vs. 10.2±3.1 days before discharge).
Musculoskeletal	2	Schweizer	2016	Guide for Reconstructions of Scaphoid Fractures	S	22 (Test=9, Control=13)	Age: Test = 15-47 years (mean=27) Control=14-41 years (mean=24) All male	Computer-Assisted 3-Dimensional Reconstructions of Scaphoid Fractures and Nonunions With and Without the Use of Patient-Specific Guides: Early Clinical Outcomes and Postoperative Assessments of Reconstruction Accuracy.	Aim: to assess the accuracy of the reduction of surgically reconstructed scaphoid nonunions or fractures using 3-dimensional computer-based planning with and without patient-specific guides.	+ In the test group, average residual displacement was 7˚ after surgery, and 8 of 9 scaphoids healed after 2 to 6 months, with partial nonunion after 9 months observed in one patient. In the control group, residual displacement after surgery was 26˚, and 11 of 13 scaphoids healed between 2 and 34 months. 2 scaphoids in the control did not consolidate. The difference in the accuracy of reconstruction was significant (p=0.001). Average operation time was shorter (p=0.01) for the test group.
Musculoskeletal	2	Yang	2014	Upper cervical spine model and navigation template	S	49 (Test=21, Control=28)	Age: 5-81 years (mean=37.6 years) 21 male, 28 female	Clinical Application of Computer-aided Design-Rapid Prototyping in C1–C2 Operation Techniques for Complex Atlantoaxial Instability	Aim: to evaluate the clinical efficacy of AM models in surgical treatments for atlantoaxial instability.	+ The test group had lower intraoperative fluoroscopy frequency (p<0.001), operation time (posterior p=0.001, anterior p=0.004) and blood loss for the posterior approach (p=0.006) than the control group. The screw malposition rate showed no difference between the two groups.
Musculoskeletal	2	Yang	2015	Spine model	P	126 (Test-50, Control=76)	Age: Test=12.52 ± 5.45 years, Control=12.11 ± 6.42 years Test=12 male, 38 female, Control=33 male, 43 female	Application of 3D Rapid Prototyping Technology in Posterior Corrective Surgery for Lenke 1 Adolescent Idiopathic Scoliosis Patients	Aim: to retrospectively evaluate the effectiveness of AM technology in corrective surgery for Lenke 1 adolescent idiopathic scoliosis (AIS) patients based on operation time, perioperative blood loss, transfusion volume, postoperative hemoglobin (Hb), postoperative complications, and length of hospital stay.	+ The test group had shorter operation time, less blood loss and transfusion volume, and higher postoperative Hb (all, P<0.001). However, no significant differences were observed in complication rate, length of hospital stay, and postoperative radiological outcomes between 2 groups (all, P>0.05).
Circulatory	3	Schievano	2007	Model of the right ventricular outflow tract and pulmonary trunk	P	12 (4 did not undergo percutaneous pulmonary valve implantation (PPVI), 4 successfully underwent PPVI, and 4 unsuccessfully underwent PPVI)	Age: 9-39 years (mean 20 years) 7 male, 5 female	Percutaneous Pulmonary Valve Implantation Based on Rapid Prototyping of Right Ventricular Outflow Tract and Pulmonary Trunk from MR Data	Aim: To retrospectively determine if magnetic resonance imaging data can be used to create rigid models that are accurate representations of the right ventricular outflow tract and pulmonary trunk anatomy and if such models can be used to refine the selection of patients for PPVI.	NS Both cardiologists correctly determined from the models that the 4 subjects who did not undergo PPVI were unsuitable for PPVI. Of the 8 subjects who underwent PPVI, only 4 were successful. Both cardiologists correctly identified the suitability of 5 of these subjects from the models. Therefore the cardiologists had a 75% accuracy when selecting patient suitability for PPVI using 3D models.
Oral and Maxillofacial	3	Adolphs	2014	Splint for orthognathic surgery	T	10 (Same participants used for test and control)	No information provided	RapidSplint: virtual splint generation for orthognathic surgery – results of a pilot series	Aim: to demonstrate that clinically usable splints for orthognathic surgery can be produced by computer-assisted technology.	NS 8 of 10 AM splints, and 6 out of conventional splints could be used clinically to achieve and maintain final occlusion after orthognathic surgery. In the five patients where both types of splints were available, their occlusal fitting was assessed as being equivalent, and in one case the AM splint showed better occlusal fitting than the traditional splint.
Oral and Maxillofacial	3	Arisan	2010	Dental screw placement guides	S	54 (28 guides in system I, 32 guides in system II)	Age: 28-73 years (mean=48.4 years) 16 male, 38 female	Accuracy of Two Stereolithographic Guide Systems for Computer-Aided Implant Placement: A Computed Tomography-Based Clinical Comparative Study	Aim: To compare the accuracy of two AM guide systems for dental implant placement.	+ Implants that were placed by bone-supported guides had higher deviations than implants that were placed by mucosa-supported guides (p<0.05) but there was no difference in deviation between implant brands (p <0.08).
Oral and Maxillofacial	3	Morris	2013	Mandible or midface model	P	20 (Test=10, Control=10)	Age: Test=40.5 ± 5.6 years, Control=66.1 ± 3.0 years Test=6 male, 4 female, Control=8 male, 2 female	Utility of Stereolithographic Models in Osteocutaneous Free Flap Reconstruction of the Head and Neck	Aim: to assess whether am AM model decreased the operative time enough to offset the cost of manufacturing the model.	- No significant differences were observed for total operative time between groups, but the total reconstructive time was lower in the control group (p=0.05).
Oral and Maxillofacial	3	Shahbazian	2013	Surgical guide for autotransplantation of teeth	S	40 (Test=20, Control=20)	Age: Test=9-18 years (mean=11 years), Control=9-18 years (mean=12 years) Test=12 male, 8 female, Control=13 male, 7 female	Validation of the cone beam computed tomography-based stereolithographic surgical guide aiding autotransplantation of teeth: clinical case-control study	Aim: to compare the outcome of AM surgical planning and transfer technique for tooth autotransplantation to conventional autotransplantation.	NS 6 from the control group showed 1 or more complications, compared to 2 from the test group.
Oral and Maxillofacial	3	Weitz	2010	Templates for navigated dental implantation	S	10 (Same participants used for test and control)	Age: 19-46 years (mean=29.9 years) 6 male, 4 female	Accuracy of templates for navigated implantation made by rapid prototyping with DICOM datasets of cone beam computer tomography (CBCT)	Aim: to compare the accuracy of AM surgical templates with templates made from conventional impressions for placing implants.	NS The conventional templates enabled excellent fitting accuracy but the accuracy of implants fitted with the AM templates was not sufficient and could result in significant iatrogenic complications.
Musculoskeletal	3	Otsuki	2013	Cutting guide for curved peri-acetabular osteotomy	S	18 (Test=7, Control=11)	Age: No information provided Test=All 7 female, Control= No information provided	Developing a novel custom cutting guide for curved peri-acetabular osteotomy	Aim: to evaluate the efficacy of this new method and cutting guide.	NS The rate of osteotomy-related complications did not differ significantly between the two groups [illegible] almost exactly to the planned cutting line in all patients.

Quality assessment items: 1. Clear hypothesis/aim/objective; 2. Clear outcome measures; 3. Patient characteristics described; 4. Interventions clearly described; 5. Distributions of confounders described; 6. Findings clearly described; 7. Estimates given of random variability; 8. Adverse events reported; 9. Patients lost to follow-up described; 10. Probability values reported; 11. Recruitment pool represents population; 12. Participants represent population; 13. Staff/places/facilities match std. treatment; 14. Participants blinded to intervention; 15. Those measuring outcomes blinded; 16. Data dredging reported; 17. Adjusted for different lengths of follow-up; 18. Appropriate statistical tests; 19. Reliable compliance with intervention; 20. Accurate/reliable outcome measures; 21. Groups recruited from same population; 22. Groups recruited over same timeframe; 23. Subjects randomised into intervention; 24. Randomised intervention concealed; 25. Adjustment for confounding; 26. Losses to follow-up accounted for; 27. Sufficient power

Y = Yes; N = No; U = Unable to determine; NA = Not applicable

First author	Date	1	2	3	4	5	6	7	8	9	10	11	12	13	14	15	16	17	18	19	20	21	22	23	24	25	26	27
de Farias	2014	Y	Y	Y	Y	Y	Y	Y	Y	NA	Y	U	U	Y	Y	Y	Y	NA	Y	Y	Y	Y	Y	Y	Y	U	NA	Y
Ahrberg	2015	Y	Y	Y	Y	Y	Y	Y	N	NA	Y	U	U	Y	Y	Y	Y	NA	Y	Y	Y	Y	Y	Y	Y	Y	NA	Y
D'Urso	1999a	Y	Y	Y	Y	Y	Y	Y	N	NA	N	U	U	Y	N	N	Y	NA	Y	Y	Y	Y	Y	Y	U	N	NA	Y
Al-Ahmad	2013	Y	Y	Y	Y	NA	Y	Y	N	Y	Y	U	U	Y	Y	Y	Y	Y	Y	Y	Y	Y	Y	Y	Y	Y	Y	Y
Ayoub	2014	Y	Y	Y	Y	N	Y	Y	N	NA	N	U	U	Y	N	N	Y	NA	Y	Y	Y	Y	Y	Y	U	N	NA	Y
Goh	2015	Y	Y	Y	Y	N	Y	Y	N	Y	Y	U	U	Y	N	Y	Y	Y	Y	Y	Y	Y	Y	Y	U	N	Y	Y
Van de Velde	2010	Y	Y	Y	Y	N	Y	Y	Y	Y	Y	U	U	Y	N	Y	Y	Y	Y	Y	Y	Y	Y	Y	U	N	Y	Y
Vercruyssen	2014	Y	Y	Y	Y	N	Y	Y	Y	Y	N	U	U	Y	N	Y	Y	Y	Y	Y	Y	Y	Y	Y	U	N	Y	Y
Chareancholvanich	2013	Y	Y	Y	Y	N	Y	Y	Y	Y	Y	U	U	Y	N	Y	Y	N	Y	Y	Y	Y	Y	Y	U	N	Y	Y
Chen	2015	Y	Y	Y	Y	Y	Y	Y	Y	Y	N	U	U	Y	Y	N	Y	Y	Y	Y	Y	Y	Y	Y	Y	Y	Y	Y
Du	2013	Y	Y	N	N	Y	Y	Y	N	Y	Y	U	U	Y	N	N	Y	N	Y	Y	Y	Y	Y	Y	U	N	Y	Y
Gan	2015	Y	Y	Y	Y	Y	Y	Y	Y	NA	Y	U	U	Y	N	Y	Y	NA	Y	Y	Y	Y	Y	Y	U	N	NA	Y
Hendel	2012	Y	Y	N	Y	N	Y	Y	Y	N	Y	Y	Y	Y	N	Y	Y	U	Y	Y	Y	Y	Y	Y	N	N	U	Y
Maini	2016	Y	Y	Y	Y	N	Y	Y	Y	NA	Y	U	U	Y	N	N	Y	NA	Y	Y	Y	Y	Y	Y	U	N	NA	Y
Merc	2013	Y	Y	Y	Y	Y	Y	Y	Y	NA	Y	Y	Y	Y	N	N	Y	NA	Y	Y	Y	Y	Y	Y	U	N	NA	Y
Shuang	2016	Y	Y	N	Y	Y	Y	Y	Y	Y	Y	U	U	Y	N	N	Y	U	Y	Y	Y	Y	Y	Y	Y	Y	Y	Y
Stephens	2016	Y	Y	Y	Y	N	Y	Y	N	Y	Y	U	U	U	Y	Y	Y	Y	Y	Y	Y	Y	Y	Y	Y	N	Y	Y
Wu	2011	Y	Y	N	Y	N	Y	Y	Y	Y	Y	Y	Y	Y	N	N	Y	N	Y	Y	Y	Y	Y	Y	U	N	U	Y
Yang	2016	Y	Y	N	Y	N	Y	Y	Y	NA	Y	U	U	Y	N	N	Y	NA	Y	Y	Y	Y	Y	Y	U	N	NA	Y
You	2016	Y	Y	N	Y	Y	Y	Y	Y	Y	Y	Y	Y	Y	N	N	Y	N	Y	Y	Y	Y	Y	Y	U	Y	Y	Y
Zhang	2011a	Y	Y	N	Y	Y	Y	Y	N	Y	Y	U	U	Y	N	N	Y	N	Y	Y	Y	Y	Y	Y	U	Y	Y	Y
Arisan	2013	Y	Y	Y	Y	Y	Y	Y	N	Y	Y	U	U	Y	N	Y	Y	Y	Y	Y	Y	Y	Y	N	NA	Y	Y	Y
Azuma	2014	Y	Y	Y	Y	N	Y	Y	N	N	N	U	U	Y	N	N	Y	N	Y	Y	Y	U	U	N	NA	N	N	Y
Brown	2015	Y	Y	Y	Y	Y	Y	Y	N	NA	Y	Y	Y	Y	N	Y	Y	NA	Y	Y	Y	U	U	N	NA	Y	NA	Y
Calvo-Guirado	2011	Y	Y	N	Y	N	Y	Y	N	Y	N	U	U	Y	N	N	Y	Y	Y	Y	Y	Y	U	U	U	U	Y	N
Hanasono	2012	Y	Y	N	Y	Y	Y	Y	N	Y	Y	U	U	Y	N	N	Y	N	Y	Y	Y	N	Y	N	NA	N	Y	Y
Inokoshi	2012	Y	Y	Y	Y	N	Y	Y	N	NA	N	U	U	U	N	N	Y	NA	Y	Y	N	NA	NA	N	NA	N	NA	Y
Kozakiewicz	2011	Y	Y	Y	Y	N	Y	N	N	Y	Y	Y	Y	Y	N	N	Y	Y	Y	Y	Y	Y	N	N	NA	N	Y	Y
Lee	2009	Y	Y	Y	Y	N	Y	Y	N	N	Y	Y	Y	Y	Y	Y	Y	N	Y	Y	Y	Y	Y	U	N	U	U	Y
Modabber	2012a	Y	Y	N	Y	N	Y	Y	Y	NA	Y	U	U	Y	N	N	Y	NA	Y	Y	Y	Y	Y	Y	U	N	NA	Y
Resnick	2016	Y	Y	Y	Y	N	Y	Y	N	NA	Y	U	U	Y	N	N	NA	NA	Y	Y	Y	N	N	U	NA	N	NA	Y
Rogers-Vizena	2016	Y	Y	N	Y	N	Y	Y	Y	NA	Y	U	U	Y	N	N	NA	NA	Y	Y	Y	U	U	N	NA	N	NA	N
Sumida	2015	Y	Y	N	N	Y	Y	Y	N	NA	Y	U	U	U	N	N	U	NA	Y	Y	Y	U	U	U	NA	N	NA	Y
Zhang	2011b	N	Y	N	Y	N	N	N	N	Y	N	Y	Y	Y	N	N	Y	U	Y	Y	Y	N	N	N	NA	N	Y	Y
Hirao	2014	N	Y	N	Y	N	Y	Y	Y	Y	Y	U	U	Y	N	N	U	Y	Y	Y	Y	U	U	N	NA	N	Y	Y
Iannotti	2015	Y	Y	N	Y	N	Y	Y	N	NA	Y	U	U	Y	N	Y	Y	NA	Y	Y	Y	N	N	N	U	N	NA	Y
Karlin	2016	Y	Y	N	N	N	Y	N	N	NA	N	U	U	Y	N	N	U	NA	Y	Y	Y	N	U	N	NA	N	NA	N
Li	2015a	Y	Y	Y	Y	Y	Y	Y	Y	Y	Y	U	U	Y	N	N	Y	N	Y	Y	Y	U	Y	N	NA	Y	Y	Y
Li	2016a	Y	Y	Y	Y	Y	Y	Y	Y	NA	Y	Y	Y	Y	N	N	NA	NA	Y	Y	Y	Y	Y	N	NA	Y	NA	Y
Schweizer	2016	Y	Y	N	Y	N	Y	Y	Y	Y	Y	U	U	Y	N	N	Y	N	Y	Y	Y	U	U	N	NA	N	Y	Y
Yang	2014	Y	Y	Y	Y	Y	Y	Y	Y	Y	Y	Y	Y	Y	N	N	Y	Y	Y	Y	Y	U	U	N	NA	N	Y	Y
Yang	2015	Y	Y	Y	Y	Y	Y	Y	Y	Y	Y	U	U	Y	N	N	Y	U	Y	Y	Y	U	Y	N	NA	N	Y	Y
Schievano	2007	Y	Y	N	Y	N	Y	Y	N	NA	N	N	N	Y	N	Y	NA	NA	Y	Y	Y	U	U	N	N	N	NA	N
Adolphs	2014	Y	Y	N	Y	NA	N	N	N	NA	N	U	U	Y	N	N	U	NA	Y	Y	U	NA	NA	NA	NA	NA	NA	N
Arisan	2010	Y	Y	Y	Y	Y	Y	Y	N	N	Y	U	U	Y	N	N	Y	N	Y	Y	Y	Y	U	N	NA	N	Y	Y
Morris	2013	Y	Y	N	Y	Y	Y	Y	N	NA	Y	U	U	Y	N	N	NA	NA	Y	Y	Y	U	U	N	NA	N	NA	Y
Shahbazian	2013	Y	Y	N	Y	Y	Y	N	Y	N	N	U	U	Y	N	Y	Y	N	Y	Y	Y	N	N	N	NA	N	U	N
Weitz	2010	Y	N	N	N	N	Y	Y	Y	NA	N	U	U	U	N	N	U	NA	Y	Y	Y	NA	NA	NA	NA	NA	NA	N
Otsuki	2013	Y	Y	N	Y	N	N	Y	Y	Y	N	U	U	Y	N	N	Y	N	Y	Y	Y	N	U	N	NA	N	Y	N

Medical sector	Level of evidence	First author	Date	Category	No. of participants	Title
Neoplasms	4	Huang	2012	S	31	A digital model individual template and CT-guided 125I seed implants for malignant tumors of the head and neck
Neoplasms	4	Silberstein	2014	P	5	Physical Models of Renal Malignancies Using Standard Cross-sectional Imaging and 3-Dimensional Printers: A Pilot Study
Neoplasms	4	Tao	2010	P	8	Application of computer-aided technique in pelvic precise model preparation and individualized prosthesis implantation
Neoplasms	4	von Rundstedt	2016	P	10	Utility of patient-specific silicone renal models for planning and rehearsal of complex tumour resections prior to robot-assisted laparoscopic partial nephrectomy.
Neoplasms	4	Xiao	2016	P	5	En Bloc Resection of Primary Malignant Bone Tumor in the Cervical Spine Based on 3-Dimensional Printing Technology.
Neoplasms	4	Zhang	2016a	P	10	Evaluation of three-dimensional printing for laparoscopic partial nephrectomy of renal tumors: a preliminary report
Nervous	4	Konrad	2010	S	263	Customized, Miniature Rapid-Prototype Stereotactic Frames for Use in Deep Brain Stimulator Surgery: Initial Clinical Methodology and Experience from 263 Patients from 2002 to 2008
Circulatory	4	Farooqi	2016	P	6	Application of Virtual Three-Dimensional Models for Simultaneous Visualization of Intracardiac Anatomic Relationships in Double Outlet Right Ventricle.
Circulatory	4	Gilon	2002	P	35	Effect of Three-Dimensional Valve Shape on the Hemodynamics of Aortic Stenosis: Three-Dimensional Echocardiographic Stereolithography and Patient Studies
Circulatory	4	Jacobs	2008	P	3	3D-Imaging of cardiac structures using 3D heart models for planning in heart surgery: a preliminary study
Circulatory	4	Liu	2016b	P	8	The Value of 3D Printing Models of Left Atrial Appendage Using Real-Time 3D Transesophageal Echocardiographic Data in Left Atrial Appendage Occlusion: Applications toward an Era of Truly Personalized Medicine.
Circulatory	4	Ngan	2006	P	4	The rapid prototyping of anatomic models in pulmonary atresia
Circulatory	4	Ripley	2016	P	16	3D printing based on cardiac CT assists anatomic visualization prior to transcatheter aortic valve replacement.
Circulatory	4	Weinstock	2015	P	4	Optimizing cerebrovascular surgical and endovascular procedures in children via personalized 3D printing
Digestive	4	Ko	2016	S	35	Novel 3D-printing technique for caps to enable tailored therapeutic endoscopy
Digestive	4	Zein	2013	P	6	Three-Dimensional Print of a Liver for Preoperative Planning in Living Donor Liver Transplantation
Oral and Maxillofacial	4	Asaumi	2001	P	2	Comparison of three-dimensional computed tomography with rapid prototype models in the management of coronoid hyperplasia
Oral and Maxillofacial	4	Barakat	2013	S	6	Clinical and radiographic evaluation of a computer-generated guiding device in bilateral sagittal split osteotomies
Oral and Maxillofacial	4	Bosc	2017	S	18	Mandibular reconstruction after cancer: an in-house approach to manufacturing cutting guides
Oral and Maxillofacial	4	Cassetta	2013	T	20	Accuracy of Two Stereolithographic Surgical Templates: A Retrospective Study
Oral and Maxillofacial	4	Chai	2015	P	7	Treating Parry-Romberg Syndrome Using Three-Dimensional Scanning and Printing and the Anterolateral Thigh Dermal Adipofascial Flap
Oral and Maxillofacial	4	Chan	2015	S	10	3D Rapid Prototyping for Otolaryngology—Head and Neck Surgery: Applications in Image-Guidance, Surgical Simulation and Patient-Specific Modeling
Oral and Maxillofacial	4	Chen	2014	P	6	Three-Dimensional Preoperative Design of Distraction Osteogenesis for Hemifacial Microsomia
Oral and Maxillofacial	4	Chen	2010	S	4	Modular Preoperative Planning Software for Computer-Aided Oral Implantology and the Application of a Novel Stereolithographic Template: A Pilot Study
Oral and Maxillofacial	4	Ciprandi	2012	T	5	Calcium Phosphate Cement in Orbital Reconstructions
Oral and Maxillofacial	4	Clelland	2016	S	18	Split-mouth comparison of splinted and nonsplinted prostheses on short implants: 3-year results
Oral and Maxillofacial	4	Cornelius	2016	S	12	Iterations of computer- and template assisted mandibular or maxillary reconstruction with free flaps containing the lateral scapular border-Evolution of a biplanar plug-on cutting guide.
Oral and Maxillofacial	4	Cui	2014	P	3	Surgical Planning, Three-Dimensional Model Surgery and Preshaped Implants in Treatment of Bilateral Craniomaxillofacial Post-Traumatic Deformities
Oral and Maxillofacial	4	Curcio	2007	P	14	Use of models in surgical predictability of oral rehabilitations
Oral and Maxillofacial	4	Dérand	2012	S	4	Imaging, Virtual Planning, Design, and Production of Patient-Specific Implants and Clinical Validation in Craniomaxillofacial Surgery
Oral and Maxillofacial	4	D'haese	2012	S	13	A Prospective Study on the Accuracy of Mucosally Supported Stereolithographic Surgical Guides in Fully Edentulous Maxillae
Oral and Maxillofacial	4	Di Giacomo	2011	S	12	Accuracy and Complications of Computer-Designed Selective Laser Sintering Surgical Guides for Flapless Dental Implant Placement and Immediate Definitive Prosthesis Installation
Oral and Maxillofacial	4	Di Giacomo	2005	S	4	Clinical Application of Stereolithographic Surgical Guides for Implant Placement: Preliminary Results
Oral and Maxillofacial	4	Di Giacomo	2016	S	9	A selective laser sintering prototype guide used to fabricate immediate interim fixed complete arch prostheses in flapless dental implant surgery: Technique description and clinical results.
Oral and Maxillofacial	4	El-Gengehi	2015	S	24	Evaluation of the Accuracy of Computer-Guided Mandibular Fracture Reduction
Oral and Maxillofacial	4	Ersoy	2008	S	21	Reliability of implant placement with stereolithographic surgical guides generated from computed tomography: clinical data from 94 implants
Oral and Maxillofacial	4	Fan	2007	P	17	Late Reconstruction of the Complex Orbital Fractures With Computer-Aided Design and Computer-Aided Manufacturing Technique
Oral and Maxillofacial	4	Feng	2011	S	4	Mirror imaging and preshaped titanium plates in the treatment of unilateral malar and zygomatic arch fractures
Oral and Maxillofacial	4	Fiaschi	2016	T	12	Surgical results of cranioplasty with a polymethylmethacrylate customized cranial implant in pediatric patients: a single-center experience.
Oral and Maxillofacial	4	Gaggl	1998	P	3	Treatment planning for sinus lift augmentations through use of 3-dimensional milled models derived from computed tomography scans
Oral and Maxillofacial	4	Guo	2009	T	61	Reconstruction of Orbital Floor Fractures
Oral and Maxillofacial	4	Huang	2017	P	97	Personalized Reconstruction of Traumatic Orbital Defects Based on Precise Three-Dimensional Orientation and Measurements of the Globe.
Oral and Maxillofacial	4	Jiang	2015	T	18	Functional Evaluation of a CAD/CAM Prosthesis for Immediate Defect Repair after Total Maxillectomy: A Case Series of 18 Patients with Maxillary Sinus Cancer
Oral and Maxillofacial	4	Jiao	2014	T	11	Rehabilitation of maxillectomy defects with obturator prostheses fabricated using computer-aided design and rapid prototyping: a pilot study.
Oral and Maxillofacial	4	Kim	2004	P	182	Evaluation of the prognosis and causes of failure in 182 cases of autogenous tooth transplantation
Oral and Maxillofacial	4	Kocyiğit	2013	S	10	Evaluation of Preoperative Model Surgery and the Use of a Maxillary Sinus Surgical Template in Sinus Floor Augmentation Surgery
Oral and Maxillofacial	4	Komiyama	2012	S	29	Soft Tissue Conditions and Marginal Bone Changes around Immediately Loaded Implants Inserted in Edentate Jaws Following Computer Guided Treatment Planning and Flapless Surgery: A 31-Year Clinical Follow-Up
Oral and Maxillofacial	4	Kozakiewicz	2009	P	6	Clinical application of 3D pre-bent titanium implants for orbital floor fractures
Oral and Maxillofacial	4	Labadie	2008	S	4	Clinical Validation of Percutaneous Cochlear Implant Surgery: Initial Report
Oral and Maxillofacial	4	Lethaus	2010	T	11	Surgical and prosthetic reconsiderations in patients with maxillectomy
Oral and Maxillofacial	4	Li	2013a	T	6	Digital Design and Individually Fabricated Titanium Implants for the Reconstruction of Traumatic Zygomatico-Orbital Defects
Oral and Maxillofacial	4	Li	2015b	T	12	Clinical Feasibility and Efficacy of Using Virtual Surgical Planning in Bimaxillary Orthognathic Surgery Without Intermediate Splint
Oral and Maxillofacial	4	Li	2013b	S	6	A novel method of computer aided orthognathic surgery using individual CAD/CAM templates: a combination of osteotomy and repositioning guides
Oral and Maxillofacial	4	Li	2016c	S	7	Treatment of Dentofacial Deformities Secondary to Osteochondroma of the Mandibular Condyle Using Virtual Surgical Planning and 3-Dimensional Printed Surgical Templates
Oral and Maxillofacial	4	Li	2016b	S	9	A new design of CAD/CAM surgical template system for two-piece narrowing genioplasty.
Oral and Maxillofacial	4	Lin	2016	S	5	Mandibular angle split osteotomy based on a novel augmented reality navigation using specialized robot-assisted arms--A feasibility study.
Oral and Maxillofacial	4	Lieger	2010	P	29	Computer-Assisted Design and Manufacture of Implants in the Late Reconstruction of Extensive Orbital Fractures
Oral and Maxillofacial	4	Liu	2010	S	11	Accuracy of surgical positioning of orthodontic miniscrews with a computer-aided design and manufacturing template
Oral and Maxillofacial	4	Liu	2013	S	8	Digital Surgical Templates for Managing High-Energy Zygomaticomaxillary Complex Injuries Associated With Orbital Volume Change: A Quantitative Assessment
Oral and Maxillofacial	4	Liu	2009	S	7	Applying Computer Techniques in Maxillofacial Reconstruction Using a Fibula Flap: A Messenger and an Evaluation Method
Oral and Maxillofacial	4	Liu	2014a	S	15	Technical procedures for template-guided surgery for mandibular reconstruction based on digital design and manufacturing
Oral and Maxillofacial	4	Martorelli	2012	S	6	A comparison between customized clear and removable orthodontic appliances manufactured using RP and CNC techniques
Oral and Maxillofacial	4	Mazzoni	2015	S	10	Computer-Aided Design and Computer-Aided Manufacturing Cutting Guides and Customized Titanium Plates Are Useful in Upper Maxilla Waferless Repositioning
Oral and Maxillofacial	4	Modabber	2012a	S	15	Computer-assisted Mandibular Reconstruction with Vascularized Iliac Crest Bone Graft
Oral and Maxillofacial	4	Morea	2011	S	4	Precise Insertion of Orthodontic Miniscrews with a Stereolithographic Surgical Guide Based on Cone Beam Computed Tomography Data: A Pilot Study
Oral and Maxillofacial	4	Muller	2003	P	52	The Application of Rapid Prototyping Techniques in Cranial Reconstruction and Preoperative Planning in Neurosurgery
Oral and Maxillofacial	4	Murray	2008	P	3	Advanced technology in the management of fibrous dysplasia
Oral and Maxillofacial	4	Ozan	2011	S	54	Correlation Between Bone Density and Angular Deviation of Implants Placed Using CT-Generated Surgical Guides
Oral and Maxillofacial	4	Ozan	2009	S	30	Clinical Accuracy of 3 Different Types of Computed Tomography-Derived Stereolithographic Surgical Guides in Implant Placement
Oral and Maxillofacial	4	Papaspyridakos	2012a	T	14	Computer-assisted design/computer-assisted manufacturing zirconia implant fixed complete prostheses: clinical results and technical complications up to 4 years of function
Oral and Maxillofacial	4	Park	2015	P	127	Mirror-Imaged Rapid Prototype Skull Model and Pre-Molded Synthetic Scaffold to Achieve Optimal Orbital Cavity Reconstruction
Oral and Maxillofacial	4	Park	2016	T	21	Cranioplasty Enhanced by Three-Dimensional Printing: Custom-Made Three-Dimensional-Printed Titanium Implants for Skull Defects.
Oral and Maxillofacial	4	Pettersson	2010	S	25	Accuracy of Virtually Planned and Template Guided Implant Surgery on Edentate Patientscid_285 527..537
Oral and Maxillofacial	4	Prisman	2014	P	10	Value of preoperative mandibular plating in reconstruction of the mandible
Oral and Maxillofacial	4	Reiser	2015	S	17	V-Stand—A Versatile Surgical Platform for Oromandibular Reconstruction Using a 3-Dimensional Virtual Modeling System
Oral and Maxillofacial	4	Rotaru	2012	T	10	Cranioplasty With Custom-Made Implants: Analyzing the Cases of 10 Patients
Oral and Maxillofacial	4	Sailer	1998	P	20	The value of stereolithographic models for preoperative diagnosis of craniofacial deformities and planning of surgical corrections
Oral and Maxillofacial	4	Schantz	2006	T	5	Cranioplasty after trephination using a novel biodegradable burr hole cover: technical case report
Oral and Maxillofacial	4	Scolozzi	2009	P	10	Accuracy and Predictability in Use of AO Three-Dimensionally Preformed Titanium Mesh Plates for Posttraumatic Orbital Reconstruction: A Pilot Study
Oral and Maxillofacial	4	Shan	2015	T	2	Surgical Reconstruction of Maxillary and Mandibular Defects Using a Printed Titanium Mesh
Oral and Maxillofacial	4	Shehab	2013	S	6	A novel design of a computer-generated splint for vertical repositioning of the maxilla after Le Fort I osteotomy
Oral and Maxillofacial	4	Shen	2015	P	17	Presurgical Nasoalveolar Molding for Cleft Lip and Palate: The Application of Digitally Designed Molds
Oral and Maxillofacial	4	Shu	2014	S	8	Accuracy of using computer-aided rapid prototyping templates for mandible reconstruction with an iliac crest graft
Oral and Maxillofacial	4	Staffa	2006	S	26	Custom made cranioplasty prostheses in porous hydroxy-apatite using 3D design techniques: 7 years experience in 25 patients
Oral and Maxillofacial	4	Stubinger	2014	S	10	Deviations between Placed and Planned Implant Positions: An Accuracy Pilot Study of Skeletally Supported Stereolithographic Surgical Templates
Oral and Maxillofacial	4	Sun	2013a	S	15	Accuracy of Upper Jaw Positioning With Intermediate Splint Fabrication After Virtual Planning in Bimaxillary Orthognathic Surgery
Oral and Maxillofacial	4	Sun	2013b	S	6	Error analysis of a CAD/CAM method for unidirectional mandibular distraction osteogenesis in the treatment of hemifacial microsomia
Oral and Maxillofacial	4	Suomalainen	2015	P	102	Rapid prototyping modelling in oral and maxillofacial surgery: A two year retrospective study.
Oral and Maxillofacial	4	Tabaković	2015	T	10	Application of Computer-Aided Designing and Rapid Prototyping Technologies in Reconstruction of Blowout Fractures of the Orbital Floor
Oral and Maxillofacial	4	Tang	2010	P	46	Individual Design and Rapid Prototyping in Reconstruction of Orbital Wall Defects
Oral and Maxillofacial	4	Turgut	2012	T	11	Computer-Aided Design and Manufacture and Rapid Prototyped Polymethylmethacrylate Reconstruction
Oral and Maxillofacial	4	Valente	2007	S	25	Accuracy and clinical outcomes of computer aided oral implant surgery: a preliminary study on 25 patients
Oral and Maxillofacial	4	Van Assche	2010	S	8	Accuracy assessment of computer-assisted flapless implant placement in partial edentulism
Oral and Maxillofacial	4	Verhamme	2012	S	5	A clinically relevant validation method for implant placement after virtual planning
Oral and Maxillofacial	4	Vrielinck	2003	S	12	Image-based planning and clinical validation of zygoma and pterygoid implant placement in patients with severe bone atrophy using customized drill guides. Preliminary results from a prospective clinical follow-up study
Oral and Maxillofacial	4	Wang	2013a	P	10	Three-dimensional virtual technology in reconstruction of mandibular defect including condyle using double-barrel vascularized fibula flap
Oral and Maxillofacial	4	Wiggins	2013	S	113	Cranioplasty With Custom-Made Titanium Plates—14 Years Experience
Oral and Maxillofacial	4	Won	2013	P	21	Improving pre-operative planning for complex total hip replacement with a Rapid Prototype model enabling surgical simulation
Oral and Maxillofacial	4	Wong	2005	S	13	Comparison of 2 Methods of Making Surgical Models for Correction of Facial Asymmetry
Oral and Maxillofacial	4	Xu	2015a	S	24	Combined Use of Rapid-Prototyping Model and Surgical Guide in Correction of Mandibular Asymmetry Malformation Patients With Normal Occlusal Relationship
Oral and Maxillofacial	4	Yin	2014	S	36	Mandibular Distraction Combined With Orthognathic Techniques for the Correction of Severe Adult Mandibular Hypoplasia
Oral and Maxillofacial	4	Ying	2015	S	14	Correction of facial asymmetry associated with vertical maxillary excess and mandibular prognathism by combined orthognathic surgery and guiding templates and splints fabricated by rapid prototyping technique
Oral and Maxillofacial	4	Yu	2013	P	15	CAD presurgical nasoalveolar molding effects on the maxillary morphology in infants with UCLP
Oral and Maxillofacial	4	Yuan	2013	T	10	Accuracy evaluation of a new three-dimensional reproduction method of edentulous dental casts, and wax occlusion rims with jaw relation
Oral and Maxillofacial	4	Zhang	2015	T	12	Computer-Aided Design and Computer-Aided Manufacturing Hydroxyapatite/Epoxide Acrylate Maleic Compound Construction for Craniomaxillofacial Bone Defects
Oral and Maxillofacial	4	Zhang	2009	S	6	Application of navigation template to fixation of sacral fracture using three-dimensional reconstruction and reverse engineering technique
Oral and Maxillofacial	4	Zhang	2016b	P	30	Accuracy of virtual surgical planning in two-jaw orthognathic surgery: comparison of planned and actual results
Oral and Maxillofacial	4	Zheng	2012	T	9	Mandible reconstruction assisted by preoperative virtual surgical simulation
Oral and Maxillofacial	4	Zhong	2015	T	10	Quantitative Analysis of Dual-Purpose, Patient- Specific Craniofacial Implants for Correction of Temporal Deformity
Oral and Maxillofacial	4	Zinser	2012	S	8	Computer-assisted orthognathic surgery: feasibility study using multiple CAD/CAM surgical splints
Oral and Maxillofacial	4	Zou	2015	S	6	Application of Computer Techniques in Correcting Mild Zygomatic Assymetry With Unilateral Reduction Malarplasty
Musculoskeletal	4	Bauer	2015	P	19	Preoperative Computer Simulation and Patient-specific Guides are Safe and Effective to Correct Forearm Deformity in Children
Musculoskeletal	4	Chen	2016a	S	5	A novel navigation template for fixation of acetabular posterior column fractures with antegrade lag screws: design and application
Musculoskeletal	4	Dai	2007	T	10	Computer-Aided Custom-Made Hemipelvic Prosthesis Used in Extensive Pelvic Lesions
Musculoskeletal	4	Deng	2016	S	10	The accuracy and the safety of individualized 3D printing screws insertion templates for cervical screw insertion.
Musculoskeletal	4	Dhanshyani	2011	P	6	Rapid prototyping models for dysplastic hip surgeries in Malaysia
Musculoskeletal	4	Guarino	2007	P	13	Rapid Prototyping Technology for Surgeries of the Pediatric Spine and Pelvis
Musculoskeletal	4	Hananouchi	2009	S	24	Tailor-made surgical guide based on rapid prototyping technique for cup insertion in total hip arthroplasty
Musculoskeletal	4	Hsieh	2010	P	12	Repositioning Osteotomy for Intra-Articular Malunion of Distal Radius With Radiocarpal and/or Distal Radioulnar Joint Subluxation
Musculoskeletal	4	Hu	2016	S	151	A comparative study on the accuracy of pedicle screw placement assisted by personalized rapid prototyping template between pre- and post-operation in patients with relatively normal mid-upper thoracic spine.
Musculoskeletal	4	Jiang	2016	S	32	Accuracy assessment of atlantoaxial pedicle screws assisted by a novel drill guide template.
Musculoskeletal	4	Kaneyama	2015	S	20	Safe and Accurate Midcervical Pedicle Screw Insertion Procedure With the Patient-Specifi c Screw Guide Template System
Musculoskeletal	4	Kawaguchi	2012	S	11	Development of a New Technique for Pedicle Screw and Magerl Screw Insertion Using a 3-Dimensional Image Guide
Musculoskeletal	4	Kunz	2010	P	45	Computer-Assisted Hip Resurfacing Using Individualized Drill Templates
Musculoskeletal	4	Kunz	2011	P	80	Registration stability of physical tem-plates in hip surgery
Musculoskeletal	4	Li	2013c	P	25	Revision of Complex Acetabular Defects Using Cages with the Aid of Rapid Prototyping.
Musculoskeletal	4	Li	2015c	T	26	Custom Acetabular Cages Offer Stable Fixation and Improved Hip Scores for Revision THA With Severe Bone Defects
Musculoskeletal	4	Lin	2013	P	10	Application of three-dimensional model in the management of irreducible atlanto-axial dislocation
Musculoskeletal	4	Liu	2016a	S	10	Preliminary application of a multi-level 3D printing drill guide template for pedicle screw placement in severe and rigid scoliosis.
Musculoskeletal	4	Lu	2009a	S	25	A Novel Patient-Specific Navigational Template for Cervical Pedicle Screw Placement
Musculoskeletal	4	Lu	2012	S	16	Accuracy and efficacy of thoracic pedicle screws in scoliosis with patient-specific drill template
Musculoskeletal	4	Mao	2010	P	16	Clinical application of computer-designed polystyrene models in complex severe spinal deformities: a pilot study
Musculoskeletal	4	Miyake	2012	S	20	Computer-Assisted Corrective Osteotomy for Malunited Diaphyseal Forearm Fractures
Musculoskeletal	4	Murase	2005	P	7	Does Three-dimensional Computer Simulation Improve Results of Scaphoid Nonunion Surgery?
Musculoskeletal	4	Murase	2008	S	22	Three-Dimensional Corrective Osteotomy of Malunited Fractures of the Upper Extremity with Use of a Computer Simulation System
Musculoskeletal	4	Parchi	2013	P	8	Computer Tomography Prototyping and Virtual Procedure Simulation in Difficult Cases of Hip Replacement Surgery
Musculoskeletal	4	Putzier	2014	S	4	A New Navigational Tool for Pedicle Screw Placement in Patients with Severe Scoliosis: A Pilot Study to Prove Feasibility, Accuracy, and Identify Operative Challenges.
Musculoskeletal	4	Schweizer	2013	S	6	Three-Dimensional Correction of Distal Radius Intra-Articular Malunions Using Patient-Specific Drill Guides
Musculoskeletal	4	Takemoto	2016	S	36	Additive-manufactured patient-specific titanium templates for thoracic pedicle screw placement: novel design with reduced contact area.
Musculoskeletal	4	Takeyasu	2013	S	30	Preoperative, Computer Simulation-Based, Three-Dimensional Corrective Osteotomy for Cubitus Varus Deformity with Use of a Custom-Designed Surgical Device
Musculoskeletal	4	Tricot	2012	S	3	3D-corrective osteotomy using surgical guides for posttraumatic distal humeral deformity
Musculoskeletal	4	Yang	2011	S	21	Personalised modified osteotomy using computer-aided design–rapid prototyping to correct thoracic deformities
Musculoskeletal	4	Zeng	2015	P	38	Evaluation of three-dimensional printing for internal fixation of unstable pelvic fracture from minimal invasive para-rectus abdominis approach: a preliminary report
Musculoskeletal	4	Zhang	2011c	S	18	Application of computer-aided design osteotomy template for treatment of cubitus varus deformity in teenagers: A pilot study
Musculoskeletal	4	Zhuang	2015	P	14	Surgical Treatment of Acetabulum Top Compression Fracture with Sea Gull Sign
Musculoskeletal	4	Hurson	2007	P	20	Rapid prototyping in the assessment, classification and preoperative planning of acetabular fractures
Musculoskeletal	4	Kunz	2012	S	31	Hip resurfacing with individualized drill templates— comparison between anterolateral and posterior approach
Musculoskeletal	4	Xu	2015b	P	10	Application of Rapid Prototyping Pelvic Model for Patients with DDH to Facilitate Arthroplasty Planning: A Pilot Study.
Musculoskeletal	4	Zeng	2016	P	10	A combination of three-dimensional printing and computer-assisted virtual surgical procedure for preoperative planning of acetabular fracture reduction.
External Devices	4	Fabry	2002	T	43	Hearing aid physical fit: The next revolution?
External Devices	4	Gibson	2014	T	15	Functionally Optimized Orthoses for Early Rheumatoid Arthritis Foot Disease: A Study of Mechanisms and Patient Experience
External Devices	4	Liu	2014b	T	10	Newly Designed Foot Orthosis for Children with Residual Clubfoot After Ponseti Casting
External Devices	4	Pallari	2010	T	7	Mass Customization of Foot Orthoses for Rheumatoid Arthritis Using Selective Laser Sintering
External Devices	4	Raux	2014	T	30	Tridimensional trunk surface acquisition for brace manufacturing in idiopathic scoliosis
External Devices	4	Telfer	2013a	T	24	Dose-response effects of customised foot orthoses on lower limb kinematics and kinetics in pronated foot type
External Devices	4	Telfer	2013b	T	12	Dose-response effects of customised foot orthoses on lower limb muscle activity and plantar pressures in pronated foot type
Neoplasms	5	Akiba	2015	P	1	A Three-Dimensional Mediastinal Model Created with Rapid Prototyping in a Patient with Ectopic Thymoma
Neoplasms	5	D'Urso	1999b	P	1	Biomodel guided Stereotaxy
Neoplasms	5	Krauel	2016	P	3	Use of 3D Prototypes for Complex Surgical Oncologic Cases
Neoplasms	5	Mainenti	2009	P	1	Ameloblastic fibro-odontosarcoma: a case report
Neoplasms	5	Wong	2015	T	1	One-step reconstruction with a 3D-printed, biomechanically evaluated custom implant after complex pelvic tumor resection
Neoplasms	5	Wiebe	2015	S	1	Customized vaginal vault brachytherapy with computed tomography imaging-derived applicator prototyping
Nervous	5	D'Urso	1999c	P	16	Cerebrovascular biomodelling: a technical note
Circulatory	5	Bosi	2015	P	1	Patient-Specific Finite Element Models to Support Clinical Decisions: A Lesson Learnt from a Case Study of Percutaneous Pulmonary Valve Implantation
Circulatory	5	Dankowski	2014	P	1	3D heart model printing for preparation of percutaneous structural interventions: description of the technology and case report
Circulatory	5	Ejaz	2014	S	3	Color-coded patient-specific physical models of congenital heart disease
Circulatory	5	Erbano	2013	P	3	Rapid prototyping of three-dimensional biomodels as an adjuvant in the surgical planning for intracranial aneurysms
Circulatory	5	Gosnell	2016	P	1	Integration of Computed Tomography and Three-Dimensional Echocardiography for Hybrid Three-Dimensional Printing in Congenital Heart Disease.
Circulatory	5	Hermsen	2017	P	2	Scan, plan, print, practice, perform: Development and use of a patient-specific 3-dimensional printed model in adult cardiac surgery.
Circulatory	5	Kiraly	2016	P	1	Three-dimensional printed prototypes refine the anatomy of post-modified Norwood-1 complex aortic arch obstruction and allow presurgical simulation of the repair.
Circulatory	5	Lan	2016	P	1	Development of Three-Dimensional Printed Craniocerebral Models for Simulated Neurosurgery.
Circulatory	5	Mottle-link	2008	P	1	Physical Models Aiding in Complex Congenital Heart Surgery
Circulatory	5	Pepper	2013	S	34	Implantation of an individually computer-designed and manufactured external support for the Marfan aortic root
Circulatory	5	Riesenkampff	2009	P	11	The practical clinical value of three-dimensional models of complex congenitally malformed hearts
Circulatory	5	Sodian	2009	P	1	3-Dimensional Printing of Models to Create Custom-Made Devices for Coil Embolization of an Anastomotic Leak After Aortic Arch Replacement
Circulatory	5	Sardari Nia	2016	P	1	Preoperative planning with three-dimensional reconstruction of patient's anatomy, rapid prototyping and simulation for endoscopic mitral valve repair.
Circulatory	5	Shimamura	2012	P	4	Three-Dimensional Replica of a Life-Sized Model of Aortic Arch Aneurysm for Preoperative Assessments
Circulatory	5	Tam	2013	P	1	3D Printing of an Aortic Aneurysm to Facilitate Decision Making and Device Selection for Endovascular Aneurysm Repair in Complex Neck Anatomy
Circulatory	5	Vranicar	2008	P	12	The use of stereolithographic hand held models for evaluation of congenital anomalies of the great arteries
Circulatory	5	Wurm	2004	P	13	Cerebrovascular stereolithographic biomodeling for aneurysm surgery
Respiratory	5	Booysen	2006	S	1	Anaesthetic mouthpiece development through QFD and customer interaction with functional prototypes
Skin	5	Chae	2015	P	1	3D-Printed haptic "Reverse" models for preoperative planning in soft tissue reconstruction: A case report
Skin	5	Wei	2016	T	2	3D-printed transparent facemasks in the treatment of facial hypertrophic scars of young children with burns.
Oral and Maxillofacial	5	Abe	1998	P	7	Model-based surgical planning and simulation of cranial base surgery
Oral and Maxillofacial	5	Abou-ElFetouh	2011	S	2	Computer-guided rapid-prototyped templates for segmental mandibular osteotomies: a preliminary report
Oral and Maxillofacial	5	Adolphs	2013	P	2	Craniomaxillofacial surgery planning based on 3D models derived from Cone-Beam CT data
Oral and Maxillofacial	5	Aita-Holmes	2015	T	1	Digital capture, design, and manufacturing of an extraoral device for a clarinet player with Bell's palsy
Oral and Maxillofacial	5	Aitasalo	2014	T	12	Craniofacial bone reconstruction with bioactive fiber-reinforced composite implant
Oral and Maxillofacial	5	Bai	2012	T	7	An Alternative Approach for Mandible Reconstruction
Oral and Maxillofacial	5	Bai	2010	S	1	CAD/CAM surface templates as an alternative to the intermediate wafer in orthognathic surgery
Oral and Maxillofacial	5	Bellini	2007	P	1	Bi-directional distraction in the treatment of micro-orbitism: A case report
Oral and Maxillofacial	5	Bibb	2006	T	1	Trial fitting of a removable partial denture framework made using computer-aided design and rapid prototyping techniques
Oral and Maxillofacial	5	Brie	2012	T	8	A new custom made bioceramic implant for the repair of large and complex craniofacial bone defects
Oral and Maxillofacial	5	Byun	2015	S	1	Endodontic Treatment of an Anomalous Anterior Tooth with the Aid of a 3-dimensional Printed Physical Tooth Model
Oral and Maxillofacial	5	Cao	2010	T	39	Application of EH Compound Artificial Bone Material Combined With Computerized Three-Dimensional Reconstruction in Craniomaxillofacial Surgery
Oral and Maxillofacial	5	Cassetta	2016	S	1	Accelerating orthodontic tooth movement: A new, minimally-invasive corticotomy technique using a 3D-printed surgical template.
Oral and Maxillofacial	5	Cesarano	2005	T	1	Customization of Load-Bearing Hydroxyapatite Lattice Scaffolds
Oral and Maxillofacial	5	Cho	2013	T	1	Mirror-image anterior crown fabrication with computer-aided design and rapid prototyping technology: A clinical report
Oral and Maxillofacial	5	Chow	2006	S	5	Zygomatic Implants—Protocol for Immediate Occlusal Loading: A Preliminary Report
Oral and Maxillofacial	5	Chua	1998	P	1	Rapid Prototyping Assisted Surgery Planning
Oral and Maxillofacial	5	Ciocca	2010a	T	1	Immediate facial rehabilitation in cancer patients using CAD–CAM and rapid prototyping technology: a pilot study
Oral and Maxillofacial	5	Ciocca	2010b	S	1	Rehabilitation of the nose using CAD-CAM and rapid prototyping technology after ablative surgery of squamous cell carcinoma: A pilot clinical report
Oral and Maxillofacial	5	Ciocca	2012	S	1	CAD/CAM guided secondary mandibular reconstruction of a discontinuity defect after ablative cancer surgery
Oral and Maxillofacial	5	Da Rosa	2004	P	1	Rapid Prototyping in Maxillofacial Surgery and Traumatology: Case Report
Oral and Maxillofacial	5	Elbashti	2016	T	1	The Role of Digitization in the Rapid Reproduction of an Obturator in a Frail Elderly Patient.
Oral and Maxillofacial	5	Fang	2013	S	2	Complex Facial Deformity Reconstruction With a Surgical Guide Incorporating a Built-in Occlusal Stent as the Positioning Reference
Oral and Maxillofacial	5	Feng	2010	P	1	Mirror imaging and preshaped titanium plates in the treatment of unilateral malar and zygomatic arch fractures
Oral and Maxillofacial	5	Fernandes	2007	P	1	Computer-aided surgery using 3D rendering of maxillofacial pathology and trauma
Oral and Maxillofacial	5	Figliuzzi	2012	T	1	A novel root analogue dental implant using CT scan and CAD/CAM: selective laser melting technology
Oral and Maxillofacial	5	Flugge	2013	S	1	Three-Dimensional Plotting and Printing of an Implant Drilling Guide: Simplifying Guided Implant Surgery
Oral and Maxillofacial	5	Goh	2009	T	31	Customised fabricated implants after previous failed cranioplasty
Oral and Maxillofacial	5	Gong	2012	P	7	Correction of maxillary deformity in infants with bilateral cleft lip and palate using computer-assisted design
Oral and Maxillofacial	5	Gopakumar	2004	T	1	RP in medicine: a case study in cranial reconstructive surgery
Oral and Maxillofacial	5	Hatamleh	2013a	T	1	Construction of an Implant-Retained Auricular Prosthesis with the Aid of Contemporary Digital Technologies: A Clinical Report
Oral and Maxillofacial	5	Hatamleh	2013b	T	4	Management of Extensive Frontal Cranioplasty Defects
Oral and Maxillofacial	5	He	2015	T	7	Novel Arch Bar Fabricated With a Computer-Aided Design and Three-Dimensional Printing: A Feasibility Study
Oral and Maxillofacial	5	Herlin	2011	S	1	Rapid prototyping in craniofacial surgery: Using a positioning guide after zygomatic osteotomy e A case report
Oral and Maxillofacial	5	Hierl	2013	S	3	CAD-CAM–Assisted Esthetic Facial Surgery
Oral and Maxillofacial	5	Holck	1999	P	2	Benefits of Stereolithography in Orbital Reconstruction
Oral and Maxillofacial	5	Hou	2011	P	7	Immediate Reconstruction of Bilateral Mandible Defects: Management Based on Computer-Aided Design/Computer-Aided Manufacturing Rapid Prototyping Technology in Combination With Vascularized Fibular Osteomyocutaneous Flap
Oral and Maxillofacial	5	Hu	2007	P	1	Reconstruction of a palatomaxillary defect with vascularized iliac bone combined with a superficial inferior epigastric artery flap and zygomatic implants as anchorage
Oral and Maxillofacial	5	Jang	2013	P	4	Autotransplantation of immature third molars using a computer-aided rapid prototyping model: a report of 4 cases
Oral and Maxillofacial	5	Juergens	2012	S	1	A Computer-Assisted Diagnostic and Treatment Concept to Increase Accuracy and Safety in the Extracranial Correction of Cranial Vault Asymmetries
Oral and Maxillofacial	5	Keightley	2010	S	1	Autotransplantation of an immature premolar, with the aid of cone beam CT and computeraided prototyping: a case report
Oral and Maxillofacial	5	Kfir	2013	P	1	The diagnosis and conservative treatment of a complex type 3 dens invaginatus using cone beam computed tomography (CBCT) and 3D plastic models
Oral and Maxillofacial	5	Kim	2007	S	1	Surgical positioning of orthodontic miniimplants with guides fabricated on models replicated with cone-beam computed tomography
Oral and Maxillofacial	5	Kim	2008	S	1	Clinical application of a stereolithographic surgical guide for simple positioning of orthodontic mini-implants
Oral and Maxillofacial	5	Kübler	1995	P	21	Repair of human skull defects using osteoinductive bone alloimplants
Oral and Maxillofacial	5	Kusek	2009	P	1	The use of laser technology (Er;Cr:YSGG) and stereolithography to aid in the placement of a subperiosteal implant: case study
Oral and Maxillofacial	5	Lee	2015	S	1	Keyhole System: A Computer-Assisted Designed and Computer-Assisted Manufactured Maxillomandibular Complex Repositioner in Orthognathic Surgery
Oral and Maxillofacial	5	Li	2009	P	1	Application of computer-aided three-dimensional skull model with rapid prototyping technique in repair of zygomatico-orbito-maxillary complex fracture
Oral and Maxillofacial	5	Li	2012	P	1	Preliminary Application of Virtual Simulation and Reposition Template for Zygomatico-orbitomaxillary Complex Fracture
Oral and Maxillofacial	5	Liu	2014c	S	1	Additive manufacturing and digital design assisted precise apicoectomy: a case study
Oral and Maxillofacial	5	Lucey	2010	P	1	Case report: Cone-beam CT imaging in the management of a double tooth
Oral and Maxillofacial	5	Matsuo	2010	T	2	Clinical application of a custom-made bioresorbable raw particulate hydroxyapatite/poly-L-lactide mesh tray for mandibular reconstruction
Oral and Maxillofacial	5	Mavili	2007	P	3	Use of Three-Dimensional Medical Modeling Methods for Precise Planning of Orthognathic Surgery
Oral and Maxillofacial	5	Mendez	2015	P	2	Customized ''In-Office'' Three-Dimensional Printing for Virtual Surgical Planning in Craniofacial Surgery
Oral and Maxillofacial	5	Oliveira	2010	P	1	Recessive dystrophic epidermolysis bullosa—oral rehabilitation using stereolithography and immediate endosseous implants
Oral and Maxillofacial	5	Olszewski	2010	P	1	Innovative procedure for computer-assisted genioplasty: three-dimensional cephalometry, rapid-prototyping model and surgical splint
Oral and Maxillofacial	5	Ozan	2012	S	1	Clinical Application of Stereolithographic Surgical Guide With a Handpiece Guidance Apparatus: A Case Report
Oral and Maxillofacial	5	Papaspyridakos	2015	S	1	Full Mouth Implant Rehabilitation with Staged Approach: 6-Year Clinical Follow-Up
Oral and Maxillofacial	5	Papaspyridakos	2010	S	1	Immediate Loading of the Maxilla with Prefabricated Interim Prosthesis Using Interactive Planning Software, and CAD/CAM Rehabilitation with Definitive Zirconia Prosthesis: 2-Year Clinical Follow-up
Oral and Maxillofacial	5	Papaspyridakos	2012b	S	1	Flapless CAD/CAM-guided surgery for staged transition from failing dentition to complete arch implant rehabilitation: a 3-year clinical report
Oral and Maxillofacial	5	Papaspyridakos	2008	S	1	Complete arch implant rehabilitation using subtractive rapid prototyping and porcelain fused to zirconia prosthesis: A clinical report
Oral and Maxillofacial	5	Park	2012	P	1	Autotransplantation with Simultaneous Sinus Floor Elevation
Oral and Maxillofacial	5	Park	2014	P	1	Optimizing Third Molar Autotransplantation: Applications of Reverse-Engineered Surgical Templates and Rapid Prototyping of Three-Dimensional Teeth
Oral and Maxillofacial	5	Rohner	2013	P	1	Importance of patient-specific intraoperative guides in complex maxillofacial reconstruction
Oral and Maxillofacial	5	Salmi	2013	T	1	A digital process for additive manufacturing of occlusal splints: a clinical pilot study
Oral and Maxillofacial	5	Sammartino	2004	S	1	Stereolithography in Oral Implantology A Comparison of Surgical Guides
Oral and Maxillofacial	5	Seitz	2016	T	1	A Combination of Various Technologies in the Fabrication of a Removable Partial Denture--A Case Study.
Oral and Maxillofacial	5	Shengwei	2014	T	7	Combined Use of an Anterolateral Thigh Flap and Rapid Prototype Modeling to Reconstruct Maxillary Oncologic Resections and Midface Defects
Oral and Maxillofacial	5	Shin	2015	S	1	A case report of a surgical guide fabricated via intraoral scanning-based implant planning and wax-based rapid prototyping
Oral and Maxillofacial	5	Singare	2007	P	2	Individually Prefabricated Prosthesis for Maxilla Reconstruction
Oral and Maxillofacial	5	Sultan	2011	S	3	Custom-Made, 3D, Intraoperative Surgical Guides for Nasal Reconstruction
Oral and Maxillofacial	5	Suzuki	2005	P	2	Application of a rapid-prototyped temporal bone model for surgical planning
Oral and Maxillofacial	5	Thomas	2013	S	4	Use of a titanium cutting guide to assist with raising and inset of a DCIA free flap
Oral and Maxillofacial	5	Toso	2005	P	16	Usefulness of Computed Tomography in pre-surgical evaluation of maxillo-facial pathology with rapid prototyping and surgical pre-planning by virtual reality
Oral and Maxillofacial	5	Turgut	2009	T	10	Use of Rapid Prototyping in Prosthetic Auricular Restoration
Oral and Maxillofacial	5	Verweij	2016	P	1	Autotransplantation of Premolars With a 3-Dimensional Printed Titanium Replica of the Donor Tooth Functioning as a Surgical Guide: Proof of Concept.
Oral and Maxillofacial	5	Watson	2014	T	1	Correction of Facial and Mandibular Asymmetry Using a Computer Aided Design/ Computer Aided Manufacturing Prefabricated Titanium Implant
Oral and Maxillofacial	5	Wiechmann	2008	T	1	Herbst appliance in lingual orthodontics
Oral and Maxillofacial	5	Wang	2013b	S	5	Application of Computer Techniques in Repair of Oblique Facial Clefts With Outer-Table Calvarial Bone Grafts
Oral and Maxillofacial	5	Wang	2015	P	1	Complex reconstruction of facial deformity and function after severe gunshot injury: one case report
Oral and Maxillofacial	5	Yoshioka	2010	T	1	Fabrication of an Orbital Prosthesis Using a Non-contact Three-Dimensional Digitizer and Rapid-Prototyping System
Oral and Maxillofacial	5	Zhen-yu	2010	P	1	Computer-assisted design and rapid prototyping technology for the underlay of hemifacial atrophy
Oral and Maxillofacial	5	Zhou	2010	T	6	Accurate Reconstruction of Discontinuous Mandible Using a Reverse Engineering/ Computer-Aided Design/Rapid Prototyping Technique: A Preliminary Clinical Study
Oral and Maxillofacial	5	Zhu	2011	P	15	Registration Strategy Using Occlusal Splint Based on Augmented Reality for Mandibular Angle Oblique Split Osteotomy
Musculoskeletal	5	Ai	2011	P	2	Transoral Atlantoaxial Reduction Plate Internal Fixation with Transoral Transpedicular or Articular Mass Screw of C2 for the Treatment of Irreducible Atlantoaxial Dislocation
Musculoskeletal	5	Bellanova	2013	S	4	Surgical Guides (Patient-Specific Instruments) for Pediatric Tibial Bone Sarcoma Resection and Allograft Reconstruction
Musculoskeletal	5	Brown	2002	S	8	Application of Computer-Generated Stereolithography and Interpositioning Template in Acetabular Fractures: A Report of Eight Cases
Musculoskeletal	5	Chen	2016b	T	1	Image-guided installation of 3D-printed patient-specific implant and its application in pelvic tumor resection and reconstruction surgery.
Musculoskeletal	5	D'Urso	1999d	P	5	Spinal biomodeling
Musculoskeletal	5	D'Urso	2005	P	20	Biomodeling as an Aid to Spinal Instrumentation
Musculoskeletal	5	Fan	2015	P	3	Implantation of customized 3-D printed titanium prosthesis in limb salvage surgery: a case series and review of the literature.
Musculoskeletal	5	Fang	2012	P	1	Guiding Templates For Pedicle Screws Insertion: A preliminary Study
Musculoskeletal	5	Hsu	2015	P	1	Patient-Specific 3-Dimensional Printed Titanium Truss Cage With Tibiotalocalcaneal Arthrodesis for Salvage of Persistent Distal Tibia Nonunion
Musculoskeletal	5	Hung	2008	P	1	Clinical Application of Rapid Prototype Model in Pediatric Proximal Femoral Corrective Osteotomy
Musculoskeletal	5	Li	2014	P	2	Composite artificial semi-knee joint system
Musculoskeletal	5	Lu	2009b	P	9	A novel computer-assisted drill guide template for placement of C2 laminar screws
Musculoskeletal	5	Minns	2003	P	1	The use of a reconstructed three-dimensional solid model from CT to aid the surgical management of a total knee arthroplasty: a case study
Musculoskeletal	5	Otsuki	2016	P	3	Utility of a custom screw insertion guide and a full-scale, color-coded 3D plaster model for guiding safe surgical exposure and screw insertion during spine revision surgery.
Musculoskeletal	5	Paiva	2007	P	1	Application of the stereolithography technique in complex spine surgery
Musculoskeletal	5	Papandrea	2014	P	1	New perspectives on treatment of both bone forearm malunions: three-dimensional (3D) modeling and printing
Musculoskeletal	5	Rahmati	2012	P	1	An improved methodology for design of custom-made hip prostheses to be fabricated using additive manufacturing technologies
Musculoskeletal	5	Singhal	2016	P	2	Improved Surgery Planning Using 3-D Printing: a Case Study.
Musculoskeletal	5	Tan	2015	P	4	A Novel Preoperative Planning Technique Using a Combination of CT Angiography and Three-Dimensional Printing for Complex Toe-to-Hand Reconstruction.
Musculoskeletal	5	Toyoda	2013	P	1	Novel Approach for the Effi cient Use of a Full-Scale, 3-Dimensional Model for Cervical Posterior Fixation
Musculoskeletal	5	Tricot	2012	P	3	3D-corrective osteotomy using surgical guides for posttraumatic distal humeral deformity
Musculoskeletal	5	Truscott	2007	P	1	Using RP to promote collaborative design of customised medical implants
Musculoskeletal	5	Yamazaki	2006	P	1	Surgical Simulation of Instrumented Posterior Occipitocervical Fusion in a Child With Congenital Skeletal Anomaly
External Devices	5	Buehler	2016	T	1	Investigating the Implications of 3D Printing in Special Education
External Devices	5	Eggbeer	2011	T	1	Computer-aided methods in bespoke breast prosthesis design and fabrication
External Devices	5	Faustini	2005	T	1	Design and Analysis of Orthogonally Compliant Features for Local Contact Pressure Relief in Transtibial Prostheses
External Devices	5	Hsu	2010	T	1	The development of a rapid prototyping prosthetic socket coated with a resin layer for transtibial amputees
External Devices	5	Mavroidis	2011	T	1	Patient specific ankle-foot orthoses using rapid prototyping
External Devices	5	Qiao	2016	T	3	A novel combination of computer-assisted reduction technique and three dimensional printed patient-specific external fixator for treatment of tibial fractures
External Devices	5	Rogers	2008	T	1	Case Report: Variably Compliant Transtibial Prosthetic Socket Fabricated Using Solid Freeform Fabrication
External Devices	5	Sengeh	2013	T	1	A Variable-Impedance Prosthetic Socket for a Transtibial Amputee Designed from Magnetic Resonance Imaging Data
External Devices	5	South	2010	T	1	Manufacture of Energy Storage and Return Prosthetic Feet Using Selective Laser Sintering
External Devices	5	Tay	2002	T	1	A CASD-CASM method for prosthetic socket fabrication using the FDM technology
External Devices	5	Yoshikawa	2015	T	1	Rehand: Realistic Electric Prosthetic Hand Created with a 3D Printer

PRISMA 2009 Checklist

Section/topic	#	Checklist item	Reported on page #
**TITLE**			
Title	1	Identify the report as a systematic review, meta-analysis, or both.	1
**ABSTRACT**			
Structured summary	2	Provide a structured summary including, as applicable: background; objectives; data sources; study eligibility criteria, participants, and interventions; study appraisal and synthesis methods; results; limitations; conclusions and implications of key findings; systematic review registration number.	1
**INTRODUCTION**			
Rationale	3	Describe the rationale for the review in the context of what is already known.	2
Objectives	4	Provide an explicit statement of questions being addressed with reference to participants, interventions, comparisons, outcomes, and study design (PICOS).	2
**METHODS**			
Protocol and registration	5	Indicate if a review protocol exists, if and where it can be accessed (e.g., Web address), and, if available, provide registration information including registration number.	2
Eligibility criteria	6	Specify study characteristics (e.g., PICOS, length of follow-up) and report characteristics (e.g., years considered, language, publication status) used as criteria for eligibility, giving rationale.	3
Information sources	7	Describe all information sources (e.g., databases with dates of coverage, contact with study authors to identify additional studies) in the search and date last searched.	2
Search	8	Present full electronic search strategy for at least one database, including any limits used, such that it could be repeated.	Appendix1
Study selection	9	State the process for selecting studies (i.e., screening, eligibility, included in systematic review, and, if applicable, included in the meta-analysis).	3
Data collection process	10	Describe method of data extraction from reports (e.g., piloted forms, independently, in duplicate) and any processes for obtaining and confirming data from investigators.	3
Data items	11	List and define all variables for which data were sought (e.g., PICOS, funding sources) and any assumptions and simplifications made.	2
Risk of bias in individual studies	12	Describe methods used for assessing risk of bias of individual studies (including specification of whether this was done at the study or outcome level), and how this information is to be used in any data synthesis.	3
Summary measures	13	State the principal summary measures (e.g., risk ratio, difference in means).	3
Synthesis of results	14	Describe the methods of handling data and combining results of studies, if done, including measures of consistency (e.g., I^2) for each meta-analysis.	N/A

For peer review only - http://bmjopen.bmj.com/site/about/guidelines.xhtml

PRISMA 2009 Checklist

Section/topic	#	Checklist item	Reported on page #
Risk of bias across studies	15	Specify any assessment of risk of bias that may affect the cumulative evidence (e.g., publication bias, selective reporting within studies).	Table 3
Additional analyses	16	Describe methods of additional analyses (e.g., sensitivity or subgroup analyses, meta-regression), if done, indicating which were pre-specified.	N/A
**RESULTS**			
Study selection	17	Give numbers of studies screened, assessed for eligibility, and included in the review, with reasons for exclusions at each stage, ideally with a flow diagram.	Figure 1
Study characteristics	18	For each study, present characteristics for which data were extracted (e.g., study size, PICOS, follow-up period) and provide the citations.	Table 2
Risk of bias within studies	19	Present data on risk of bias of each study and, if available, any outcome level assessment (see item 12).	Table 3
Results of individual studies	20	For all outcomes considered (benefits or harms), present, for each study: (a) simple summary data for each intervention group (b) effect estimates and confidence intervals, ideally with a forest plot.	Table 2
Synthesis of results	21	Present results of each meta-analysis done, including confidence intervals and measures of consistency.	N/A
Risk of bias across studies	22	Present results of any assessment of risk of bias across studies (see Item 15).	N/A
Additional analysis	23	Give results of additional analyses, if done (e.g., sensitivity or subgroup analyses, meta-regression [see Item 16]).	N/A
**DISCUSSION**			
Summary of evidence	24	Summarize the main findings including the strength of evidence for each main outcome; consider their relevance to key groups (e.g., healthcare providers, users, and policy makers).	4
Limitations	25	Discuss limitations at study and outcome level (e.g., risk of bias), and at review-level (e.g., incomplete retrieval of identified research, reporting bias).	5
Conclusions	26	Provide a general interpretation of the results in the context of other evidence, and implications for future research.	5
**FUNDING**			
Funding	27	Describe sources of funding for the systematic review and other support (e.g., supply of data); role of funders for the systematic review.	5

From: Moher D, Liberati A, Tetzlaff J, Altman DG, The PRISMA Group (2009). Preferred Reporting Items for Systematic Reviews and Meta-Analyses: The PRISMA Statement. PLoS Med 6(6): e1000097. doi:10.1371/journal.pmed1000097

For more information, visit: www.prisma-statement.org.

For peer review only - http://bmjopen.bmj.com/site/about/guidelines.xhtml

BMJ Open

Clinical efficacy and effectiveness of 3D printing: A systematic review

Journal:	*BMJ Open*
Manuscript ID	bmjopen-2017-016891.R1
Article Type:	Research
Date Submitted by the Author:	23-Aug-2017
Complete List of Authors:	Diment, Laura; University of Oxford, Department of Engineering, Institute of Biomedical Engineering<br>Thompson, Mark; University of Oxford, Department of Engineering, Institute of Biomedical Engineering<br>Bergmann, Jeroen; University of Oxford, Department of Engineering, Institute of Biomedical Engineering
**Primary Subject Heading**:	Patient-centred medicine
Secondary Subject Heading:	Evidence based practice
Keywords:	Printing, Three-Dimensional, Health Care Evaluation Mechanisms, Medical devices, Personalised healthcare, Fabrication, Additive manufacturing

SCHOLARONE™
Manuscripts

Title page

Clinical efficacy and effectiveness of 3D printing: A systematic review

Corresponding Author:

Laura E. Diment
DPhil student
Department of Engineering Science
University of Oxford
Parks Road, Oxford, UK
OX1 3PJ
laura.diment@eng.ox.ac.uk
Ph. 01865 227374

Co-authors:

Mark S. Thompson
Director, Oxford Mechanobiology Group
Department of Engineering Science
University of Oxford
Parks Road, Oxford, UK
OX1 3PJ

Jeroen HM. Bergmann
Associate Director, Oxford Biodesign Programme
Department of Engineering Science
University of Oxford
Parks Road, Oxford, UK
OX1 3PJ

Word count: 3815

For peer review only

ABSTRACT

Objective

To evaluate the clinical efficacy and effectiveness of using 3D printing to develop medical devices across all medical fields.

Design

PRISMA-compliant systematic review

Data sources

PubMed, Web of Science, OVID, IEEE Xplore and Google Scholar.

Methods

A double-blinded review method was used to select all abstracts that reported on clinical trials of a 3D printed medical device. The studies were ranked according to their level of evidence, divided into medical fields based on the International Classification of Diseases chapter divisions, and categorised into whether they were used for preoperative planning, aiding surgery or therapy. The Downs and Black Quality Index critical appraisal tool was used to assess the quality of reporting, external validity, risk of bias, risk of confounding and power of each study.

Results

Of the 3084 abstracts screened, 350 studies met the inclusion criteria. Oral and Maxillofacial Surgery contained 58.3% of studies, and 23.7% covered the Musculoskeletal System. Only 21 studies were randomised controlled trials (RCTs), and all fitted within these two fields. The majority of RCTs were 3D printed anatomical models for preoperative planning and guides for aiding surgery. The main benefits of these devices were decreased surgical operation times and increased surgical accuracy.

Conclusions

All medical fields that assessed 3D printed devices concluded that they were clinically effective. The fields that most rigorously assessed 3D printed devices were Oral and Maxillofacial Surgery and the Musculoskeletal System, both of which concluded that the 3D printed devices out-performed their conventional comparators. However, the efficacy and effectiveness of 3D printed devices remains undetermined for the majority of medical fields. 3D printed devices can play an important role in healthcare, but more rigorous and long-term assessments are needed to determine if 3D printed devices are clinically relevant before these devices become part of standard clinical practice.

Strengths and limitations of the study

- This review provides a broad overview of how 3D printing is being used across all medical fields.
- The review systematically compares the way efficacy and effectiveness have been tested for 3D printed medical devices in different medical fields, showing where gaps in research are.
- The study is limited to a critical appraisal of individual studies, rather than a meta-analysis, because of the breadth of uses (from anatomical models and surgical guides to therapeutic devices) and the lack of comparable hypotheses.
- The study is limited to devices that have been used in clinical trials. There is substantial research going into developing new 3D printed technologies such as biological printing but these devices have yet to be trialled in humans.

For peer review only - http://bmjopen.bmj.com/site/about/guidelines.xhtml

INTRODUCTION

3D printing is likely to play a pivotal role in transforming healthcare and clinical practice because it provides the opportunity to create customised devices designed for the complexity and individual variances of the patient populations.[1]

Additive manufacturing, more commonly referred to as 3D printing, is an industrial production technique that enables a 3D digital model to be converted into a physical model by printing it layer by layer. For decades 3D printing has been used for rapid prototyping, but the recent advances in the available materials, speed, resolution, accuracy, reliability, cost and repeatability of 3D printing technologies have broadened the possibilities for clinical uses.[2,3] Many medical fields are already using 3D printing to manufacture custom surgical tools, guides, dose delivery devices, implants, external prosthetics or orthotics, and devices for preoperative planning or education.[2,4,5] Tailoring devices and procedures to the patient is expected to reduce the time required for surgery, treatment or recovery, while increasing the accuracy and success of the outcome.[1] The worldwide 3D printing industry's revenue from products and services is over $4 billion and fast-growing, with 13.1% of the industry attributed to the medical sector.[6]

In most medical fields, 3D printing applications are still in the research and development stage or have only just entered clinical practice within the last decade, and hence there has been a lack of research into the clinical efficacy, effectiveness and long-term follow-up in comparison to traditional technologies.[7] *Efficacy* refers to the performance of the device under ideal and controlled conditions, and *effectiveness* refers to its performance under typical clinical conditions.[8] The Food and Drug Administration (FDA) states that 3D printed medical devices are required to meet the same regulations as their non-3D printed counterparts. Because 3D printed devices can have different safety and efficacy issues than the equivalent devices, additional testing may be required to demonstrate safety, efficacy and effectiveness.[9] Hospitals and clinics will need to adopt new medical product procedures when they want to introduce 3D printing for healthcare, and more evidence of device efficacy and effectiveness will help make an informed discussion before prescribing 3D printed devices for patients. Providing a critical appraisal of the efficacy and effectiveness of 3D printed medical devices gives healthcare professionals a resource to assess the validity of the devices, and provides researchers with an overview of areas that require further research and validation.

Previous systematic reviews summarise 3D printed medical devices being used in specific medical fields, such as plastic and reconstructive surgery,[10] preoperative planning for liver resections,[11] and increasingly available 3D printing processes used in dentistry and mandibular reconstructions.[12–14] Systematic reviews on the advantages of 3D printed devices over conventional methods in surgery have found improved clinical outcomes, and reductions in operating times and manufacturing costs.[5,15] However, the reviews do not critically appraise the studies, and are therefore subject to bias. The only systematic review that did assess bias for a very specific field found no difference in patient outcomes when 3D printed instrumentation for total knee replacements was compared to conventional instrumentation.[16] This highlights the need for a systematic review that incorporates a critical appraisal of the studies that are included.

This review aims to assess the clinical efficacy and effectiveness of 3D printed devices through performing a systematic literature search, categorising reports of 3D printed device usage by medical field and purpose, and assessing the reports' scientific quality. The key findings of studies that compare 3D printed medical devices to their non-3D printed counterparts are presented, and the gaps in research highlighted.

METHODS

Search strategy

The applied protocol (not previously registered) used systematic methods to search for relevant studies, screen them for eligibility, and assess their quality. The review follows the Preferred Reporting Items for Systematic Reviews and Meta-Analyses (PRISMA) guidelines.[17]

A literature search was performed using PubMed, Web of Science, OVID, IEEE Xplore and Google Scholar. A combination of 45 relevant keywords were used to collect all studies that included a 3D printed device and a clinical trial. Due to the word and character limit search restrictions in IEEE Xplore and Google Scholar, a narrower search of 15 terms was performed for these databases. The search includes all publications up to January 2017. The complete search strategy is provided in the supplementary material. Mendeley was used as a reference manager.

For peer review only - http://bmjopen.bmj.com/site/about/guidelines.xhtml

Study selection

After removing all duplicates from the databases, the title and abstract of each publication was reviewed using a double-blinded method (undertaken by LD and JB) to determine eligibility for inclusion.

The inclusion criteria were:

i. **Relevance -** papers were required to report first-hand on the results of a clinical study that assessed the efficacy and effectiveness of a 3D printed device.
ii. **Language -** only papers written or translated into English were included in the review.
iii. **Peer-review -** records that had no peer-review or where the level of peer-review could not be traced were excluded from the review.

Where the results of the reviewers (LD and JB) conflicted, the reviewers discussed their reasoning for inclusion/exclusion, and where required for clarification of the paper's relevance, the paper's introduction and methods were assessed. Consensus on eligibility was achieved for all papers. Included articles underwent a full-text review and references cited in these studies were also examined for relevance using the same inclusion criteria.

Quality assessment

The studies were then rated according to their level of evidence, based on the Oxford Centre for Evidence-based Medicine (CEBM) Levels of Evidence,[18] as summarised in Table 1. Systematic reviews and opinion papers were excluded because they cannot provide the original data in a suitable format to be assessed using the quality assessment method used in this review. Where studies used 3D printed devices but the hypothesis, aim or objective was focused on an outcome other than the effect of the 3D printed device, the paper has been graded as the level of evidence shown for the device. All levels of evidence were included in the review but only those that used a control group (Levels 1 to 3) were critically analysed.

Table 1. CEBM Levels of Evidence

Level	Types of study
1	- Randomised controlled trials (RCTs)
2	- Cohort studies
3	- Case-control studies<br>- Poor quality estimates of data that include sensitivity analyses incorporating clinically sensible variations
4	- Case-series studies
5	- Case reports<br>- Studies that do not provide quantitative evidence to support a hypothesis

Studies were then placed in medical fields based on the International Classification of Diseases (ICD) chapter divisions[19] and categorised by the purpose of the device. The three categories were; devices used for preoperative planning, devices to aid surgery, and therapeutic devices.

To analyse the quality of the studies under review, critical appraisal tools were assessed to find one that met the following criteria:

- Suitable for assessing the quality of both randomised and non-randomised studies,
- Well-regarded and commonly used for quality assessments in systematic reviews.
- Demonstrates internal consistency, test-retest reliability and criterion validity.
- Assesses the risk of bias, the participant selection methods, the study protocol, and the validity, reliability and responsiveness of the study's results.
- Simple and intuitive to interpret.
- Does not assign weights to the items in the scale where there is a lack of empirical evidence to support the assignment.[20]

For peer review only - http://bmjopen.bmj.com/site/about/guidelines.xhtml

The Downs and Black Quality Index[21] meets all but the final criterion on this list. To overcome this limitation, responses have been left in their raw form (yes/no/unable to determine/not applicable, in accordance with the definitions provided by Downs and Black[21]) and presented in a table to enable the reader to visualise the category trends, as recommended by the Cochrane Handbook for Systematic Reviews of Interventions.[20]

The study details and data relating to the quality of study design and reporting were manually extracted from each paper by one reviewer (LD). The study details included medical field, study design, device category, device purpose, number of participants, age and gender of participants, and aims and outcomes of the study. The data relating to study design and reporting were extracted according to the criteria used by the Downs and Black Quality Index, divided into reporting, external validity, bias, confounding and power. Where there were uncertainties as to how the study fitted with the Downs and Black Quality Index criteria, the other two authors were consulted and in all cases, an agreement was reached.

RESULTS

The search yielded 4505 records and, after removing duplicates, 3084 abstracts were screened. A total of 350 studies met the inclusion criteria and were included in the review (Figure 1).

Due to the large number of included studies, this paper gives an overview of the level of evidence found for 3D printed medical devices in each medical field and then focuses on the outcomes of the Level 1 studies. Information for all studies included in the review and a critical appraisal of all comparative studies is provided in the supplementary material.

A wide range of 3D printed devices have been developed and clinically trialled. The most common device types were anatomical models for preplanning surgeries (40.9%), followed by guides to aid in surgery (37.1%). The studies covered 9 medical fields, with the majority (58.3%) of studies falling into the Oral and Maxillofacial Surgery field, which included dentistry and orthopaedic surgery of the jaw, face and skull, and those covering the Musculoskeletal System (23.7%) making up the second group. Some studies spanned multiple medical fields, such as neoplasms that occur in the circulatory system. In these cases, the authors selected the most prominent field of the paper. Only 14.0% of included studies used a control group (Levels 1 to 3 in Figure 2), whereas 41.4% were level 5 studies. The average number of participants per controlled trial was 41 (36 for RCTs), compared to 17 for all studies.

All 21 Level 1 studies fell within the Oral and Maxillofacial Surgery or Musculoskeletal System categories, with one cross-field study fitting in both Oral and Maxillofacial Surgery and Neoplasms (Table 2 summarises all RCTs). All Level 1 studies, with the exception of Stephens et al.,[22] used objective measures as their indicators of device efficacy and effectiveness. Of these, the 7 models used for preoperative planning were all modelling bone or fracture fixation plates, though the locations spanned skull, face, mandible, arm, spine, hip and ankle. All aimed to assess the effectiveness of using the models for surgical planning in treating specific conditions. Six out of 7 studies used operating time, 4 used accuracy of the surgery and 3 used blood loss as key indicators of device effectiveness. Aesthetics, recovery time and post-surgery function were also used as indicators of device effectiveness. All Level 1 studies of 3D printed models for preoperative planning that used time, accuracy or blood loss as indicators of device effectiveness reported that the test group had better outcomes than the control group, with statistical significance at $p<0.05$ found for 4 studies that assessed operating time, 3 studies that assessed accuracy, and 2 studies that assessed blood loss. The two studies that assessed recovery time found no difference between groups, one of the two studies that assessed user function found statistically significant differences in the test group, and the other found no difference between groups.

Of the 11 RCTs that assessed 3D printed guides or templates that supported navigation and surgery (of the mandible, teeth, shoulder, spine, hip or knee), 9 studies used accuracy of positioning as an indicator of device effectiveness, all of which found an improvement when using the guide or template, and 8 of which showed statistically significant results at $p<0.05$. All 4 studies that used time as an indicator of device effectiveness showed decreased operating times when using the guide or template (all with $p<0.05$), and 5 of the 6 studies that used changes at the operation site (blood loss, inflammatory response, tactile sensation or aesthetics) found statistically significant improvements at $p<0.05$.

Only 3 therapeutic devices were tested using RCTs. These were zirconia crowns, which were found to have a better marginal gap and internal fit than the conventional crowns tested ($p<0.05$), cranial implants, which showed less vertical ridge resorption over time ($p<0.05$), and osteosynthesis plates for intercondylar humeral

For peer review only - http://bmjopen.bmj.com/site/about/guidelines.xhtml

fractures, which showed a reduction in operating time ($p<0.05$) but no difference in elbow function during recovery.

While most Level 1 studies found statistically significant improvements at $p<0.05$ when using the 3D printed device, none performed an analysis on the clinical relevance of these improvements.

For peer review only

For peer review only - http://bmjopen.bmj.com/site/about/guidelines.xhtml

Table 2. Summary of RCTs (Type: P = preoperative planning, S = surgical tool or guide, T = therapeutic device)

Sector	First author, date	Device	Type	Size (n)	Participant details	Aim	Outcomes
Neoplasms	de Farias, 2014[23]	Mandible model	P	37 (Test=17, Control=20)	Age: 9 to 74 years (Test mean = 39.57 years, Control mean = 53.11 years). Test = 14M/3F, Control = 15M/5F	Aim: to evaluate the efficacy of using 3D printed models to plan head and neck surgeries.	Reconstruction time decreased; Test = 43.7 mins, Control = 127.7 mins, p = 0.001. Though statistical significance was not demonstrated, the size of the bone flap taken for reconstruction was typically smaller, and the aesthetic results better in the test group.
Oral and Maxillofacial	Ahrberg, 2015[24]	All-ceramic zirconia crowns	T	25 (Same participants used for test and control)	Age: >18 years 10M/15F	Aim: to evaluate the marginal and internal fit of 3D printedzirconia crowns and three-unit fixed dental prostheses resulting from direct versus indirect digitalization.	The marginal gap was smaller; Test = 61.08 ± 24.77 µm, Control = 70.40 ± 28.87 µm, p<0.05, and the fit was better at the centro-occlusal site; Test = 155.57 ± 49.85, Control = 171.51 ± 60.98, p<0.05. The fit improved at the mid-axial wall and axio-occlusal transition but the results lacked statistical significance.
Oral and Maxillofacial	D'Urso, 1999[25]	Cranio-maxillofacial model	P	30 (Test=15, Control=15)	No information provided	Aim: to assess whether biomodels in addition to standard imaging have greater utility in patient education, diagnosis and operative planning than the standard imaging alone.	3D printed models improved operative planning accuracy; Test = 82.21%, Control = 44.09%, p<0.01 and diagnosis accuracy; Test = 95.23%, Control = 65.63%, p<0.01. Measurement accuracy improved; Test error = 7.91%, Control error = 44.14%, p<0.05). Surgeons estimated that operating time over 45 operations was also reduced by 17.63%.
Oral and Maxillofacial	Al-Ahmad, 2013[26]	Surgical guide for sagittal splitting ramus osteotomy	S	8 (One side used as test, the other as control)	Age: 18-30 years (mean=23 years)	Aim: to evaluate the effectiveness of sagittal splitting ramus osteotomy in reducing the incidence and severity of neurosensory alterations, using a 3D printed surgical guide.	The Semmes–Weinstein monofilament tactile threshold was considered abnormal at >2.83 on the χ^2 test. At 1 week after surgery; Test = 67% abnormal, Control = 83% abnormal. This difference showed statistical significance (p<0.05) at 3 months post-surgery for chin and 6 months post-surgery for lower lip. For the two-point discrimination, the lower lip showed statistical significance at 1 week (Test = 9 mm, Control = 22 mm), and the chin at 6 months (Test = 11 mm, Control = 30 mm).
Oral and Maxillofacial	Ayoub, 2014[27]	Guide for mandibular reconstruction	S	20 (Test=10, Control=10)	Age: Test=52.3 ± 19.2 years, Control=54.7 ± 14.2 years). Test=4M/6F, Control=6M/4F	Aim: to evaluate the benefits of computer-assisted mandibular reconstruction with iliac crest bone grafts regarding the intraoperative time for transplant shaping, ischemia, duration of surgery, amount of bone removed and the change in postoperative condyle position compared to conventional surgery.	Computer-assisted surgery shortened the time of transplant ischemia; Test = 96.1 ± 15.8 mins, Control = 122.9 ± 20.4 mins, p<0.005, and defect reconstruction; Test = 6.2 ± 4.9 mins, Control = 20.3 ± 7.4 mins, p<0.001. There was less bone harvested; Test = no difference regarding defect size, Control = 16.8 ± 5.6 mm larger transplant size than defect size, p<0.001. The intercondylar distance before compared to after surgery was less affected; Test = 1.3 ± 0.2 mm, Control = 5.5 ± 2.5 mm, p<0.001.
Oral and Maxillofacial	Goh, 2015[28]	Polycaprolactone scaffold for ridge preservation	T	13 (Test=6, Control=7)	Age: 29–60 years (mean=46.8 years). 7M/7F (1 lost to follow-up, so excluded from results)	Aim: to evaluate the feasibility and effectiveness of using a polycaprolactone scaffold in fresh extraction sockets for ridge preservation.	The test group showed less vertical ridge resorption in all aspects (mesio-buccal, mid-buccal and disto-buccal) with statistically significant results in the mesio-buccal aspect; Test = 0.13 ± 0.96 mm, Control = -2.18 ± 1.02mm, p = 0.008), with better maintenance of ridge height after 6 months.
Oral and Maxillofacial	Van de Velde, 2010[29]	Maxilla surgical guide	S	13 (Test=36 tooth implants, Control=34 tooth implants)	Age: 39-75 years (mean =55.7 years), 4M/9F	Aim: to compare the outcome of dental implants placed using a flapless protocol with a 3D printed guide and immediate loading with a conventional protocol and loading.	The height of the attached mucosa at 1 week post-surgery was less; Test = 3.26 ± 1.57 mm, Control = 6.01 ± 1.10 mm, p<0.05. Marginal bone levels were not statistically significantly different between test and control implants; Test = 1.95 mm ± 0.70 and 1.93mm ± 0.42 after 18 months. Opinion about speech, function, aesthetics and self-confidence was statistically significantly better for the test side after 1 week when using a VAS rating scale, p<0.05.
Oral and Maxillofacial	Vercruyssen, 2014[30]	Surgical guides for edentulous jaws	S	59 (72 jaws, 12 in each group)	Age:31-78 years 31 male jaws, 41 female jaws	Aim: To assess the accuracy of guided surgery compared to mental navigation or the use of a surgical template, in fully edentulous jaws.	There was lower deviation at the entry point (1.4mm), at the apex (1.6 mm) and angular deviation (3.0°) when using the 3D printed guiding systems (all p<0.05). There were negligible differences between bone and mucosa support or type of guidance.
Musculoskeletal	Chareancholvanich, 2013[31]	Cutting guides for total knee replacement	S	80 (Test=40, Control=40)	Age: 55-84 years (mean = 69.5 years) 6M/34F	Aim: to compare patient-specific cutting guides with conventional instrumentation in total knee replacement.	The tibial component in the test group was marginally closer to neutral alignment; Test = 89.8 ± 1.2°, Control = 90.5 ± 1.6°, p=0.03. The 3D printed guides also shortened the bone-cutting time by 3.6 mins (p<0.001) and the operating time by 5.1 mins (p=0.019), without differences in post-operative blood loss (p=0.528) or need for blood transfusion (p=0.789). These minimal advantages of the 3D printed guides are unlikely to be clinically relevant.
Musculoskeletal	Chen, 2015[32]	Guide plate for pedicle screw fixation	S	43 (Test=20, Control=23)	Age: Test=35-70 years (mean=52.3 years) Control=37-72 years (mean=55.4 years) Test=9M/11F, Control=12M/11F	Aim: to evaluate the clinical efficacy of use of a 3D printing guide plate in posterior lumbar pedicle screw fixation.	Placement time for each screw was shorter (Test = 4.9 ± 2.1 mins, Control = 6.5 ± 2.2 mins), the amount of hemorrhage was less (Test = 8.0 ± 11.1 mL, Control = 59.9 ± 13.0 mL), and the fluoroscopy times of each screw placement was lower (Test = 0.5 ± 0.4 mins, Control = 1.2 ± 0.7 mins), p<0.05 for all tests. The excellent and good screw placement rate was 100% in the test group and 98.4% in the control group, with no statistical difference. No complications were reported.

For peer review only - http://bmjopen.bmj.com/site/about/guidelines.xhtml

Musculoskeletal	Du, 2013[33]	Templates to aid in pin placement in hip resurfacing arthroplasty	S	34 (Test-16, Control=18)	Ages: 37-55 years	Aim: To assess whether 3D printed patient-specific templates aid in accurate intraoperative pin placement.	The prosthesis stem shaft angle was greater; Test = 138.68 ± 8.85°, Control = 118.9 ± 12.8, p=0.001. The locating template can provide precise and dependable location for hip resurfacing femoral components during arthroplasty and ensure the valgus stem placement necessary for optimal outcomes.
Musculoskeletal	Gan, 2015[34]	Navigational template for total knee arthroplasty	S	70 (Test=35, Control=35)	Age: Test=63-75 years (mean=68 years), Control=64-72 years (mean=67.8 years) Test=10M/25F, Control=9M/26F	Aim: to validates a novel patient-specific navigational template for total knee arthroplasty.	The 3D printed navigational template reduced operation time (Test = 45 ± 8 mins, Control = 60 ± 10 mins, p<0.001) and blood loss (Test = 200 ± 45 mL, Control = 290±60 mL, p<0.001). The mean deviation from the neutral axis also decreased for the frontal femoral component (Test = 1.0±0.8°, Control = 2.6±1.8°, p<0.001) and the frontal tibial component (Test = 1.2±1.0°, Control = 2.8±1.5°, p<0.001).
Musculoskeletal	Hendel, 2012[35]	Guide for determining glenoid component position	S	31 (Test=15, Control=16)	No information provided	Aim: to compare patient-specific instruments with standard surgical instruments in determining glenoid component position.	The 3D printing technology decreased the average deviation of implant position for inclination; Test = 2.9 ± 3.4°, Control = 11.6 ± 7.0°, p<0.0001, and medial-lateral offset; Test = 1.0±0.9mm, Control = 1.9±1.0mm, p<0.05. The greatest benefit was observed in patients with retroversion >16˚; Test = 1.2±2.0˚, Control = 10±4.4˚, p<0.001.
Musculoskeletal	Maini, 2016[36]	Plate for acetabulum fracture fixation	P	21 (Test=10, Control=11)	Age: 18-60 years (mean=38.7 years) Test=9M/1F, Control=9M/2F	Aim: to evaluate the accuracy of patient-specific pre-contoured plates.	Greater reduction was achieved, as evaluated by CT scan; Test = 8.36 ± 5.82 mm, Control = 2.99 ± 1.34mm, p<0.05. Reduced blood loss; Test = 620 ± 247 mL, Control = 720 ± 286 mL, and surgical time; Test = 120 ± 38 mins, Control = 132 ± 41 mins, were observed but the results were not statistically significant (p>0.05). All plates fitted well to the pelvis intra-operatively.
Musculoskeletal	Merc, 2013[37]	drill guide for screw placement	S	19 (Test=9 (54 screws), Control=10(54 screws))	Age: Test=59 ± 5 years, Control=62 ±12 years Test=4M/5F, Control=5M/5F	Aim: to evaluate the accuracy of a multi-level drill guide template for lumbar and first sacral pedicle screw placement and to compare it with the freehand technique under fluoroscopy supervision.	The incidence of cortex perforation was reduced; Test = 0, Control = 8, p<0.05. So was the deviation of pedicle screw position in the sagittal plane; Test = 2 ± 10˚, Control = -12 ± 8˚, p<0.05, but there was no discernable difference in the transverse plane deviation or displacement in sagittal or transverse planes.
Musculoskeletal	Shuang, 2016[38]	Osteosynthesis Plates	T	13 (Test=6, Control=7)	Age: Test=31-62 years (mean=46.2 years) Control=27-59 years (mean=40.3 years) Test= 4M/2F, Control=6M/1F	Aim: to evaluate the efficacy custom 3D printed osteosynthesis plates in the treatment of intercondylar humeral fractures.	Operative time was shorter; Test = 70.6 ± 12.1 mins, Control = 92.3 ± 17.4 mins, p<0.05. The mean time to bone union was 3.4 months. No difference was found in the rate of patients with good elbow function, or in the ranges of elbow flexion/extension and pronation/supination (all p>0.05).
Musculoskeletal	Stephens, 2016[22]	Bone models	P	58 (Test=29, Control=29)	Age: >60 years (mean=73 years) 5M/53F	Aim: to investigate the efficacy of 3D printed bone models as a tool to facilitate initiation of bisphosphonate treatment among individuals who are newly diagnosed with osteoporosis.	Using the 9-item Brief Illness Perception Questionnaire with a 0-10 rating scale, the test group were more emotionally affected by osteoporosis immediately after the interview (Test = 4.08±0.41, Control = 2.89 ± 0.40, p>0.05) and reported a greater understanding of osteoporosis at the 2 month follow-up (Test = 7.19 ± 0.51, Control = 5.72 ± 0.49, p<0.04).
Musculoskeletal	Wu, 2011[39]	Spine model	P	62 (Test=34, Control=28) 677 screws inserted	Age: 4-22 years (mean=11 years)	Aim: to compare the accuracy and safety of pedicle screw placement in congenital scoliosis using the 3D printing technique versus conventional fluoroscopy.	Results for thoracolumbar and lumbar, respectively: Shorter operation time; Test = 6.5 and 5.1 hours, Control = 8.5 and 6.3 hours, p<0.05. Higher scoliosis correction ratio; Test = 83.8 and 81.8, Control = 76.6 and 64.7, p<0.05. Accuracy of screw placement; Test = 94.4% and 91.6%, Control = 86.1% and 82.0%, p<0.05.
Musculoskeletal	Yang, 2016[40]	Model of trimalleolar fracture	P	30 (Test=15, Control=15)	Age: 31-42 years (mean=36.5 years) 16M/14F	Aim: to evaluate the effectiveness of using 3D printed models for surgical planning in treating trimalleolar fractures and in physician-patient communication.	Shorter operation time; Test = 184.32 ± 4.65 mins, Control = 212.32 ± 8.17 mins, p<0.001. Less blood loss; Test = 846.68 ± 26.11 mL, Control = 1029.65 ± 72.18 mL, p<0.001. No statistically significant differences were observed in complication rate, length of hospital stay, and postoperative radiological outcomes (all, p>0.05).
Musculoskeletal	You, 2016[41]	Proximal humeral fracture model	P	66 (Test=34, Control=32)	61-76 years (Test = 66.09±4.09 years, Control = 66.28± 4.10 years) 27M/39F	Aim: to investigate the feasibility and clinical potential of using 3D printed models versus the conventional thin-layer CT scan for the treatment of proximal humeral fractures in old people.	Surgery time decreased; Test = 77.65 ± 8.09 mins, Control = 92.03 ± 10.31 mins, p<0.05, there was less blood loss; Test = 235.29 ± 63.40 mL, Control = 281.25 ± 57.85 mL, p<0.05, and a lower number of fluoroscopy; Test = 7.12 ± 1.57, Control = 10.59 ± 1.36, p<0.05. The results showed no statistically significant difference in time to union (p>0.05).
Musculoskeletal	Zhang, 2011[42]	Acetabular navigational template	S	22 (Test=11, Control=11)	Age: Test=48.6 ± 6.8 years, Control=49.3 ± 4.9 years Test=7M/4F, Control=5M/6F	Aim: to compare a customised 3D printed acetabular navigational template for total hip arthroplasty (THA) to conventional THA in adult single development dysplasia of the hip.	The templates facilitated accurate placement of acetabular components in dysplasia of acetabulum. At 1 year follow-up there were smaller differences from the predetermined angles of 45° abduction and 18° anteversion; Test = 1.6 ± 0.4°, 1.9 ± 1.1°, Control = 5.8 ± 2.9°, 3.9 ± 2.5°, p<0.05.

Critical appraisal of the RCTs from each medical field are shown in Table 3. The table includes the abridged quality index questions. Studies with higher levels of evidence typically rated better across all areas assessed using the quality index. However, many of the RCTs did not demonstrate external validity, and did not blind

For peer review only - http://bmjopen.bmj.com/site/about/guidelines.xhtml

participants or those measuring the outcomes, or assess confounding factors between groups. Only 57% of the RCTs included follow-up.

Table 3. Critical appraisal of studies with the highest level of evidence from each medical field.

+ Yes
- No
? Unable to determine
x Not applicable

Medical sector	First author	Date	Reporting										External validity			Internal validity - bias							Internal validity - confounding						Power
			1. Clear hypothesis/aim/objective	2. Clear outcome measures	3. Patient characteristics described	4. Interventions clearly described	5. Distributions of confounders described	6. Findings clearly described	7. Estimates given of random variability	8. Adverse events reported	9. Patients lost to follow-up described	10. Probability values reported	11. Recruitment pool represents population	12. Participants represent population	13. Staff/places/facilities match std. treatment	14. Participants blinded to intervention	15. Those measuring outcomes blinded	16. Data dredging reported	17. Adjusted for different lengths of follow-up	18. Appropriate statistical tests	19. Reliable compliance with intervention	20. Accurate/reliable outcome measures	21. Groups recruited from same population	22. Groups recruited over same timeframe	23. Subjects randomised into intervention	24. Randomised intervention concealed	25. Adjustment for confounding	26. Losses to follow-up accounted for	27. Sufficient power
Neoplasms	de Farias	2014	+	+	+	+	+	+	+	+	x	+	?	?	+	+	+	+	x	+	+	+	+	+	+	+	?	x	+
Oral and Maxillofacial	Ahrberg	2015	+	+	+	+	+	+	+	-	x	+	?	?	+	+	+	+	x	+	+	+	+	+	+	+	+	x	+
Oral and Maxillofacial	D'Urso	1999	+	+	+	+	+	+	+	-	x	-	?	?	+	-	-	+	x	+	+	+	+	+	+	?	-	x	+
Oral and Maxillofacial	Al-Ahmad	2013	+	+	+	+	x	+	+	-	+	+	?	?	+	+	+	+	+	+	+	+	+	+	+	+	+	+	+
Oral and Maxillofacial	Ayoub	2014	+	+	+	+	-	+	+	-	x	-	?	?	+	-	-	+	x	+	+	+	+	+	+	?	-	x	+
Oral and Maxillofacial	Goh	2015	+	+	+	+	-	+	+	-	+	+	?	?	+	-	+	+	+	+	+	+	+	+	+	?	-	+	+
Oral and Maxillofacial	Van de Velde	2010	+	+	+	+	-	+	+	+	+	+	?	?	+	-	+	+	+	+	+	+	+	+	+	?	-	+	+
Oral and Maxillofacial	Vercruyssen	2014	+	+	+	+	-	+	+	+	+	-	?	?	+	-	+	+	+	+	+	+	+	+	+	?	-	+	+
Musculoskeletal	Chareancholvanich	2013	+	+	+	+	-	+	+	+	+	+	?	?	+	-	+	+	-	+	+	+	+	+	+	?	-	+	+
Musculoskeletal	Chen	2015	+	+	+	+	+	+	+	+	+	-	?	?	+	+	-	+	+	+	+	+	+	+	+	+	+	+	+
Musculoskeletal	Du	2012	+	+	-	-	+	+	+	-	+	+	?	?	+	-	-	+	-	+	+	+	+	+	+	?	-	+	+
Musculoskeletal	Gan	2015	+	+	+	+	+	+	+	+	x	+	?	?	+	-	+	+	x	+	+	+	+	+	+	?	-	x	+
Musculoskeletal	Hendel	2012	+	+	-	+	-	+	+	+	-	+	+	+	+	-	+	+	?	+	+	+	+	+	+	-	-	?	+
Musculoskeletal	Maini	2016	+	+	+	+	-	+	+	+	x	+	?	?	+	-	-	+	x	+	+	+	+	+	+	?	-	x	+
Musculoskeletal	Merc	2013	+	+	+	+	+	+	+	+	x	+	+	+	+	-	-	+	x	+	+	+	+	+	+	?	-	x	+
Musculoskeletal	Shuang	2016	+	+	-	+	+	+	+	+	+	+	?	?	+	-	-	+	?	+	+	+	+	+	+	+	+	+	+
Musculoskeletal	Stephens	2016	+	+	+	+	-	+	+	-	+	+	?	?	?	+	+	+	+	+	+	+	+	+	+	+	-	+	+
Musculoskeletal	Wu	2011	+	+	-	+	-	+	+	+	+	+	+	+	+	-	-	+	-	+	+	+	+	+	+	?	-	+	+
Musculoskeletal	Yang	2016	+	+	-	+	-	+	+	+	x	+	?	?	+	-	-	+	x	+	+	+	+	+	+	?	-	x	+
Musculoskeletal	You	2016	+	+	-	+	+	+	+	+	+	+	+	+	+	-	-	+	-	+	+	+	+	+	+	?	+	+	+
Musculoskeletal	Zhang	2011	+	+	-	+	+	+	+	-	+	+	?	?	+	-	-	+	-	+	+	+	+	+	+	?	+	+	+

DISCUSSION

The results show that a wide range of 3D printed devices have been clinically trialled but few papers have rigorously assessed the efficacy or effectiveness of clinical 3D printed devices. The 3D printed devices tested were mainly designed for planning surgeries, particularly using anatomical models or surgical guides to aid cutting or navigating, with little attention dedicated to 3D printed interventions. This is likely due to the lower complexity of the anatomical models and surgical guides and the smaller risk to the patient than therapeutic devices, resulting in less rigorous regulation and safety testing requirements. Approximately twice as many studies were in Oral and Maxillofacial Surgery than in the Musculoskeletal System, the two largest fields, but the Musculoskeletal System, despite its more recent uptake of 3D printing, had almost double the number of Level 1 studies. This is perhaps because clinical trials in musculoskeletal surgery typically aim to demonstrate quantitative clinical outcomes, driving more rapid growth in the level of evidence of studies than oral and maxillofacial surgery where aesthetics is a priority and individual outcomes are harder to quantify.

The RCT listed under Neoplasms was an anatomical model used for planning mandibular surgery, but with a focus on tumour removal, fitting into both categories. Therefore no RCTs were found outside the Oral and Maxillofacial Surgery or Musculoskeletal System fields. The primary indicators of success used to determine

For peer review only - http://bmjopen.bmj.com/site/about/guidelines.xhtml

the efficacy and effectiveness of 3D printed devices across all medical fields were operating time, accuracy of surgery or positioning, fit, changes at the operation site (particularly blood loss, inflammation and aesthetics), recovery time and functional outcomes. From the studies that compared against a control, the 3D printed devices used for preoperative planning and aiding surgery consistently found decreases in operating time and increases in surgical accuracy, the two most commonly reported indicators of effectiveness. Operating time is an obvious choice as an indicator for device success because it is easy to measure and quantify and corresponds to decreased blood loss and faster recovery. The main aims of anatomical models and surgical guides are to provide better information to the surgeon on the surgical site and to guide the surgeon's hand. Therefore measuring the accuracy of the surgeries indicates whether these aims have been met. To make more decisive conclusions regarding the performance of 3D printed medical devices in Oral and Maxillofacial Surgery and Musculoskeletal Surgery it is important to assess the long-term effects. Few papers assessed the long-term differences of using the 3D printed devices and those that did had mixed results as to whether there were any differences in recovery times and outcomes. The other aspect that should be addressed in future studies is the appropriate choice of outcome measures. In the studies analysed in this review there appears to be no evaluation of how the selected outcome measures match the purpose of the device.

Therapeutic devices such as implants, prosthetic limbs and orthotics offer the promise of revolutionising the medical industry because of their ability to be custom-made for the patient, but most therapeutic devices are still early-stage, with little research into their efficacy and effectiveness in a clinical setting. There were not enough therapeutic devices tested against a control group to find and evaluate repeated measures of success.

Most studies reviewed were poor quality, meaning that their study designs demonstrated levels of evidence for the 3D printed device of 4 or 5, with no use of a control group. The 41.4% of papers that reported on individual case studies or provided only qualitative results did not clinically validate the devices that they reported on, and therefore add little value to their fields. Few included follow-up, or addressed the external validity, bias or confounding factors (see supplementary material for full comparison of studies). The vast majority of papers reported positive outcomes for the patients, but few studies demonstrated clinically significant findings. Even the Level 1 studies demonstrated limited external validity and rarely blinded participants or those measuring the outcomes to the intervention.

The RCTs all demonstrated statistically significant results in favour of using the 3D printed device over the current clinical practice to which it was compared. However, one RCT[31] concluded that the improvement with 3D printing was too small to provide a clinical advantage and none performed an analysis on the clinical relevance of these differences. The studies consistently used p-values of <0.05 as their measure of device success, but taking into consideration the false discovery rate, it is likely that this will only show a real effect about 70% of the time under ideal testing conditions.[43] While the critical analysis in Table 3 shows all Level 1 studies as adequately powered, this is because the Quality Index bases its definition of "adequately powered" on whether a difference due to chance is less than 5%. Therefore, given that all Level 1 studies had 40 or fewer participants in each group, many of these studies are likely to be underpowered.[43] Considering the possibility of underpowered studies, the publication bias towards positive trials, and the fact that all RCTs in this review were single site trials, the likelihood that the results shown in these papers reflect a real effect is lower than suggested by the reviewed studies.[44] Until independent groups start validating 3D printed devices, it is also difficult to avoid researcher bias.

Most 3D printed device studies that were excluded from this review had uses in medical training, or as moulds for manufacturing and therefore did not meet the inclusion requirements. A few other studies were excluded because they had not been translated into English.

Previous reviews have documented the uses of 3D printing for developing patient-specific medical devices. However, there has been limited research into assessing the efficacy and effectiveness of these devices. This systematic review is the first to compare 3D printed devices across all medical fields and assess their efficacy and effectiveness, describing key benefits that have been found from using 3D printed devices clinically. It demonstrates that the fields of Oral and Maxillofacial Surgery and the Musculoskeletal System are leading the way in validating 3D printed devices for clinical use. Multiple high-quality studies have been performed on surgical guides for maxillofacial, hip and knee surgeries. This growing body of comparable high-quality research sets an example for other fields to emulate in order to demonstrate the efficacy and effectiveness required to integrate these 3D printed devices into clinical practice.

For peer review only - http://bmjopen.bmj.com/site/about/guidelines.xhtml

A critical appraisal of the efficacy and effectiveness of 3D printed devices across medical fields provides clinicians with an evidence-based approach to determine the applicability of 3D printing within their field. It also gives researchers an overview of areas that require further research and validation. It encourages investigators to discover what methods have been successfully validated in other medical fields and promotes potential collaborations between fields.

3D printing provides a way of customising devices to improve patient outcomes. Available techniques and materials will increase, as 3D printing technology continues to be developed. There is therefore a growing need for validation of new devices, materials and techniques to ensure best patient outcomes. Much research has already gone into developing 3D printed devices for medical purposes. However, this drive for new technology development has not yet been matched with a drive for critical appraisal of the devices to demonstrate their efficacy and effectiveness. Even the fields that are leading the way in critically evaluating new 3D printed devices will be required to increase their output as 3D printing continues to grow in popularity and functionality.

The early research covered by this review shows that 3D printing can be valuable for use in medicine. The next important step to take is growing the body of research that focuses on validating 3D printed devices. All fields require more rigorous and long-term assessments into the efficacy, effectiveness and safety of 3D printed devices before they are introduced into standard clinical practice. The study is limited to a critical appraisal of individual studies, rather than a meta-analysis, because of the breadth of uses for anatomical models, surgical guides and therapeutic devices and the lack of comparable hypotheses. Funders can take an active role in promoting not only early technological development, but also the subsequent clinical trials. Demonstration of clinical efficacy, effectiveness and device safety will become increasingly important as higher risk 3D printed devices are developed and unconventional manufacturers, such as hospitals and clinics, incorporate 3D printing of patient-specific medical devices into standard clinical practice.

Fields such as Oral and Maxillofacial surgery, who were early in the uptake of 3D printing, are beginning to stabilise, with a steady number of studies being published each year, but other fields, such as Musculoskeletal and Circulatory Systems, are more recently gaining traction, with increasing numbers of studies performed each year. 3D printed drug delivery devices and biological 3D printing technologies for printing tissue show huge promise, but have not been clinically trialled and so are not included here.[45] This is likely to change within the near future. It is therefore recommended that in this fast-growing and dynamic environment, this review is updated every few years.

CONCLUSION

This review demonstrates that 3D printing is already being used to develop a broad range of medical devices with clinically effective results. The medical fields of Oral and Maxillofacial Surgery and the Musculoskeletal System are leading the way in validating the efficacy and effectiveness of 3D printed devices, and have found that 3D printed anatomical models and surgical guides are reducing operating times and increasing surgical accuracy. However, the efficacy and effectiveness of 3D printed devices remains undetermined for the majority of medical fields. 3D printed devices can have an important role to play in healthcare, but more rigorous and long-term assessments are needed to determine if 3D printed devices are clinically relevant before these devices can become part of standard clinical practice.

Contributors: LD, JB and MT designed the review. LD and JB collected and analysed the data with input from MT. LD drafted the manuscript. All authors contributed to subsequent drafts and approved the final version of the manuscript.

Funding: The researchers are financially supported by the General Sir John Monash Foundation and the Wellcome Trust.

Competing interests: None declared

Data sharing statement: All additional data is available in the supplementary material.

For peer review only - http://bmjopen.bmj.com/site/about/guidelines.xhtml

REFERENCES

1. Zadpoor AA, Malda J. Additive Manufacturing of Biomaterials, Tissues, and Organs. Ann Biomed Eng. 2017;45(1):1–11.

2. Banks J. Adding value in additive manufacturing: Researchers in the United Kingdom and Europe look to 3D printing for customization. IEEE Pulse. 2013;4(6):22–6.

3. Royal Academy of Engineering. Additive Manufacturing : Opportunities and Constraints [Internet]. Royal Academy of Engineering. 2013. Available from: http://www.raeng.org.uk/publications/reports/additive-manufacturing

4. Lee Ventola C. Medical applications for 3D printing: Current and projected uses. P T. 2014;39(10):704–11.

5. Tack P, Victor J, Gemmel P, Annemans L. 3D-printing techniques in a medical setting: a systematic literature review. Biomed Eng Online. 2016;15(1):115.

6. Caffrey T, Wohlers T. Additive manufacturing state of the industry. Manuf Eng. 2015;154(5):67–78.

7. Mulford JS, Babazadeh S, Mackay N. Three-dimensional printing in orthopaedic surgery: Review of current and future applications. ANZ J Surg. 2016;

8. Revicki DA, Frank L. Pharmacoeconomic evaluation in the real world: Effectiveness versus efficacy studies. Vol. 15, PharmacoEconomics. 1999. p. 423–34.

9. FDA. Technical Considerations for Additive Manufactured Devices - Draft Guidance for Industry and Food and Drug Administration Staff. 2016.

10. Bauermeister AJ, Zuriarrain A, Newman MI. Three-Dimensional Printing in Plastic and Reconstructive Surgery: A Systematic Review. Ann Plast Surg. 2015;

11. Soon DSC, Chae MP, Pilgrim CHC, Rozen WM, Spychal RT, Hunter-Smith DJ. 3D haptic modelling for preoperative planning of hepatic resection: A systematic review. Vol. 10, Annals of Medicine and Surgery. 2016. p. 1–7.

12. Patzelt SBM, Spies BC, Kohal RJ. CAD/CAM-fabricated implant-supported restorations: A systematic review. Clin Oral Implants Res. 2015;26:77–85.

13. Bidra AS, Taylor TD, Agar JR. Computer-aided technology for fabricating complete dentures: Systematic review of historical background, current status, and future perspectives. Vol. 109, Journal of Prosthetic Dentistry. 2013. p. 361–6.

14. Tarsitano A, Del Corso G, Ciocca L, Scotti R, Marchetti C. Mandibular reconstructions using computer-aided design/computer-aided manufacturing: A systematic review of a defect-based reconstructive algorithm. J Cranio-Maxillofacial Surg. 2015;43(9):1785–91.

15. Martelli N, Serrano C, Van Den Brink H, Pineau J, Prognon P, Borget I, et al. Advantages and disadvantages of 3-dimensional printing in surgery: A systematic review. Vol. 159, Surgery (United States). 2016. p. 1485–500.

16. Zhang QM, Chen JY, Li H, Chai W, Ni M, Zhang ZD, et al. No Evidence of Superiority in Reducing Outliers of Component Alignment for Patient-specific Instrumentation for Total Knee Arthroplasty: A Systematic Review. Vol. 7, Orthopaedic Surgery. 2015. p. 19–25.

17. Liberati A, Altman DG, Tetzlaff J, Mulrow C, Gøtzsche PC, Ioannidis JPA, et al. The PRISMA statement for reporting systematic reviews and meta-analyses of studies that evaluate health care interventions: explanation and elaboration. In: Journal of clinical epidemiology. 2009. p. e1-34.

18. Oxford Centre for Evidence-based Medicine. Levels of Evidence. Univ Oxford [Internet]. 2009;4–5. Available from: www.cebm.net

19. World Health Organization. International Statistical Classification of Diseases and Related Health Problems (ICD) 10th Revision - Version:2010 [Internet]. Occupational Health. 2010. Available from: http://apps.who.int/classifications/icd10/browse/2010/en

20. Higgins JPT, Green S. Cochrane Handbook for Systematic Reviews of Interventions Version 5.1.0

For peer review only - http://bmjopen.bmj.com/site/about/guidelines.xhtml

[updated March 2011]. Vol. 4, The Cochrane Collaboration. 2011.

21. Downs SH, Black N. The feasibility of creating a checklist for the assessment of the methodological quality both of randomised and non-randomised studies of health care interventions. J Epidemiol Community Health. 1998;52(6):377–84.

22. Stephens MH, Grey A, Fernandez J, Kalluru R, Faasse K, Horne A, et al. 3-D bone models to improve treatment initiation among patients with osteoporosis: A randomised controlled pilot trial. Psychol Health. 2016;31(4):487–97.

23. de Farias TP, Dias FL, Galvão MS, Boasquevisque E, Pastl AC, Albuquerque Sousa B. Use of prototyping in preoperative planning for patients with head and neck tumors. Head Neck. 2013;1–31.

24. Ahrberg D, Lauer HC, Ahrberg M, Weigl P. Evaluation of fit and efficiency of CAD/CAM fabricated all-ceramic restorations based on direct and indirect digitalization: a double-blinded, randomized clinical trial. Clin Oral Investig. 2015;

25. D'Urso PS, Barker TM, Earwaker WJ, Bruce LJ, Atkinson RL, Lanigan MW, et al. Stereolithographic biomodelling in cranio-maxillofacial surgery: a prospective trial. J Craniomaxillofac Surg. 1999;27(1):30–7.

26. Al-Ahmad HT, M Saleh MW, Hussein AM. Evaluation of an innovative computer-assisted sagittal split ramus osteotomy to reduce neurosensory alterations following orthognathic surgery: a pilot study. Int J Med Robot. 2013;9(2):134–41.

27. Ayoub N, Ghassemi A, Rana M, Gerressen M, Riediger D, Hölzle F, et al. Evaluation of computer-assisted mandibular reconstruction with vascularized iliac crest bone graft compared to conventional surgery: a randomized prospective clinical trial. Trials. 2014;15:114.

28. Goh BT, Teh LY, Tan DBP, Zhang Z, Teoh SH. Novel 3D polycaprolactone scaffold for ridge preservation - a pilot randomised controlled clinical trial. Clin Oral Implants Res. 2015;26(3):271–7.

29. Van de Velde T, Sennerby L, De Bruyn H. The clinical and radiographic outcome of implants placed in the posterior maxilla with a guided flapless approach and immediately restored with a provisional rehabilitation: A randomized clinical trial. Clin Oral Implants Res. 2010;21(11):1223–33.

30. Vercruyssen M, Cox C, Coucke W, Naert I, Jacobs R, Quirynen M. A randomized clinical trial comparing guided implant surgery (bone- or mucosa-supported) with mental navigation or the use of a pilot-drill template. J Clin Periodontol. 2014;41(7):717–23.

31. Chareancholvanich K, Narkbunnam R, Pornrattanamaneewong C. A prospective randomised controlled study of patient-specific cutting guides compared with conventional instrumentation in total knee replacement. Bone Joint J. 2013;95–B:354–9.

32. Chen HL, Wu DY, Yang HL, Guo KJ. Clinical Use of 3D Printing Guide Plate in Posterior Lumbar Pedicle Screw Fixation. Med Sci Monit. 2015;21:3948–54.

33. Du H, Tian XX, Li T Sen, Yang JS, Li KH, Pei GX, et al. Use of patient-specific templates in hip resurfacing arthroplasty: Experience from sixteen cases. Int Orthop. 2013;37(5):777–82.

34. Gan Y, Ding J, Xu Y, Hou C. Accuracy and efficacy of osteotomy in total knee arthroplasty with patient-specific navigational template. Int J Clin Exp Med. 2015;8(8):12192–201.

35. Hendel MD, Bryan J a, Barsoum WK, Rodriguez EJ, Brems JJ, Evans PJ, et al. Comparison of patient-specific instruments with standard surgical instruments in determining glenoid component position: a randomized prospective clinical trial. J Bone Joint Surg Am. 2012;94(23):2167–75.

36. Maini L, Sharma A, Jha S, Sharma A, Tiwari A. Three-dimensional printing and patient-specific pre-contoured plate: future of acetabulum fracture fixation? Eur J Trauma Emerg Surg. 2016;1–10.

37. Merc M, Drstvensek I, Vogrin M, Brajlih T, Recnik G. A multi-level rapid prototyping drill guide template reduces the perforation risk of pedicle screw placement in the lumbar and sacral spine. Arch Orthop Trauma Surg. 2013;133(7):893–9.

38. Shuang F, Hu W, Shao Y, Li H, Zou H. Treatment of Intercondylar Humeral Fractures With 3D-Printed Osteosynthesis Plates. Medicine (Baltimore). 2016;95(3):e2461.

For peer review only - http://bmjopen.bmj.com/site/about/guidelines.xhtml

39. Wu Z-X, Huang L-Y, Sang H-X, Ma Z-S, Wan S-Y, Cui G, et al. Accuracy and safety assessment of pedicle screw placement using the rapid prototyping technique in severe congenital scoliosis. J spinal Disord {&} Tech. 2011;24(7):444–50.

40. Yang L, Shang XW, Fan JN, He ZX, Wang JJ, Liu M, et al. Application of 3D Printing in the Surgical Planning of Trimalleolar Fracture and Doctor-Patient Communication. Biomed Res Int. 2016;2016.

41. You W, Liu LJ, Chen HX, Xiong JY, Wang DM, Huang JH, et al. Application of 3D printing technology on the treatment of complex proximal humeral fractures (Neer3-part and 4-part) in old people. Orthop Traumatol Surg Res. 2016;

42. Zhang YZ, Chen B, Lu S, Yang Y, Zhao JM, Liu R, et al. Preliminary application of computer-assisted patient-specific acetabular navigational template for total hip arthroplasty in adult single development dysplasia of the hip. Int J Med Robot Comput Assist Surg. 2011;7(4):469–74.

43. Colquhoun D. An investigation of the false discovery rate and the misinterpretation of p-values. R Soc Open Sci [Internet]. 2014;1(3):140216–140216. Available from: http://rsos.royalsocietypublishing.org/cgi/doi/10.1098/rsos.140216

44. Ioannidis JPA. Why most published research findings are false. Vol. 2, PLoS Medicine. 2005. p. 0696–701.

45. Shafiee A, Atala A. Printing Technologies for Medical Applications. Trends Mol Med. 2016;22(3):254–65.

FIGURE LEGENDS

Figure 1. Flow chart of the selection and sorting method

Figure 2. Number of studies per level of evidence given by medical field. Levels of evidence are colour coded and fields are visually separated by alternating between white and grey columns.

For peer review only

For peer review only - http://bmjopen.bmj.com/site/about/guidelines.xhtml

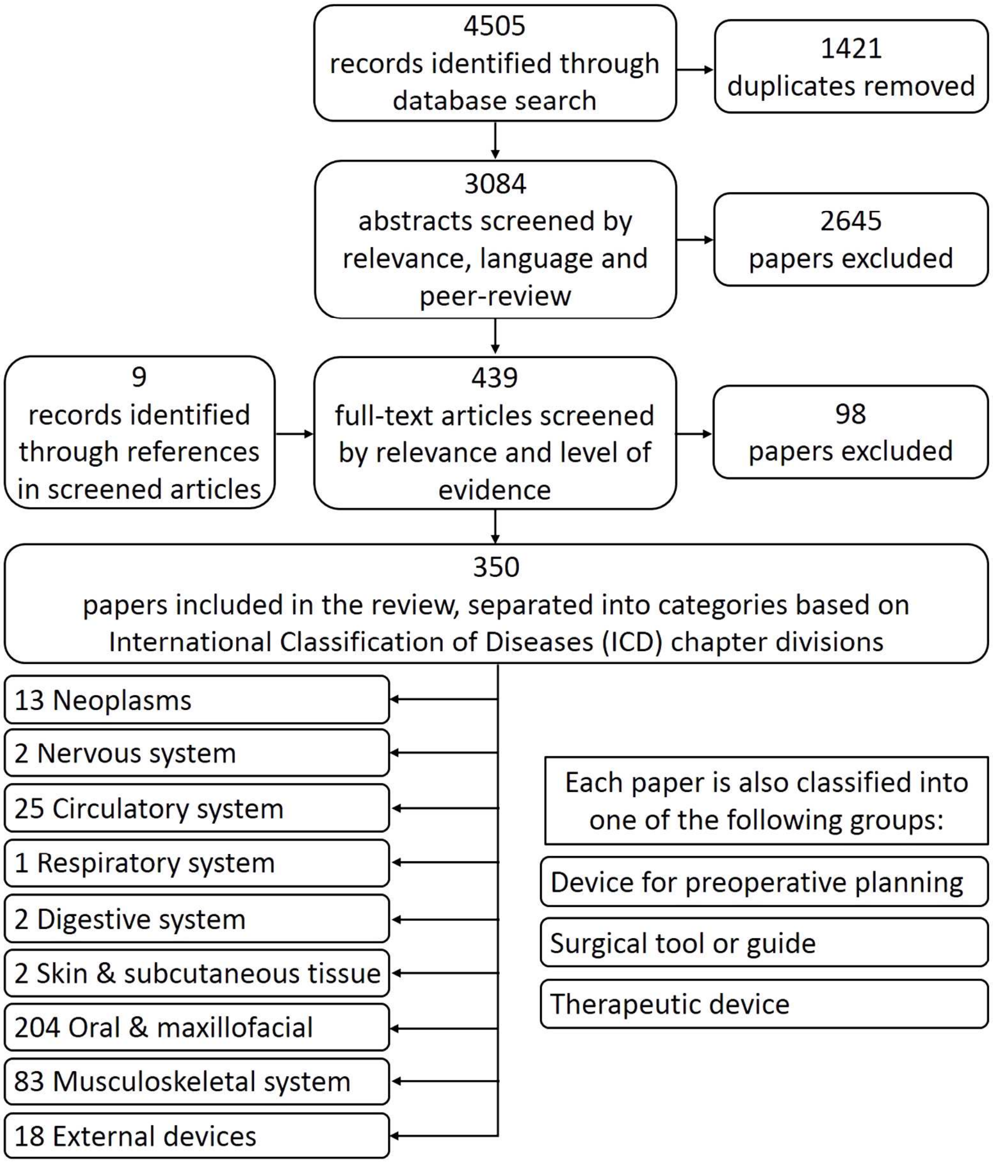

Figure 1. Flow chart of the selection and sorting method.

226x266mm (300 x 300 DPI)

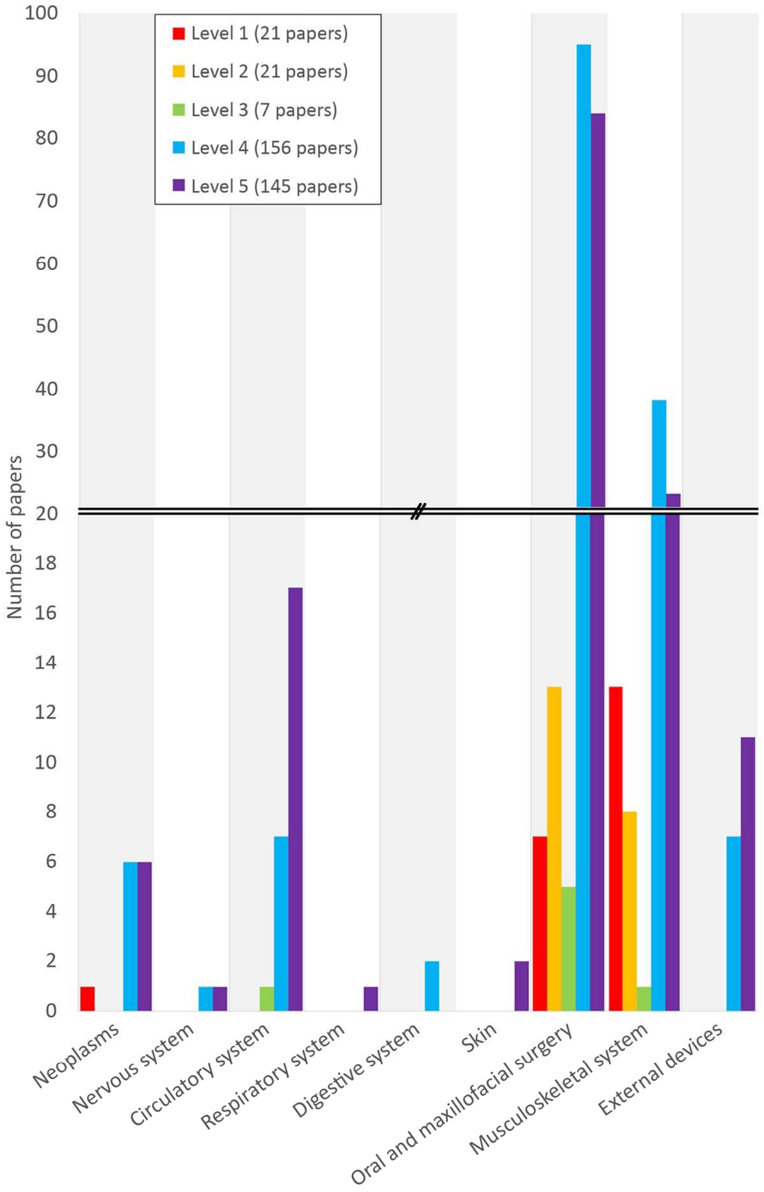

Figure 2. Number of studies per level of evidence given by medical field. Levels of evidence are colour coded and fields are visually separated by alternating between white and grey columns.

164x246mm (300 x 300 DPI)

1. Database search

IEEE Xplore and Google Scholar required a simplified search.

Database	PubMed (All article types)	ISI Web of Science (Article, review, case report, clinical trial, book or report)	OVID (Journal OR book)	IEEE Xplore (All article types)	Google Scholar (Exclude patents and citations)
Search Term	"**health care evaluation mechanisms**" OR "randomized controlled trial" OR "randomised controlled trial" OR "clinical trial" OR (efficacy OR effectiveness OR validation OR validity OR feasibility OR cohort OR observational OR longitudinal OR retrospective OR comparative OR "exploratory study" OR "case-control study" OR "case study" AND (clinical OR medical OR hospital OR biomedical OR assistive OR "health care" OR patients OR patient)) AND ("**Printing, Three-Dimensional**" OR "Three Dimensional Printing" OR "Three-Dimensional Printing" OR "3 Dimensional Printing" OR "3D Printing" OR "3-D Printing" OR stereolithography OR "laser melting" OR "electron beam melting" OR "fused deposition modeling" OR "fused deposition modelling" OR "rapid manufacturing" OR "rapid prototyping" OR "layered manufacturing" OR "digital manufacturing" OR bioprinting OR "3D prototyping" OR "3D fabrication" OR "rapid fabrication" OR "freeform fabrication")			randomized controlled trial OR "clinical trial" OR (efficacy OR validation OR feasibility OR "case study" AND (clinical OR medical OR hospital OR assistive OR "health care")) AND ("Three Dimensional Printing" OR "3D Printing" OR "rapid prototyping" OR "additive manufacturing")	"randomized controlled" OR "clinical trial" efficacy OR validation OR feasibility OR "case study" clinical OR medical OR hospital OR assistive OR "health care" "Three Dimensional Printing" OR "3D Printing" OR "rapid prototyping" OR "additive manufacturing"
**Number of studies**	913	700	1158	754	980

Number of studies: 4505

2. Duplicate removal

The first four databases were combined, then relevant additional results from Google Scholar were added.

Number of studies: 3084

For peer review only - http://bmjopen.bmj.com/site/about/guidelines.xhtml

Medical sector	Level of evidence	First author	Date	Device	Category	No. of participants	Participant details	Title	Aim	Outcomes	1. Clear hypothesis/aim/objective	2. Clear outcome measures	3. Patient characteristics described	4. Interventions clearly described	5. Distributions of confounders described	6. Findings clearly described	7. Estimates given of random variability	8. Adverse events reported	9. Patients lost to follow-up described	10. Probability values reported	11. Recruitment pool represents population	12. Participants represent population	13. Staff/places/facilities match std. treatment	14. Participants blinded to intervention	15. Those measuring outcomes blinded	16. Data dredging reported	17. Adjusted for different lengths of follow-up	18. Appropriate statistical tests	19. Reliable compliance with intervention	20. Accurate/reliable outcome measures	21. Groups recruited from same population	22. Groups recruited over same timeframe	23. Subjects randomised into intervention	24. Randomised intervention concealed	25. Adjustment for confounding	26. Losses to follow-up accounted for	27. Sufficient power
Neoplasms	1	de Farias	2014	Mandible model	P	37 (Test=17, Control=20)	Age: 9-74 years (Test mean=39.57 years, Control mean=53.11 years) Test=14 male, 3 female, Control=15 male, 5 female	Use of prototyping in preoperative planning for patients with head and neck tumors	Aim: to evaluate the efficacy of using AM models to plan head and neck surgeries.	+ The average reconstruction time decreased; Test=43.7 minutes, Control=127.7 minutes, p=0.001). Though significance was not given, the size of the bone flap taken for reconstruction was typically smaller, and the aesthetic results better in the test group.	⊞	⊞	⊞	⊞	⊞	⊞	⊞	⊞	X	⊞	?	?	⊞	⊞	⊞	⊞	X	⊞	⊞	⊞	⊞	⊞	⊞	⊞	?	X	⊞
Oral and Maxillofacial	1	Ahrberg	2015	All-ceramic zirconia crowns	T	25 (Same participants used for test and control)	Age: >18 years 10 male, 15 female	Evaluation of fit and efficiency of CAD/CAM fabricated all-ceramic restorations based on direct and indirect digitalization: a double-blinded, randomized clinical trial.	Aim: to evaluate the marginal and internal fit of AM fabricated zirconia crowns and three-unit fixed dental prostheses resulting from direct versus indirect digitalization.	+ The mean for the marginal gap was significantly (p<0.05) smaller 61.08 μm (±24.77 μm) for the test group compared with the control 70.40 μm (±28.87 μm). The test group showed significantly lower values of internal fit at the centro-occlusal site.	⊞	⊞	⊞	⊞	⊞	⊞	⊞	⊟	X	⊞	?	?	⊞	⊞	⊞	⊞	X	⊞	⊞	⊞	⊞	⊞	⊞	⊞	⊞	X	⊞
Oral and Maxillofacial	1	D'Urso	1999a	Cranio-maxillofacial model	P	30 (Test=15, Control=15)	No information provided	Stereolithographic biomodelling in cranio-maxillofacial surgery: a prospective trial	Aim: Assess whether biomodels in addition to standard imaging have greater utility in the surgery performed than the standard imaging alone.	+ Biomodels significantly improved operative planning, diagnosis and measurement accuracy. Operating time was also reduced by an estimated 17.63%.	⊞	⊞	⊞	⊞	⊞	⊞	⊞	⊟	X	⊟	?	?	⊞	⊟	⊟	⊞	X	⊞	⊞	⊞	⊞	⊞	⊞	?	⊟	X	⊞
Oral and Maxillofacial	1	Al-Ahmad	2013	Surgical guide for sagittal splitting ramus osteotomy	S	8 (One side used as test, the other as control)	Age: 18-30 years (mean=23 years)	Evaluation of an innovative computer-assisted sagittal split ramus osteotomy to reduce neurosensory alterations following orthognathic surgery: a pilot study	Aim: to evaluate the effectiveness of SSRO in reducing the incidence and severity of neurosensory alterations, using an AM surgical guide.	+ The test patients had lower postoperative abnormal thresholds for the Semmes–Weinstein monofilaments on lower lip and chin (p<0.05 at 3 months) and for the two-point discrimination on lower lip (p<0.05 at 1week) and chin (p<0.05 at 6 months), with fewer abnormal self-reported changes in lower lip sensation (p<0.05 at 1week) after surgery.	⊞	⊞	⊞	⊞	X	⊞	⊞	⊟	⊞	⊞	?	?	⊞	⊞	⊞	⊞	⊞	⊞	⊞	⊞	⊞	⊞	⊞	⊞	⊞	⊞	⊞
Oral and Maxillofacial	1	Ayoub	2014	Guide for mandibular reconstruction	S	20 (Test=10, Control=10)	Age: Test=52.3 ± 19.2 years, Control=54.7 ± 14.2 years) Test=4 male, 6 female, Control=6 male, 4 female	Evaluation of computer-assisted mandibular reconstruction with vascularized iliac crest bone graft compared to conventional surgery: a randomized prospective clinical trial	Aim: to evaluate the benefits of computer-assisted mandibular reconstruction with iliac crest bone grafts regarding the intraoperative time for transplant shaping, ischemia, duration of surgery, amount of bone removed and the hange in postoperative condyle position compared to conventional surgery.	+ Computer-assisted surgery shortened the time of transplant ischemia (p<0.005) and defect reconstruction (p<0.001) compared to conventional surgery. There was less bone harvested in the test group (p<0.001), and the intercondylar distance before compared to after surgery was less affected in the computer-assisted than in the conventional group (p<0.001).	⊞	⊞	⊞	⊞	⊟	⊞	⊞	⊟	X	⊟	?	?	⊞	⊟	⊟	⊞	X	⊞	⊞	⊞	⊞	⊞	⊞	?	⊟	X	⊞
Oral and Maxillofacial	1	Goh	2015	Polycaprolactone scaffold for ridge preservation	T	13 (Test=6, Control=7)	Age: 29–60 years (mean=46.8 years) 7 male, 7 female (1 was lost to follow-up, so was excluded from results)	Novel 3D polycaprolactone scaffold for ridge preservation – a pilot randomised controlled clinical trial	Aim: to evaluate the feasibility and effectiveness of using a polycaprolactone scaffold in fresh extraction sockets for ridge preservation.	+ The test group showed less vertical ridge resorption (significant in the mesio-buccal aspect: p = 0.008), with better maintenance of ridge height after 6 months.	⊞	⊞	⊞	⊞	⊟	⊞	⊞	⊟	⊞	⊞	?	?	⊞	⊟	⊞	⊞	⊞	⊞	⊞	⊞	⊞	⊞	⊞	?	⊟	⊞	⊞
Oral and Maxillofacial	1	Van de Velde	2010	Maxilla surgical guide	S	13 (Test=36 tooth implants, Control=34 tooth implants)	Age: 39-75 years (mean =55.7 years) 4 male, 9 female	The clinical and radiographic outcome of implants placed in the posterior maxilla with a guided flapless approach and immediately restored with a provisional rehabilitation: a randomized clinical trial	Aim: to compare the outcome of dental implants placed using a flapless protocol with an AM guide and immediate loading with a conventional protocol and loading.	+ Implants can successfully integrate in the posterior maxilla using a flapless approach with immediate loading. The mucosal tissues around implants changed significantly less compared with controls (p<0.05). Marginal bone levels were not significantly different between test and control implants. Opinion about speech, function, aesthetics and self-confidence was significantly better for the test side after 1 week (p<0.05).	⊞	⊞	⊞	⊞	⊟	⊞	⊞	⊞	⊞	⊞	?	?	⊞	⊟	⊞	⊞	⊞	⊞	⊞	⊞	⊞	⊞	⊞	?	⊟	⊞	⊞
Oral and Maxillofacial	1	Vercruyssen	2014	Surgical guides for edentulous jaws	S	59 (72 jaws)	Age:31-78 years 31 male jaws, 41 female jaws	A randomized clinical trial comparing guided implant surgery (bone- or mucosasupported) with mental navigation or the use of a pilot-drill template	Aim: To assess the accuracy of guided surgery compared to mental navigation or the use of a surgical template, in fully edentulous jaws.	+ Significantly lower mean deviation at the entry point, at the apex and angular deviation when using the AM guiding systems than when using mental navigation or a conventional surgical template (p<0.05). Differences between bone and mucosa support or type of guidance were negligible.	⊞	⊞	⊞	⊞	⊟	⊞	⊞	⊞	⊞	⊟	?	?	⊞	⊟	⊞	⊞	⊞	⊞	⊞	⊞	⊞	⊞	⊞	?	⊟	⊞	⊞
Musculoskeletal	1	Chareancholvanich	2013	Cutting guides for total knee replacement	S	80 (Test=40, Control=40)	Age: 55-84 years (mean: 69.5 years) 6 male, 34 female	A prospective randomised controlled study of patient-specific cutting guides compared with conventional instrumentation in total knee replacement	Aim: to compare patient-specific cutting guides with conventional instrumentation in total knee replacement.	+ The tibial component in the test group was closer to neutral alignment (p = 0.030), but the size of the difference was small (89.8°±1.2° vs 90.5°±1.6°). The AM guides also shortened the bone-cutting time by a 3.6 minutes (p < 0.001) and the operating time by 5.1 minutes (p = 0.019), without differences in post-operative blood loss (p = 0.528) or need for blood transfusion (p = 0.789). These minimal advantages of the AM guides are unlikely to be clinically relevant.	⊞	⊞	⊞	⊞	⊟	⊞	⊞	⊞	⊞	⊞	?	?	⊞	⊟	⊞	⊞	⊟	⊞	⊞	⊞	⊞	⊞	⊞	?	⊟	⊞	⊞
Musculoskeletal	1	Chen	2015	Guide plate for pedicle screw fixation	S	43 (Test=20, Control=23)	Age: Test=35–70 years (mean=52.3 years) Control=37–72 years (mean=55.4 years) Test=9 male, 11 female Control=12 male, 11 female	Clinical Use of 3D Printing Guide Plate in Posterior Lumbar Pedicle Screw Fixation.	Aim: to evaluate the clinical efficacy of use of a 3D printing guide plate in posterior lumbar pedicle screw fixation.	+ The time of placement for each screw was shorter, the amount of hemorrhage was less, and the fluoroscopy times of each screw placement was lower in the test group than in the control group (all p<0.05). The excellent and good screw placement rate was 100% in the test group and 98.4% in the control group, without any statistical difference (p>0.05). No obvious complications were reported in either group.	⊞	⊞	⊞	⊞	⊞	⊞	⊞	⊞	⊞	⊟	?	?	⊞	⊞	⊟	⊞	⊞	⊞	⊞	⊞	⊞	⊞	⊞	⊞	⊞	⊞	⊞
Musculoskeletal	1	Du	2013	Templates to aid in pin placement in hip resurfacing arthroplasty	S	34 (Test-16, Control=18)	Ages: 37-55 years	Use of patient-specific templates in hip resurfacing arthroplasty: experience from sixteen cases	Aim: To assess whether AM patient-specific templates aid in accurate intraoperative pin placement.	+ The prosthesis stem shaft angle was greater for the test group (p=0.001). The locating template can provide precise and dependable location for hip resurfacing femoral components during arthroplasty and ensure the valgus stem placement necessary for optimal outcomes.	⊞	⊞	⊟	⊟	⊞	⊞	⊞	⊟	⊞	⊞	?	?	⊞	⊟	⊟	⊞	⊟	⊞	⊞	⊞	⊞	⊞	⊞	?	⊟	⊞	⊞
Musculoskeletal	1	Gan	2015	Navigational template for total knee arthroplasty	S	70 (Test=35, Control=35)	Age: Test= 63-75 years (mean=68.5±4.8 years), Control=64-72 years (mean=67.8±3.4 years) Test=10 male, 25 female, Control=9 male, 26 female	Accuracy and efficacy of osteotomy in total knee arthroplasty with patient-specific navigational template	Aim: to validates a novel patient-specific navigational template for total knee arthroplasty.	+ The AM navigational template significantly reduced errors, operation time and blood loss (p<0.05).	⊞	⊞	⊞	⊞	⊞	⊞	⊞	⊞	X	⊞	?	?	⊞	⊟	⊞	⊞	X	⊞	⊞	⊞	⊞	⊞	⊞	?	⊟	X	⊞
Musculoskeletal	1	Hendel	2012	Guide for determining glenoid component position	S	31 (Test=15, Control=16)	No information provided	Comparison of Patient-Specific Instruments with Standard Surgical Instruments in Determining Glenoid Cmponent Position	Aim: to compare patient-specific instruments with standard surgical instruments in determining glenoid component position.	+ The AM technology significantly decreased (p < 0.05) the average deviation of implant position for inclination and medial-lateral offset. The greatest benefit was observed in patients with retroversion >16˚ (p < 0.001).	⊞	⊞	⊟	⊞	⊟	⊞	⊞	⊞	⊟	⊞	⊞	⊞	⊞	⊟	⊞	⊞	?	⊞	⊞	⊞	⊞	⊞	⊞	⊟	⊟	?	⊞
Musculoskeletal	1	Maini	2016	Plate for acetabulum fracture fixation	P	21 (Test=10, Control=11)	Age: 18-60 years (mean=38.7 years) Test = 9 male, 1 female Control = 9 male, 2 female	Three-dimensional printing and patient-specific pre-contoured plate: future of acetabulum fracture fixation?	Aim: to evaluate the accuracy of patient-specific pre-contoured plates.	+ Reduction achieved, as evaluated by CT scan, was more in the test group than the control group (p<0.05). Reduced blood loss (100 ml less) and surgical time (12 min less) were observed in the test group but the results were not significant (p>0.05). All the pre-contoured plates fitted well to the pelvis intra-operatively.	⊞	⊞	⊞	⊞	⊟	⊞	⊞	⊞	X	⊞	?	?	⊞	⊟	⊟	⊞	X	⊞	⊞	⊞	⊞	⊞	⊞	?	⊟	X	⊞
Musculoskeletal	1	Merc	2013	drill guide for screw placement	S	19 (Test=9 (54 screws), Control=10(54 screws))	Age: Test=59 ± 5 years, Control=62 ±12 years Test=4 male, 5 female, Control=5 male, 5 female	A multi-level rapid prototyping drill guide template reduces the perforation risk of pedicle screw placement in the lumbar and sacral spine	Aim: to evaluate the accuracy of a multi-level drill guide template for lumbar and first sacral pedicle screw placement and to compare it with the freehand technique under fluoroscopy supervision.	+ The incidence of cortex perforation was significantly reduced in the test group. So was the deviation and displacement level of screws in the sagital plane but not in the transverse plane.	⊞	⊞	⊞	⊞	⊞	⊞	⊞	⊞	X	⊞	⊞	⊞	⊞	⊟	⊟	⊞	X	⊞	⊞	⊞	⊞	⊞	⊞	?	⊟	X	⊞
Musculoskeletal	1	Shuang	2016	Osteosynthesis Plates	T	13 (Test=6, Control=7)	Age: Test=31-62 years (mean=46.2 years) Control=27-59 years (mean=40.3 years) Test= 4 male, 2 female, Control=6 male, 1 female	Treatment of Intercondylar Humeral Fractures With 3D-Printed Osteosynthesis Plates.	Aim: to evaluate the efficacy custom AM osteosynthesis plates in the treatment of intercondylar humeral fractures.	+ Operative time was shorter for the test group than the control group (p=0.026). The mean time to bone union was 3.4 months. The 2 groups showed no significant difference in the rate of patients with excellent or good elbow function, or in the ranges of elbow flexion/extension and pronation/supination (p>0.05).	⊞	⊞	⊟	⊞	⊞	⊞	⊞	⊞	⊞	⊞	?	?	⊞	⊟	⊟	⊞	?	⊞	⊞	⊞	⊞	⊞	⊞	⊞	⊞	⊞	⊞
Musculoskeletal	1	Stephens	2016	Bone models	P	58 (Test=29, Control=29)	Age: >60 years (mean=73 years) 5 male, 53 female	3-D bone models to improve treatment initiation among patients with osteoporosis: A randomised controlled pilot trial.	Aim: to investigate the efficacy of AM bone models as a tool to facilitate initiation of bisphosphonate treatment among individuals who are newly diagnosed with osteoporosis.	+ The test group were more emotionally affected by osteoporosis immediately after the interview (p=0.04) and reported a greater understanding of osteoporosis at follow-up (p=0.04), than the control group.	⊞	⊞	⊞	⊞	⊟	⊞	⊞	⊟	⊞	⊞	?	?	?	⊞	⊞	⊞	⊞	⊞	⊞	⊞	⊞	⊞	⊞	⊞	⊟	⊞	⊞
Musculoskeletal	1	Wu	2011	Spine model	P	62 (Test=34, Control=28) 677 screws inserted	Age: 4-22 years (mean=11 years)	Accuracy and Safety Assessment of Pedicle Screw Placement Using the Rapid Prototyping Technique in Severe Congenital Scoliosis	Aim: to compare the accuracy and safety of pedicle screw placement in congenital scoliosis using the AM technique versus conventional fluoroscopy.	+ Accuracy of placement = 89.7% (84.7% in control group; 93.5% in test group). Compared with the fluoroscopy method, the AM assisted technique had a shorter operation time and higher scoliosis correction rate (p<0.05).	⊞	⊞	⊟	⊞	⊟	⊞	⊞	⊞	⊞	⊞	⊞	⊞	⊞	⊟	⊟	⊞	⊟	⊞	⊞	⊞	⊞	⊞	⊞	?	⊟	?	⊞
Musculoskeletal	1	Yang	2016	Model of trimalleolar fracture	P	30 (Test=15, Control=15)	Age: 31-42 years (mean=36.5 years) 16 male, 14 female	Application of 3D Printing in the Surgical Planning of Trimalleolar Fracture and Doctor-Patient Communication.	Aim: to evaluate the effectiveness of using AM models for surgical planning in treating trimalleolar fractures and in physician-patient communication.	+ Both the operation time and the intraoperative blood loss were less in the test group than in the control group (p<0.05). Patient satisfaction with using the AM device for communication was scored highly.	⊞	⊞	⊟	⊞	⊟	⊞	⊞	⊞	X	⊞	?	?	⊞	⊟	⊟	⊞	X	⊞	⊞	⊞	⊞	⊞	⊞	?	⊟	X	⊞
Musculoskeletal	1	You	2016	Proximal humeral fracture model	P	66 (Test=34, Control=32)	61-76 years (mean for Test = 66.09 ± 4.09 years, mean for Control = 66.28 ± 4.10 years) 27 male, 39 female	Application of 3D printing technology on the treatment of complex proximal humeral fractures (Neer3-part and 4-part) in old people.	Aim: to investigate the feasibility and clinical potential of using AM models versus the conventional strategy (thin-layer CT scan) for the treatment of complicated proximal humeral fractures in old people.	+ Reduced surgery duration, less blood loss during surgery, and lower number of fluoroscopy can be observed in the test group compared with the control group (P<0.05). The results showed no significant difference in time to union between the two groups (P>0.05).	⊞	⊞	⊟	⊞	⊞	⊞	⊞	⊞	⊞	⊞	⊞	⊞	⊞	⊟	⊟	⊞	⊟	⊞	⊞	⊞	⊞	⊞	⊞	?	⊞	⊞	⊞
Musculoskeletal	1	Zhang	2011a	Acetabular navigational template	S	22 (Test=11, Control=11)	Age: Test=48.6 ± 6.8 years, Control=49.3 ± 4.9 years Test=7 male, 4 female, Control=5 male, 6 female	Preliminary application of computer-assisted patient-specific acetabular navigational template for total hip arthroplasty in adult single development dysplasia of the hip	Aim: to compare a patient-specific AM acetabular navigational template for total hip arthroplasty (THA) to conventional THA in adult single development dysplasia of the hip.	+ The test group showed smaller differences from the predetermined angles than the control group (p<0.05).	⊞	⊞	⊟	⊞	⊞	⊞	⊞	⊟	⊞	⊞	?	?	⊞	⊟	⊟	⊞	⊟	⊞	⊞	⊞	⊞	⊞	⊞	?	⊞	⊞	⊞
Oral and Maxillofacial	2	Arisan	2013	Bone and mucosa supported guides for dental implants	S	54 (Mucosa-supported=12, Bone-supported=15, Control=27)	Age: 28-74 years (mean=52.5 years) 23 male, 31 female	Implant Positioning Errors in Freehand and Computer-Aided Placement Methods: A Single-Blind Clinical Comparative Study	Aim: to compare the dental implant positioning errors related to the use of freehand and computer-aided treatment methods.	+ Interproximal emergence, insufficient interimplant distance, and improper parallelism errors were higher in implants placed using the freehand method (p <0.0001, p <.00001, and p <0.001 respectively).	⊞	⊞	⊞	⊞	⊞	⊞	⊞	⊟	⊞	⊞	?	?	⊞	⊟	⊞	⊞	⊞	⊞	⊞	⊞	⊞	⊞	⊟	X	⊞	⊞	⊞
Oral and Maxillofacial	2	Azuma	2014	Mandible model	S	28 (Test=12, Control=16)	Age: Test=34-91, Control=55-82 18 male, 10 female	Mandibular reconstruction using plates prebent to fit rapid prototyping 3-dimensional printing models ameliorates contour deformity	Aim: To evaluate the clinical efficacy and esthetic outcome of using preoperatively manufactured reconstruction plates, produced using AM models, in mandibular reconstruction.	+ The mandibular symmetry in patients receiving the AM-model-based prebent plates was better than that in patients receiving conventional reconstructive surgery (p<0.05).	⊞	⊞	⊞	⊞	⊟	⊞	⊞	⊟	⊟	⊟	?	?	⊞	⊟	⊟	⊞	⊟	⊞	⊞	⊞	?	?	⊟	X	⊟	⊟	⊞
Oral and Maxillofacial	2	Brown	2015	Orthodontic bracket system	T	96 (G1: Test=31, G2: Direct bonded conventional=33, G3: Indirect bonded conventional=32)	Age: G1=13.42 years, G2=3.58 years, G3=13.42 years G1 (AM) =17 male, 15 female G2=16 male, 15 female G3=17 male, 16 female	Effectiveness and efficiency of a CAD/CAM orthodontic bracket system.	Aim to retrospectively compare: patients who were indirectly bonded with CAD/CAM self-ligating appliances with those direct bonded with conventional self-ligating appliances, and those indirectly bonded with conventional self-ligating appliances.	+ No statistically significant difference was found on ABO Cast-Radiograph Evaluation scores among the 3 treatment groups (p=0.13). The mean treatment times were different (p<0.05, G1: 13.8 ± 3.4 months, G2: 21.9 ± 5.0 months; G3: 16.9 ± 4.1 months.	⊞	⊞	⊞	⊞	⊞	⊞	⊞	⊟	X	⊞	⊞	⊞	⊞	⊟	⊞	⊞	X	⊞	⊞	⊞	?	?	⊟	X	⊞	X	⊞
Oral and Maxillofacial	2	Calvo-Guirado	2011	Composite powder molding for bone grafts	P	20 (Test=10, Control=10)	No information provided	Calculation of bone graft volume using 3D reconstruction system	Aim: to validate the use of AM bioreplicas for calculating bone volumes prior to planning bone regeneration surgery.	NS No differences were recorded for measurements of bone defect between patient bone and the biological models (p>0.05) but there were also no differences between the test group and control group arising from post-surgical complications observed at the post-operatory fifteen and thirty day examinations.	⊞	⊞	⊟	⊞	⊟	⊞	⊞	⊟	⊞	⊟	?	?	⊞	⊟	⊟	⊞	⊞	⊞	⊞	⊞	⊞	?	?	?	?	⊞	⊟
Oral and Maxillofacial	2	Hanasono	2012	Guide for mandibular reconstruction	S	76 (Test=38, Control=38 defect-matched)	Age:Test=51.0 ± 14.4 years, Control=56.4 ± 15.1 years Test=26 male, 12 female Control=28 male, 10 female	Computer-Assisted Design and Rapid Prototype Modeling in Microvascular Mandible Reconstruction	Aim: to evaluate the use of AM modeling to improve the speed and accuracy of mandibular reconstruction.	+ The operative time for the test group was faster than the operative time defect-matched control group (p=0.0006). The change in position of selected bony landmarks was less in the test group than in the control group (p=0.001).	⊞	⊞	⊟	⊞	⊞	⊞	⊞	⊟	⊞	⊞	?	?	⊞	⊟	⊟	⊞	⊟	⊞	⊞	⊞	⊟	⊞	⊟	X	⊟	⊞	⊞
Oral and Maxillofacial	2	Inokoshi	2012	AM complete dentures	T	10 (Same participants used for test and control)	Age: 78.1 ± 5.6 years	Evaluation of a complete denture trial method applying rapid prototyping	Aim: to compare an AM method for complete dentures with the conventional method.	NS Prosthotontists rated esthetics and stability lower for the AM method (p<0.05), but chair time as faster (p<0.01). No significant differences were found between the two methods in regards to patient scores.	⊞	⊞	⊞	⊞	⊟	⊞	⊞	⊟	X	⊟	?	?	?	⊟	⊟	⊞	X	⊞	⊞	⊟	X	X	⊟	X	⊟	X	⊞
Oral and Maxillofacial	2	Kozakiewicz	2011	Model for developing patient-specific orbital wall implants	P	24 (Test=12, Control=12)	Age: Test=36 ± 10 years, Control=40 ± 20 years Test=9 male, 3 female, Control=9 male, 3 female	Treatment with individual orbital wall implants in humans - 1-Year ophthalmologic evaluation	Aim: to compare the functional results, 1 year after treatment, of using an AM reconstruction method for orbital wall repair with a conventional reconstruction method.	+ At 1 year follow up, the test group had better outcomes than the control group (diplopia: p=0.021, upgaze vertical visual disparity reduction: p=0.007)	⊞	⊞	⊞	⊞	⊟	⊞	⊟	⊟	⊞	⊞	⊞	⊞	⊞	⊟	⊟	⊞	⊞	⊞	⊞	⊞	⊞	⊟	⊟	X	⊟	⊞	⊞
Oral and Maxillofacial	2	Lee	2009	AM prefabricated prostheses for cranioplasty	T	131 (Autograft (G1)=91, conventional prosthesis (G2)=23, AM prefabricated prosthesis (G3)=17)	Age: G1=43.84 ± 17.91 years, G2=40.96 ± 15.48 years, G3=40.24 ± 11.97 years G1=62 male, 29 female, G2=14 male, 9 female, G3=12 male, 5 female	Cranioplasty using polymethyl methacrylate prostheses	Aim: to assess whether prefabricated AM prostheses outperform intra-operatively moulded prostheses and perform comparably to the gold-standard of autograft bone flaps in reducing operating time, blood loss and surgical complications in elective delayed cranioplasty operations, after decompressive craniectomy, to repair large (> 100 cm2) cranial defects.	+ G3 patients required less operating time than G2 patients (p<0.001), and operating time did not differ significantly between G1 and G3 (p>0.05). Intra-operative blood loss was lower for G3 than G2 (p=0.015) and did not differ significantly between G1 and G3 (p>0.05). The infection rate for G3 was lower than for G2 and was comparable to G1.	⊞	⊞	⊞	⊞	⊟	⊞	⊞	⊟	⊟	⊞	⊞	⊞	⊞	⊞	⊞	⊞	⊟	⊞	⊞	⊞	⊞	⊞	?	⊟	?	?	⊞
Oral and Maxillofacial	2	Modabber	2012a	Guide for jaw reconstruction	S	10 (Test=5, Control=5)	Age: 37-74 years (mean=52.4 years) 8 male, 2 female	Evaluation of computer-assisted jaw reconstruction with free vascularized fibular flap compared to conventional surgery: a clinical pilot study	Aim: to assess whether the AM method has an influence on the duration of the surgery and the amount of bone removed in fibular reconstruction of maxillary or mandibular defects.	+ Shaping procedure and ischaemic time was shorter for the test group than the control (p=0.014). There was no change between the defect size of the fibula and the necessary transplant size for the test group, but a mean change of 1.92cm occured for the control (p=0.001).	⊞	⊞	⊟	⊞	⊟	⊞	⊞	⊞	X	⊞	?	?	⊞	⊟	⊟	⊞	X	⊞	⊞	⊞	⊞	⊞	⊞	?	⊟	X	⊞
Oral and Maxillofacial	2	Resnick	2016	Surgical splint for orthognathic surgery	T	43 - All had Le Fort I osteotomy and bilateral sagittal split osteotomy (19 in G1: facial symmetry, single-segment, 17 in G2: facial asymmetry, single-segment, and 7 in G3: multi-segment).	All groups compared AM to manual splint fabrication. No information provided on age or gender.	Is There a Difference in Cost Between Standard and Virtual Surgical Planning for Orthognathic Surgery?	Aim: to retrospectively compare time and cost differences between virtual surgical planning and 3D printed orthognathic splints with conventional planning and manufacture techniques.	+ The average times and costs were 194 ± 14.1 minutes and $2,765.94, respectively, for VSP and 540.9 ± 99.5 minutes and $3,519.18, respectively, for standard planning. All time and cost differences were statistically significant (p<0.001).	⊞	⊞	⊞	⊞	⊟	⊞	⊞	⊟	X	⊞	?	?	⊞	⊟	⊟	X	X	⊞	⊞	⊞	⊟	⊟	?	X	⊟	X	⊞
Oral and Maxillofacial	2	Rogers-Vizena	2016	Craniofacial model	P	29 (Test=9, Control=20)	Age: Test=11.4 ± 3.0 years, Control=11.1 ± 4.7 years Test=4 male, 5 female Control=11 male, 9 female	Cost-Benefit Analysis of Three-Dimensional Craniofacial Models for Midfacial Distraction: A Pilot Study.	Aim: to retrospectively assess the benefit of AM models used in planning surgeries in regards to safety, operative time and cost.	NS 7 complications occurred in 6 of the control group, whereas none occurred in the test group. The test group had a 31.3 minute (7.8%) reduction in operative time (not significant), and time-based cost savings were $1036.	⊞	⊞	⊟	⊞	⊟	⊞	⊞	⊞	X	⊞	?	?	⊞	⊟	⊟	X	X	⊞	⊞	⊞	?	?	⊟	X	⊟	X	⊟
Oral and Maxillofacial	2	Sumida	2015	membranes for bone augmentation in implant treatment	T	26 (Test=13, Control=13)	Age: Test=48.6 ± 9.4 years, Control= 47.8 ± 8.8 years Test=4 male, 9 female, Control=3 male, 10 female	Custom-made titanium devices as membranes for bone augmentation in implant treatment: Clinical application and the comparison with conventional titanium mesh	Aim: to compare titanium AM devices with conventional methods for reconstructing alveolar bone for implantation.	+ There was no significant difference between groups in occurance of mucosal rupture (p=0.27). However, the operation time was shorter for the test group (p<0.01), and fewer screws were used to retain the membrane than the control group (p<0.01).	⊞	⊞	⊟	⊟	⊞	⊞	⊞	⊟	X	⊞	?	?	?	⊟	⊟	?	X	⊞	⊞	⊞	?	?	?	X	⊟	X	⊞
Oral and Maxillofacial	2	Zhang	2011b	Titanium plate to guide temporomandibular joint reconstruction	S	35 (Test=11, Control=24)	Age: Test=14-65 years (mean=42.3 years), Control=24-56 years (mean=34.9) Test=5 male, 6 female, Control=14 male, 10 female	Application of Rapid Prototyping for Temporomandibular Joint Reconstruction	Aim: to compare the operative time between temporomandibular joint reconstruction surgery using AM guides and conventional surgery.	+ Operative time was shorter for the test group than the control (p<0.05). The outcome of the surgeries was good, with the condyle-incisor, condyle–mental foramen, and condyle-angle from the postoperative CT scan showing no significant difference between the 2 sides (p>0.05).	⊟	⊞	⊟	⊞	⊟	⊟	⊟	⊟	⊞	⊟	⊞	⊞	⊞	⊟	⊟	⊞	?	⊞	⊞	⊞	⊟	⊟	⊟	X	⊟	⊞	⊞
Musculoskeletal	2	Hirao	2014	Guide for total ankle arthroplasty	S	38 (Test=24, Control=14)	No information provided	Use of a Custom-made Surgical Guide in Total Ankle Arthroplasty in Rheumatoid Arthritis Cases	Aim: to retrospectively compare the accuracy and stability of bone cutting for total ankle arthroplasty when using AM surgical guides to using conventional guides.	+ The error angle of the tibial bone cut was smaller (p = 0.02), and the tilting angle between components was smaller in the test group than the control group (P = 0.01).	⊟	⊞	⊟	⊞	⊟	⊞	⊞	⊞	⊞	⊞	?	?	⊞	⊟	⊟	?	⊞	⊞	⊞	⊞	?	?	⊟	X	⊟	⊞	⊞
Musculoskeletal	2	Iannotti	2015	Template for glenoid implant positioning	P	46 (Test=25, Control=21)	No information provided	Three-Dimensional Imaging and Templating Improve Glenoid Implant Positioning	Aim: to assess whether 3D imaging and the use of patient-specific instrumentation results in more accurate placement of the glenoid component than 3D imaging and standard instrumentation.	NS There was no significant difference between the test group and the control group in achieving the desired implant position, but both performed better than a retrospective sample that used 2D imaging with standard instrumentation.	⊞	⊞	⊟	⊞	⊟	⊞	⊞	⊟	X	⊞	?	?	⊞	⊟	⊞	⊞	X	⊞	⊞	⊞	⊟	⊟	⊟	?	⊟	X	⊞
Musculoskeletal	2	Karlin	2016	Spine model	P	17 (Test=7, Control =10)	Age: Test = 5-14 years (mean=10) Control = 11-18 years (mean=13) Test = 3 male, 4 female Control = 4 male, 6 female	The surgical treatment of spinal deformity in children with myelomeningocele: the role of personalized three-dimensional printed models.	Aim: to retrospectively review children for whom AM spine models were used for surgical planning (Test) and compare them through subjective and objective criteria to a similar group that had no models (Control).	NS The models improved planning and performance of patient-specific spinal instrumentation that was secure and low profile. The efficiency of the surgery as measured by intraoperative fluoroscopy time and blood loss and the extent of the deformity correction was comparable or superior in the test group.	⊞	⊞	⊟	⊟	⊟	⊞	⊟	⊟	X	⊟	?	?	⊞	⊟	⊟	?	X	⊞	⊞	⊞	⊟	?	⊟	X	⊟	X	⊟
Musculoskeletal	2	Li	2015a	Lumbar vertebral model	P	37 (Test=15, Control=22)	Age: Test=52.36 ± 4.57 years, Control= 50.83 ± 3.60 years Test=9 male, 6 female, Control= 15 male, 7 female	Application of the polystyrene model made by 3-D printing rapid prototyping technology for operation planning in revision lumbar discectomy	Aim: to evaluate the effectiveness of 3-D rapid prototyping technology in revision lumbar discectomy.	+ The test group had a shorter operation time (p<0.001) and less blood loss (p<0.001) than the control group. There was no difference between groups for complication rate or clinical metrics.	⊞	⊞	⊞	⊞	⊞	⊞	⊞	⊞	⊞	⊞	?	?	⊞	⊟	⊟	⊞	⊟	⊞	⊞	⊞	?	⊞	⊟	X	⊞	⊞	⊞
Musculoskeletal	2	Li	2016a	Model of pelvis and pelvic fracture	P	157 (Test=81, Control=76)	Age: Test=34.0 ± 8.2 years, Control = 32.2 ± 9.4 years Test=55 male, 26 female, Control=51 male, 25 female	Comparative use of the computer-aided angiography and rapid prototyping technology versus conventional imaging in the management of the Tile C pelvic fractures	Aim to compare the two groups on length of surgical procedure, intra-operative complications, intra- and postoperative blood loss, postoperative pain, postoperative nausea and vomiting (PONV), length of stay, and type of discharge.	+ The test group experienced shorter surgery time (p=0.035), less blood loss (31.0±8.2 g/L vs. 36.2±7.4 g/L), less pain (2.5±2.3 NRS score vs. 2.8±2.0 NRS score), and shorter recoveries (7.8±2.0 days vs. 10.2±3.1 days before discharge).	⊞	⊞	⊞	⊞	⊞	⊞	⊞	⊞	X	⊞	⊞	⊞	⊞	⊟	⊟	X	X	⊞	⊞	⊞	⊞	⊞	⊟	X	⊞	X	⊞
Musculoskeletal	2	Schweizer	2016	Guide for Reconstructions of Scaphoid Fractures	S	22 (Test=9, Control=13)	Age: Test = 15-47 years (mean=27) Control=14-41 years (mean=24) All male	Computer-Assisted 3-Dimensional Reconstructions of Scaphoid Fractures and Nonunions With and Without the Use of Patient-Specific Guides: Early Clinical Outcomes and Postoperative Assessments of Reconstruction Accuracy.	Aim: to assess the accuracy of the reduction of surgically reconstructed scaphoid nonunions or fractures using 3-dimensional computer-based planning with and without patient-specific guides.	+ In the test group, average residual displacement was 7˚ after surgery, and 8 of 9 scaphoids healed after 2 to 6 months, with partial nonunion after 9 months observed in one patient. In the control group, residual displacement after surgery was 26˚, and 11 of 13 scaphoids healed between 2 and 34 months. 2 scaphoids in the control did not consolidate. The difference in the accuracy of reconstruction was significant (p=0.001). Average operation time was shorter (p=0.01) for the test group.	⊞	⊞	⊟	⊞	⊟	⊞	⊞	⊞	⊞	⊞	?	?	⊞	⊟	⊟	⊞	⊟	⊞	⊞	⊞	?	?	⊟	X	⊟	⊞	⊞
Musculoskeletal	2	Yang	2014	Upper cervical spine model and navigation template	S	49 (Test=21, Control=28)	Age: 5-81 years (mean=37.6 years) 21 male, 28 female	Clinical Application of Computer-aided Design-Rapid Prototyping in C1–C2 Operation Techniques for Complex Atlantoaxial Instability	Aim: to evaluate the clinical efficacy of AM models in surgical treatments for atlantoaxial instability.	+ The test group had lower intraoperative fluoroscopy frequency (p<0.001), operation time (posterior p=0.001, anterior p=0.004) and blood loss for the posterior approach (p=0.006) than the control group. The screw malposition rate showed no difference between the two groups.	⊞	⊞	⊞	⊞	⊞	⊞	⊞	⊞	⊞	⊞	⊞	⊞	⊞	⊟	⊟	⊞	⊞	⊞	⊞	⊞	?	?	⊟	X	⊟	⊞	⊞
Musculoskeletal	2	Yang	2015	Spine model	P	126 (Test-50, Control=76)	Age: Test=12.52 ± 5.45 years, Control=12.11 ± 6.42 years Test=12 male, 38 female, Control=33 male, 43 female	Application of 3D Rapid Prototyping Technology in Posterior Corrective Surgery for Lenke 1 Adolescent Idiopathic Scoliosis Patients	Aim: to retrospectively evaluate the effectiveness of AM technology in corrective surgery for Lenke 1 adolescent idiopathic scoliosis (AIS) patients based on operation time, perioperative blood loss, transfusion volume, postoperative hemoglobin (Hb), postoperative complications, and length of hospital stay.	+ The test group had shorter operation time, less blood loss and transfusion volume, and higher postoperative Hb (all, P<0.001). However, no significant differences were observed in complication rate, length of hospital stay, and postoperative radiological outcomes between 2 groups (all, P>0.05).	⊞	⊞	⊞	⊞	⊞	⊞	⊞	⊞	⊞	⊞	?	?	⊞	⊟	⊟	⊞	?	⊞	⊞	⊞	?	⊞	⊟	X	⊟	⊞	⊞
Circulatory	3	Schievano	2007	Model of the right ventricular outflow tract and pulmonary trunk	P	12 (4 did not undergo percutaneous pulmonary valve implantation (PPVI), 4 successfully underwent PPVI, and 4 unsuccessfully underwent PPVI)	Age: 9-39 years (mean 20 years) 7 male, 5 female	Percutaneous Pulmonary Valve Implantation Based on Rapid Prototyping of Right Ventricular Outflow Tract and Pulmonary Trunk from MR Data	Aim: To retrospectively determine if magnetic resonance imaging data can be used to create rigid models that are accurate representations of the right ventricular outflow tract and pulmonary trunk anatomy and if such models can be used to refine the selection of patients for PPVI.	NS Both cardiologists correctly determined from the models that the 4 subjects who did not undergo PPVI were unsuitable for PPVI. Of the 8 subjects who underwent PPVI, only 4 were successful. Both cardiologists correctly identified the suitability of 5 of these subjects from the models. Therefore the cardiologists had a 75% accuracy when selecting patient suitability for PPVI using 3D models.	⊞	⊞	⊟	⊞	⊟	⊞	⊞	⊟	X	⊟	⊟	⊟	⊞	⊟	⊞	X	X	⊞	⊞	⊞	?	?	⊟	⊟	⊟	X	⊟
Oral and Maxillofacial	3	Adolphs	2014	Splint for orthognathic surgery	T	10 (Same participants used for test and control)	No information provided	RapidSplint: virtual splint generation for orthognathic surgery – results of a pilot series	Aim: to demonstrate that clinically usable splints for orthognathic surgery can be produced by computer-assisted technology.	NS 8 of 10 AM splints, and 6 out of conventional splints could be used clinically to achieve and maintain final occlusion after orthognathic surgery. In the five patients where both types of splints were available, their occlusal fitting was assessed as being equivalent, and in one case the AM splint showed better occlusal fitting than the traditional splint.	⊞	⊞	⊟	⊞	X	⊟	⊟	⊟	X	⊟	?	?	⊞	⊟	⊟	?	X	⊞	⊞	?	X	X	X	X	X	X	⊟
Oral and Maxillofacial	3	Arisan	2010	Dental screw placement guides	S	54 (28 guides in system I, 32 guides in system II)	Age: 28-73 years (mean=48.4 years) 16 male, 38 female	Accuracy of Two Stereolithographic Guide Systems for Computer-Aided Implant Placement: A Computed Tomography-Based Clinical Comparative Study	Aim: To compare the accuracy of two AM guide systems for dental implant placement.	+ Implants that were placed by bone-supported guides had higher deviations than implants that were placed by mucosa-supported guides (p<0.05) but there was no difference in deviation between implant brands (p <0.08).	⊞	⊞	⊞	⊞	⊞	⊞	⊞	⊟	⊟	⊞	?	?	⊞	⊟	⊟	⊞	⊟	⊞	⊞	⊞	⊞	?	⊟	X	⊟	⊞	⊞
Oral and Maxillofacial	3	Morris	2013	Mandible or midface model	P	20 (Test=10, Control=10)	Age: Test=40.5 ± 5.6 years, Control=66.1 ± 3.0 years Test=6 male, 4 female, Control=8 male, 2 female	Utility of Stereolithographic Models in Osteocutaneous Free Flap Reconstruction of the Head and Neck	Aim: to assess whether am AM model decreased the operative time enough to offset the cost of manufacturing the model.	- No significant differences were observed for total operative time between groups, but the total reconstructive time was lower in the control group (p=0.05).	⊞	⊞	⊟	⊞	⊞	⊞	⊞	⊟	X	⊞	?	?	⊞	⊟	⊟	X	X	⊞	⊞	⊞	?	?	⊟	X	⊟	X	⊞
Oral and Maxillofacial	3	Shahbazian	2013	Surgical guide for autotransplantation of teeth	S	40 (Test=20, Control=20)	Age: Test=9-18 years (mean=11 years), Control=9-18 years (mean=12 years) Test=12 male, 8 female, Control=13 male, 7 female	Validation of the cone beam computed tomography-based stereolithographic surgical guide aiding autotransplantation of teeth: clinical case-control study	Aim: to compare the outcome of AM surgical planning and transfer technique for tooth autotransplantation to conventional autotransplantation.	NS 6 from the control group showed 1 or more complications, compared to 2 from the test group.	⊞	⊞	⊟	⊞	⊞	⊞	⊟	⊞	⊟	⊟	?	?	⊞	⊟	⊞	⊞	⊟	⊞	⊞	⊞	⊟	⊟	⊟	X	⊟	?	⊟
Oral and Maxillofacial	3	Weitz	2010	Templates for navigated dental implantation	S	10 (Same participants used for test and control)	Age: 19-46 years (mean=29.9 years) 6 male, 4 female	Accuracy of templates for navigated implantation made by rapid prototyping with DICOM datasets of cone beam computer tomography (CBCT)	Aim: to compare the accuracy of AM surgical templates with templates made from conventional impressions for placing implants.	NS The conventional templates enabled excellent fitting accuracy but the accuracy of implants fitted with the AM templates was not sufficient and could result in significant iatrogenic complications.	⊞	⊟	⊟	⊟	⊟	⊞	⊞	⊞	X	⊟	?	?	?	⊟	⊟	?	X	⊞	⊞	⊞	X	X	X	X	X	X	⊟
Musculoskeletal	3	Otsuki	2013	Cutting guide for curved peri-acetabular osteotomy	S	18 (Test=7, Control=11)	Age: No information provided Test=All 7 female, Control= No information provided	Developing a novel custom cutting guide for curved peri-acetabular osteotomy	Aim: to evaluate the efficacy of this new method and cutting guide.	NS The rate of osteotomy-related complications did not differ significantly between the two groups (p>0.1). The actual cutting line corresponded almost exactly to the planned cutting line in all patients.	⊞	⊞	⊟	⊞	⊟	⊟	⊞	⊞	⊞	⊟	?	?	⊞	⊟	⊟	⊞	⊟	⊞	⊞	⊞	⊟	?	⊟	X	⊟	⊞	⊟

⊞ Yes
⊟ No
? Unable to determine
X Not applicable

Medical sector	Level of evidence	First author	Date	Category	No. of participants	Title
Neoplasms	4	Huang	2012	S	31	A digital model individual template and CT-guided 125I seed implants for malignant tumors of the head and neck
Neoplasms	4	Silberstein	2014	P	5	Physical Models of Renal Malignancies Using Standard Cross-sectional Imaging and 3-Dimensional Printers: A Pilot Study
Neoplasms	4	Tao	2010	P	8	Application of computer-aided technique in pelvic precise model preparation and individualized prosthesis implantation
Neoplasms	4	von Rundstedt	2016	P	10	Utility of patient-specific silicone renal models for planning and rehearsal of complex tumour resections prior to robot-assisted laparoscopic partial nephrectomy.
Neoplasms	4	Xiao	2016	P	5	En Bloc Resection of Primary Malignant Bone Tumor in the Cervical Spine Based on 3-Dimensional Printing Technology.
Neoplasms	4	Zhang	2016a	P	10	Evaluation of three-dimensional printing for laparoscopic partial nephrectomy of renal tumors: a preliminary report
Nervous	4	Konrad	2010	S	263	Customized, Miniature Rapid-Prototype Stereotactic Frames for Use in Deep Brain Stimulator Surgery: Initial Clinical Methodology and Experience from 263 Patients from 2002 to 2008
Circulatory	4	Farooqi	2016	P	6	Application of Virtual Three-Dimensional Models for Simultaneous Visualization of Intracardiac Anatomic Relationships in Double Outlet Right Ventricle.
Circulatory	4	Gilon	2002	P	35	Effect of Three-Dimensional Valve Shape on the Hemodynamics of Aortic Stenosis: Three-Dimensional Echocardiographic Stereolithography and Patient Studies
Circulatory	4	Jacobs	2008	P	3	3D-Imaging of cardiac structures using 3D heart models for planning in heart surgery: a preliminary study
Circulatory	4	Liu	2016b	P	8	The Value of 3D Printing Models of Left Atrial Appendage Using Real-Time 3D Transesophageal Echocardiographic Data in Left Atrial Appendage Occlusion: Applications toward an Era of Truly Personalized Medicine.
Circulatory	4	Ngan	2006	P	4	The rapid prototyping of anatomic models in pulmonary atresia
Circulatory	4	Ripley	2016	P	16	3D printing based on cardiac CT assists anatomic visualization prior to transcatheter aortic valve replacement.
Circulatory	4	Weinstock	2015	P	4	Optimizing cerebrovascular surgical and endovascular procedures in children via personalized 3D printing
Digestive	4	Ko	2016	S	35	Novel 3D-printing technique for caps to enable tailored therapeutic endoscopy
Digestive	4	Zein	2013	P	6	Three-Dimensional Print of a Liver for Preoperative Planning in Living Donor Liver Transplantation
Oral and Maxillofacial	4	Asaumi	2001	P	2	Comparison of three-dimensional computed tomography with rapid prototype models in the management of coronoid hyperplasia
Oral and Maxillofacial	4	Barakat	2013	S	6	Clinical and radiographic evaluation of a computer-generated guiding device in bilateral sagittal split osteotomies
Oral and Maxillofacial	4	Bosc	2017	S	18	Mandibular reconstruction after cancer: an in-house approach to manufacturing cutting guides
Oral and Maxillofacial	4	Cassetta	2013	T	20	Accuracy of Two Stereolithographic Surgical Templates: A Retrospective Study
Oral and Maxillofacial	4	Chai	2015	P	7	Treating Parry-Romberg Syndrome Using Three-Dimensional Scanning and Printing and the Anterolateral Thigh Dermal Adipofascial Flap
Oral and Maxillofacial	4	Chan	2015	S	10	3D Rapid Prototyping for Otolaryngology—Head and Neck Surgery: Applications in Image-Guidance, Surgical Simulation and Patient-Specific Modeling
Oral and Maxillofacial	4	Chen	2014	P	6	Three-Dimensional Preoperative Design of Distraction Osteogenesis for Hemifacial Microsomia
Oral and Maxillofacial	4	Chen	2010	S	4	Modular Preoperative Planning Software for Computer-Aided Oral Implantology and the Application of a Novel Stereolithographic Template: A Pilot Study
Oral and Maxillofacial	4	Ciprandi	2012	T	5	Calcium Phosphate Cement in Orbital Reconstructions
Oral and Maxillofacial	4	Clelland	2016	S	18	Split-mouth comparison of splinted and nonsplinted prostheses on short implants: 3-year results
Oral and Maxillofacial	4	Cornelius	2016	S	12	Iterations of computer- and template assisted mandibular or maxillary reconstruction with free flaps containing the lateral scapular border-Evolution of a biplanar plug-on cutting guide.
Oral and Maxillofacial	4	Cui	2014	P	3	Surgical Planning, Three-Dimensional Model Surgery and Preshaped Implants in Treatment of Bilateral Craniomaxillofacial Post-Traumatic Deformities
Oral and Maxillofacial	4	Curcio	2007	P	14	Use of models in surgical predictability of oral rehabilitations
Oral and Maxillofacial	4	Dérand	2012	S	4	Imaging, Virtual Planning, Design, and Production of Patient-Specific Implants and Clinical Validation in Craniomaxillofacial Surgery
Oral and Maxillofacial	4	D'haese	2012	S	13	A Prospective Study on the Accuracy of Mucosally Supported Stereolithographic Surgical Guides in Fully Edentulous Maxillae
Oral and Maxillofacial	4	Di Giacomo	2011	S	12	Accuracy and Complications of Computer-Designed Selective Laser Sintering Surgical Guides for Flapless Dental Implant Placement and Immediate Definitive Prosthesis Installation
Oral and Maxillofacial	4	Di Giacomo	2005	S	4	Clinical Application of Stereolithographic Surgical Guides for Implant Placement: Preliminary Results
Oral and Maxillofacial	4	Di Giacomo	2016	S	9	A selective laser sintering prototype guide used to fabricate immediate interim fixed complete arch prostheses in flapless dental implant surgery: Technique description and clinical results.
Oral and Maxillofacial	4	El-Gengehi	2015	S	24	Evaluation of the Accuracy of Computer-Guided Mandibular Fracture Reduction
Oral and Maxillofacial	4	Ersoy	2008	S	21	Reliability of implant placement with stereolithographic surgical guides generated from computed tomography: clinical data from 94 implants
Oral and Maxillofacial	4	Fan	2007	P	17	Late Reconstruction of the Complex Orbital Fractures With Computer-Aided Design and Computer-Aided Manufacturing Technique
Oral and Maxillofacial	4	Feng	2011	S	4	Mirror imaging and preshaped titanium plates in the treatment of unilateral malar and zygomatic arch fractures
Oral and Maxillofacial	4	Fiaschi	2016	T	12	Surgical results of cranioplasty with a polymethylmethacrylate customized cranial implant in pediatric patients: a single-center experience.
Oral and Maxillofacial	4	Gaggl	1998	P	3	Treatment planning for sinus lift augmentations through use of 3-dimensional milled models derived from computed tomography scans
Oral and Maxillofacial	4	Guo	2009	T	61	Reconstruction of Orbital Floor Fractures
Oral and Maxillofacial	4	Huang	2017	P	97	Personalized Reconstruction of Traumatic Orbital Defects Based on Precise Three-Dimensional Orientation and Measurements of the Globe.
Oral and Maxillofacial	4	Jiang	2015	T	18	Functional Evaluation of a CAD/CAM Prosthesis for Immediate Defect Repair after Total Maxillectomy: A Case Series of 18 Patients with Maxillary Sinus Cancer
Oral and Maxillofacial	4	Jiao	2014	T	11	Rehabilitation of maxillectomy defects with obturator prostheses fabricated using computer-aided design and rapid prototyping: a pilot study.
Oral and Maxillofacial	4	Kim	2004	P	182	Evaluation of the prognosis and causes of failure in 182 cases of autogenous tooth transplantation
Oral and Maxillofacial	4	Kocyiğit	2013	S	10	Evaluation of Preoperative Model Surgery and the Use of a Maxillary Sinus Surgical Template in Sinus Floor Augmentation Surgery
Oral and Maxillofacial	4	Komiyama	2012	S	29	Soft Tissue Conditions and Marginal Bone Changes around Immediately Loaded Implants Inserted in Edentate Jaws Following Computer Guided Treatment Planning and Flapless Surgery: A 31-Year Clinical Follow-Up
Oral and Maxillofacial	4	Kozakiewicz	2009	P	6	Clinical application of 3D pre-bent titanium implants for orbital floor fractures
Oral and Maxillofacial	4	Labadie	2008	S	4	Clinical Validation of Percutaneous Cochlear Implant Surgery: Initial Report
Oral and Maxillofacial	4	Lethaus	2010	T	11	Surgical and prosthetic reconsiderations in patients with maxillectomy
Oral and Maxillofacial	4	Li	2013a	T	6	Digital Design and Individually Fabricated Titanium Implants for the Reconstruction of Traumatic Zygomatico-Orbital Defects
Oral and Maxillofacial	4	Li	2015b	T	12	Clinical Feasibility and Efficacy of Using Virtual Surgical Planning in Bimaxillary Orthognathic Surgery Without Intermediate Splint
Oral and Maxillofacial	4	Li	2013b	S	6	A novel method of computer aided orthognathic surgery using individual CAD/CAM templates: a combination of osteotomy and repositioning guides
Oral and Maxillofacial	4	Li	2016c	S	7	Treatment of Dentofacial Deformities Secondary to Osteochondroma of the Mandibular Condyle Using Virtual Surgical Planning and 3-Dimensional Printed Surgical Templates
Oral and Maxillofacial	4	Li	2016b	S	9	A new design of CAD/CAM surgical template system for two-piece narrowing genioplasty.
Oral and Maxillofacial	4	Lin	2016	S	5	Mandibular angle split osteotomy based on a novel augmented reality navigation using specialized robot-assisted arms--A feasibility study.
Oral and Maxillofacial	4	Lieger	2010	P	29	Computer-Assisted Design and Manufacture of Implants in the Late Reconstruction of Extensive Orbital Fractures
Oral and Maxillofacial	4	Liu	2010	S	11	Accuracy of surgical positioning of orthodontic miniscrews with a computer-aided design and manufacturing template
Oral and Maxillofacial	4	Liu	2013	S	8	Digital Surgical Templates for Managing High-Energy Zygomaticomaxillary Complex Injuries Associated With Orbital Volume Change: A Quantitative Assessment
Oral and Maxillofacial	4	Liu	2009	S	7	Applying Computer Techniques in Maxillofacial Reconstruction Using a Fibula Flap: A Messenger and an Evaluation Method
Oral and Maxillofacial	4	Liu	2014a	S	15	Technical procedures for template-guided surgery for mandibular reconstruction based on digital design and manufacturing
Oral and Maxillofacial	4	Martorelli	2012	S	6	A comparison between customized clear and removable orthodontic appliances manufactured using RP and CNC techniques
Oral and Maxillofacial	4	Mazzoni	2015	S	10	Computer-Aided Design and Computer-Aided Manufacturing Cutting Guides and Customized Titanium Plates Are Useful in Upper Maxilla Waferless Repositioning
Oral and Maxillofacial	4	Modabber	2012a	S	15	Computer-assisted Mandibular Reconstruction with Vascularized Iliac Crest Bone Graft
Oral and Maxillofacial	4	Morea	2011	S	4	Precise Insertion of Orthodontic Miniscrews with a Stereolithographic Surgical Guide Based on Cone Beam Computed Tomography Data: A Pilot Study
Oral and Maxillofacial	4	Muller	2003	P	52	The Application of Rapid Prototyping Techniques in Cranial Reconstruction and Preoperative Planning in Neurosurgery
Oral and Maxillofacial	4	Murray	2008	P	3	Advanced technology in the management of fibrous dysplasia
Oral and Maxillofacial	4	Ozan	2011	S	54	Correlation Between Bone Density and Angular Deviation of Implants Placed Using CT-Generated Surgical Guides
Oral and Maxillofacial	4	Ozan	2009	S	30	Clinical Accuracy of 3 Different Types of Computed Tomography-Derived Stereolithographic Surgical Guides in Implant Placement
Oral and Maxillofacial	4	Papaspyridakos	2012a	T	14	Computer-assisted design/computer-assisted manufacturing zirconia implant fixed complete prostheses: clinical results and technical complications up to 4 years of function
Oral and Maxillofacial	4	Park	2015	P	127	Mirror-Imaged Rapid Prototype Skull Model and Pre-Molded Synthetic Scaffold to Achieve Optimal Orbital Cavity Reconstruction
Oral and Maxillofacial	4	Park	2016	T	21	Cranioplasty Enhanced by Three-Dimensional Printing: Custom-Made Three-Dimensional-Printed Titanium Implants for Skull Defects.
Oral and Maxillofacial	4	Pettersson	2010	S	25	Accuracy of Virtually Planned and Template Guided Implant Surgery on Edentate Patientscid_285 527..537
Oral and Maxillofacial	4	Prisman	2014	P	10	Value of preoperative mandibular plating in reconstruction of the mandible
Oral and Maxillofacial	4	Reiser	2015	S	17	V-Stand—A Versatile Surgical Platform for Oromandibular Reconstruction Using a 3-Dimensional Virtual Modeling System
Oral and Maxillofacial	4	Rotaru	2012	T	10	Cranioplasty With Custom-Made Implants: Analyzing the Cases of 10 Patients
Oral and Maxillofacial	4	Sailer	1998	P	20	The value of stereolithographic models for preoperative diagnosis of craniofacial deformities and planning of surgical corrections
Oral and Maxillofacial	4	Schantz	2006	T	5	Cranioplasty after trephination using a novel biodegradable burr hole cover: technical case report
Oral and Maxillofacial	4	Scolozzi	2009	P	10	Accuracy and Predictability in Use of AO Three-Dimensionally Preformed Titanium Mesh Plates for Posttraumatic Orbital Reconstruction: A Pilot Study
Oral and Maxillofacial	4	Shan	2015	T	2	Surgical Reconstruction of Maxillary and Mandibular Defects Using a Printed Titanium Mesh
Oral and Maxillofacial	4	Shehab	2013	S	6	A novel design of a computer-generated splint for vertical repositioning of the maxilla after Le Fort I osteotomy
Oral and Maxillofacial	4	Shen	2015	P	17	Presurgical Nasoalveolar Molding for Cleft Lip and Palate: The Application of Digitally Designed Molds
Oral and Maxillofacial	4	Shu	2014	S	8	Accuracy of using computer-aided rapid prototyping templates for mandible reconstruction with an iliac crest graft
Oral and Maxillofacial	4	Staffa	2006	S	26	Custom made cranioplasty prostheses in porous hydroxy-apatite using 3D design techniques: 7 years experience in 25 patients
Oral and Maxillofacial	4	Stubinger	2014	S	10	Deviations between Placed and Planned Implant Positions: An Accuracy Pilot Study of Skeletally Supported Stereolithographic Surgical Templates
Oral and Maxillofacial	4	Sun	2013a	S	15	Accuracy of Upper Jaw Positioning With Intermediate Splint Fabrication After Virtual Planning in Bimaxillary Orthognathic Surgery
Oral and Maxillofacial	4	Sun	2013b	S	6	Error analysis of a CAD/CAM method for unidirectional mandibular distraction osteogenesis in the treatment of hemifacial microsomia
Oral and Maxillofacial	4	Suomalainen	2015	P	102	Rapid prototyping modelling in oral and maxillofacial surgery: A two year retrospective study.
Oral and Maxillofacial	4	Tabaković	2015	T	10	Application of Computer-Aided Designing and Rapid Prototyping Technologies in Reconstruction of Blowout Fractures of the Orbital Floor
Oral and Maxillofacial	4	Tang	2010	P	46	Individual Design and Rapid Prototyping in Reconstruction of Orbital Wall Defects
Oral and Maxillofacial	4	Turgut	2012	T	11	Computer-Aided Design and Manufacture and Rapid Prototyped Polymethylmethacrylate Reconstruction
Oral and Maxillofacial	4	Valente	2007	S	25	Accuracy and clinical outcomes of computer aided oral implant surgery: a preliminary study on 25 patients
Oral and Maxillofacial	4	Van Assche	2010	S	8	Accuracy assessment of computer-assisted flapless implant placement in partial edentulism
Oral and Maxillofacial	4	Verhamme	2012	S	5	A clinically relevant validation method for implant placement after virtual planning
Oral and Maxillofacial	4	Vrielinck	2003	S	12	Image-based planning and clinical validation of zygoma and pterygoid implant placement in patients with severe bone atrophy using customized drill guides. Preliminary results from a prospective clinical follow-up study
Oral and Maxillofacial	4	Wang	2013a	P	10	Three-dimensional virtual technology in reconstruction of mandibular defect including condyle using double-barrel vascularized fibula flap
Oral and Maxillofacial	4	Wiggins	2013	S	113	Cranioplasty With Custom-Made Titanium Plates—14 Years Experience
Oral and Maxillofacial	4	Won	2013	P	21	Improving pre-operative planning for complex total hip replacement with a Rapid Prototype model enabling surgical simulation
Oral and Maxillofacial	4	Wong	2005	S	13	Comparison of 2 Methods of Making Surgical Models for Correction of Facial Asymmetry
Oral and Maxillofacial	4	Xu	2015a	S	24	Combined Use of Rapid-Prototyping Model and Surgical Guide in Correction of Mandibular Asymmetry Malformation Patients With Normal Occlusal Relationship
Oral and Maxillofacial	4	Yin	2014	S	36	Mandibular Distraction Combined With Orthognathic Techniques for the Correction of Severe Adult Mandibular Hypoplasia
Oral and Maxillofacial	4	Ying	2015	S	14	Correction of facial asymmetry associated with vertical maxillary excess and mandibular prognathism by combined orthognathic surgery and guiding templates and splints fabricated by rapid prototyping technique
Oral and Maxillofacial	4	Yu	2013	P	15	CAD presurgical nasoalveolar molding effects on the maxillary morphology in infants with UCLP
Oral and Maxillofacial	4	Yuan	2013	T	10	Accuracy evaluation of a new three-dimensional reproduction method of edentulous dental casts, and wax occlusion rims with jaw relation
Oral and Maxillofacial	4	Zhang	2015	T	12	Computer-Aided Design and Computer-Aided Manufacturing Hydroxyapatite/Epoxide Acrylate Maleic Compound Construction for Craniomaxillofacial Bone Defects
Oral and Maxillofacial	4	Zhang	2009	S	6	Application of navigation template to fixation of sacral fracture using three-dimensional reconstruction and reverse engineering technique
Oral and Maxillofacial	4	Zhang	2016b	P	30	Accuracy of virtual surgical planning in two-jaw orthognathic surgery: comparison of planned and actual results
Oral and Maxillofacial	4	Zheng	2012	T	9	Mandible reconstruction assisted by preoperative virtual surgical simulation
Oral and Maxillofacial	4	Zhong	2015	T	10	Quantitative Analysis of Dual-Purpose, Patient- Specific Craniofacial Implants for Correction of Temporal Deformity
Oral and Maxillofacial	4	Zinser	2012	S	8	Computer-assisted orthognathic surgery: feasibility study using multiple CAD/CAM surgical splints
Oral and Maxillofacial	4	Zou	2015	S	6	Application of Computer Techniques in Correcting Mild Zygomatic Assymetry With Unilateral Reduction Malarplasty
Musculoskeletal	4	Bauer	2015	P	19	Preoperative Computer Simulation and Patient-specific Guides are Safe and Effective to Correct Forearm Deformity in Children
Musculoskeletal	4	Chen	2016a	S	5	A novel navigation template for fixation of acetabular posterior column fractures with antegrade lag screws: design and application
Musculoskeletal	4	Dai	2007	T	10	Computer-Aided Custom-Made Hemipelvic Prosthesis Used in Extensive Pelvic Lesions
Musculoskeletal	4	Deng	2016	S	10	The accuracy and the safety of individualized 3D printing screws insertion templates for cervical screw insertion.
Musculoskeletal	4	Dhanshyani	2011	P	6	Rapid prototyping models for dysplastic hip surgeries in Malaysia
Musculoskeletal	4	Guarino	2007	P	13	Rapid Prototyping Technology for Surgeries of the Pediatric Spine and Pelvis
Musculoskeletal	4	Hananouchi	2009	S	24	Tailor-made surgical guide based on rapid prototyping technique for cup insertion in total hip arthroplasty
Musculoskeletal	4	Hsieh	2010	P	12	Repositioning Osteotomy for Intra-Articular Malunion of Distal Radius With Radiocarpal and/or Distal Radioulnar Joint Subluxation
Musculoskeletal	4	Hu	2016	S	151	A comparative study on the accuracy of pedicle screw placement assisted by personalized rapid prototyping template between pre- and post-operation in patients with relatively normal mid-upper thoracic spine.
Musculoskeletal	4	Jiang	2016	S	32	Accuracy assessment of atlantoaxial pedicle screws assisted by a novel drill guide template.
Musculoskeletal	4	Kaneyama	2015	S	20	Safe and Accurate Midcervical Pedicle Screw Insertion Procedure With the Patient-Specifi c Screw Guide Template System
Musculoskeletal	4	Kawaguchi	2012	S	11	Development of a New Technique for Pedicle Screw and Magerl Screw Insertion Using a 3-Dimensional Image Guide
Musculoskeletal	4	Kunz	2010	P	45	Computer-Assisted Hip Resurfacing Using Individualized Drill Templates
Musculoskeletal	4	Kunz	2011	P	80	Registration stability of physical tem-plates in hip surgery
Musculoskeletal	4	Li	2013c	P	25	Revision of Complex Acetabular Defects Using Cages with the Aid of Rapid Prototyping.
Musculoskeletal	4	Li	2015c	T	26	Custom Acetabular Cages Offer Stable Fixation and Improved Hip Scores for Revision THA With Severe Bone Defects
Musculoskeletal	4	Lin	2013	P	10	Application of three-dimensional model in the management of irreducible atlanto-axial dislocation
Musculoskeletal	4	Liu	2016a	S	10	Preliminary application of a multi-level 3D printing drill guide template for pedicle screw placement in severe and rigid scoliosis.
Musculoskeletal	4	Lu	2009a	S	25	A Novel Patient-Specific Navigational Template for Cervical Pedicle Screw Placement
Musculoskeletal	4	Lu	2012	S	16	Accuracy and efficacy of thoracic pedicle screws in scoliosis with patient-specific drill template
Musculoskeletal	4	Mao	2010	P	16	Clinical application of computer-designed polystyrene models in complex severe spinal deformities: a pilot study
Musculoskeletal	4	Miyake	2012	S	20	Computer-Assisted Corrective Osteotomy for Malunited Diaphyseal Forearm Fractures
Musculoskeletal	4	Murase	2005	P	7	Does Three-dimensional Computer Simulation Improve Results of Scaphoid Nonunion Surgery?
Musculoskeletal	4	Murase	2008	S	22	Three-Dimensional Corrective Osteotomy of Malunited Fractures of the Upper Extremity with Use of a Computer Simulation System
Musculoskeletal	4	Parchi	2013	P	8	Computer Tomography Prototyping and Virtual Procedure Simulation in Difficult Cases of Hip Replacement Surgery
Musculoskeletal	4	Putzier	2014	S	4	A New Navigational Tool for Pedicle Screw Placement in Patients with Severe Scoliosis: A Pilot Study to Prove Feasibility, Accuracy, and Identify Operative Challenges.
Musculoskeletal	4	Schweizer	2013	S	6	Three-Dimensional Correction of Distal Radius Intra-Articular Malunions Using Patient-Specific Drill Guides
Musculoskeletal	4	Takemoto	2016	S	36	Additive-manufactured patient-specific titanium templates for thoracic pedicle screw placement: novel design with reduced contact area.
Musculoskeletal	4	Takeyasu	2013	S	30	Preoperative, Computer Simulation-Based, Three-Dimensional Corrective Osteotomy for Cubitus Varus Deformity with Use of a Custom-Designed Surgical Device
Musculoskeletal	4	Tricot	2012	S	3	3D-corrective osteotomy using surgical guides for posttraumatic distal humeral deformity
Musculoskeletal	4	Yang	2011	S	21	Personalised modified osteotomy using computer-aided design–rapid prototyping to correct thoracic deformities
Musculoskeletal	4	Zeng	2015	P	38	Evaluation of three-dimensional printing for internal fixation of unstable pelvic fracture from minimal invasive para-rectus abdominis approach: a preliminary report
Musculoskeletal	4	Zhang	2011c	S	18	Application of computer-aided design osteotomy template for treatment of cubitus varus deformity in teenagers: A pilot study
Musculoskeletal	4	Zhuang	2015	P	14	Surgical Treatment of Acetabulum Top Compression Fracture with Sea Gull Sign
Musculoskeletal	4	Hurson	2007	P	20	Rapid prototyping in the assessment, classification and preoperative planning of acetabular fractures
Musculoskeletal	4	Kunz	2012	S	31	Hip resurfacing with individualized drill templates— comparison between anterolateral and posterior approach
Musculoskeletal	4	Xu	2015b	P	10	Application of Rapid Prototyping Pelvic Model for Patients with DDH to Facilitate Arthroplasty Planning: A Pilot Study.
Musculoskeletal	4	Zeng	2016	P	10	A combination of three-dimensional printing and computer-assisted virtual surgical procedure for preoperative planning of acetabular fracture reduction.
External Devices	4	Fabry	2002	T	43	Hearing aid physical fit: The next revolution?
External Devices	4	Gibson	2014	T	15	Functionally Optimized Orthoses for Early Rheumatoid Arthritis Foot Disease: A Study of Mechanisms and Patient Experience
External Devices	4	Liu	2014b	T	10	Newly Designed Foot Orthosis for Children with Residual Clubfoot After Ponseti Casting
External Devices	4	Pallari	2010	T	7	Mass Customization of Foot Orthoses for Rheumatoid Arthritis Using Selective Laser Sintering
External Devices	4	Raux	2014	T	30	Tridimensional trunk surface acquisition for brace manufacturing in idiopathic scoliosis
External Devices	4	Telfer	2013a	T	24	Dose-response effects of customised foot orthoses on lower limb kinematics and kinetics in pronated foot type
External Devices	4	Telfer	2013b	T	12	Dose-response effects of customised foot orthoses on lower limb muscle activity and plantar pressures in pronated foot type
Neoplasms	5	Akiba	2015	P	1	A Three-Dimensional Mediastinal Model Created with Rapid Prototyping in a Patient with Ectopic Thymoma
Neoplasms	5	D'Urso	1999b	P	1	Biomodel guided Stereotaxy
Neoplasms	5	Krauel	2016	P	3	Use of 3D Prototypes for Complex Surgical Oncologic Cases
Neoplasms	5	Mainenti	2009	P	1	Ameloblastic fibro-odontosarcoma: a case report
Neoplasms	5	Wong	2015	T	1	One-step reconstruction with a 3D-printed, biomechanically evaluated custom implant after complex pelvic tumor resection
Neoplasms	5	Wiebe	2015	S	1	Customized vaginal vault brachytherapy with computed tomography imaging-derived applicator prototyping
Nervous	5	D'Urso	1999c	P	16	Cerebrovascular biomodelling: a technical note
Circulatory	5	Bosi	2015	P	1	Patient-Specific Finite Element Models to Support Clinical Decisions: A Lesson Learnt from a Case Study of Percutaneous Pulmonary Valve Implantation
Circulatory	5	Dankowski	2014	P	1	3D heart model printing for preparation of percutaneous structural interventions: description of the technology and case report
Circulatory	5	Ejaz	2014	S	3	Color-coded patient-specific physical models of congenital heart disease
Circulatory	5	Erbano	2013	P	3	Rapid prototyping of three-dimensional biomodels as an adjuvant in the surgical planning for intracranial aneurysms
Circulatory	5	Gosnell	2016	P	1	Integration of Computed Tomography and Three-Dimensional Echocardiography for Hybrid Three-Dimensional Printing in Congenital Heart Disease.
Circulatory	5	Hermsen	2017	P	2	Scan, plan, print, practice, perform: Development and use of a patient-specific 3-dimensional printed model in adult cardiac surgery.
Circulatory	5	Kiraly	2016	P	1	Three-dimensional printed prototypes refine the anatomy of post-modified Norwood-1 complex aortic arch obstruction and allow presurgical simulation of the repair.
Circulatory	5	Lan	2016	P	1	Development of Three-Dimensional Printed Craniocerebral Models for Simulated Neurosurgery.
Circulatory	5	Mottle-link	2008	P	1	Physical Models Aiding in Complex Congenital Heart Surgery
Circulatory	5	Pepper	2013	S	34	Implantation of an individually computer-designed and manufactured external support for the Marfan aortic root
Circulatory	5	Riesenkampff	2009	P	11	The practical clinical value of three-dimensional models of complex congenitally malformed hearts
Circulatory	5	Sodian	2009	P	1	3-Dimensional Printing of Models to Create Custom-Made Devices for Coil Embolization of an Anastomotic Leak After Aortic Arch Replacement
Circulatory	5	Sardari Nia	2016	P	1	Preoperative planning with three-dimensional reconstruction of patient's anatomy, rapid prototyping and simulation for endoscopic mitral valve repair.
Circulatory	5	Shimamura	2012	P	4	Three-Dimensional Replica of a Life-Sized Model of Aortic Arch Aneurysm for Preoperative Assessments
Circulatory	5	Tam	2013	P	1	3D Printing of an Aortic Aneurysm to Facilitate Decision Making and Device Selection for Endovascular Aneurysm Repair in Complex Neck Anatomy
Circulatory	5	Vranicar	2008	P	12	The use of stereolithographic hand held models for evaluation of congenital anomalies of the great arteries
Circulatory	5	Wurm	2004	P	13	Cerebrovascular stereolithographic biomodeling for aneurysm surgery
Respiratory	5	Booysen	2006	S	1	Anaesthetic mouthpiece development through QFD and customer interaction with functional prototypes
Skin	5	Chae	2015	P	1	3D-Printed haptic “Reverse” models for preoperative planning in soft tissue reconstruction: A case report
Skin	5	Wei	2016	T	2	3D-printed transparent facemasks in the treatment of facial hypertrophic scars of young children with burns.
Oral and Maxillofacial	5	Abe	1998	P	7	Model-based surgical planning and simulation of cranial base surgery
Oral and Maxillofacial	5	Abou-ElFetouh	2011	S	2	Computer-guided rapid-prototyped templates for segmental mandibular osteotomies: a preliminary report
Oral and Maxillofacial	5	Adolphs	2013	P	2	Craniomaxillofacial surgery planning based on 3D models derived from Cone-Beam CT data
Oral and Maxillofacial	5	Aita-Holmes	2015	T	1	Digital capture, design, and manufacturing of an extraoral device for a clarinet player with Bell’s palsy
Oral and Maxillofacial	5	Aitasalo	2014	T	12	Craniofacial bone reconstruction with bioactive fiber-reinforced composite implant
Oral and Maxillofacial	5	Bai	2012	T	7	An Alternative Approach for Mandible Reconstruction
Oral and Maxillofacial	5	Bai	2010	S	1	CAD/CAM surface templates as an alternative to the intermediate wafer in orthognathic surgery
Oral and Maxillofacial	5	Bellini	2007	P	1	Bi-directional distraction in the treatment of micro-orbitism: A case report
Oral and Maxillofacial	5	Bibb	2006	T	1	Trial fitting of a removable partial denture framework made using computer-aided design and rapid prototyping techniques
Oral and Maxillofacial	5	Brie	2012	T	8	A new custom made bioceramic implant for the repair of large and complex craniofacial bone defects
Oral and Maxillofacial	5	Byun	2015	S	1	Endodontic Treatment of an Anomalous Anterior Tooth with the Aid of a 3-dimensional Printed Physical Tooth Model
Oral and Maxillofacial	5	Cao	2010	T	39	Application of EH Compound Artificial Bone Material Combined With Computerized Three-Dimensional Reconstruction in Craniomaxillofacial Surgery
Oral and Maxillofacial	5	Cassetta	2016	S	1	Accelerating orthodontic tooth movement: A new, minimally-invasive corticotomy technique using a 3D-printed surgical template.
Oral and Maxillofacial	5	Cesarano	2005	T	1	Customization of Load-Bearing Hydroxyapatite Lattice Scaffolds
Oral and Maxillofacial	5	Cho	2013	T	1	Mirror-image anterior crown fabrication with computer-aided design and rapid prototyping technology: A clinical report
Oral and Maxillofacial	5	Chow	2006	S	5	Zygomatic Implants—Protocol for Immediate Occlusal Loading: A Preliminary Report
Oral and Maxillofacial	5	Chua	1998	P	1	Rapid Prototyping Assisted Surgery Planning
Oral and Maxillofacial	5	Ciocca	2010a	T	1	Immediate facial rehabilitation in cancer patients using CAD–CAM and rapid prototyping technology: a pilot study
Oral and Maxillofacial	5	Ciocca	2010b	S	1	Rehabilitation of the nose using CAD-CAM and rapid prototyping technology after ablative surgery of squamous cell carcinoma: A pilot clinical report
Oral and Maxillofacial	5	Ciocca	2012	S	1	CAD/CAM guided secondary mandibular reconstruction of a discontinuity defect after ablative cancer surgery
Oral and Maxillofacial	5	Da Rosa	2004	P	1	Rapid Prototyping in Maxillofacial Surgery and Traumatology: Case Report
Oral and Maxillofacial	5	Elbashti	2016	T	1	The Role of Digitization in the Rapid Reproduction of an Obturator in a Frail Elderly Patient.
Oral and Maxillofacial	5	Fang	2013	S	2	Complex Facial Deformity Reconstruction With a Surgical Guide Incorporating a Built-in Occlusal Stent as the Positioning Reference
Oral and Maxillofacial	5	Feng	2010	P	1	Mirror imaging and preshaped titanium plates in the treatment of unilateral malar and zygomatic arch fractures
Oral and Maxillofacial	5	Fernandes	2007	P	1	Computer-aided surgery using 3D rendering of maxillofacial pathology and trauma
Oral and Maxillofacial	5	Figliuzzi	2012	T	1	A novel root analogue dental implant using CT scan and CAD/CAM: selective laser melting technology
Oral and Maxillofacial	5	Flugge	2013	S	1	Three-Dimensional Plotting and Printing of an Implant Drilling Guide: Simplifying Guided Implant Surgery
Oral and Maxillofacial	5	Goh	2009	T	31	Customised fabricated implants after previous failed cranioplasty
Oral and Maxillofacial	5	Gong	2012	P	7	Correction of maxillary deformity in infants with bilateral cleft lip and palate using computer-assisted design
Oral and Maxillofacial	5	Gopakumar	2004	T	1	RP in medicine: a case study in cranial reconstructive surgery
Oral and Maxillofacial	5	Hatamleh	2013a	T	1	Construction of an Implant-Retained Auricular Prosthesis with the Aid of Contemporary Digital Technologies: A Clinical Report
Oral and Maxillofacial	5	Hatamleh	2013b	T	4	Management of Extensive Frontal Cranioplasty Defects
Oral and Maxillofacial	5	He	2015	T	7	Novel Arch Bar Fabricated With a Computer-Aided Design and Three-Dimensional Printing: A Feasibility Study
Oral and Maxillofacial	5	Herlin	2011	S	1	Rapid prototyping in craniofacial surgery: Using a positioning guide after zygomatic osteotomy e A case report
Oral and Maxillofacial	5	Hierl	2013	S	3	CAD-CAM–Assisted Esthetic Facial Surgery
Oral and Maxillofacial	5	Holck	1999	P	2	Benefits of Stereolithography in Orbital Reconstruction
Oral and Maxillofacial	5	Hou	2011	P	7	Immediate Reconstruction of Bilateral Mandible Defects: Management Based on Computer-Aided Design/Computer-Aided Manufacturing Rapid Prototyping Technology in Combination With Vascularized Fibular Osteomyocutaneous Flap
Oral and Maxillofacial	5	Hu	2007	P	1	Reconstruction of a palatomaxillary defect with vascularized iliac bone combined with a superficial inferior epigastric artery flap and zygomatic implants as anchorage
Oral and Maxillofacial	5	Jang	2013	P	4	Autotransplantation of immature third molars using a computer-aided rapid prototyping model: a report of 4 cases
Oral and Maxillofacial	5	Juergens	2012	S	1	A Computer-Assisted Diagnostic and Treatment Concept to Increase Accuracy and Safety in the Extracranial Correction of Cranial Vault Asymmetries
Oral and Maxillofacial	5	Keightley	2010	S	1	Autotransplantation of an immature premolar, with the aid of cone beam CT and computeraided prototyping: a case report
Oral and Maxillofacial	5	Kfir	2013	P	1	The diagnosis and conservative treatment of a complex type 3 dens invaginatus using cone beam computed tomography (CBCT) and 3D plastic models
Oral and Maxillofacial	5	Kim	2007	S	1	Surgical positioning of orthodontic miniimplants with guides fabricated on models replicated with cone-beam computed tomography
Oral and Maxillofacial	5	Kim	2008	S	1	Clinical application of a stereolithographic surgical guide for simple positioning of orthodontic mini-implants
Oral and Maxillofacial	5	Kübler	1995	P	21	Repair of human skull defects using osteoinductive bone alloimplants
Oral and Maxillofacial	5	Kusek	2009	P	1	The use of laser technology (Er;Cr:YSGG) and stereolithography to aid in the placement of a subperiosteal implant: case study
Oral and Maxillofacial	5	Lee	2015	S	1	Keyhole System: A Computer-Assisted Designed and Computer-Assisted Manufactured Maxillomandibular Complex Repositioner in Orthognathic Surgery
Oral and Maxillofacial	5	Li	2009	P	1	Application of computer-aided three-dimensional skull model with rapid prototyping technique in repair of zygomatico-orbito-maxillary complex fracture
Oral and Maxillofacial	5	Li	2012	P	1	Preliminary Application of Virtual Simulation and Reposition Template for Zygomatico-orbitomaxillary Complex Fracture
Oral and Maxillofacial	5	Liu	2014c	S	1	Additive manufacturing and digital design assisted precise apicoectomy: a case study
Oral and Maxillofacial	5	Lucey	2010	P	1	Case report: Cone-beam CT imaging in the management of a double tooth
Oral and Maxillofacial	5	Matsuo	2010	T	2	Clinical application of a custom-made bioresorbable raw particulate hydroxyapatite/poly-L-lactide mesh tray for mandibular reconstruction
Oral and Maxillofacial	5	Mavili	2007	P	3	Use of Three-Dimensional Medical Modeling Methods for Precise Planning of Orthognathic Surgery
Oral and Maxillofacial	5	Mendez	2015	P	2	Customized ‘‘In-Office’’ Three-Dimensional Printing for Virtual Surgical Planning in Craniofacial Surgery
Oral and Maxillofacial	5	Oliveira	2010	P	1	Recessive dystrophic epidermolysis bullosa—oral rehabilitation using stereolithography and immediate endosseous implants
Oral and Maxillofacial	5	Olszewski	2010	P	1	Innovative procedure for computer-assisted genioplasty: three-dimensional cephalometry, rapid-prototyping model and surgical splint
Oral and Maxillofacial	5	Ozan	2012	S	1	Clinical Application of Stereolithographic Surgical Guide With a Handpiece Guidance Apparatus: A Case Report
Oral and Maxillofacial	5	Papaspyridakos	2015	S	1	Full Mouth Implant Rehabilitation with Staged Approach: 6-Year Clinical Follow-Up
Oral and Maxillofacial	5	Papaspyridakos	2010	S	1	Immediate Loading of the Maxilla with Prefabricated Interim Prosthesis Using Interactive Planning Software, and CAD/CAM Rehabilitation with Definitive Zirconia Prosthesis: 2-Year Clinical Follow-up
Oral and Maxillofacial	5	Papaspyridakos	2012b	S	1	Flapless CAD/CAM-guided surgery for staged transition from failing dentition to complete arch implant rehabilitation: a 3-year clinical report
Oral and Maxillofacial	5	Papaspyridakos	2008	S	1	Complete arch implant rehabilitation using subtractive rapid prototyping and porcelain fused to zirconia prosthesis: A clinical report
Oral and Maxillofacial	5	Park	2012	P	1	Autotransplantation with Simultaneous Sinus Floor Elevation
Oral and Maxillofacial	5	Park	2014	P	1	Optimizing Third Molar Autotransplantation: Applications of Reverse-Engineered Surgical Templates and Rapid Prototyping of Three-Dimensional Teeth
Oral and Maxillofacial	5	Rohner	2013	P	1	Importance of patient-specific intraoperative guides in complex maxillofacial reconstruction
Oral and Maxillofacial	5	Salmi	2013	T	1	A digital process for additive manufacturing of occlusal splints: a clinical pilot study
Oral and Maxillofacial	5	Sammartino	2004	S	1	Stereolithography in Oral Implantology A Comparison of Surgical Guides
Oral and Maxillofacial	5	Seitz	2016	T	1	A Combination of Various Technologies in the Fabrication of a Removable Partial Denture--A Case Study.
Oral and Maxillofacial	5	Shengwei	2014	T	7	Combined Use of an Anterolateral Thigh Flap and Rapid Prototype Modeling to Reconstruct Maxillary Oncologic Resections and Midface Defects
Oral and Maxillofacial	5	Shin	2015	S	1	A case report of a surgical guide fabricated via intraoral scanning-based implant planning and wax-based rapid prototyping
Oral and Maxillofacial	5	Singare	2007	P	2	Individually Prefabricated Prosthesis for Maxilla Reconstruction
Oral and Maxillofacial	5	Sultan	2011	S	3	Custom-Made, 3D, Intraoperative Surgical Guides for Nasal Reconstruction
Oral and Maxillofacial	5	Suzuki	2005	P	2	Application of a rapid-prototyped temporal bone model for surgical planning
Oral and Maxillofacial	5	Thomas	2013	S	4	Use of a titanium cutting guide to assist with raising and inset of a DCIA free flap
Oral and Maxillofacial	5	Toso	2005	P	16	Usefulness of Computed Tomography in pre-surgical evaluation of maxillo-facial pathology with rapid prototyping and surgical pre-planning by virtual reality
Oral and Maxillofacial	5	Turgut	2009	T	10	Use of Rapid Prototyping in Prosthetic Auricular Restoration
Oral and Maxillofacial	5	Verweij	2016	P	1	Autotransplantation of Premolars With a 3-Dimensional Printed Titanium Replica of the Donor Tooth Functioning as a Surgical Guide: Proof of Concept.
Oral and Maxillofacial	5	Watson	2014	T	1	Correction of Facial and Mandibular Asymmetry Using a Computer Aided Design/ Computer Aided Manufacturing Prefabricated Titanium Implant
Oral and Maxillofacial	5	Wiechmann	2008	T	1	Herbst appliance in lingual orthodontics
Oral and Maxillofacial	5	Wang	2013b	S	5	Application of Computer Techniques in Repair of Oblique Facial Clefts With Outer-Table Calvarial Bone Grafts
Oral and Maxillofacial	5	Wang	2015	P	1	Complex reconstruction of facial deformity and function after severe gunshot injury: one case report
Oral and Maxillofacial	5	Yoshioka	2010	T	1	Fabrication of an Orbital Prosthesis Using a Non-contact Three-Dimensional Digitizer and Rapid-Prototyping System
Oral and Maxillofacial	5	Zhen-yu	2010	P	1	Computer-assisted design and rapid prototyping technology for the underlay of hemifacial atrophy
Oral and Maxillofacial	5	Zhou	2010	T	6	Accurate Reconstruction of Discontinuous Mandible Using a Reverse Engineering/ Computer-Aided Design/Rapid Prototyping Technique: A Preliminary Clinical Study
Oral and Maxillofacial	5	Zhu	2011	P	15	Registration Strategy Using Occlusal Splint Based on Augmented Reality for Mandibular Angle Oblique Split Osteotomy
Musculoskeletal	5	Ai	2011	P	2	Transoral Atlantoaxial Reduction Plate Internal Fixation with Transoral Transpedicular or Articular Mass Screw of C2 for the Treatment of Irreducible Atlantoaxial Dislocation
Musculoskeletal	5	Bellanova	2013	S	4	Surgical Guides (Patient-Specific Instruments) for Pediatric Tibial Bone Sarcoma Resection and Allograft Reconstruction
Musculoskeletal	5	Brown	2002	S	8	Application of Computer-Generated Stereolithography and Interpositioning Template in Acetabular Fractures: A Report of Eight Cases
Musculoskeletal	5	Chen	2016b	T	1	Image-guided installation of 3D-printed patient-specific implant and its application in pelvic tumor resection and reconstruction surgery.
Musculoskeletal	5	D'Urso	1999d	P	5	Spinal biomodeling
Musculoskeletal	5	D'Urso	2005	P	20	Biomodeling as an Aid to Spinal Instrumentation
Musculoskeletal	5	Fan	2015	P	3	Implantation of customized 3-D printed titanium prosthesis in limb salvage surgery: a case series and review of the literature.
Musculoskeletal	5	Fang	2012	P	1	Guiding Templates For Pedicle Screws Insertion: A preliminary Study
Musculoskeletal	5	Hsu	2015	P	1	Patient-Specific 3-Dimensional Printed Titanium Truss Cage With Tibiotalocalcaneal Arthrodesis for Salvage of Persistent Distal Tibia Nonunion
Musculoskeletal	5	Hung	2008	P	1	Clinical Application of Rapid Prototype Model in Pediatric Proximal Femoral Corrective Osteotomy
Musculoskeletal	5	Li	2014	P	2	Composite artificial semi-knee joint system
Musculoskeletal	5	Lu	2009b	P	9	A novel computer-assisted drill guide template for placement of C2 laminar screws
Musculoskeletal	5	Minns	2003	P	1	The use of a reconstructed three-dimensional solid model from CT to aid the surgical management of a total knee arthroplasty: a case study
Musculoskeletal	5	Otsuki	2016	P	3	Utility of a custom screw insertion guide and a full-scale, color-coded 3D plaster model for guiding safe surgical exposure and screw insertion during spine revision surgery.
Musculoskeletal	5	Paiva	2007	P	1	Application of the stereolithography technique in complex spine surgery
Musculoskeletal	5	Papandrea	2014	P	1	New perspectives on treatment of both bone forearm malunions: three-dimensional (3D) modeling and printing
Musculoskeletal	5	Rahmati	2012	P	1	An improved methodology for design of custom-made hip prostheses to be fabricated using additive manufacturing technologies
Musculoskeletal	5	Singhal	2016	P	2	Improved Surgery Planning Using 3-D Printing: a Case Study.
Musculoskeletal	5	Tan	2015	P	4	A Novel Preoperative Planning Technique Using a Combination of CT Angiography and Three-Dimensional Printing for Complex Toe-to-Hand Reconstruction.
Musculoskeletal	5	Toyoda	2013	P	1	Novel Approach for the Effi cient Use of a Full-Scale, 3-Dimensional Model for Cervical Posterior Fixation
Musculoskeletal	5	Tricot	2012	P	3	3D-corrective osteotomy using surgical guides for posttraumatic distal humeral deformity
Musculoskeletal	5	Truscott	2007	P	1	Using RP to promote collaborative design of customised medical implants
Musculoskeletal	5	Yamazaki	2006	P	1	Surgical Simulation of Instrumented Posterior Occipitocervical Fusion in a Child With Congenital Skeletal Anomaly
External Devices	5	Buehler	2016	T	1	Investigating the Implications of 3D Printing in Special Education
External Devices	5	Eggbeer	2011	T	1	Computer-aided methods in bespoke breast prosthesis design and fabrication
External Devices	5	Faustini	2005	T	1	Design and Analysis of Orthogonally Compliant Features for Local Contact Pressure Relief in Transtibial Prostheses
External Devices	5	Hsu	2010	T	1	The development of a rapid prototyping prosthetic socket coated with a resin layer for transtibial amputees
External Devices	5	Mavroidis	2011	T	1	Patient specific ankle-foot orthoses using rapid prototyping
External Devices	5	Qiao	2016	T	3	A novel combination of computer-assisted reduction technique and three dimensional printed patient-specific external fixator for treatment of tibial fractures
External Devices	5	Rogers	2008	T	1	Case Report: Variably Compliant Transtibial Prosthetic Socket Fabricated Using Solid Freeform Fabrication
External Devices	5	Sengeh	2013	T	1	A Variable-Impedance Prosthetic Socket for a Transtibial Amputee Designed from Magnetic Resonance Imaging Data
External Devices	5	South	2010	T	1	Manufacture of Energy Storage and Return Prosthetic Feet Using Selective Laser Sintering
External Devices	5	Tay	2002	T	1	A CASD-CASM method for prosthetic socket fabrication using the FDM technology
External Devices	5	Yoshikawa	2015	T	1	Rehand: Realistic Electric Prosthetic Hand Created with a 3D Printer

PRISMA 2009 Checklist

Section/topic	#	Checklist item	Reported on page #
**TITLE**			
Title	1	Identify the report as a systematic review, meta-analysis, or both.	1
**ABSTRACT**			
Structured summary	2	Provide a structured summary including, as applicable: background; objectives; data sources; study eligibility criteria, participants, and interventions; study appraisal and synthesis methods; results; limitations; conclusions and implications of key findings; systematic review registration number.	1
**INTRODUCTION**			
Rationale	3	Describe the rationale for the review in the context of what is already known.	2
Objectives	4	Provide an explicit statement of questions being addressed with reference to participants, interventions, comparisons, outcomes, and study design (PICOS).	2
**METHODS**			
Protocol and registration	5	Indicate if a review protocol exists, if and where it can be accessed (e.g., Web address), and, if available, provide registration information including registration number.	2
Eligibility criteria	6	Specify study characteristics (e.g., PICOS, length of follow-up) and report characteristics (e.g., years considered, language, publication status) used as criteria for eligibility, giving rationale.	3
Information sources	7	Describe all information sources (e.g., databases with dates of coverage, contact with study authors to identify additional studies) in the search and date last searched.	2
Search	8	Present full electronic search strategy for at least one database, including any limits used, such that it could be repeated.	Appendix1
Study selection	9	State the process for selecting studies (i.e., screening, eligibility, included in systematic review, and, if applicable, included in the meta-analysis).	3
Data collection process	10	Describe method of data extraction from reports (e.g., piloted forms, independently, in duplicate) and any processes for obtaining and confirming data from investigators.	3
Data items	11	List and define all variables for which data were sought (e.g., PICOS, funding sources) and any assumptions and simplifications made.	2
Risk of bias in individual studies	12	Describe methods used for assessing risk of bias of individual studies (including specification of whether this was done at the study or outcome level), and how this information is to be used in any data synthesis.	3
Summary measures	13	State the principal summary measures (e.g., risk ratio, difference in means).	3
Synthesis of results	14	Describe the methods of handling data and combining results of studies, if done, including measures of consistency (e.g., I^2) for each meta-analysis.	N/A

For peer review only - http://bmjopen.bmj.com/site/about/guidelines.xhtml

PRISMA 2009 Checklist

Section/topic	#	Checklist item	Reported on page #
Risk of bias across studies	15	Specify any assessment of risk of bias that may affect the cumulative evidence (e.g., publication bias, selective reporting within studies).	Table 3
Additional analyses	16	Describe methods of additional analyses (e.g., sensitivity or subgroup analyses, meta-regression), if done, indicating which were pre-specified.	N/A
**RESULTS**			
Study selection	17	Give numbers of studies screened, assessed for eligibility, and included in the review, with reasons for exclusions at each stage, ideally with a flow diagram.	Figure 1
Study characteristics	18	For each study, present characteristics for which data were extracted (e.g., study size, PICOS, follow-up period) and provide the citations.	Table 2
Risk of bias within studies	19	Present data on risk of bias of each study and, if available, any outcome level assessment (see item 12).	Table 3
Results of individual studies	20	For all outcomes considered (benefits or harms), present, for each study: (a) simple summary data for each intervention group (b) effect estimates and confidence intervals, ideally with a forest plot.	Table 2
Synthesis of results	21	Present results of each meta-analysis done, including confidence intervals and measures of consistency.	N/A
Risk of bias across studies	22	Present results of any assessment of risk of bias across studies (see Item 15).	N/A
Additional analysis	23	Give results of additional analyses, if done (e.g., sensitivity or subgroup analyses, meta-regression [see Item 16]).	N/A
**DISCUSSION**			
Summary of evidence	24	Summarize the main findings including the strength of evidence for each main outcome; consider their relevance to key groups (e.g., healthcare providers, users, and policy makers).	4
Limitations	25	Discuss limitations at study and outcome level (e.g., risk of bias), and at review-level (e.g., incomplete retrieval of identified research, reporting bias).	5
Conclusions	26	Provide a general interpretation of the results in the context of other evidence, and implications for future research.	5
**FUNDING**			
Funding	27	Describe sources of funding for the systematic review and other support (e.g., supply of data); role of funders for the systematic review.	5

From: Moher D, Liberati A, Tetzlaff J, Altman DG, The PRISMA Group (2009). Preferred Reporting Items for Systematic Reviews and Meta-Analyses: The PRISMA Statement. PLoS Med 6(6): e1000097. doi:10.1371/journal.pmed1000097

For more information, visit: www.prisma-statement.org.

For peer review only - http://bmjopen.bmj.com/site/about/guidelines.xhtml

BMJ Open

Clinical efficacy and effectiveness of 3D printing: A systematic review

Journal:	*BMJ Open*
Manuscript ID	bmjopen-2017-016891.R2
Article Type:	Research
Date Submitted by the Author:	19-Sep-2017
Complete List of Authors:	Diment, Laura; University of Oxford, Department of Engineering, Institute of Biomedical Engineering<br>Thompson, Mark; University of Oxford, Department of Engineering, Institute of Biomedical Engineering<br>Bergmann, Jeroen; University of Oxford, Department of Engineering, Institute of Biomedical Engineering
**Primary Subject Heading**:	Patient-centred medicine
Secondary Subject Heading:	Evidence based practice
Keywords:	Printing, Three-Dimensional, Health Care Evaluation Mechanisms, Medical devices, Personalised healthcare, Fabrication, Additive manufacturing

SCHOLARONE™
Manuscripts

For peer review only - http://bmjopen.bmj.com/site/about/guidelines.xhtml

Title page

Clinical efficacy and effectiveness of 3D printing: A systematic review

Corresponding Author:

Laura E. Diment
DPhil student
Department of Engineering Science
University of Oxford
Parks Road, Oxford, UK
OX1 3PJ
laura.diment@eng.ox.ac.uk
Ph. 01865 227374

Co-authors:

Mark S. Thompson
Director, Oxford Mechanobiology Group
Department of Engineering Science
University of Oxford
Parks Road, Oxford, UK
OX1 3PJ

Jeroen HM. Bergmann
Associate Director, Oxford Biodesign Programme
Department of Engineering Science
University of Oxford
Parks Road, Oxford, UK
OX1 3PJ

Word count: 3815

For peer review only

ABSTRACT

Objective

To evaluate the clinical efficacy and effectiveness of using 3D printing to develop medical devices across all medical fields.

Design

PRISMA-compliant systematic review

Data sources

PubMed, Web of Science, OVID, IEEE Xplore and Google Scholar.

Methods

A double-blinded review method was used to select all abstracts up to January 2017 that reported on clinical trials of a 3D printed medical device. The studies were ranked according to their level of evidence, divided into medical fields based on the International Classification of Diseases chapter divisions, and categorised into whether they were used for preoperative planning, aiding surgery or therapy. The Downs and Black Quality Index critical appraisal tool was used to assess the quality of reporting, external validity, risk of bias, risk of confounding and power of each study.

Results

Of the 3084 abstracts screened, 350 studies met the inclusion criteria. Oral and Maxillofacial Surgery contained 58.3% of studies, and 23.7% covered the Musculoskeletal System. Only 21 studies were randomised controlled trials (RCTs), and all fitted within these two fields. The majority of RCTs were 3D printed anatomical models for preoperative planning and guides for aiding surgery. The main benefits of these devices were decreased surgical operation times and increased surgical accuracy.

Conclusions

All medical fields that assessed 3D printed devices concluded that they were clinically effective. The fields that most rigorously assessed 3D printed devices were Oral and Maxillofacial Surgery and the Musculoskeletal System, both of which concluded that the 3D printed devices out-performed their conventional comparators. However, the efficacy and effectiveness of 3D printed devices remains undetermined for the majority of medical fields. 3D printed devices can play an important role in healthcare, but more rigorous and long-term assessments are needed to determine if 3D printed devices are clinically relevant before they become part of standard clinical practice.

For peer review

Strengths and limitations of the study

- The first use of a rigorous systematic literature review design applied to 3D printing for clinical uses.
- Validated quality assessment and clinical level of evidence tools are used to assess the progress made in different medical fields.
- The study is limited to a critical appraisal of individual studies, rather than a meta-analysis, because of the breadth of uses (from anatomical models and surgical guides to therapeutic devices) and the lack of comparable hypotheses.
- Due to the speed of innovation in the field, the review will need to be updated frequently.

For peer review only - http://bmjopen.bmj.com/site/about/guidelines.xhtml

INTRODUCTION

3D printing is likely to play a pivotal role in transforming healthcare and clinical practice because it provides the opportunity to create customised devices designed for the complexity and individual variances of the patient populations.[1]

Additive manufacturing, more commonly referred to as 3D printing, is an industrial production technique that enables a 3D digital model to be converted into a physical model by printing it layer by layer. For decades 3D printing has been used for rapid prototyping, but the recent advances in the available materials, speed, resolution, accuracy, reliability, cost and repeatability of 3D printing technologies have broadened the possibilities for clinical uses.[2,3] Many medical fields are already using 3D printing to manufacture custom surgical tools, guides, dose delivery devices, implants, external prosthetics or orthotics, and devices for preoperative planning or education.[2,4,5] Tailoring devices and procedures to the patient is expected to reduce the time required for surgery, treatment or recovery, while increasing the accuracy and success of the outcome.[1] The worldwide 3D printing industry's revenue from products and services is over $4 billion and fast-growing, with 13.1% of the industry attributed to the medical sector.[6]

In most medical fields, 3D printing applications are still in the research and development stage or have only just entered clinical practice within the last decade, and hence there has been a lack of research into the clinical efficacy, effectiveness and long-term follow-up in comparison to traditional technologies.[7] *Efficacy* refers to the performance of the device under ideal and controlled conditions, and *effectiveness* refers to its performance under typical clinical conditions.[8] The Food and Drug Administration (FDA) states that 3D printed medical devices are required to meet the same regulations as their non-3D printed counterparts. Because 3D printed devices can have different safety and efficacy issues than the equivalent devices, additional testing may be required to demonstrate safety, efficacy and effectiveness.[9] Hospitals and clinics will need to adopt new medical product procedures when they want to introduce 3D printing for healthcare, and more evidence of device efficacy and effectiveness will help make an informed discussion before prescribing 3D printed devices for patients. Providing a critical appraisal of the efficacy and effectiveness of 3D printed medical devices gives healthcare professionals a resource to assess the validity of the devices, and provides researchers with an overview of areas that require further research and validation.

Previous systematic reviews summarise 3D printed medical devices being used in specific medical fields, such as plastic and reconstructive surgery,[10] preoperative planning for liver resections,[11] and increasingly available 3D printing processes used in dentistry and mandibular reconstructions.[12–14] Systematic reviews on the advantages of 3D printed devices over conventional methods in surgery have found improved clinical outcomes, and reductions in operating times and manufacturing costs.[5,15] However, the reviews do not critically appraise the studies, and are therefore subject to bias. The only systematic review that did assess bias for a very specific field found no difference in patient outcomes when 3D printed instrumentation for total knee replacements was compared to conventional instrumentation.[16] This highlights the need for a systematic review that incorporates a critical appraisal of the studies that are included.

This review aims to assess the clinical efficacy and effectiveness of 3D printed devices through performing a systematic literature search, categorising reports of 3D printed device usage by medical field and purpose, and assessing the reports' scientific quality. The key findings of studies that compare 3D printed medical devices to their non-3D printed counterparts are presented, and the gaps in research highlighted.

METHODS

Search strategy

The applied protocol (not previously registered) used systematic methods to search for relevant studies, screen them for eligibility, and assess their quality. The review follows the Preferred Reporting Items for Systematic Reviews and Meta-Analyses (PRISMA) guidelines.[17]

A literature search was performed using PubMed, Web of Science, OVID, IEEE Xplore and Google Scholar. A combination of 45 relevant keywords were used to collect all studies that included a 3D printed device and a clinical trial. Due to the word and character limit search restrictions in IEEE Xplore and Google Scholar, a narrower search of 15 terms was performed for these databases. The search includes all publications up to January 2017. The complete search strategy is provided in the supplementary material. Mendeley was used as a reference manager.

For peer review only - http://bmjopen.bmj.com/site/about/guidelines.xhtml

Study selection

After removing all duplicates from the databases, the title and abstract of each publication was reviewed using a double-blinded method (undertaken by LD and JB) to determine eligibility for inclusion.

The inclusion criteria were:

i. **Relevance -** papers were required to report first-hand on the results of a clinical study that assessed the efficacy and effectiveness of a 3D printed device.
ii. **Language -** only papers written or translated into English were included in the review.
iii. **Peer-review -** records that had no peer-review or where the level of peer-review could not be traced were excluded from the review.

Where the results of the reviewers (LD and JB) conflicted, the reviewers discussed their reasoning for inclusion/exclusion, and where required for clarification of the paper's relevance, the paper's introduction and methods were assessed. Consensus on eligibility was achieved for all papers. Included articles underwent a full-text review and references cited in these studies were also examined for relevance using the same inclusion criteria.

Quality assessment

The studies were then rated according to their level of evidence, based on the Oxford Centre for Evidence-based Medicine (CEBM) Levels of Evidence,[18] as summarised in Table 1. Systematic reviews and opinion papers were excluded because they cannot provide the original data in a suitable format to be assessed using the quality assessment method used in this review. Where studies used 3D printed devices but the hypothesis, aim or objective was focused on an outcome other than the effect of the 3D printed device, the paper has been graded as the level of evidence shown for the device. All levels of evidence were included in the review but only those that used a control group (Levels 1 to 3) were critically analysed.

Table 1. CEBM Levels of Evidence

Level	Types of study
1	- Randomised controlled trials (RCTs)
2	- Cohort studies
3	- Case-control studies<br>- Poor quality estimates of data that include sensitivity analyses incorporating clinically sensible variations
4	- Case-series studies
5	- Case reports<br>- Studies that do not provide quantitative evidence to support a hypothesis

Studies were then placed in medical fields based on the International Classification of Diseases (ICD) chapter divisions[19] and categorised by the purpose of the device. The three categories were; devices used for preoperative planning, devices to aid surgery, and therapeutic devices.

To analyse the quality of the studies under review, critical appraisal tools were assessed to find one that met the following criteria:

- Suitable for assessing the quality of both randomised and non-randomised studies,

- Well-regarded and commonly used for quality assessments in systematic reviews.

- Demonstrates internal consistency, test-retest reliability and criterion validity.

- Assesses the risk of bias, the participant selection methods, the study protocol, and the validity, reliability and responsiveness of the study's results.

- Simple and intuitive to interpret.

- Does not assign weights to the items in the scale where there is a lack of empirical evidence to support the assignment.[20]

For peer review only - http://bmjopen.bmj.com/site/about/guidelines.xhtml

The Downs and Black Quality Index[21] meets all but the final criterion on this list. To overcome this limitation, responses have been left in their raw form (yes/no/unable to determine/not applicable, in accordance with the definitions provided by Downs and Black[21]) and presented in a table to enable the reader to visualise the category trends, as recommended by the Cochrane Handbook for Systematic Reviews of Interventions.[20]

The study details and data relating to the quality of study design and reporting were manually extracted from each paper by one reviewer (LD). The study details included medical field, study design, device category, device purpose, number of participants, age and gender of participants, and aims and outcomes of the study. The data relating to study design and reporting were extracted according to the criteria used by the Downs and Black Quality Index, divided into reporting, external validity, bias, confounding and power. Where there were uncertainties as to how the study fitted with the Downs and Black Quality Index criteria, the other two authors were consulted and in all cases, an agreement was reached.

RESULTS

The search yielded 4505 records and, after removing duplicates, 3084 abstracts were screened. A total of 350 studies met the inclusion criteria and were included in the review (Figure 1).

Due to the large number of included studies, this paper gives an overview of the level of evidence found for 3D printed medical devices in each medical field and then focuses on the outcomes of the Level 1 studies. Information for all studies included in the review and a critical appraisal of all comparative studies is provided in the supplementary material.

A wide range of 3D printed devices have been developed and clinically trialled. The most common device types were anatomical models for preplanning surgeries (40.9%), followed by guides to aid in surgery (37.1%). The studies covered 9 medical fields, with the majority (58.3%) of studies falling into the Oral and Maxillofacial Surgery field, which included dentistry and orthopaedic surgery of the jaw, face and skull, and those covering the Musculoskeletal System (23.7%) making up the second group. Some studies spanned multiple medical fields, such as neoplasms that occur in the circulatory system. In these cases, the authors selected the most prominent field of the paper. Only 14.0% of included studies used a control group (Levels 1 to 3 in Figure 2), whereas 41.4% were level 5 studies. The average number of participants per controlled trial was 41 (36 for RCTs), compared to 17 for all studies.

All 21 Level 1 studies fell within the Oral and Maxillofacial Surgery or Musculoskeletal System categories, with one cross-field study fitting in both Oral and Maxillofacial Surgery and Neoplasms (Table 2 summarises all RCTs). All Level 1 studies, with the exception of Stephens et al.,[22] used objective measures as their indicators of device efficacy and effectiveness. Of these, the 7 models used for preoperative planning were all modelling bone or fracture fixation plates, though the locations spanned skull, face, mandible, arm, spine, hip and ankle. All aimed to assess the effectiveness of using the models for surgical planning in treating specific conditions. Six out of 7 studies used operating time, 4 used accuracy of the surgery and 3 used blood loss as key indicators of device effectiveness. Aesthetics, recovery time and post-surgery function were also used as indicators of device effectiveness. All Level 1 studies of 3D printed models for preoperative planning that used time, accuracy or blood loss as indicators of device effectiveness reported that the test group had better outcomes than the control group, with statistical significance at $p<0.05$ found for 4 studies that assessed operating time, 3 studies that assessed accuracy, and 2 studies that assessed blood loss. The two studies that assessed recovery time found no difference between groups, one of the two studies that assessed user function found statistically significant differences in the test group, and the other found no difference between groups.

Of the 11 RCTs that assessed 3D printed guides or templates that supported navigation and surgery (of the mandible, teeth, shoulder, spine, hip or knee), 9 studies used accuracy of positioning as an indicator of device effectiveness, all of which found an improvement when using the guide or template, and 8 of which showed statistically significant results at $p<0.05$. All 4 studies that used time as an indicator of device effectiveness showed decreased operating times when using the guide or template (all with $p<0.05$), and 5 of the 6 studies that used changes at the operation site (blood loss, inflammatory response, tactile sensation or aesthetics) found statistically significant improvements at $p<0.05$.

Only 3 therapeutic devices were tested using RCTs. These were zirconia crowns, which were found to have a better marginal gap and internal fit than the conventional crowns tested ($p<0.05$), cranial implants, which showed less vertical ridge resorption over time ($p<0.05$), and osteosynthesis plates for intercondylar humeral

For peer review only - http://bmjopen.bmj.com/site/about/guidelines.xhtml

fractures, which showed a reduction in operating time ($p<0.05$) but no difference in elbow function during recovery.

While most Level 1 studies found statistically significant improvements at $p<0.05$ when using the 3D printed device, none performed an analysis on the clinical relevance of these improvements.

For peer review only

For peer review only - http://bmjopen.bmj.com/site/about/guidelines.xhtml

Table 2. Summary of RCTs (Type: P = preoperative planning, S = surgical tool or guide, T = therapeutic device)

Sector	First author, date	Device	Type	Size (n)	Participant details	Aim	Outcomes
Neoplasms	de Farias, 2014[23]	Mandible model	P	37 (Test=17, Control=20)	Age: 9 to 74 years (Test mean = 39.57 years, Control mean = 53.11 years). Test = 14M/3F, Control = 15M/5F	Aim: to evaluate the efficacy of using 3D printed models to plan head and neck surgeries.	Reconstruction time decreased; Test = 43.7 mins, Control = 127.7 mins, p = 0.001. Though statistical significance was not demonstrated, the size of the bone flap taken for reconstruction was typically smaller, and the aesthetic results better in the test group.
Oral and Maxillofacial	Ahrberg, 2015[24]	All-ceramic zirconia crowns	T	25 (Same participants used for test and control)	Age: >18 years 10M/15F	Aim: to evaluate the marginal and internal fit of 3D printedzirconia crowns and three-unit fixed dental prostheses resulting from direct versus indirect digitalization.	The marginal gap was smaller; Test = 61.08 ± 24.77 µm, Control = 70.40 ± 28.87 µm, p<0.05, and the fit was better at the centro-occlusal site; Test = 155.57 ± 49.85, Control = 171.51 ± 60.98, p<0.05. The fit improved at the mid-axial wall and axio-occlusal transition but the results lacked statistical significance.
Oral and Maxillofacial	D'Urso, 1999[25]	Cranio-maxillofacial model	P	30 (Test=15, Control=15)	No information provided	Aim: to assess whether biomodels in addition to standard imaging have greater utility in patient education, diagnosis and operative planning than the standard imaging alone.	3D printed models improved operative planning accuracy; Test = 82.21%, Control = 44.09%, p<0.01 and diagnosis accuracy; Test = 95.23%, Control = 65.63%, p<0.01. Measurement accuracy improved; Test error = 7.91%, Control error = 44.14%, p<0.05). Surgeons estimated that operating time over 45 operations was also reduced by 17.63%.
Oral and Maxillofacial	Al-Ahmad, 2013[26]	Surgical guide for sagittal splitting ramus osteotomy	S	8 (One side used as test, the other as control)	Age: 18-30 years (mean=23 years)	Aim: to evaluate the effectiveness of sagittal splitting ramus osteotomy in reducing the incidence and severity of neurosensory alterations, using a 3D printed surgical guide.	The Semmes–Weinstein monofilament tactile threshold was considered abnormal at >2.83 on the χ^2 test. At 1 week after surgery; Test = 67% abnormal, Control = 83% abnormal. This difference showed statistical significance (p<0.05) at 3 months post-surgery for chin and 6 months post-surgery for lower lip. For the two-point discrimination, the lower lip showed statistical significance at 1 week (Test = 9 mm, Control = 22 mm), and the chin at 6 months (Test = 11 mm, Control = 30 mm).
Oral and Maxillofacial	Ayoub, 2014[27]	Guide for mandibular reconstruction	S	20 (Test=10, Control=10)	Age: Test=52.3 ± 19.2 years, Control=54.7 ± 14.2 years). Test=4M/6F, Control=6M/4F	Aim: to evaluate the benefits of computer-assisted mandibular reconstruction with iliac crest bone grafts regarding the intraoperative time for transplant shaping, ischemia, duration of surgery, amount of bone removed and the change in postoperative condyle position compared to conventional surgery.	Computer-assisted surgery shortened the time of transplant ischemia; Test = 96.1 ± 15.8 mins, Control = 122.9 ± 20.4 mins, p<0.005, and defect reconstruction; Test = 6.2 ± 4.9 mins, Control = 20.3 ± 7.4 mins, p<0.001. There was less bone harvested; Test = no difference regarding defect size, Control = 16.8 ± 5.6 mm larger transplant size than defect size, p<0.001. The intercondylar distance before compared to after surgery was less affected; Test = 1.3 ± 0.2 mm, Control = 5.5 ± 2.5 mm, p<0.001.
Oral and Maxillofacial	Goh, 2015[28]	Polycaprolactone scaffold for ridge preservation	T	13 (Test=6, Control=7)	Age: 29–60 years (mean=46.8 years). 7M/7F (1 lost to follow-up, so excluded from results)	Aim: to evaluate the feasibility and effectiveness of using a polycaprolactone scaffold in fresh extraction sockets for ridge preservation.	The test group showed less vertical ridge resorption in all aspects (mesio-buccal, mid-buccal and disto-buccal) with statistically significant results in the mesio-buccal aspect; Test = 0.13 ± 0.96 mm, Control = -2.18 ± 1.02mm, p = 0.008), with better maintenance of ridge height after 6 months.
Oral and Maxillofacial	Van de Velde, 2010[29]	Maxilla surgical guide	S	13 (Test=36 tooth implants, Control=34 tooth implants)	Age: 39-75 years (mean =55.7 years), 4M/9F	Aim: to compare the outcome of dental implants placed using a flapless protocol with a 3D printed guide and immediate loading with a conventional protocol and loading.	The height of the attached mucosa at 1 week post-surgery was less; Test = 3.26 ± 1.57 mm, Control = 6.01 ± 1.10 mm, p<0.05. Marginal bone levels were not statistically significantly different between test and control implants; Test = 1.95 mm ± 0.70 and 1.93mm ± 0.42 after 18 months. Opinion about speech, function, aesthetics and self-confidence was statistically significantly better for the test side after 1 week when using a VAS rating scale, p<0.05.
Oral and Maxillofacial	Vercruyssen, 2014[30]	Surgical guides for edentulous jaws	S	59 (72 jaws, 12 in each group)	Age:31-78 years 31 male jaws, 41 female jaws	Aim: To assess the accuracy of guided surgery compared to mental navigation or the use of a surgical template, in fully edentulous jaws.	There was lower deviation at the entry point (1.4mm), at the apex (1.6 mm) and angular deviation (3.0°) when using the 3D printed guiding systems (all p<0.05). There were negligible differences between bone and mucosa support or type of guidance.
Musculoskeletal	Chareancholvanich, 2013[31]	Cutting guides for total knee replacement	S	80 (Test=40, Control=40)	Age: 55-84 years (mean = 69.5 years) 6M/34F	Aim: to compare patient-specific cutting guides with conventional instrumentation in total knee replacement.	The tibial component in the test group was marginally closer to neutral alignment; Test = 89.8 ± 1.2°, Control = 90.5 ± 1.6°, p=0.03. The 3D printed guides also shortened the bone-cutting time by 3.6 mins (p<0.001) and the operating time by 5.1 mins (p=0.019), without differences in post-operative blood loss (p=0.528) or need for blood transfusion (p=0.789). These minimal advantages of the 3D printed guides are unlikely to be clinically relevant.
Musculoskeletal	Chen, 2015[32]	Guide plate for pedicle screw fixation	S	43 (Test=20, Control=23)	Age: Test=35-70 years (mean=52.3 years) Control=37-72 years (mean=55.4 years) Test=9M/11F, Control=12M/11F	Aim: to evaluate the clinical efficacy of use of a 3D printing guide plate in posterior lumbar pedicle screw fixation.	Placement time for each screw was shorter (Test = 4.9 ± 2.1 mins, Control = 6.5 ± 2.2 mins), the amount of hemorrhage was less (Test = 8.0 ± 11.1 mL, Control = 59.9 ± 13.0 mL), and the fluoroscopy times of each screw placement was lower (Test = 0.5 ± 0.4 mins, Control = 1.2 ± 0.7 mins), p<0.05 for all tests. The excellent and good screw placement rate was 100% in the test group and 98.4% in the control group, with no statistical difference. No complications were reported.

For peer review only - http://bmjopen.bmj.com/site/about/guidelines.xhtml

Musculoskeletal	Du, 2013[33]	Templates to aid in pin placement in hip resurfacing arthroplasty	S	34 (Test-16, Control=18)	Ages: 37-55 years	Aim: To assess whether 3D printed patient-specific templates aid in accurate intraoperative pin placement.	The prosthesis stem shaft angle was greater; Test = 138.68 ± 8.85°, Control = 118.9 ± 12.8, p=0.001. The locating template can provide precise and dependable location for hip resurfacing femoral components during arthroplasty and ensure the valgus stem placement necessary for optimal outcomes.
Musculoskeletal	Gan, 2015[34]	Navigational template for total knee arthroplasty	S	70 (Test=35, Control=35)	Age: Test=63-75 years (mean=68 years), Control=64-72 years (mean=67.8 years) Test=10M/25F, Control=9M/26F	Aim: to validates a novel patient-specific navigational template for total knee arthroplasty.	The 3D printed navigational template reduced operation time (Test = 45 ± 8 mins, Control = 60 ± 10 mins, p<0.001) and blood loss (Test = 200 ± 45 mL, Control = 290±60 mL, p<0.001). The mean deviation from the neutral axis also decreased for the frontal femoral component (Test = 1.0±0.8°, Control = 2.6±1.8°, p<0.001) and the frontal tibial component (Test = 1.2±1.0°, Control = 2.8±1.5°, p<0.001).
Musculoskeletal	Hendel, 2012[35]	Guide for determining glenoid component position	S	31 (Test=15, Control=16)	No information provided	Aim: to compare patient-specific instruments with standard surgical instruments in determining glenoid component position.	The 3D printing technology decreased the average deviation of implant position for inclination; Test = 2.9 ± 3.4°, Control = 11.6 ± 7.0°, p<0.0001, and medial-lateral offset; Test = 1.0±0.9mm, Control = 1.9±1.0mm, p<0.05. The greatest benefit was observed in patients with retroversion >16˚; Test = 1.2±2.0˚, Control = 10±4.4˚, p<0.001.
Musculoskeletal	Maini, 2016[36]	Plate for acetabulum fracture fixation	P	21 (Test=10, Control=11)	Age: 18-60 years (mean=38.7 years) Test=9M/1F, Control=9M/2F	Aim: to evaluate the accuracy of patient-specific pre-contoured plates.	Greater reduction was achieved, as evaluated by CT scan; Test = 8.36 ± 5.82 mm, Control = 2.99 ± 1.34mm, p<0.05. Reduced blood loss; Test = 620 ± 247 mL, Control = 720 ± 286 mL, and surgical time; Test = 120 ± 38 mins, Control = 132 ± 41 mins, were observed but the results were not statistically significant (p>0.05). All plates fitted well to the pelvis intra-operatively.
Musculoskeletal	Merc, 2013[37]	drill guide for screw placement	S	19 (Test=9 (54 screws), Control=10(54 screws))	Age: Test=59 ± 5 years, Control=62 ±12 years Test=4M/5F, Control=5M/5F	Aim: to evaluate the accuracy of a multi-level drill guide template for lumbar and first sacral pedicle screw placement and to compare it with the freehand technique under fluoroscopy supervision.	The incidence of cortex perforation was reduced; Test = 0, Control = 8, p<0.05. So was the deviation of pedicle screw position in the sagittal plane; Test = 2 ± 10˚, Control = -12 ± 8˚, p<0.05, but there was no discernable difference in the transverse plane deviation or displacement in sagittal or transverse planes.
Musculoskeletal	Shuang, 2016[38]	Osteosynthesis Plates	T	13 (Test=6, Control=7)	Age: Test=31-62 years (mean=46.2 years) Control=27-59 years (mean=40.3 years) Test= 4M/2F, Control=6M/1F	Aim: to evaluate the efficacy custom 3D printed osteosynthesis plates in the treatment of intercondylar humeral fractures.	Operative time was shorter; Test = 70.6 ± 12.1 mins, Control = 92.3 ± 17.4 mins, p<0.05. The mean time to bone union was 3.4 months. No difference was found in the rate of patients with good elbow function, or in the ranges of elbow flexion/extension and pronation/supination (all p>0.05).
Musculoskeletal	Stephens, 2016[22]	Bone models	P	58 (Test=29, Control=29)	Age: >60 years (mean=73 years) 5M/53F	Aim: to investigate the efficacy of 3D printed bone models as a tool to facilitate initiation of bisphosphonate treatment among individuals who are newly diagnosed with osteoporosis.	Using the 9-item Brief Illness Perception Questionnaire with a 0-10 rating scale, the test group were more emotionally affected by osteoporosis immediately after the interview (Test = 4.08±0.41, Control = 2.89 ± 0.40, p>0.05) and reported a greater understanding of osteoporosis at the 2 month follow-up (Test = 7.19 ± 0.51, Control = 5.72 ± 0.49, p<0.04).
Musculoskeletal	Wu, 2011[39]	Spine model	P	62 (Test=34, Control=28) 677 screws inserted	Age: 4-22 years (mean=11 years)	Aim: to compare the accuracy and safety of pedicle screw placement in congenital scoliosis using the 3D printing technique versus conventional fluoroscopy.	Results for thoracolumbar and lumbar, respectively: Shorter operation time; Test = 6.5 and 5.1 hours, Control = 8.5 and 6.3 hours, p<0.05. Higher scoliosis correction ratio; Test = 83.8 and 81.8, Control = 76.6 and 64.7, p<0.05. Accuracy of screw placement; Test = 94.4% and 91.6%, Control = 86.1% and 82.0%, p<0.05.
Musculoskeletal	Yang, 2016[40]	Model of trimalleolar fracture	P	30 (Test=15, Control=15)	Age: 31-42 years (mean=36.5 years) 16M/14F	Aim: to evaluate the effectiveness of using 3D printed models for surgical planning in treating trimalleolar fractures and in physician-patient communication.	Shorter operation time; Test = 184.32 ± 4.65 mins, Control = 212.32 ± 8.17 mins, p<0.001. Less blood loss; Test = 846.68 ± 26.11 mL, Control = 1029.65 ± 72.18 mL, p<0.001. No statistically significant differences were observed in complication rate, length of hospital stay, and postoperative radiological outcomes (all, p>0.05).
Musculoskeletal	You, 2016[41]	Proximal humeral fracture model	P	66 (Test=34, Control=32)	61-76 years (Test = 66.09±4.09 years, Control = 66.28± 4.10 years) 27M/39F	Aim: to investigate the feasibility and clinical potential of using 3D printed models versus the conventional thin-layer CT scan for the treatment of proximal humeral fractures in old people.	Surgery time decreased; Test = 77.65 ± 8.09 mins, Control = 92.03 ± 10.31 mins, p<0.05, there was less blood loss; Test = 235.29 ± 63.40 mL, Control = 281.25 ± 57.85 mL, p<0.05, and a lower number of fluoroscopy; Test = 7.12 ± 1.57, Control = 10.59 ± 1.36, p<0.05. The results showed no statistically significant difference in time to union (p>0.05).
Musculoskeletal	Zhang, 2011[42]	Acetabular navigational template	S	22 (Test=11, Control=11)	Age: Test=48.6 ± 6.8 years, Control=49.3 ± 4.9 years Test=7M/4F, Control=5M/6F	Aim: to compare a customised 3D printed acetabular navigational template for total hip arthroplasty (THA) to conventional THA in adult single development dysplasia of the hip.	The templates facilitated accurate placement of acetabular components in dysplasia of acetabulum. At 1 year follow-up there were smaller differences from the predetermined angles of 45° abduction and 18° anteversion; Test = 1.6 ± 0.4°, 1.9 ± 1.1°, Control = 5.8 ± 2.9°, 3.9 ± 2.5°, p<0.05.

Critical appraisal of the RCTs from each medical field are shown in Table 3. The table includes the abridged quality index questions. Studies with higher levels of evidence typically rated better across all areas assessed using the quality index. However, many of the RCTs did not demonstrate external validity, and did not blind

For peer review only - http://bmjopen.bmj.com/site/about/guidelines.xhtml

participants or those measuring the outcomes, or assess confounding factors between groups. Only 57% of the RCTs included follow-up.

Table 3. Critical appraisal of studies with the highest level of evidence from each medical field.

- + Yes
- - No
- ? Unable to determine
- x Not applicable

Medical sector	First author	Date	1. Clear hypothesis/aim/objective	2. Clear outcome measures	3. Patient characteristics described	4. Interventions clearly described	5. Distributions of confounders described	6. Findings clearly described	7. Estimates given of random variability	8. Adverse events reported	9. Patients lost to follow-up described	10. Probability values reported	11. Recruitment pool represents population	12. Participants represent population	13. Staff/places/facilities match std. treatment	14. Participants blinded to intervention	15. Those measuring outcomes blinded	16. Data dredging reported	17. Adjusted for different lengths of follow-up	18. Appropriate statistical tests	19. Reliable compliance with intervention	20. Accurate/reliable outcome measures	21. Groups recruited from same population	22. Groups recruited over same timeframe	23. Subjects randomised into intervention	24. Randomised intervention concealed	25. Adjustment for confounding	26. Losses to follow-up accounted for	27. Sufficient power
			Reporting										External validity			Internal validity - bias							Internal validity - confounding						Power
Neoplasms	de Farias	2014	+	+	+	+	+	+	+	+	x	+	?	?	+	+	+	+	x	+	+	+	+	+	+	+	?	x	+
Oral and Maxillofacial	Ahrberg	2015	+	+	+	+	+	+	+	-	x	+	?	?	+	+	+	+	x	+	+	+	+	+	+	+	+	x	+
Oral and Maxillofacial	D'Urso	1999	+	+	+	+	+	+	+	-	x	-	?	?	+	-	-	+	x	+	+	+	+	+	+	?	-	x	+
Oral and Maxillofacial	Al-Ahmad	2013	+	+	+	+	x	+	+	-	+	+	?	?	+	+	+	+	+	+	+	+	+	+	+	+	+	+	+
Oral and Maxillofacial	Ayoub	2014	+	+	+	+	-	+	+	-	x	-	?	?	+	-	-	+	x	+	+	+	+	+	+	?	-	x	+
Oral and Maxillofacial	Goh	2015	+	+	+	+	-	+	+	-	+	+	?	?	+	-	+	+	+	+	+	+	+	+	+	?	-	+	+
Oral and Maxillofacial	Van de Velde	2010	+	+	+	+	-	+	+	+	+	+	?	?	+	-	+	+	+	+	+	+	+	+	+	?	-	+	+
Oral and Maxillofacial	Vercruyssen	2014	+	+	+	+	-	+	+	+	+	-	?	?	+	-	+	+	+	+	+	+	+	+	+	?	-	+	+
Musculoskeletal	Chareancholvanich	2013	+	+	+	+	-	+	+	+	+	+	?	?	+	-	+	+	-	+	+	+	+	+	+	?	-	+	+
Musculoskeletal	Chen	2015	+	+	+	+	+	+	+	+	+	-	?	?	+	+	-	+	+	+	+	+	+	+	+	+	+	+	+
Musculoskeletal	Du	2012	+	+	-	-	+	+	+	-	+	+	?	?	+	-	-	+	-	+	+	+	+	+	+	?	-	+	+
Musculoskeletal	Gan	2015	+	+	+	+	+	+	+	+	x	+	?	?	+	-	+	+	x	+	+	+	+	+	+	?	-	x	+
Musculoskeletal	Hendel	2012	+	+	-	+	-	+	+	+	-	+	+	+	+	-	+	+	?	+	+	+	+	+	+	-	-	?	+
Musculoskeletal	Maini	2016	+	+	+	+	-	+	+	+	x	+	?	?	+	-	-	+	x	+	+	+	+	+	+	?	-	x	+
Musculoskeletal	Merc	2013	+	+	+	+	+	+	+	+	x	+	+	+	+	-	-	+	x	+	+	+	+	+	+	?	-	x	+
Musculoskeletal	Shuang	2016	+	+	-	+	+	+	+	+	+	+	?	?	+	-	-	+	?	+	+	+	+	+	+	+	+	+	+
Musculoskeletal	Stephens	2016	+	+	+	+	-	+	+	-	+	+	?	?	?	+	+	+	+	+	+	+	+	+	+	+	-	+	+
Musculoskeletal	Wu	2011	+	+	-	+	-	+	+	+	+	+	+	+	+	-	-	+	-	+	+	+	+	+	+	?	-	+	+
Musculoskeletal	Yang	2016	+	+	-	+	-	+	+	+	x	+	?	?	+	-	-	+	x	+	+	+	+	+	+	?	-	x	+
Musculoskeletal	You	2016	+	+	-	+	+	+	+	+	+	+	+	+	+	-	-	+	-	+	+	+	+	+	+	?	+	+	+
Musculoskeletal	Zhang	2011	+	+	-	+	+	+	+	-	+	+	?	?	+	-	-	+	-	+	+	+	+	+	+	?	+	+	+

DISCUSSION

The results show that a wide range of 3D printed devices have been clinically trialled but few papers have rigorously assessed the efficacy or effectiveness of clinical 3D printed devices. The 3D printed devices tested were mainly designed for planning surgeries, particularly using anatomical models or surgical guides to aid cutting or navigating, with little attention dedicated to 3D printed interventions. This is likely due to the lower complexity of the anatomical models and surgical guides and the smaller risk to the patient than therapeutic devices, resulting in less rigorous regulation and safety testing requirements. Approximately twice as many studies were in Oral and Maxillofacial Surgery than in the Musculoskeletal System, the two largest fields, but the Musculoskeletal System, despite its more recent uptake of 3D printing, had almost double the number of Level 1 studies. This is perhaps because clinical trials in musculoskeletal surgery typically aim to demonstrate quantitative clinical outcomes, driving more rapid growth in the level of evidence of studies than oral and maxillofacial surgery where aesthetics is a priority and individual outcomes are harder to quantify.

The RCT listed under Neoplasms was an anatomical model used for planning mandibular surgery, but with a focus on tumour removal, fitting into both categories. Therefore no RCTs were found outside the Oral and Maxillofacial Surgery or Musculoskeletal System fields. The primary indicators of success used to determine

For peer review only - http://bmjopen.bmj.com/site/about/guidelines.xhtml

the efficacy and effectiveness of 3D printed devices across all medical fields were operating time, accuracy of surgery or positioning, fit, changes at the operation site (particularly blood loss, inflammation and aesthetics), recovery time and functional outcomes. From the studies that compared against a control, the 3D printed devices used for preoperative planning and aiding surgery consistently found decreases in operating time and increases in surgical accuracy, the two most commonly reported indicators of effectiveness. Operating time is an obvious choice as an indicator for device success because it is easy to measure and quantify and corresponds to decreased blood loss and faster recovery. The main aims of anatomical models and surgical guides are to provide better information to the surgeon on the surgical site and to guide the surgeon's hand. Therefore measuring the accuracy of the surgeries indicates whether these aims have been met. To make more decisive conclusions regarding the performance of 3D printed medical devices in Oral and Maxillofacial Surgery and Musculoskeletal Surgery it is important to assess the long-term effects. Few papers assessed the long-term differences of using the 3D printed devices and those that did had mixed results as to whether there were any differences in recovery times and outcomes. The other aspect that should be addressed in future studies is the appropriate choice of outcome measures. In the studies analysed in this review there appears to be no evaluation of how the selected outcome measures match the purpose of the device.

Therapeutic devices such as implants, prosthetic limbs and orthotics offer the promise of revolutionising the medical industry because of their ability to be custom-made for the patient, but most therapeutic devices are still early-stage, with little research into their efficacy and effectiveness in a clinical setting. There were not enough therapeutic devices tested against a control group to find and evaluate repeated measures of success.

Most studies reviewed were poor quality, meaning that their study designs demonstrated levels of evidence for the 3D printed device of 4 or 5, with no use of a control group. The 41.4% of papers that reported on individual case studies or provided only qualitative results did not clinically validate the devices that they reported on, and therefore add little value to their fields. Few included follow-up, or addressed the external validity, bias or confounding factors (see supplementary material for full comparison of studies). The vast majority of papers reported positive outcomes for the patients, but few studies demonstrated clinically significant findings. Even the Level 1 studies demonstrated limited external validity and rarely blinded participants or those measuring the outcomes to the intervention.

The RCTs all demonstrated statistically significant results in favour of using the 3D printed device over the current clinical practice to which it was compared. However, one RCT[31] concluded that the improvement with 3D printing was too small to provide a clinical advantage and none performed an analysis on the clinical relevance of these differences. The studies consistently used p-values of <0.05 as their measure of device success, but taking into consideration the false discovery rate, it is likely that this will only show a real effect about 70% of the time under ideal testing conditions.[43] While the critical analysis in Table 3 shows all Level 1 studies as adequately powered, this is because the Quality Index bases its definition of "adequately powered" on whether a difference due to chance is less than 5%. Therefore, given that all Level 1 studies had 40 or fewer participants in each group, many of these studies are likely to be underpowered.[43] Considering the possibility of underpowered studies, the publication bias towards positive trials, and the fact that all RCTs in this review were single site trials, the likelihood that the results shown in these papers reflect a real effect is lower than suggested by the reviewed studies.[44] Until independent groups start validating 3D printed devices, it is also difficult to avoid researcher bias.

Most 3D printed device studies that were excluded from this review had uses in medical training, or as moulds for manufacturing and therefore did not meet the inclusion requirements. A few other studies were excluded because they had not been translated into English.

Previous reviews have documented the uses of 3D printing for developing patient-specific medical devices. However, there has been limited research into assessing the efficacy and effectiveness of these devices. This rigorous systematic review design is the first to compare 3D printed devices across all medical fields and assess their efficacy and effectiveness, describing key benefits that have been found from using 3D printed devices clinically. The progress made in different medical fields is compared using validated quality assessment and clinical level of evidence tools. It demonstrates that the fields of Oral and Maxillofacial Surgery and the Musculoskeletal System are leading the way in validating 3D printed devices for clinical use. Multiple high-quality studies have been performed on surgical guides for maxillofacial, hip and knee surgeries. This growing body of comparable high-quality research sets an example for other fields to emulate

For peer review only - http://bmjopen.bmj.com/site/about/guidelines.xhtml

in order to demonstrate the efficacy and effectiveness required to integrate these 3D printed devices into clinical practice.

A critical appraisal of the efficacy and effectiveness of 3D printed devices across medical fields provides clinicians with an evidence-based approach to determine the applicability of 3D printing within their field. It also gives researchers an overview of areas that require further research and validation. It encourages investigators to discover what methods have been successfully validated in other medical fields and promotes potential collaborations between fields.

3D printing provides a way of customising devices to improve patient outcomes. Available techniques and materials will increase, as 3D printing technology continues to be developed. There is therefore a growing need for validation of new devices, materials and techniques to ensure best patient outcomes. Much research has already gone into developing 3D printed devices for medical purposes. However, this drive for new technology development has not yet been matched with a drive for critical appraisal of the devices to demonstrate their efficacy and effectiveness. Even the fields that are leading the way in critically evaluating new 3D printed devices will be required to increase their output as 3D printing continues to grow in popularity and functionality.

The early research covered by this review shows that 3D printing can be valuable for use in medicine. The next important step to take is growing the body of research that focuses on validating 3D printed devices. All fields require more rigorous and long-term assessments into the efficacy, effectiveness and safety of 3D printed devices before they are introduced into standard clinical practice. The study is limited to a critical appraisal of individual studies, rather than a meta-analysis, because of the breadth of uses for anatomical models, surgical guides and therapeutic devices and the lack of comparable hypotheses. Funders can take an active role in promoting not only early technological development, but also the subsequent clinical trials. Demonstration of clinical efficacy, effectiveness and device safety will become increasingly important as higher risk 3D printed devices are developed and unconventional manufacturers, such as hospitals and clinics, incorporate 3D printing of patient-specific medical devices into standard clinical practice.

Fields such as Oral and Maxillofacial surgery, who were early in the uptake of 3D printing, are beginning to stabilise, with a steady number of studies being published each year, but other fields, such as Musculoskeletal and Circulatory Systems, are more recently gaining traction, with increasing numbers of studies performed each year. 3D printed drug delivery devices and biological 3D printing technologies for printing tissue show huge promise, but have not been clinically trialled and so are not included here.[45] This is likely to change within the near future. It is therefore recommended that in this fast-growing and dynamic environment, this review is updated every few years.

CONCLUSION

This review demonstrates that 3D printing is already being used to develop a broad range of medical devices with clinically effective results. The medical fields of Oral and Maxillofacial Surgery and the Musculoskeletal System are leading the way in validating the efficacy and effectiveness of 3D printed devices, and have found that 3D printed anatomical models and surgical guides are reducing operating times and increasing surgical accuracy. However, the efficacy and effectiveness of 3D printed devices remains undetermined for the majority of medical fields. 3D printed devices can have an important role to play in healthcare, but more rigorous and long-term assessments are needed to determine if 3D printed devices are clinically relevant before these devices can become part of standard clinical practice.

Contributors: LD, JB and MT designed the review. LD and JB collected and analysed the data with input from MT. LD drafted the manuscript. All authors contributed to subsequent drafts and approved the final version of the manuscript.

Funding: The researchers are financially supported by the General Sir John Monash Foundation and the Wellcome Trust.

Competing interests: None declared

Data sharing statement: All additional data is available in the supplementary material.

For peer review only - http://bmjopen.bmj.com/site/about/guidelines.xhtml

REFERENCES

1. Zadpoor AA, Malda J. Additive Manufacturing of Biomaterials, Tissues, and Organs. Ann Biomed Eng. 2017;45(1):1–11.
2. Banks J. Adding value in additive manufacturing: Researchers in the United Kingdom and Europe look to 3D printing for customization. IEEE Pulse. 2013;4(6):22–6.
3. Royal Academy of Engineering. Additive Manufacturing : Opportunities and Constraints [Internet]. Royal Academy of Engineering. 2013. Available from: http://www.raeng.org.uk/publications/reports/additive-manufacturing
4. Lee Ventola C. Medical applications for 3D printing: Current and projected uses. P T. 2014;39(10):704–11.
5. Tack P, Victor J, Gemmel P, Annemans L. 3D-printing techniques in a medical setting: a systematic literature review. Biomed Eng Online. 2016;15(1):115.
6. Caffrey T, Wohlers T. Additive manufacturing state of the industry. Manuf Eng. 2015;154(5):67–78.
7. Mulford JS, Babazadeh S, Mackay N. Three-dimensional printing in orthopaedic surgery: Review of current and future applications. ANZ J Surg. 2016;
8. Revicki DA, Frank L. Pharmacoeconomic evaluation in the real world: Effectiveness versus efficacy studies. Vol. 15, PharmacoEconomics. 1999. p. 423–34.
9. FDA. Technical Considerations for Additive Manufactured Devices - Draft Guidance for Industry and Food and Drug Administration Staff. 2016.
10. Bauermeister AJ, Zuriarrain A, Newman MI. Three-Dimensional Printing in Plastic and Reconstructive Surgery: A Systematic Review. Ann Plast Surg. 2015;
11. Soon DSC, Chae MP, Pilgrim CHC, Rozen WM, Spychal RT, Hunter-Smith DJ. 3D haptic modelling for preoperative planning of hepatic resection: A systematic review. Vol. 10, Annals of Medicine and Surgery. 2016. p. 1–7.
12. Patzelt SBM, Spies BC, Kohal RJ. CAD/CAM-fabricated implant-supported restorations: A systematic review. Clin Oral Implants Res. 2015;26:77–85.
13. Bidra AS, Taylor TD, Agar JR. Computer-aided technology for fabricating complete dentures: Systematic review of historical background, current status, and future perspectives. Vol. 109, Journal of Prosthetic Dentistry. 2013. p. 361–6.
14. Tarsitano A, Del Corso G, Ciocca L, Scotti R, Marchetti C. Mandibular reconstructions using computer-aided design/computer-aided manufacturing: A systematic review of a defect-based reconstructive algorithm. J Cranio-Maxillofacial Surg. 2015;43(9):1785–91.
15. Martelli N, Serrano C, Van Den Brink H, Pineau J, Prognon P, Borget I, et al. Advantages and disadvantages of 3-dimensional printing in surgery: A systematic review. Vol. 159, Surgery (United States). 2016. p. 1485–500.
16. Zhang QM, Chen JY, Li H, Chai W, Ni M, Zhang ZD, et al. No Evidence of Superiority in Reducing Outliers of Component Alignment for Patient-specific Instrumentation for Total Knee Arthroplasty: A Systematic Review. Vol. 7, Orthopaedic Surgery. 2015. p. 19–25.
17. Liberati A, Altman DG, Tetzlaff J, Mulrow C, Gøtzsche PC, Ioannidis JPA, et al. The PRISMA statement for reporting systematic reviews and meta-analyses of studies that evaluate health care interventions: explanation and elaboration. In: Journal of clinical epidemiology. 2009. p. e1-34.
18. Oxford Centre for Evidence-based Medicine. Levels of Evidence. Univ Oxford [Internet]. 2009;4–5. Available from: www.cebm.net
19. World Health Organization. International Statistical Classification of Diseases and Related Health Problems (ICD) 10th Revision - Version:2010 [Internet]. Occupational Health. 2010. Available from: http://apps.who.int/classifications/icd10/browse/2010/en
20. Higgins JPT, Green S. Cochrane Handbook for Systematic Reviews of Interventions Version 5.1.0

For peer review only - http://bmjopen.bmj.com/site/about/guidelines.xhtml

[updated March 2011]. Vol. 4, The Cochrane Collaboration. 2011.

21. Downs SH, Black N. The feasibility of creating a checklist for the assessment of the methodological quality both of randomised and non-randomised studies of health care interventions. J Epidemiol Community Health. 1998;52(6):377–84.

22. Stephens MH, Grey A, Fernandez J, Kalluru R, Faasse K, Horne A, et al. 3-D bone models to improve treatment initiation among patients with osteoporosis: A randomised controlled pilot trial. Psychol Health. 2016;31(4):487–97.

23. de Farias TP, Dias FL, Galvão MS, Boasquevisque E, Pastl AC, Albuquerque Sousa B. Use of prototyping in preoperative planning for patients with head and neck tumors. Head Neck. 2013;1–31.

24. Ahrberg D, Lauer HC, Ahrberg M, Weigl P. Evaluation of fit and efficiency of CAD/CAM fabricated all-ceramic restorations based on direct and indirect digitalization: a double-blinded, randomized clinical trial. Clin Oral Investig. 2015;

25. D'Urso PS, Barker TM, Earwaker WJ, Bruce LJ, Atkinson RL, Lanigan MW, et al. Stereolithographic biomodelling in cranio-maxillofacial surgery: a prospective trial. J Craniomaxillofac Surg. 1999;27(1):30–7.

26. Al-Ahmad HT, M Saleh MW, Hussein AM. Evaluation of an innovative computer-assisted sagittal split ramus osteotomy to reduce neurosensory alterations following orthognathic surgery: a pilot study. Int J Med Robot. 2013;9(2):134–41.

27. Ayoub N, Ghassemi A, Rana M, Gerressen M, Riediger D, Hölzle F, et al. Evaluation of computer-assisted mandibular reconstruction with vascularized iliac crest bone graft compared to conventional surgery: a randomized prospective clinical trial. Trials. 2014;15:114.

28. Goh BT, Teh LY, Tan DBP, Zhang Z, Teoh SH. Novel 3D polycaprolactone scaffold for ridge preservation - a pilot randomised controlled clinical trial. Clin Oral Implants Res. 2015;26(3):271–7.

29. Van de Velde T, Sennerby L, De Bruyn H. The clinical and radiographic outcome of implants placed in the posterior maxilla with a guided flapless approach and immediately restored with a provisional rehabilitation: A randomized clinical trial. Clin Oral Implants Res. 2010;21(11):1223–33.

30. Vercruyssen M, Cox C, Coucke W, Naert I, Jacobs R, Quirynen M. A randomized clinical trial comparing guided implant surgery (bone- or mucosa-supported) with mental navigation or the use of a pilot-drill template. J Clin Periodontol. 2014;41(7):717–23.

31. Chareancholvanich K, Narkbunnam R, Pornrattanamaneewong C. A prospective randomised controlled study of patient-specific cutting guides compared with conventional instrumentation in total knee replacement. Bone Joint J. 2013;95–B:354–9.

32. Chen HL, Wu DY, Yang HL, Guo KJ. Clinical Use of 3D Printing Guide Plate in Posterior Lumbar Pedicle Screw Fixation. Med Sci Monit. 2015;21:3948–54.

33. Du H, Tian XX, Li T Sen, Yang JS, Li KH, Pei GX, et al. Use of patient-specific templates in hip resurfacing arthroplasty: Experience from sixteen cases. Int Orthop. 2013;37(5):777–82.

34. Gan Y, Ding J, Xu Y, Hou C. Accuracy and efficacy of osteotomy in total knee arthroplasty with patient-specific navigational template. Int J Clin Exp Med. 2015;8(8):12192–201.

35. Hendel MD, Bryan J a, Barsoum WK, Rodriguez EJ, Brems JJ, Evans PJ, et al. Comparison of patient-specific instruments with standard surgical instruments in determining glenoid component position: a randomized prospective clinical trial. J Bone Joint Surg Am. 2012;94(23):2167–75.

36. Maini L, Sharma A, Jha S, Sharma A, Tiwari A. Three-dimensional printing and patient-specific pre-contoured plate: future of acetabulum fracture fixation? Eur J Trauma Emerg Surg. 2016;1–10.

37. Merc M, Drstvensek I, Vogrin M, Brajlih T, Recnik G. A multi-level rapid prototyping drill guide template reduces the perforation risk of pedicle screw placement in the lumbar and sacral spine. Arch Orthop Trauma Surg. 2013;133(7):893–9.

38. Shuang F, Hu W, Shao Y, Li H, Zou H. Treatment of Intercondylar Humeral Fractures With 3D-Printed Osteosynthesis Plates. Medicine (Baltimore). 2016;95(3):e2461.

For peer review only - http://bmjopen.bmj.com/site/about/guidelines.xhtml

39. Wu Z-X, Huang L-Y, Sang H-X, Ma Z-S, Wan S-Y, Cui G, et al. Accuracy and safety assessment of pedicle screw placement using the rapid prototyping technique in severe congenital scoliosis. J spinal Disord {&} Tech. 2011;24(7):444–50.

40. Yang L, Shang XW, Fan JN, He ZX, Wang JJ, Liu M, et al. Application of 3D Printing in the Surgical Planning of Trimalleolar Fracture and Doctor-Patient Communication. Biomed Res Int. 2016;2016.

41. You W, Liu LJ, Chen HX, Xiong JY, Wang DM, Huang JH, et al. Application of 3D printing technology on the treatment of complex proximal humeral fractures (Neer3-part and 4-part) in old people. Orthop Traumatol Surg Res. 2016;

42. Zhang YZ, Chen B, Lu S, Yang Y, Zhao JM, Liu R, et al. Preliminary application of computer-assisted patient-specific acetabular navigational template for total hip arthroplasty in adult single development dysplasia of the hip. Int J Med Robot Comput Assist Surg. 2011;7(4):469–74.

43. Colquhoun D. An investigation of the false discovery rate and the misinterpretation of p-values. R Soc Open Sci [Internet]. 2014;1(3):140216–140216. Available from: http://rsos.royalsocietypublishing.org/cgi/doi/10.1098/rsos.140216

44. Ioannidis JPA. Why most published research findings are false. Vol. 2, PLoS Medicine. 2005. p. 0696–701.

45. Shafiee A, Atala A. Printing Technologies for Medical Applications. Trends Mol Med. 2016;22(3):254–65.

FIGURE LEGENDS

Figure 1. Flow chart of the selection and sorting method

Figure 2. Number of studies per level of evidence given by medical field. Levels of evidence are colour coded and fields are visually separated by alternating between white and grey columns.

For peer review only

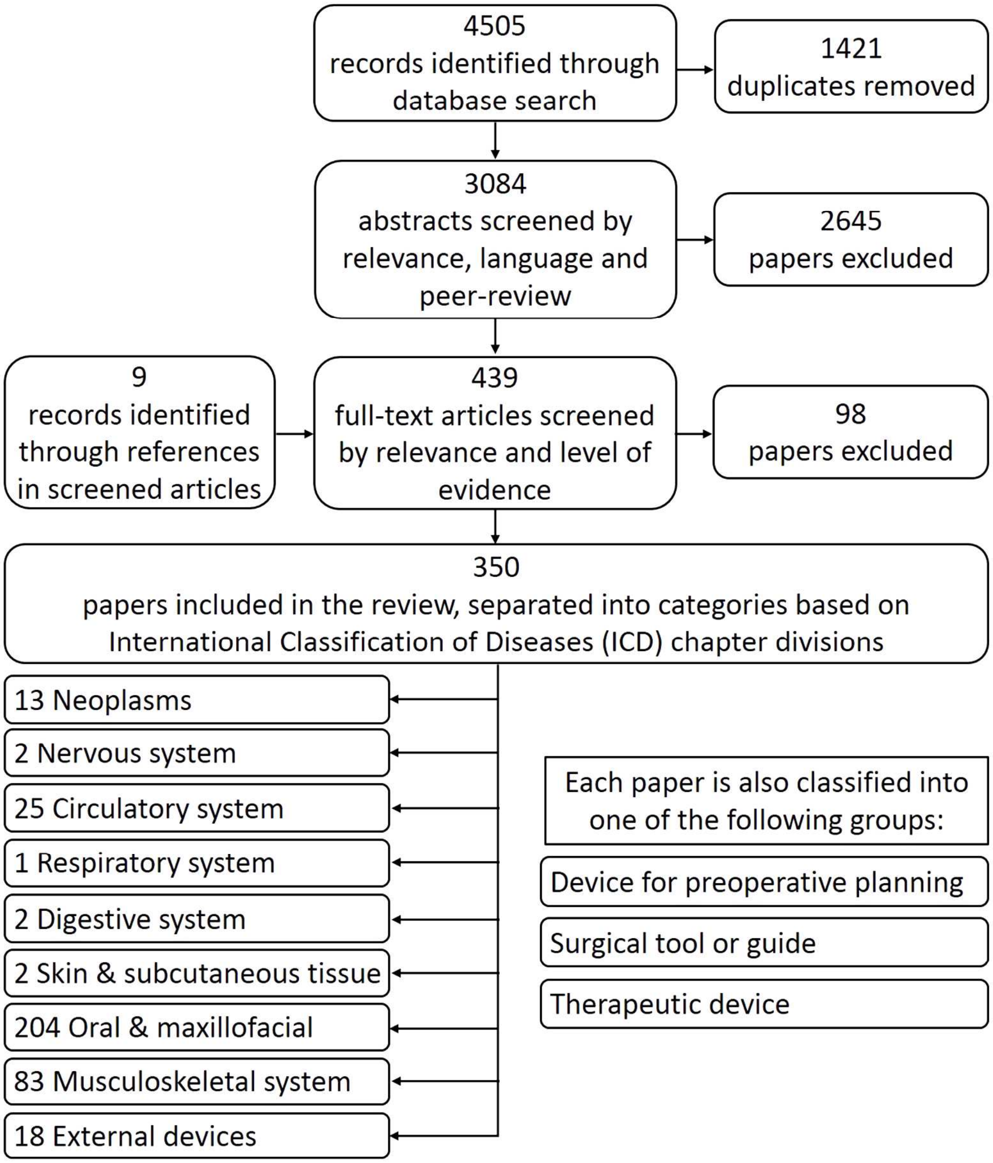

Figure 1. Flow chart of the selection and sorting method.

226x266mm (300 x 300 DPI)

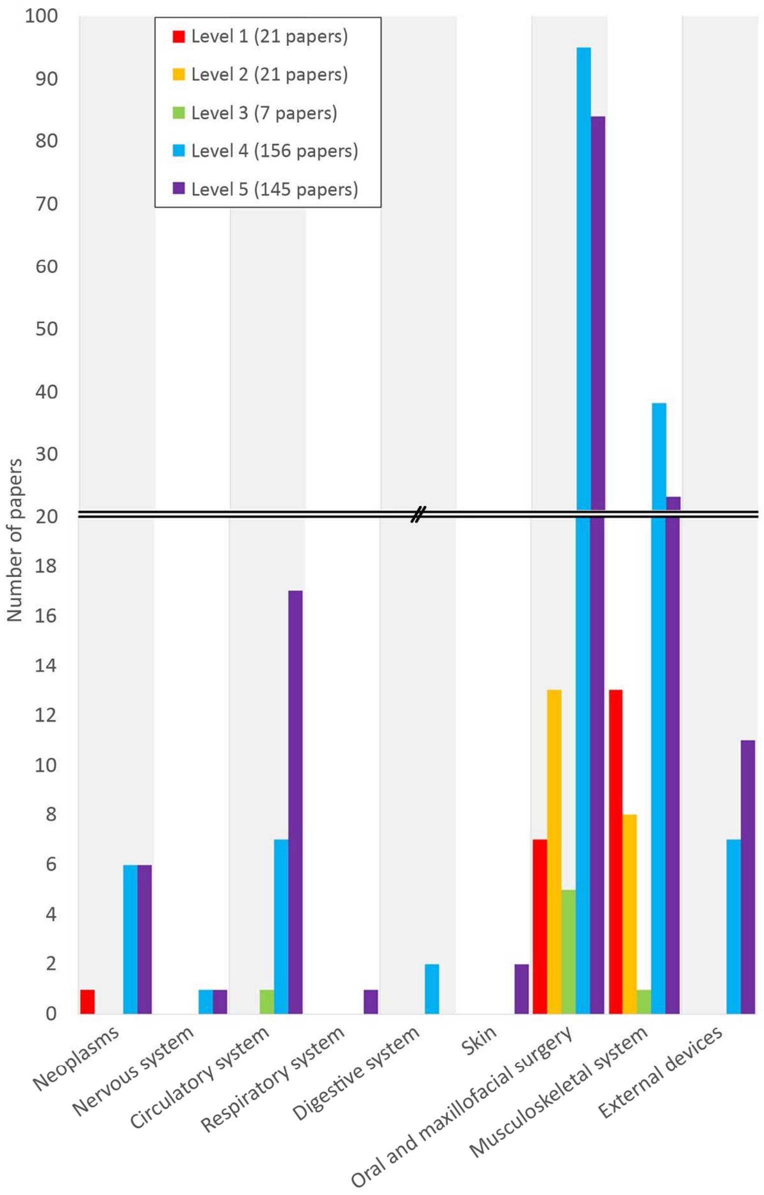

Figure 2. Number of studies per level of evidence given by medical field. Levels of evidence are colour coded and fields are visually separated by alternating between white and grey columns.

164x246mm (300 x 300 DPI)

1. Database search

IEEE Xplore and Google Scholar required a simplified search.

Database	PubMed (All article types)	ISI Web of Science (Article, review, case report, clinical trial, book or report)	OVID (Journal OR book)	IEEE Xplore (All article types)	Google Scholar (Exclude patents and citations)
Search Term	"**health care evaluation mechanisms**" OR "randomized controlled trial" OR "randomised controlled trial" OR "clinical trial" OR (efficacy OR effectiveness OR validation OR validity OR feasibility OR cohort OR observational OR longitudinal OR retrospective OR comparative OR "exploratory study" OR "case-control study" OR "case study" AND (clinical OR medical OR hospital OR biomedical OR assistive OR "health care" OR patients OR patient)) AND ("**Printing, Three-Dimensional**" OR "Three Dimensional Printing" OR "Three-Dimensional Printing" OR "3 Dimensional Printing" OR "3D Printing" OR "3-D Printing" OR stereolithography OR "laser melting" OR "electron beam melting" OR "fused deposition modeling" OR "fused deposition modelling" OR "rapid manufacturing" OR "rapid prototyping" OR "layered manufacturing" OR "digital manufacturing" OR bioprinting OR "3D prototyping" OR "3D fabrication" OR "rapid fabrication" OR "freeform fabrication")			randomized controlled trial OR "clinical trial" OR (efficacy OR validation OR feasibility OR "case study" AND (clinical OR medical OR hospital OR assistive OR "health care")) AND ("Three Dimensional Printing" OR "3D Printing" OR "rapid prototyping" OR "additive manufacturing")	"randomized controlled" OR "clinical trial" efficacy OR validation OR feasibility OR "case study" clinical OR medical OR hospital OR assistive OR "health care" "Three Dimensional Printing" OR "3D Printing" OR "rapid prototyping" OR "additive manufacturing"
**Number of studies**	913	700	1158	754	980

Number of studies: 4505

2. Duplicate removal

The first four databases were combined, then relevant additional results from Google Scholar were added.

Number of studies: 3084

For peer review only - http://bmjopen.bmj.com/site/about/guidelines.xhtml

Medical sector	Level of evidence	First author	Date	Device	Category	No. of participants	Participant details	Title	Aim	Outcomes	1. Clear hypothesis/aim/objective	2. Clear outcome measures	3. Patient characteristics described	4. Interventions clearly described	5. Distributions of confounders described	6. Findings clearly described	7. Estimates given of random variability	8. Adverse events reported	9. Patients lost to follow-up described	10. Probability values reported	11. Recruitment pool represents population	12. Participants represent population	13. Staff/places/facilities match std. treatment	14. Participants blinded to intervention	15. Those measuring outcomes blinded	16. Data dredging reported	17. Adjusted for different lengths of follow-up	18. Appropriate statistical tests	19. Reliable compliance with intervention	20. Accurate/reliable outcome measures	21. Groups recruited from same population	22. Groups recruited over same timeframe	23. Subjects randomised into intervention	24. Randomised intervention concealed	25. Adjustment for confounding	26. Losses to follow-up accounted for	27. Sufficient power
Neoplasms	1	de Farias	2014	Mandible model	P	37 (Test=17, Control=20)	Age: 9-74 years (Test mean=39.57 years, Control mean=53.11 years) Test=14 male, 3 female, Control=15 male, 5 female	Use of prototyping in preoperative planning for patients with head and neck tumors	Aim: to evaluate the efficacy of using AM models to plan head and neck surgeries.	+ The average reconstruction time decreased; Test=43.7 minutes, Control=127.7 minutes, p=0.001). Though significance was not given, the size of the bone flap taken for reconstruction was typically smaller, and the aesthetic results better in the test group.	⊞	⊞	⊞	⊞	⊞	⊞	⊞	⊞	X	⊞	?	?	⊞	⊞	⊞	⊞	X	⊞	⊞	⊞	⊞	⊞	⊞	⊞	?	X	⊞
Oral and Maxillofacial	1	Ahrberg	2015	All-ceramic zirconia crowns	T	25 (Same participants used for test and control)	Age: >18 years 10 male, 15 female	Evaluation of fit and efficiency of CAD/CAM fabricated all-ceramic restorations based on direct and indirect digitalization: a double-blinded, randomized clinical trial.	Aim: to evaluate the marginal and internal fit of AM fabricated zirconia crowns and three-unit fixed dental prostheses resulting from direct versus indirect digitalization.	+ The mean for the marginal gap was significantly (p<0.05) smaller 61.08 μm (±24.77 μm) for the test group compared with the control 70.40 μm (±28.87 μm). The test group showed significantly lower values of internal fit at the centro-occlusal site.	⊞	⊞	⊞	⊞	⊞	⊞	⊞	⊟	X	⊞	?	?	⊞	⊞	⊞	⊞	X	⊞	⊞	⊞	⊞	⊞	⊞	⊞	⊞	X	⊞
Oral and Maxillofacial	1	D'Urso	1999a	Cranio-maxillofacial model	P	30 (Test=15, Control=15)	No information provided	Stereolithographic biomodelling in cranio-maxillofacial surgery: a prospective trial	Aim: Assess whether biomodels in addition to standard imaging have greater utility in the surgery performed than the standard imaging alone.	+ Biomodels significantly improved operative planning, diagnosis and measurement accuracy. Operating time was also reduced by an estimated 17.63%.	⊞	⊞	⊞	⊞	⊞	⊞	⊞	⊟	X	⊟	?	?	⊞	⊟	⊟	⊞	X	⊞	⊞	⊞	⊞	⊞	⊞	?	⊟	X	⊞
Oral and Maxillofacial	1	Al-Ahmad	2013	Surgical guide for sagittal splitting ramus osteotomy	S	8 (One side used as test, the other as control)	Age: 18-30 years (mean=23 years)	Evaluation of an innovative computer-assisted sagittal split ramus osteotomy to reduce neurosensory alterations following orthognathic surgery: a pilot study	Aim: to evaluate the effectiveness of SSRO in reducing the incidence and severity of neurosensory alterations, using an AM surgical guide.	+ The test patients had lower postoperative abnormal thresholds for the Semmes–Weinstein monofilaments on lower lip and chin (p<0.05 at 3 months) and for the two-point discrimination on lower lip (p<0.05 at 1week) and chin (p<0.05 at 6 months), with fewer abnormal self-reported changes in lower lip sensation (p<0.05 at 1week) after surgery.	⊞	⊞	⊞	⊞	X	⊞	⊞	⊟	⊞	⊞	?	?	⊞	⊞	⊞	⊞	⊞	⊞	⊞	⊞	⊞	⊞	⊞	⊞	⊞	⊞	⊞
Oral and Maxillofacial	1	Ayoub	2014	Guide for mandibular reconstruction	S	20 (Test=10, Control=10)	Age: Test=52.3 ± 19.2 years, Control=54.7 ± 14.2 years) Test=4 male, 6 female, Control=6 male, 4 female	Evaluation of computer-assisted mandibular reconstruction with vascularized iliac crest bone graft compared to conventional surgery: a randomized prospective clinical trial	Aim: to evaluate the benefits of computer-assisted mandibular reconstruction with iliac crest bone grafts regarding the intraoperative time for transplant shaping, ischemia, duration of surgery, amount of bone removed and the hange in postoperative condyle position compared to conventional surgery.	+ Computer-assisted surgery shortened the time of transplant ischemia (p<0.005) and defect reconstruction (p<0.001) compared to conventional surgery. There was less bone harvested in the test group (p<0.001), and the intercondylar distance before compared to after surgery was less affected in the computer-assisted than in the conventional group (p<0.001).	⊞	⊞	⊞	⊞	⊟	⊞	⊞	⊟	X	⊟	?	?	⊞	⊟	⊟	⊞	X	⊞	⊞	⊞	⊞	⊞	⊞	?	⊟	X	⊞
Oral and Maxillofacial	1	Goh	2015	Polycaprolactone scaffold for ridge preservation	T	13 (Test=6, Control=7)	Age: 29–60 years (mean=46.8 years) 7 male, 7 female (1 was lost to follow-up, so was excluded from results)	Novel 3D polycaprolactone scaffold for ridge preservation – a pilot randomised controlled clinical trial	Aim: to evaluate the feasibility and effectiveness of using a polycaprolactone scaffold in fresh extraction sockets for ridge preservation.	+ The test group showed less vertical ridge resorption (significant in the mesio-buccal aspect: p = 0.008), with better maintenance of ridge height after 6 months.	⊞	⊞	⊞	⊞	⊟	⊞	⊞	⊟	⊞	⊞	?	?	⊞	⊟	⊞	⊞	⊞	⊞	⊞	⊞	⊞	⊞	⊞	?	⊟	⊞	⊞
Oral and Maxillofacial	1	Van de Velde	2010	Maxilla surgical guide	S	13 (Test=36 tooth implants, Control=34 tooth implants)	Age: 39-75 years (mean =55.7 years) 4 male, 9 female	The clinical and radiographic outcome of implants placed in the posterior maxilla with a guided flapless approach and immediately restored with a provisional rehabilitation: a randomized clinical trial	Aim: to compare the outcome of dental implants placed using a flapless protocol with an AM guide and immediate loading with a conventional protocol and loading.	+ Implants can successfully integrate in the posterior maxilla using a flapless approach with immediate loading. The mucosal tissues around implants changed significantly less compared with controls (p<0.05). Marginal bone levels were not significantly different between test and control implants. Opinion about speech, function, aesthetics and self-confidence was significantly better for the test side after 1 week (p<0.05).	⊞	⊞	⊞	⊞	⊟	⊞	⊞	⊞	⊞	⊞	?	?	⊞	⊟	⊞	⊞	⊞	⊞	⊞	⊞	⊞	⊞	⊞	?	⊟	⊞	⊞
Oral and Maxillofacial	1	Vercruyssen	2014	Surgical guides for edentulous jaws	S	59 (72 jaws)	Age:31-78 years 31 male jaws, 41 female jaws	A randomized clinical trial comparing guided implant surgery (bone- or mucosasupported) with mental navigation or the use of a pilot-drill template	Aim: To assess the accuracy of guided surgery compared to mental navigation or the use of a surgical template, in fully edentulous jaws.	+ Significantly lower mean deviation at the entry point, at the apex and angular deviation when using the AM guiding systems than when using mental navigation or a conventional surgical template (p<0.05). Differences between bone and mucosa support or type of guidance were negligible.	⊞	⊞	⊞	⊞	⊟	⊞	⊞	⊞	⊞	⊟	?	?	⊞	⊟	⊞	⊞	⊞	⊞	⊞	⊞	⊞	⊞	⊞	?	⊟	⊞	⊞
Musculoskeletal	1	Chareancholvanich	2013	Cutting guides for total knee replacement	S	80 (Test=40, Control=40)	Age: 55-84 years (mean: 69.5 years) 6 male, 34 female	A prospective randomised controlled study of patient-specific cutting guides compared with conventional instrumentation in total knee replacement	Aim: to compare patient-specific cutting guides with conventional instrumentation in total knee replacement.	+ The tibial component in the test group was closer to neutral alignment (p = 0.030), but the size of the difference was small (89.8°±1.2° vs 90.5°±1.6°). The AM guides also shortened the bone-cutting time by a 3.6 minutes (p < 0.001) and the operating time by 5.1 minutes (p = 0.019), without differences in post-operative blood loss (p = 0.528) or need for blood transfusion (p = 0.789). These minimal advantages of the AM guides are unlikely to be clinically relevant.	⊞	⊞	⊞	⊞	⊟	⊞	⊞	⊞	⊞	⊞	?	?	⊞	⊟	⊞	⊞	⊟	⊞	⊞	⊞	⊞	⊞	⊞	?	⊟	⊞	⊞
Musculoskeletal	1	Chen	2015	Guide plate for pedicle screw fixation	S	43 (Test=20, Control=23)	Age: Test=35–70 years (mean=52.3 years) Control=37–72 years (mean=55.4 years) Test=9 male, 11 female Control=12 male, 11 female	Clinical Use of 3D Printing Guide Plate in Posterior Lumbar Pedicle Screw Fixation.	Aim: to evaluate the clinical efficacy of use of a 3D printing guide plate in posterior lumbar pedicle screw fixation.	+ The time of placement for each screw was shorter, the amount of hemorrhage was less, and the fluoroscopy times of each screw placement was lower in the test group than in the control group (all p<0.05). The excellent and good screw placement rate was 100% in the test group and 98.4% in the control group, without any statistical difference (p>0.05). No obvious complications were reported in either group.	⊞	⊞	⊞	⊞	⊞	⊞	⊞	⊞	⊞	⊟	?	?	⊞	⊞	⊟	⊞	⊞	⊞	⊞	⊞	⊞	⊞	⊞	⊞	⊞	⊞	⊞
Musculoskeletal	1	Du	2013	Templates to aid in pin placement in hip resurfacing arthroplasty	S	34 (Test-16, Control=18)	Ages: 37-55 years	Use of patient-specific templates in hip resurfacing arthroplasty: experience from sixteen cases	Aim: To assess whether AM patient-specific templates aid in accurate intraoperative pin placement.	+ The prosthesis stem shaft angle was greater for the test group (p=0.001). The locating template can provide precise and dependable location for hip resurfacing femoral components during arthroplasty and ensure the valgus stem placement necessary for optimal outcomes.	⊞	⊞	⊟	⊟	⊞	⊞	⊞	⊟	⊞	⊞	?	?	⊞	⊟	⊟	⊞	⊟	⊞	⊞	⊞	⊞	⊞	⊞	?	⊟	⊞	⊞
Musculoskeletal	1	Gan	2015	Navigational template for total knee arthroplasty	S	70 (Test=35, Control=35)	Age: Test= 63-75 years (mean=68.5±4.8 years), Control=64-72 years (mean=67.8±3.4 years) Test=10 male, 25 female, Control=9 male, 26 female	Accuracy and efficacy of osteotomy in total knee arthroplasty with patient-specific navigational template	Aim: to validates a novel patient-specific navigational template for total knee arthroplasty.	+ The AM navigational template significantly reduced errors, operation time and blood loss (p<0.05).	⊞	⊞	⊞	⊞	⊞	⊞	⊞	⊞	X	⊞	?	?	⊞	⊟	⊞	⊞	X	⊞	⊞	⊞	⊞	⊞	⊞	?	⊟	X	⊞
Musculoskeletal	1	Hendel	2012	Guide for determining glenoid component position	S	31 (Test=15, Control=16)	No information provided	Comparison of Patient-Specific Instruments with Standard Surgical Instruments in Determining Glenoid Cmponent Position	Aim: to compare patient-specific instruments with standard surgical instruments in determining glenoid component position.	+ The AM technology significantly decreased (p < 0.05) the average deviation of implant position for inclination and medial-lateral offset. The greatest benefit was observed in patients with retroversion >16˚ (p < 0.001).	⊞	⊞	⊟	⊞	⊟	⊞	⊞	⊞	⊟	⊞	⊞	⊞	⊞	⊟	⊞	⊞	?	⊞	⊞	⊞	⊞	⊞	⊞	⊟	⊟	?	⊞
Musculoskeletal	1	Maini	2016	Plate for acetabulum fracture fixation	P	21 (Test=10, Control=11)	Age: 18-60 years (mean=38.7 years) Test = 9 male, 1 female Control = 9 male, 2 female	Three-dimensional printing and patient-specific pre-contoured plate: future of acetabulum fracture fixation?	Aim: to evaluate the accuracy of patient-specific pre-contoured plates.	+ Reduction achieved, as evaluated by CT scan, was more in the test group than the control group (p<0.05). Reduced blood loss (100 ml less) and surgical time (12 min less) were observed in the test group but the results were not significant (p>0.05). All the pre-contoured plates fitted well to the pelvis intra-operatively.	⊞	⊞	⊞	⊞	⊟	⊞	⊞	⊞	X	⊞	?	?	⊞	⊟	⊟	⊞	X	⊞	⊞	⊞	⊞	⊞	⊞	?	⊟	X	⊞
Musculoskeletal	1	Merc	2013	drill guide for screw placement	S	19 (Test=9 (54 screws), Control=10(54 screws))	Age: Test=59 ± 5 years, Control=62 ±12 years Test=4 male, 5 female, Control=5 male, 5 female	A multi-level rapid prototyping drill guide template reduces the perforation risk of pedicle screw placement in the lumbar and sacral spine	Aim: to evaluate the accuracy of a multi-level drill guide template for lumbar and first sacral pedicle screw placement and to compare it with the freehand technique under fluoroscopy supervision.	+ The incidence of cortex perforation was significantly reduced in the test group. So was the deviation and displacement level of screws in the sagital plane but not in the transverse plane.	⊞	⊞	⊞	⊞	⊞	⊞	⊞	⊞	X	⊞	⊞	⊞	⊞	⊟	⊟	⊞	X	⊞	⊞	⊞	⊞	⊞	⊞	?	⊟	X	⊞
Musculoskeletal	1	Shuang	2016	Osteosynthesis Plates	T	13 (Test=6, Control=7)	Age: Test=31-62 years (mean=46.2 years) Control=27-59 years (mean=40.3 years) Test= 4 male, 2 female, Control=6 male, 1 female	Treatment of Intercondylar Humeral Fractures With 3D-Printed Osteosynthesis Plates.	Aim: to evaluate the efficacy custom AM osteosynthesis plates in the treatment of intercondylar humeral fractures.	+ Operative time was shorter for the test group than the control group (p=0.026). The mean time to bone union was 3.4 months. The 2 groups showed no significant difference in the rate of patients with excellent or good elbow function, or in the ranges of elbow flexion/extension and pronation/supination (p>0.05).	⊞	⊞	⊟	⊞	⊞	⊞	⊞	⊞	⊞	⊞	?	?	⊞	⊟	⊟	⊞	?	⊞	⊞	⊞	⊞	⊞	⊞	⊞	⊞	⊞	⊞
Musculoskeletal	1	Stephens	2016	Bone models	P	58 (Test=29, Control=29)	Age: >60 years (mean=73 years) 5 male, 53 female	3-D bone models to improve treatment initiation among patients with osteoporosis: A randomised controlled pilot trial.	Aim: to investigate the efficacy of AM bone models as a tool to facilitate initiation of bisphosphonate treatment among individuals who are newly diagnosed with osteoporosis.	+ The test group were more emotionally affected by osteoporosis immediately after the interview (p=0.04) and reported a greater understanding of osteoporosis at follow-up (p=0.04), than the control group.	⊞	⊞	⊞	⊞	⊟	⊞	⊞	⊟	⊞	⊞	?	?	?	⊞	⊞	⊞	⊞	⊞	⊞	⊞	⊞	⊞	⊞	⊞	⊟	⊞	⊞
Musculoskeletal	1	Wu	2011	Spine model	P	62 (Test=34, Control=28) 677 screws inserted	Age: 4-22 years (mean=11 years)	Accuracy and Safety Assessment of Pedicle Screw Placement Using the Rapid Prototyping Technique in Severe Congenital Scoliosis	Aim: to compare the accuracy and safety of pedicle screw placement in congenital scoliosis using the AM technique versus conventional fluoroscopy.	+ Accuracy of placement = 89.7% (84.7% in control group; 93.5% in test group). Compared with the fluoroscopy method, the AM assisted technique had a shorter operation time and higher scoliosis correction rate (p<0.05).	⊞	⊞	⊟	⊞	⊟	⊞	⊞	⊞	⊞	⊞	⊞	⊞	⊞	⊟	⊟	⊞	⊟	⊞	⊞	⊞	⊞	⊞	⊞	?	⊟	?	⊞
Musculoskeletal	1	Yang	2016	Model of trimalleolar fracture	P	30 (Test=15, Control=15)	Age: 31-42 years (mean=36.5 years) 16 male, 14 female	Application of 3D Printing in the Surgical Planning of Trimalleolar Fracture and Doctor-Patient Communication.	Aim: to evaluate the effectiveness of using AM models for surgical planning in treating trimalleolar fractures and in physician-patient communication.	+ Both the operation time and the intraoperative blood loss were less in the test group than in the control group (p<0.05). Patient satisfaction with using the AM device for communication was scored highly.	⊞	⊞	⊟	⊞	⊟	⊞	⊞	⊞	X	⊞	?	?	⊞	⊟	⊟	⊞	X	⊞	⊞	⊞	⊞	⊞	⊞	?	⊟	X	⊞
Musculoskeletal	1	You	2016	Proximal humeral fracture model	P	66 (Test=34, Control=32)	61-76 years (mean for Test = 66.09 ± 4.09 years, mean for Control = 66.28 ± 4.10 years) 27 male, 39 female	Application of 3D printing technology on the treatment of complex proximal humeral fractures (Neer3-part and 4-part) in old people.	Aim: to investigate the feasibility and clinical potential of using AM models versus the conventional strategy (thin-layer CT scan) for the treatment of complicated proximal humeral fractures in old people.	+ Reduced surgery duration, less blood loss during surgery, and lower number of fluoroscopy can be observed in the test group compared with the control group (P<0.05). The results showed no significant difference in time to union between the two groups (P>0.05).	⊞	⊞	⊟	⊞	⊞	⊞	⊞	⊞	⊞	⊞	⊞	⊞	⊞	⊟	⊟	⊞	⊟	⊞	⊞	⊞	⊞	⊞	⊞	?	⊞	⊞	⊞
Musculoskeletal	1	Zhang	2011a	Acetabular navigational template	S	22 (Test=11, Control=11)	Age: Test=48.6 ± 6.8 years, Control=49.3 ± 4.9 years Test=7 male, 4 female, Control=5 male, 6 female	Preliminary application of computer-assisted patient-specific acetabular navigational template for total hip arthroplasty in adult single development dysplasia of the hip	Aim: to compare a patient-specific AM acetabular navigational template for total hip arthroplasty (THA) to conventional THA in adult single development dysplasia of the hip.	+ The test group showed smaller differences from the predetermined angles than the control group (p<0.05).	⊞	⊞	⊟	⊞	⊞	⊞	⊞	⊟	⊞	⊞	?	?	⊞	⊟	⊟	⊞	⊟	⊞	⊞	⊞	⊞	⊞	⊞	?	⊞	⊞	⊞
Oral and Maxillofacial	2	Arisan	2013	Bone and mucosa supported guides for dental implants	S	54 (Mucosa-supported=12, Bone-supported=15, Control=27)	Age: 28-74 years (mean=52.5 years) 23 male, 31 female	Implant Positioning Errors in Freehand and Computer-Aided Placement Methods: A Single-Blind Clinical Comparative Study	Aim: to compare the dental implant positioning errors related to the use of freehand and computer-aided treatment methods.	+ Interproximal emergence, insufficient interimplant distance, and improper parallelism errors were higher in implants placed using the freehand method (p <0.0001, p <.00001, and p <0.001 respectively).	⊞	⊞	⊞	⊞	⊞	⊞	⊞	⊟	⊞	⊞	?	?	⊞	⊟	⊞	⊞	⊞	⊞	⊞	⊞	⊞	⊞	⊟	X	⊞	⊞	⊞
Oral and Maxillofacial	2	Azuma	2014	Mandible model	S	28 (Test=12, Control=16)	Age: Test=34-91, Control=55-82 18 male, 10 female	Mandibular reconstruction using plates prebent to fit rapid prototyping 3-dimensional printing models ameliorates contour deformity	Aim: To evaluate the clinical efficacy and esthetic outcome of using preoperatively manufactured reconstruction plates, produced using AM models, in mandibular reconstruction.	+ The mandibular symmetry in patients receiving the AM-model-based prebent plates was better than that in patients receiving conventional reconstructive surgery (p<0.05).	⊞	⊞	⊞	⊞	⊟	⊞	⊞	⊟	⊟	⊟	?	?	⊞	⊟	⊟	⊞	⊟	⊞	⊞	⊞	?	?	⊟	X	⊟	⊟	⊞
Oral and Maxillofacial	2	Brown	2015	Orthodontic bracket system	T	96 (G1: Test=31, G2: Direct bonded conventional=33, G3: Indirect bonded conventional=32)	Age: G1=13.42 years, G2=3.58 years, G3=13.42 years G1 (AM) =17 male, 15 female G2=16 male, 15 female G3=17 male, 16 female	Effectiveness and efficiency of a CAD/CAM orthodontic bracket system.	Aim to retrospectively compare: patients who were indirectly bonded with CAD/CAM self-ligating appliances with those direct bonded with conventional self-ligating appliances, and those indirectly bonded with conventional self-ligating appliances.	+ No statistically significant difference was found on ABO Cast-Radiograph Evaluation scores among the 3 treatment groups (p=0.13). The mean treatment times were different (p<0.05, G1: 13.8 ± 3.4 months, G2: 21.9 ± 5.0 months; G3: 16.9 ± 4.1 months.	⊞	⊞	⊞	⊞	⊞	⊞	⊞	⊟	X	⊞	⊞	⊞	⊞	⊟	⊞	⊞	X	⊞	⊞	⊞	?	?	⊟	X	⊞	X	⊞
Oral and Maxillofacial	2	Calvo-Guirado	2011	Composite powder molding for bone grafts	P	20 (Test=10, Control=10)	No information provided	Calculation of bone graft volume using 3D reconstruction system	Aim: to validate the use of AM bioreplicas for calculating bone volumes prior to planning bone regeneration surgery.	NS No differences were recorded for measurements of bone defect between patient bone and the biological models (p>0.05) but there were also no differences between the test group and control group arising from post-surgical complications observed at the post-operatory fifteen and thirty day examinations.	⊞	⊞	⊟	⊞	⊟	⊞	⊞	⊟	⊞	⊟	?	?	⊞	⊟	⊟	⊞	⊞	⊞	⊞	⊞	⊞	?	?	?	?	⊞	⊟
Oral and Maxillofacial	2	Hanasono	2012	Guide for mandibular reconstruction	S	76 (Test=38, Control=38 defect-matched)	Age:Test=51.0 ± 14.4 years, Control=56.4 ± 15.1 years Test=26 male, 12 female Control=28 male, 10 female	Computer-Assisted Design and Rapid Prototype Modeling in Microvascular Mandible Reconstruction	Aim: to evaluate the use of AM modeling to improve the speed and accuracy of mandibular reconstruction.	+ The operative time for the test group was faster than the operative time defect-matched control group (p=0.0006). The change in position of selected bony landmarks was less in the test group than in the control group (p=0.001).	⊞	⊞	⊟	⊞	⊞	⊞	⊞	⊟	⊞	⊞	?	?	⊞	⊟	⊟	⊞	⊟	⊞	⊞	⊞	⊟	⊞	⊟	X	⊟	⊞	⊞
Oral and Maxillofacial	2	Inokoshi	2012	AM complete dentures	T	10 (Same participants used for test and control)	Age: 78.1 ± 5.6 years	Evaluation of a complete denture trial method applying rapid prototyping	Aim: to compare an AM method for complete dentures with the conventional method.	NS Prosthotontists rated esthetics and stability lower for the AM method (p<0.05), but chair time as faster (p<0.01). No significant differences were found between the two methods in regards to patient scores.	⊞	⊞	⊞	⊞	⊟	⊞	⊞	⊟	X	⊟	?	?	?	⊟	⊟	⊞	X	⊞	⊞	⊟	X	X	⊟	X	⊟	X	⊞
Oral and Maxillofacial	2	Kozakiewicz	2011	Model for developing patient-specific orbital wall implants	P	24 (Test=12, Control=12)	Age: Test=36 ± 10 years, Control=40 ± 20 years Test=9 male, 3 female, Control=9 male, 3 female	Treatment with individual orbital wall implants in humans - 1-Year ophthalmologic evaluation	Aim: to compare the functional results, 1 year after treatment, of using an AM reconstruction method for orbital wall repair with a conventional reconstruction method.	+ At 1 year follow up, the test group had better outcomes than the control group (diplopia: p=0.021, upgaze vertical visual disparity reduction: p=0.007)	⊞	⊞	⊞	⊞	⊟	⊞	⊟	⊟	⊞	⊞	⊞	⊞	⊞	⊟	⊟	⊞	⊞	⊞	⊞	⊞	⊞	⊟	⊟	X	⊟	⊞	⊞
Oral and Maxillofacial	2	Lee	2009	AM prefabricated prostheses for cranioplasty	T	131 (Autograft (G1)=91, conventional prosthesis (G2)=23, AM prefabricated prosthesis (G3)=17)	Age: G1=43.84 ± 17.91 years, G2=40.96 ± 15.48 years, G3=40.24 ± 11.97 years G1=62 male, 29 female, G2=14 male, 9 female, G3=12 male, 5 female	Cranioplasty using polymethyl methacrylate prostheses	Aim: to assess whether prefabricated AM prostheses outperform intra-operatively moulded prostheses and perform comparably to the gold-standard of autograft bone flaps in reducing operating time, blood loss and surgical complications in elective delayed cranioplasty operations, after decompressive craniectomy, to repair large (> 100 cm2) cranial defects.	+ G3 patients required less operating time than G2 patients (p<0.001), and operating time did not differ significantly between G1 and G3 (p>0.05). Intra-operative blood loss was lower for G3 than G2 (p=0.015) and did not differ significantly between G1 and G3 (p>0.05). The infection rate for G3 was lower than for G2 and was comparable to G1.	⊞	⊞	⊞	⊞	⊟	⊞	⊞	⊟	⊟	⊞	⊞	⊞	⊞	⊞	⊞	⊞	⊟	⊞	⊞	⊞	⊞	⊞	?	⊟	?	?	⊞
Oral and Maxillofacial	2	Modabber	2012a	Guide for jaw reconstruction	S	10 (Test=5, Control=5)	Age: 37-74 years (mean=52.4 years) 8 male, 2 female	Evaluation of computer-assisted jaw reconstruction with free vascularized fibular flap compared to conventional surgery: a clinical pilot study	Aim: to assess whether the AM method has an influence on the duration of the surgery and the amount of bone removed in fibular reconstruction of maxillary or mandibular defects.	+ Shaping procedure and ischaemic time was shorter for the test group than the control (p=0.014). There was no change between the defect size of the fibula and the necessary transplant size for the test group, but a mean change of 1.92cm occured for the control (p=0.001).	⊞	⊞	⊟	⊞	⊟	⊞	⊞	⊞	X	⊞	?	?	⊞	⊟	⊟	⊞	X	⊞	⊞	⊞	⊞	⊞	⊞	?	⊟	X	⊞
Oral and Maxillofacial	2	Resnick	2016	Surgical splint for orthognathic surgery	T	43 - All had Le Fort I osteotomy and bilateral sagittal split osteotomy (19 in G1: facial symmetry, single-segment, 17 in G2: facial asymmetry, single-segment, and 7 in G3: multi-segment).	All groups compared AM to manual splint fabrication. No information provided on age or gender.	Is There a Difference in Cost Between Standard and Virtual Surgical Planning for Orthognathic Surgery?	Aim: to retrospectively compare time and cost differences between virtual surgical planning and 3D printed orthognathic splints with conventional planning and manufacture techniques.	+ The average times and costs were 194 ± 14.1 minutes and $2,765.94, respectively, for VSP and 540.9 ± 99.5 minutes and $3,519.18, respectively, for standard planning. All time and cost differences were statistically significant (p<0.001).	⊞	⊞	⊞	⊞	⊟	⊞	⊞	⊟	X	⊞	?	?	⊞	⊟	⊟	X	X	⊞	⊞	⊞	⊟	⊟	?	X	⊟	X	⊞
Oral and Maxillofacial	2	Rogers-Vizena	2016	Craniofacial model	P	29 (Test=9, Control=20)	Age: Test=11.4 ± 3.0 years, Control=11.1 ± 4.7 years Test=4 male, 5 female Control=11 male, 9 female	Cost-Benefit Analysis of Three-Dimensional Craniofacial Models for Midfacial Distraction: A Pilot Study.	Aim: to retrospectively assess the benefit of AM models used in planning surgeries in regards to safety, operative time and cost.	NS 7 complications occurred in 6 of the control group, whereas none occurred in the test group. The test group had a 31.3 minute (7.8%) reduction in operative time (not significant), and time-based cost savings were $1036.	⊞	⊞	⊟	⊞	⊟	⊞	⊞	⊞	X	⊞	?	?	⊞	⊟	⊟	X	X	⊞	⊞	⊞	?	?	⊟	X	⊟	X	⊟
Oral and Maxillofacial	2	Sumida	2015	membranes for bone augmentation in implant treatment	T	26 (Test=13, Control=13)	Age: Test=48.6 ± 9.4 years, Control= 47.8 ± 8.8 years Test=4 male, 9 female, Control=3 male, 10 female	Custom-made titanium devices as membranes for bone augmentation in implant treatment: Clinical application and the comparison with conventional titanium mesh	Aim: to compare titanium AM devices with conventional methods for reconstructing alveolar bone for implantation.	+ There was no significant difference between groups in occurance of mucosal rupture (p=0.27). However, the operation time was shorter for the test group (p<0.01), and fewer screws were used to retain the membrane than the control group (p<0.01).	⊞	⊞	⊟	⊟	⊞	⊞	⊞	⊟	X	⊞	?	?	?	⊟	⊟	?	X	⊞	⊞	⊞	?	?	?	X	⊟	X	⊞
Oral and Maxillofacial	2	Zhang	2011b	Titanium plate to guide temporomandibular joint reconstruction	S	35 (Test=11, Control=24)	Age: Test=14-65 years (mean=42.3 years), Control=24-56 years (mean=34.9) Test=5 male, 6 female, Control=14 male, 10 female	Application of Rapid Prototyping for Temporomandibular Joint Reconstruction	Aim: to compare the operative time between temporomandibular joint reconstruction surgery using AM guides and conventional surgery.	+ Operative time was shorter for the test group than the control (p<0.05). The outcome of the surgeries was good, with the condyle-incisor, condyle–mental foramen, and condyle-angle from the postoperative CT scan showing no significant difference between the 2 sides (p>0.05).	⊟	⊞	⊟	⊞	⊟	⊟	⊟	⊟	⊞	⊟	⊞	⊞	⊞	⊟	⊟	⊞	?	⊞	⊞	⊞	⊟	⊟	⊟	X	⊟	⊞	⊞
Musculoskeletal	2	Hirao	2014	Guide for total ankle arthroplasty	S	38 (Test=24, Control=14)	No information provided	Use of a Custom-made Surgical Guide in Total Ankle Arthroplasty in Rheumatoid Arthritis Cases	Aim: to retrospectively compare the accuracy and stability of bone cutting for total ankle arthroplasty when using AM surgical guides to using conventional guides.	+ The error angle of the tibial bone cut was smaller (p = 0.02), and the tilting angle between components was smaller in the test group than the control group (P = 0.01).	⊟	⊞	⊟	⊞	⊟	⊞	⊞	⊞	⊞	⊞	?	?	⊞	⊟	⊟	?	⊞	⊞	⊞	⊞	?	?	⊟	X	⊟	⊞	⊞
Musculoskeletal	2	Iannotti	2015	Template for glenoid implant positioning	P	46 (Test=25, Control=21)	No information provided	Three-Dimensional Imaging and Templating Improve Glenoid Implant Positioning	Aim: to assess whether 3D imaging and the use of patient-specific instrumentation results in more accurate placement of the glenoid component than 3D imaging and standard instrumentation.	NS There was no significant difference between the test group and the control group in achieving the desired implant position, but both performed better than a retrospective sample that used 2D imaging with standard instrumentation.	⊞	⊞	⊟	⊞	⊟	⊞	⊞	⊟	X	⊞	?	?	⊞	⊟	⊞	⊞	X	⊞	⊞	⊞	⊟	⊟	⊟	?	⊟	X	⊞
Musculoskeletal	2	Karlin	2016	Spine model	P	17 (Test=7, Control =10)	Age: Test = 5-14 years (mean=10) Control = 11-18 years (mean=13) Test = 3 male, 4 female Control = 4 male, 6 female	The surgical treatment of spinal deformity in children with myelomeningocele: the role of personalized three-dimensional printed models.	Aim: to retrospectively review children for whom AM spine models were used for surgical planning (Test) and compare them through subjective and objective criteria to a similar group that had no models (Control).	NS The models improved planning and performance of patient-specific spinal instrumentation that was secure and low profile. The efficiency of the surgery as measured by intraoperative fluoroscopy time and blood loss and the extent of the deformity correction was comparable or superior in the test group.	⊞	⊞	⊟	⊟	⊟	⊞	⊟	⊟	X	⊟	?	?	⊞	⊟	⊟	?	X	⊞	⊞	⊞	⊟	?	⊟	X	⊟	X	⊟
Musculoskeletal	2	Li	2015a	Lumbar vertebral model	P	37 (Test=15, Control=22)	Age: Test=52.36 ± 4.57 years, Control= 50.83 ± 3.60 years Test=9 male, 6 female, Control= 15 male, 7 female	Application of the polystyrene model made by 3-D printing rapid prototyping technology for operation planning in revision lumbar discectomy	Aim: to evaluate the effectiveness of 3-D rapid prototyping technology in revision lumbar discectomy.	+ The test group had a shorter operation time (p<0.001) and less blood loss (p<0.001) than the control group. There was no difference between groups for complication rate or clinical metrics.	⊞	⊞	⊞	⊞	⊞	⊞	⊞	⊞	⊞	⊞	?	?	⊞	⊟	⊟	⊞	⊟	⊞	⊞	⊞	?	⊞	⊟	X	⊞	⊞	⊞
Musculoskeletal	2	Li	2016a	Model of pelvis and pelvic fracture	P	157 (Test=81, Control=76)	Age: Test=34.0 ± 8.2 years, Control = 32.2 ± 9.4 years Test=55 male, 26 female, Control=51 male, 25 female	Comparative use of the computer-aided angiography and rapid prototyping technology versus conventional imaging in the management of the Tile C pelvic fractures	Aim to compare the two groups on length of surgical procedure, intra-operative complications, intra- and postoperative blood loss, postoperative pain, postoperative nausea and vomiting (PONV), length of stay, and type of discharge.	+ The test group experienced shorter surgery time (p=0.035), less blood loss (31.0±8.2 g/L vs. 36.2±7.4 g/L), less pain (2.5±2.3 NRS score vs. 2.8±2.0 NRS score), and shorter recoveries (7.8±2.0 days vs. 10.2±3.1 days before discharge).	⊞	⊞	⊞	⊞	⊞	⊞	⊞	⊞	X	⊞	⊞	⊞	⊞	⊟	⊟	X	X	⊞	⊞	⊞	⊞	⊞	⊟	X	⊞	X	⊞
Musculoskeletal	2	Schweizer	2016	Guide for Reconstructions of Scaphoid Fractures	S	22 (Test=9, Control=13)	Age: Test = 15-47 years (mean=27) Control=14-41 years (mean=24) All male	Computer-Assisted 3-Dimensional Reconstructions of Scaphoid Fractures and Nonunions With and Without the Use of Patient-Specific Guides: Early Clinical Outcomes and Postoperative Assessments of Reconstruction Accuracy.	Aim: to assess the accuracy of the reduction of surgically reconstructed scaphoid nonunions or fractures using 3-dimensional computer-based planning with and without patient-specific guides.	+ In the test group, average residual displacement was 7˚ after surgery, and 8 of 9 scaphoids healed after 2 to 6 months, with partial nonunion after 9 months observed in one patient. In the control group, residual displacement after surgery was 26˚, and 11 of 13 scaphoids healed between 2 and 34 months. 2 scaphoids in the control did not consolidate. The difference in the accuracy of reconstruction was significant (p=0.001). Average operation time was shorter (p=0.01) for the test group.	⊞	⊞	⊟	⊞	⊟	⊞	⊞	⊞	⊞	⊞	?	?	⊞	⊟	⊟	⊞	⊟	⊞	⊞	⊞	?	?	⊟	X	⊟	⊞	⊞
Musculoskeletal	2	Yang	2014	Upper cervical spine model and navigation template	S	49 (Test=21, Control=28)	Age: 5-81 years (mean=37.6 years) 21 male, 28 female	Clinical Application of Computer-aided Design-Rapid Prototyping in C1–C2 Operation Techniques for Complex Atlantoaxial Instability	Aim: to evaluate the clinical efficacy of AM models in surgical treatments for atlantoaxial instability.	+ The test group had lower intraoperative fluoroscopy frequency (p<0.001), operation time (posterior p=0.001, anterior p=0.004) and blood loss for the posterior approach (p=0.006) than the control group. The screw malposition rate showed no difference between the two groups.	⊞	⊞	⊞	⊞	⊞	⊞	⊞	⊞	⊞	⊞	⊞	⊞	⊞	⊟	⊟	⊞	⊞	⊞	⊞	⊞	?	?	⊟	X	⊟	⊞	⊞
Musculoskeletal	2	Yang	2015	Spine model	P	126 (Test-50, Control=76)	Age: Test=12.52 ± 5.45 years, Control=12.11 ± 6.42 years Test=12 male, 38 female, Control=33 male, 43 female	Application of 3D Rapid Prototyping Technology in Posterior Corrective Surgery for Lenke 1 Adolescent Idiopathic Scoliosis Patients	Aim: to retrospectively evaluate the effectiveness of AM technology in corrective surgery for Lenke 1 adolescent idiopathic scoliosis (AIS) patients based on operation time, perioperative blood loss, transfusion volume, postoperative hemoglobin (Hb), postoperative complications, and length of hospital stay.	+ The test group had shorter operation time, less blood loss and transfusion volume, and higher postoperative Hb (all, P<0.001). However, no significant differences were observed in complication rate, length of hospital stay, and postoperative radiological outcomes between 2 groups (all, P>0.05).	⊞	⊞	⊞	⊞	⊞	⊞	⊞	⊞	⊞	⊞	?	?	⊞	⊟	⊟	⊞	?	⊞	⊞	⊞	?	⊞	⊟	X	⊟	⊞	⊞
Circulatory	3	Schievano	2007	Model of the right ventricular outflow tract and pulmonary trunk	P	12 (4 did not undergo percutaneous pulmonary valve implantation (PPVI), 4 successfully underwent PPVI, and 4 unsuccessfully underwent PPVI)	Age: 9-39 years (mean 20 years) 7 male, 5 female	Percutaneous Pulmonary Valve Implantation Based on Rapid Prototyping of Right Ventricular Outflow Tract and Pulmonary Trunk from MR Data	Aim: To retrospectively determine if magnetic resonance imaging data can be used to create rigid models that are accurate representations of the right ventricular outflow tract and pulmonary trunk anatomy and if such models can be used to refine the selection of patients for PPVI.	NS Both cardiologists correctly determined from the models that the 4 subjects who did not undergo PPVI were unsuitable for PPVI. Of the 8 subjects who underwent PPVI, only 4 were successful. Both cardiologists correctly identified the suitability of 5 of these subjects from the models. Therefore the cardiologists had a 75% accuracy when selecting patient suitability for PPVI using 3D models.	⊞	⊞	⊟	⊞	⊟	⊞	⊞	⊟	X	⊟	⊟	⊟	⊞	⊟	⊞	X	X	⊞	⊞	⊞	?	?	⊟	⊟	⊟	X	⊟
Oral and Maxillofacial	3	Adolphs	2014	Splint for orthognathic surgery	T	10 (Same participants used for test and control)	No information provided	RapidSplint: virtual splint generation for orthognathic surgery – results of a pilot series	Aim: to demonstrate that clinically usable splints for orthognathic surgery can be produced by computer-assisted technology.	NS 8 of 10 AM splints, and 6 out of conventional splints could be used clinically to achieve and maintain final occlusion after orthognathic surgery. In the five patients where both types of splints were available, their occlusal fitting was assessed as being equivalent, and in one case the AM splint showed better occlusal fitting than the traditional splint.	⊞	⊞	⊟	⊞	X	⊟	⊟	⊟	X	⊟	?	?	⊞	⊟	⊟	?	X	⊞	⊞	?	X	X	X	X	X	X	⊟
Oral and Maxillofacial	3	Arisan	2010	Dental screw placement guides	S	54 (28 guides in system I, 32 guides in system II)	Age: 28-73 years (mean=48.4 years) 16 male, 38 female	Accuracy of Two Stereolithographic Guide Systems for Computer-Aided Implant Placement: A Computed Tomography-Based Clinical Comparative Study	Aim: To compare the accuracy of two AM guide systems for dental implant placement.	+ Implants that were placed by bone-supported guides had higher deviations than implants that were placed by mucosa-supported guides (p<0.05) but there was no difference in deviation between implant brands (p <0.08).	⊞	⊞	⊞	⊞	⊞	⊞	⊞	⊟	⊟	⊞	?	?	⊞	⊟	⊟	⊞	⊟	⊞	⊞	⊞	⊞	?	⊟	X	⊟	⊞	⊞
Oral and Maxillofacial	3	Morris	2013	Mandible or midface model	P	20 (Test=10, Control=10)	Age: Test=40.5 ± 5.6 years, Control=66.1 ± 3.0 years Test=6 male, 4 female, Control=8 male, 2 female	Utility of Stereolithographic Models in Osteocutaneous Free Flap Reconstruction of the Head and Neck	Aim: to assess whether am AM model decreased the operative time enough to offset the cost of manufacturing the model.	- No significant differences were observed for total operative time between groups, but the total reconstructive time was lower in the control group (p=0.05).	⊞	⊞	⊟	⊞	⊞	⊞	⊞	⊟	X	⊞	?	?	⊞	⊟	⊟	X	X	⊞	⊞	⊞	?	?	⊟	X	⊟	X	⊞
Oral and Maxillofacial	3	Shahbazian	2013	Surgical guide for autotransplantation of teeth	S	40 (Test=20, Control=20)	Age: Test=9-18 years (mean=11 years), Control=9-18 years (mean=12 years) Test=12 male, 8 female, Control=13 male, 7 female	Validation of the cone beam computed tomography-based stereolithographic surgical guide aiding autotransplantation of teeth: clinical case-control study	Aim: to compare the outcome of AM surgical planning and transfer technique for tooth autotransplantation to conventional autotransplantation.	NS 6 from the control group showed 1 or more complications, compared to 2 from the test group.	⊞	⊞	⊟	⊞	⊞	⊞	⊟	⊞	⊟	⊟	?	?	⊞	⊟	⊞	⊞	⊟	⊞	⊞	⊞	⊟	⊟	⊟	X	⊟	?	⊟
Oral and Maxillofacial	3	Weitz	2010	Templates for navigated dental implantation	S	10 (Same participants used for test and control)	Age: 19-46 years (mean=29.9 years) 6 male, 4 female	Accuracy of templates for navigated implantation made by rapid prototyping with DICOM datasets of cone beam computer tomography (CBCT)	Aim: to compare the accuracy of AM surgical templates with templates made from conventional impressions for placing implants.	NS The conventional templates enabled excellent fitting accuracy but the accuracy of implants fitted with the AM templates was not sufficient and could result in significant iatrogenic complications.	⊞	⊟	⊟	⊟	⊟	⊞	⊞	⊞	X	⊟	?	?	?	⊟	⊟	?	X	⊞	⊞	⊞	X	X	X	X	X	X	⊟
Musculoskeletal	3	Otsuki	2013	Cutting guide for curved peri-acetabular osteotomy	S	18 (Test=7, Control=11)	Age: No information provided Test=All 7 female, Control= No information provided	Developing a novel custom cutting guide for curved peri-acetabular osteotomy	Aim: to evaluate the efficacy of this new method and cutting guide.	NS The rate of osteotomy-related complications did not differ significantly between the two groups [illegible] almost exactly to the planned cutting line in all patients.	⊞	⊞	⊟	⊞	⊟	⊟	⊞	⊞	⊞	⊟	?	?	⊞	⊟	⊟	⊞	⊟	⊞	⊞	⊞	⊟	?	⊟	X	⊟	⊞	⊟

⊞ Yes
⊟ No
? Unable to determine
X Not applicable

Medical sector	Level of evidence	First author	Date	Category	No. of participants	Title
Neoplasms	4	Huang	2012	S	31	A digital model individual template and CT-guided 125I seed implants for malignant tumors of the head and neck
Neoplasms	4	Silberstein	2014	P	5	Physical Models of Renal Malignancies Using Standard Cross-sectional Imaging and 3-Dimensional Printers: A Pilot Study
Neoplasms	4	Tao	2010	P	8	Application of computer-aided technique in pelvic precise model preparation and individualized prosthesis implantation
Neoplasms	4	von Rundstedt	2016	P	10	Utility of patient-specific silicone renal models for planning and rehearsal of complex tumour resections prior to robot-assisted laparoscopic partial nephrectomy.
Neoplasms	4	Xiao	2016	P	5	En Bloc Resection of Primary Malignant Bone Tumor in the Cervical Spine Based on 3-Dimensional Printing Technology.
Neoplasms	4	Zhang	2016a	P	10	Evaluation of three-dimensional printing for laparoscopic partial nephrectomy of renal tumors: a preliminary report
Nervous	4	Konrad	2010	S	263	Customized, Miniature Rapid-Prototype Stereotactic Frames for Use in Deep Brain Stimulator Surgery: Initial Clinical Methodology and Experience from 263 Patients from 2002 to 2008
Circulatory	4	Farooqi	2016	P	6	Application of Virtual Three-Dimensional Models for Simultaneous Visualization of Intracardiac Anatomic Relationships in Double Outlet Right Ventricle.
Circulatory	4	Gilon	2002	P	35	Effect of Three-Dimensional Valve Shape on the Hemodynamics of Aortic Stenosis: Three-Dimensional Echocardiographic Stereolithography and Patient Studies
Circulatory	4	Jacobs	2008	P	3	3D-Imaging of cardiac structures using 3D heart models for planning in heart surgery: a preliminary study
Circulatory	4	Liu	2016b	P	8	The Value of 3D Printing Models of Left Atrial Appendage Using Real-Time 3D Transesophageal Echocardiographic Data in Left Atrial Appendage Occlusion: Applications toward an Era of Truly Personalized Medicine.
Circulatory	4	Ngan	2006	P	4	The rapid prototyping of anatomic models in pulmonary atresia
Circulatory	4	Ripley	2016	P	16	3D printing based on cardiac CT assists anatomic visualization prior to transcatheter aortic valve replacement.
Circulatory	4	Weinstock	2015	P	4	Optimizing cerebrovascular surgical and endovascular procedures in children via personalized 3D printing
Digestive	4	Ko	2016	S	35	Novel 3D-printing technique for caps to enable tailored therapeutic endoscopy
Digestive	4	Zein	2013	P	6	Three-Dimensional Print of a Liver for Preoperative Planning in Living Donor Liver Transplantation
Oral and Maxillofacial	4	Asaumi	2001	P	2	Comparison of three-dimensional computed tomography with rapid prototype models in the management of coronoid hyperplasia
Oral and Maxillofacial	4	Barakat	2013	S	6	Clinical and radiographic evaluation of a computer-generated guiding device in bilateral sagittal split osteotomies
Oral and Maxillofacial	4	Bosc	2017	S	18	Mandibular reconstruction after cancer: an in-house approach to manufacturing cutting guides
Oral and Maxillofacial	4	Cassetta	2013	T	20	Accuracy of Two Stereolithographic Surgical Templates: A Retrospective Study
Oral and Maxillofacial	4	Chai	2015	P	7	Treating Parry-Romberg Syndrome Using Three-Dimensional Scanning and Printing and the Anterolateral Thigh Dermal Adipofascial Flap
Oral and Maxillofacial	4	Chan	2015	S	10	3D Rapid Prototyping for Otolaryngology—Head and Neck Surgery: Applications in Image-Guidance, Surgical Simulation and Patient-Specific Modeling
Oral and Maxillofacial	4	Chen	2014	P	6	Three-Dimensional Preoperative Design of Distraction Osteogenesis for Hemifacial Microsomia
Oral and Maxillofacial	4	Chen	2010	S	4	Modular Preoperative Planning Software for Computer-Aided Oral Implantology and the Application of a Novel Stereolithographic Template: A Pilot Study
Oral and Maxillofacial	4	Ciprandi	2012	T	5	Calcium Phosphate Cement in Orbital Reconstructions
Oral and Maxillofacial	4	Clelland	2016	S	18	Split-mouth comparison of splinted and nonsplinted prostheses on short implants: 3-year results
Oral and Maxillofacial	4	Cornelius	2016	S	12	Iterations of computer- and template assisted mandibular or maxillary reconstruction with free flaps containing the lateral scapular border-Evolution of a biplanar plug-on cutting guide.
Oral and Maxillofacial	4	Cui	2014	P	3	Surgical Planning, Three-Dimensional Model Surgery and Preshaped Implants in Treatment of Bilateral Craniomaxillofacial Post-Traumatic Deformities
Oral and Maxillofacial	4	Curcio	2007	P	14	Use of models in surgical predictability of oral rehabilitations
Oral and Maxillofacial	4	Dérand	2012	S	4	Imaging, Virtual Planning, Design, and Production of Patient-Specific Implants and Clinical Validation in Craniomaxillofacial Surgery
Oral and Maxillofacial	4	D'haese	2012	S	13	A Prospective Study on the Accuracy of Mucosally Supported Stereolithographic Surgical Guides in Fully Edentulous Maxillae
Oral and Maxillofacial	4	Di Giacomo	2011	S	12	Accuracy and Complications of Computer-Designed Selective Laser Sintering Surgical Guides for Flapless Dental Implant Placement and Immediate Definitive Prosthesis Installation
Oral and Maxillofacial	4	Di Giacomo	2005	S	4	Clinical Application of Stereolithographic Surgical Guides for Implant Placement: Preliminary Results
Oral and Maxillofacial	4	Di Giacomo	2016	S	9	A selective laser sintering prototype guide used to fabricate immediate interim fixed complete arch prostheses in flapless dental implant surgery: Technique description and clinical results.
Oral and Maxillofacial	4	El-Gengehi	2015	S	24	Evaluation of the Accuracy of Computer-Guided Mandibular Fracture Reduction
Oral and Maxillofacial	4	Ersoy	2008	S	21	Reliability of implant placement with stereolithographic surgical guides generated from computed tomography: clinical data from 94 implants
Oral and Maxillofacial	4	Fan	2007	P	17	Late Reconstruction of the Complex Orbital Fractures With Computer-Aided Design and Computer-Aided Manufacturing Technique
Oral and Maxillofacial	4	Feng	2011	S	4	Mirror imaging and preshaped titanium plates in the treatment of unilateral malar and zygomatic arch fractures
Oral and Maxillofacial	4	Fiaschi	2016	T	12	Surgical results of cranioplasty with a polymethylmethacrylate customized cranial implant in pediatric patients: a single-center experience.
Oral and Maxillofacial	4	Gaggl	1998	P	3	Treatment planning for sinus lift augmentations through use of 3-dimensional milled models derived from computed tomography scans
Oral and Maxillofacial	4	Guo	2009	T	61	Reconstruction of Orbital Floor Fractures
Oral and Maxillofacial	4	Huang	2017	P	97	Personalized Reconstruction of Traumatic Orbital Defects Based on Precise Three-Dimensional Orientation and Measurements of the Globe.
Oral and Maxillofacial	4	Jiang	2015	T	18	Functional Evaluation of a CAD/CAM Prosthesis for Immediate Defect Repair after Total Maxillectomy: A Case Series of 18 Patients with Maxillary Sinus Cancer
Oral and Maxillofacial	4	Jiao	2014	T	11	Rehabilitation of maxillectomy defects with obturator prostheses fabricated using computer-aided design and rapid prototyping: a pilot study.
Oral and Maxillofacial	4	Kim	2004	P	182	Evaluation of the prognosis and causes of failure in 182 cases of autogenous tooth transplantation
Oral and Maxillofacial	4	Kocyiğit	2013	S	10	Evaluation of Preoperative Model Surgery and the Use of a Maxillary Sinus Surgical Template in Sinus Floor Augmentation Surgery
Oral and Maxillofacial	4	Komiyama	2012	S	29	Soft Tissue Conditions and Marginal Bone Changes around Immediately Loaded Implants Inserted in Edentate Jaws Following Computer Guided Treatment Planning and Flapless Surgery: A 31-Year Clinical Follow-Up
Oral and Maxillofacial	4	Kozakiewicz	2009	P	6	Clinical application of 3D pre-bent titanium implants for orbital floor fractures
Oral and Maxillofacial	4	Labadie	2008	S	4	Clinical Validation of Percutaneous Cochlear Implant Surgery: Initial Report
Oral and Maxillofacial	4	Lethaus	2010	T	11	Surgical and prosthetic reconsiderations in patients with maxillectomy
Oral and Maxillofacial	4	Li	2013a	T	6	Digital Design and Individually Fabricated Titanium Implants for the Reconstruction of Traumatic Zygomatico-Orbital Defects
Oral and Maxillofacial	4	Li	2015b	T	12	Clinical Feasibility and Efficacy of Using Virtual Surgical Planning in Bimaxillary Orthognathic Surgery Without Intermediate Splint
Oral and Maxillofacial	4	Li	2013b	S	6	A novel method of computer aided orthognathic surgery using individual CAD/CAM templates: a combination of osteotomy and repositioning guides
Oral and Maxillofacial	4	Li	2016c	S	7	Treatment of Dentofacial Deformities Secondary to Osteochondroma of the Mandibular Condyle Using Virtual Surgical Planning and 3-Dimensional Printed Surgical Templates
Oral and Maxillofacial	4	Li	2016b	S	9	A new design of CAD/CAM surgical template system for two-piece narrowing genioplasty.
Oral and Maxillofacial	4	Lin	2016	S	5	Mandibular angle split osteotomy based on a novel augmented reality navigation using specialized robot-assisted arms--A feasibility study.
Oral and Maxillofacial	4	Lieger	2010	P	29	Computer-Assisted Design and Manufacture of Implants in the Late Reconstruction of Extensive Orbital Fractures
Oral and Maxillofacial	4	Liu	2010	S	11	Accuracy of surgical positioning of orthodontic miniscrews with a computer-aided design and manufacturing template
Oral and Maxillofacial	4	Liu	2013	S	8	Digital Surgical Templates for Managing High-Energy Zygomaticomaxillary Complex Injuries Associated With Orbital Volume Change: A Quantitative Assessment
Oral and Maxillofacial	4	Liu	2009	S	7	Applying Computer Techniques in Maxillofacial Reconstruction Using a Fibula Flap: A Messenger and an Evaluation Method
Oral and Maxillofacial	4	Liu	2014a	S	15	Technical procedures for template-guided surgery for mandibular reconstruction based on digital design and manufacturing
Oral and Maxillofacial	4	Martorelli	2012	S	6	A comparison between customized clear and removable orthodontic appliances manufactured using RP and CNC techniques
Oral and Maxillofacial	4	Mazzoni	2015	S	10	Computer-Aided Design and Computer-Aided Manufacturing Cutting Guides and Customized Titanium Plates Are Useful in Upper Maxilla Waferless Repositioning
Oral and Maxillofacial	4	Modabber	2012a	S	15	Computer-assisted Mandibular Reconstruction with Vascularized Iliac Crest Bone Graft
Oral and Maxillofacial	4	Morea	2011	S	4	Precise Insertion of Orthodontic Miniscrews with a Stereolithographic Surgical Guide Based on Cone Beam Computed Tomography Data: A Pilot Study
Oral and Maxillofacial	4	Muller	2003	P	52	The Application of Rapid Prototyping Techniques in Cranial Reconstruction and Preoperative Planning in Neurosurgery
Oral and Maxillofacial	4	Murray	2008	P	3	Advanced technology in the management of fibrous dysplasia
Oral and Maxillofacial	4	Ozan	2011	S	54	Correlation Between Bone Density and Angular Deviation of Implants Placed Using CT-Generated Surgical Guides
Oral and Maxillofacial	4	Ozan	2009	S	30	Clinical Accuracy of 3 Different Types of Computed Tomography-Derived Stereolithographic Surgical Guides in Implant Placement
Oral and Maxillofacial	4	Papaspyridakos	2012a	T	14	Computer-assisted design/computer-assisted manufacturing zirconia implant fixed complete prostheses: clinical results and technical complications up to 4 years of function
Oral and Maxillofacial	4	Park	2015	P	127	Mirror-Imaged Rapid Prototype Skull Model and Pre-Molded Synthetic Scaffold to Achieve Optimal Orbital Cavity Reconstruction
Oral and Maxillofacial	4	Park	2016	T	21	Cranioplasty Enhanced by Three-Dimensional Printing: Custom-Made Three-Dimensional-Printed Titanium Implants for Skull Defects.
Oral and Maxillofacial	4	Pettersson	2010	S	25	Accuracy of Virtually Planned and Template Guided Implant Surgery on Edentate Patientscid_285 527..537
Oral and Maxillofacial	4	Prisman	2014	P	10	Value of preoperative mandibular plating in reconstruction of the mandible
Oral and Maxillofacial	4	Reiser	2015	S	17	V-Stand—A Versatile Surgical Platform for Oromandibular Reconstruction Using a 3-Dimensional Virtual Modeling System
Oral and Maxillofacial	4	Rotaru	2012	T	10	Cranioplasty With Custom-Made Implants: Analyzing the Cases of 10 Patients
Oral and Maxillofacial	4	Sailer	1998	P	20	The value of stereolithographic models for preoperative diagnosis of craniofacial deformities and planning of surgical corrections
Oral and Maxillofacial	4	Schantz	2006	T	5	Cranioplasty after trephination using a novel biodegradable burr hole cover: technical case report
Oral and Maxillofacial	4	Scolozzi	2009	P	10	Accuracy and Predictability in Use of AO Three-Dimensionally Preformed Titanium Mesh Plates for Posttraumatic Orbital Reconstruction: A Pilot Study
Oral and Maxillofacial	4	Shan	2015	T	2	Surgical Reconstruction of Maxillary and Mandibular Defects Using a Printed Titanium Mesh
Oral and Maxillofacial	4	Shehab	2013	S	6	A novel design of a computer-generated splint for vertical repositioning of the maxilla after Le Fort I osteotomy
Oral and Maxillofacial	4	Shen	2015	P	17	Presurgical Nasoalveolar Molding for Cleft Lip and Palate: The Application of Digitally Designed Molds
Oral and Maxillofacial	4	Shu	2014	S	8	Accuracy of using computer-aided rapid prototyping templates for mandible reconstruction with an iliac crest graft
Oral and Maxillofacial	4	Staffa	2006	S	26	Custom made cranioplasty prostheses in porous hydroxy-apatite using 3D design techniques: 7 years experience in 25 patients
Oral and Maxillofacial	4	Stubinger	2014	S	10	Deviations between Placed and Planned Implant Positions: An Accuracy Pilot Study of Skeletally Supported Stereolithographic Surgical Templates
Oral and Maxillofacial	4	Sun	2013a	S	15	Accuracy of Upper Jaw Positioning With Intermediate Splint Fabrication After Virtual Planning in Bimaxillary Orthognathic Surgery
Oral and Maxillofacial	4	Sun	2013b	S	6	Error analysis of a CAD/CAM method for unidirectional mandibular distraction osteogenesis in the treatment of hemifacial microsomia
Oral and Maxillofacial	4	Suomalainen	2015	P	102	Rapid prototyping modelling in oral and maxillofacial surgery: A two year retrospective study.
Oral and Maxillofacial	4	Tabaković	2015	T	10	Application of Computer-Aided Designing and Rapid Prototyping Technologies in Reconstruction of Blowout Fractures of the Orbital Floor
Oral and Maxillofacial	4	Tang	2010	P	46	Individual Design and Rapid Prototyping in Reconstruction of Orbital Wall Defects
Oral and Maxillofacial	4	Turgut	2012	T	11	Computer-Aided Design and Manufacture and Rapid Prototyped Polymethylmethacrylate Reconstruction
Oral and Maxillofacial	4	Valente	2007	S	25	Accuracy and clinical outcomes of computer aided oral implant surgery: a preliminary study on 25 patients
Oral and Maxillofacial	4	Van Assche	2010	S	8	Accuracy assessment of computer-assisted flapless implant placement in partial edentulism
Oral and Maxillofacial	4	Verhamme	2012	S	5	A clinically relevant validation method for implant placement after virtual planning
Oral and Maxillofacial	4	Vrielinck	2003	S	12	Image-based planning and clinical validation of zygoma and pterygoid implant placement in patients with severe bone atrophy using customized drill guides. Preliminary results from a prospective clinical follow-up study
Oral and Maxillofacial	4	Wang	2013a	P	10	Three-dimensional virtual technology in reconstruction of mandibular defect including condyle using double-barrel vascularized fibula flap
Oral and Maxillofacial	4	Wiggins	2013	S	113	Cranioplasty With Custom-Made Titanium Plates—14 Years Experience
Oral and Maxillofacial	4	Won	2013	P	21	Improving pre-operative planning for complex total hip replacement with a Rapid Prototype model enabling surgical simulation
Oral and Maxillofacial	4	Wong	2005	S	13	Comparison of 2 Methods of Making Surgical Models for Correction of Facial Asymmetry
Oral and Maxillofacial	4	Xu	2015a	S	24	Combined Use of Rapid-Prototyping Model and Surgical Guide in Correction of Mandibular Asymmetry Malformation Patients With Normal Occlusal Relationship
Oral and Maxillofacial	4	Yin	2014	S	36	Mandibular Distraction Combined With Orthognathic Techniques for the Correction of Severe Adult Mandibular Hypoplasia
Oral and Maxillofacial	4	Ying	2015	S	14	Correction of facial asymmetry associated with vertical maxillary excess and mandibular prognathism by combined orthognathic surgery and guiding templates and splints fabricated by rapid prototyping technique
Oral and Maxillofacial	4	Yu	2013	P	15	CAD presurgical nasoalveolar molding effects on the maxillary morphology in infants with UCLP
Oral and Maxillofacial	4	Yuan	2013	T	10	Accuracy evaluation of a new three-dimensional reproduction method of edentulous dental casts, and wax occlusion rims with jaw relation
Oral and Maxillofacial	4	Zhang	2015	T	12	Computer-Aided Design and Computer-Aided Manufacturing Hydroxyapatite/Epoxide Acrylate Maleic Compound Construction for Craniomaxillofacial Bone Defects
Oral and Maxillofacial	4	Zhang	2009	S	6	Application of navigation template to fixation of sacral fracture using three-dimensional reconstruction and reverse engineering technique
Oral and Maxillofacial	4	Zhang	2016b	P	30	Accuracy of virtual surgical planning in two-jaw orthognathic surgery: comparison of planned and actual results
Oral and Maxillofacial	4	Zheng	2012	T	9	Mandible reconstruction assisted by preoperative virtual surgical simulation
Oral and Maxillofacial	4	Zhong	2015	T	10	Quantitative Analysis of Dual-Purpose, Patient- Specific Craniofacial Implants for Correction of Temporal Deformity
Oral and Maxillofacial	4	Zinser	2012	S	8	Computer-assisted orthognathic surgery: feasibility study using multiple CAD/CAM surgical splints
Oral and Maxillofacial	4	Zou	2015	S	6	Application of Computer Techniques in Correcting Mild Zygomatic Assymetry With Unilateral Reduction Malarplasty
Musculoskeletal	4	Bauer	2015	P	19	Preoperative Computer Simulation and Patient-specific Guides are Safe and Effective to Correct Forearm Deformity in Children
Musculoskeletal	4	Chen	2016a	S	5	A novel navigation template for fixation of acetabular posterior column fractures with antegrade lag screws: design and application
Musculoskeletal	4	Dai	2007	T	10	Computer-Aided Custom-Made Hemipelvic Prosthesis Used in Extensive Pelvic Lesions
Musculoskeletal	4	Deng	2016	S	10	The accuracy and the safety of individualized 3D printing screws insertion templates for cervical screw insertion.
Musculoskeletal	4	Dhanshyani	2011	P	6	Rapid prototyping models for dysplastic hip surgeries in Malaysia
Musculoskeletal	4	Guarino	2007	P	13	Rapid Prototyping Technology for Surgeries of the Pediatric Spine and Pelvis
Musculoskeletal	4	Hananouchi	2009	S	24	Tailor-made surgical guide based on rapid prototyping technique for cup insertion in total hip arthroplasty
Musculoskeletal	4	Hsieh	2010	P	12	Repositioning Osteotomy for Intra-Articular Malunion of Distal Radius With Radiocarpal and/or Distal Radioulnar Joint Subluxation
Musculoskeletal	4	Hu	2016	S	151	A comparative study on the accuracy of pedicle screw placement assisted by personalized rapid prototyping template between pre- and post-operation in patients with relatively normal mid-upper thoracic spine.
Musculoskeletal	4	Jiang	2016	S	32	Accuracy assessment of atlantoaxial pedicle screws assisted by a novel drill guide template.
Musculoskeletal	4	Kaneyama	2015	S	20	Safe and Accurate Midcervical Pedicle Screw Insertion Procedure With the Patient-Specifi c Screw Guide Template System
Musculoskeletal	4	Kawaguchi	2012	S	11	Development of a New Technique for Pedicle Screw and Magerl Screw Insertion Using a 3-Dimensional Image Guide
Musculoskeletal	4	Kunz	2010	P	45	Computer-Assisted Hip Resurfacing Using Individualized Drill Templates
Musculoskeletal	4	Kunz	2011	P	80	Registration stability of physical tem-plates in hip surgery
Musculoskeletal	4	Li	2013c	P	25	Revision of Complex Acetabular Defects Using Cages with the Aid of Rapid Prototyping.
Musculoskeletal	4	Li	2015c	T	26	Custom Acetabular Cages Offer Stable Fixation and Improved Hip Scores for Revision THA With Severe Bone Defects
Musculoskeletal	4	Lin	2013	P	10	Application of three-dimensional model in the management of irreducible atlanto-axial dislocation
Musculoskeletal	4	Liu	2016a	S	10	Preliminary application of a multi-level 3D printing drill guide template for pedicle screw placement in severe and rigid scoliosis.
Musculoskeletal	4	Lu	2009a	S	25	A Novel Patient-Specific Navigational Template for Cervical Pedicle Screw Placement
Musculoskeletal	4	Lu	2012	S	16	Accuracy and efficacy of thoracic pedicle screws in scoliosis with patient-specific drill template
Musculoskeletal	4	Mao	2010	P	16	Clinical application of computer-designed polystyrene models in complex severe spinal deformities: a pilot study
Musculoskeletal	4	Miyake	2012	S	20	Computer-Assisted Corrective Osteotomy for Malunited Diaphyseal Forearm Fractures
Musculoskeletal	4	Murase	2005	P	7	Does Three-dimensional Computer Simulation Improve Results of Scaphoid Nonunion Surgery?
Musculoskeletal	4	Murase	2008	S	22	Three-Dimensional Corrective Osteotomy of Malunited Fractures of the Upper Extremity with Use of a Computer Simulation System
Musculoskeletal	4	Parchi	2013	P	8	Computer Tomography Prototyping and Virtual Procedure Simulation in Difficult Cases of Hip Replacement Surgery
Musculoskeletal	4	Putzier	2014	S	4	A New Navigational Tool for Pedicle Screw Placement in Patients with Severe Scoliosis: A Pilot Study to Prove Feasibility, Accuracy, and Identify Operative Challenges.
Musculoskeletal	4	Schweizer	2013	S	6	Three-Dimensional Correction of Distal Radius Intra-Articular Malunions Using Patient-Specific Drill Guides
Musculoskeletal	4	Takemoto	2016	S	36	Additive-manufactured patient-specific titanium templates for thoracic pedicle screw placement: novel design with reduced contact area.
Musculoskeletal	4	Takeyasu	2013	S	30	Preoperative, Computer Simulation-Based, Three-Dimensional Corrective Osteotomy for Cubitus Varus Deformity with Use of a Custom-Designed Surgical Device
Musculoskeletal	4	Tricot	2012	S	3	3D-corrective osteotomy using surgical guides for posttraumatic distal humeral deformity
Musculoskeletal	4	Yang	2011	S	21	Personalised modified osteotomy using computer-aided design–rapid prototyping to correct thoracic deformities
Musculoskeletal	4	Zeng	2015	P	38	Evaluation of three-dimensional printing for internal fixation of unstable pelvic fracture from minimal invasive para-rectus abdominis approach: a preliminary report
Musculoskeletal	4	Zhang	2011c	S	18	Application of computer-aided design osteotomy template for treatment of cubitus varus deformity in teenagers: A pilot study
Musculoskeletal	4	Zhuang	2015	P	14	Surgical Treatment of Acetabulum Top Compression Fracture with Sea Gull Sign
Musculoskeletal	4	Hurson	2007	P	20	Rapid prototyping in the assessment, classification and preoperative planning of acetabular fractures
Musculoskeletal	4	Kunz	2012	S	31	Hip resurfacing with individualized drill templates— comparison between anterolateral and posterior approach
Musculoskeletal	4	Xu	2015b	P	10	Application of Rapid Prototyping Pelvic Model for Patients with DDH to Facilitate Arthroplasty Planning: A Pilot Study.
Musculoskeletal	4	Zeng	2016	P	10	A combination of three-dimensional printing and computer-assisted virtual surgical procedure for preoperative planning of acetabular fracture reduction.
External Devices	4	Fabry	2002	T	43	Hearing aid physical fit: The next revolution?
External Devices	4	Gibson	2014	T	15	Functionally Optimized Orthoses for Early Rheumatoid Arthritis Foot Disease: A Study of Mechanisms and Patient Experience
External Devices	4	Liu	2014b	T	10	Newly Designed Foot Orthosis for Children with Residual Clubfoot After Ponseti Casting
External Devices	4	Pallari	2010	T	7	Mass Customization of Foot Orthoses for Rheumatoid Arthritis Using Selective Laser Sintering
External Devices	4	Raux	2014	T	30	Tridimensional trunk surface acquisition for brace manufacturing in idiopathic scoliosis
External Devices	4	Telfer	2013a	T	24	Dose-response effects of customised foot orthoses on lower limb kinematics and kinetics in pronated foot type
External Devices	4	Telfer	2013b	T	12	Dose-response effects of customised foot orthoses on lower limb muscle activity and plantar pressures in pronated foot type
Neoplasms	5	Akiba	2015	P	1	A Three-Dimensional Mediastinal Model Created with Rapid Prototyping in a Patient with Ectopic Thymoma
Neoplasms	5	D'Urso	1999b	P	1	Biomodel guided Stereotaxy
Neoplasms	5	Krauel	2016	P	3	Use of 3D Prototypes for Complex Surgical Oncologic Cases
Neoplasms	5	Mainenti	2009	P	1	Ameloblastic fibro-odontosarcoma: a case report
Neoplasms	5	Wong	2015	T	1	One-step reconstruction with a 3D-printed, biomechanically evaluated custom implant after complex pelvic tumor resection
Neoplasms	5	Wiebe	2015	S	1	Customized vaginal vault brachytherapy with computed tomography imaging-derived applicator prototyping
Nervous	5	D'Urso	1999c	P	16	Cerebrovascular biomodelling: a technical note
Circulatory	5	Bosi	2015	P	1	Patient-Specific Finite Element Models to Support Clinical Decisions: A Lesson Learnt from a Case Study of Percutaneous Pulmonary Valve Implantation
Circulatory	5	Dankowski	2014	P	1	3D heart model printing for preparation of percutaneous structural interventions: description of the technology and case report
Circulatory	5	Ejaz	2014	S	3	Color-coded patient-specific physical models of congenital heart disease
Circulatory	5	Erbano	2013	P	3	Rapid prototyping of three-dimensional biomodels as an adjuvant in the surgical planning for intracranial aneurysms
Circulatory	5	Gosnell	2016	P	1	Integration of Computed Tomography and Three-Dimensional Echocardiography for Hybrid Three-Dimensional Printing in Congenital Heart Disease.
Circulatory	5	Hermsen	2017	P	2	Scan, plan, print, practice, perform: Development and use of a patient-specific 3-dimensional printed model in adult cardiac surgery.
Circulatory	5	Kiraly	2016	P	1	Three-dimensional printed prototypes refine the anatomy of post-modified Norwood-1 complex aortic arch obstruction and allow presurgical simulation of the repair.
Circulatory	5	Lan	2016	P	1	Development of Three-Dimensional Printed Craniocerebral Models for Simulated Neurosurgery.
Circulatory	5	Mottle-link	2008	P	1	Physical Models Aiding in Complex Congenital Heart Surgery
Circulatory	5	Pepper	2013	S	34	Implantation of an individually computer-designed and manufactured external support for the Marfan aortic root
Circulatory	5	Riesenkampff	2009	P	11	The practical clinical value of three-dimensional models of complex congenitally malformed hearts
Circulatory	5	Sodian	2009	P	1	3-Dimensional Printing of Models to Create Custom-Made Devices for Coil Embolization of an Anastomotic Leak After Aortic Arch Replacement
Circulatory	5	Sardari Nia	2016	P	1	Preoperative planning with three-dimensional reconstruction of patient's anatomy, rapid prototyping and simulation for endoscopic mitral valve repair.
Circulatory	5	Shimamura	2012	P	4	Three-Dimensional Replica of a Life-Sized Model of Aortic Arch Aneurysm for Preoperative Assessments
Circulatory	5	Tam	2013	P	1	3D Printing of an Aortic Aneurysm to Facilitate Decision Making and Device Selection for Endovascular Aneurysm Repair in Complex Neck Anatomy
Circulatory	5	Vranicar	2008	P	12	The use of stereolithographic hand held models for evaluation of congenital anomalies of the great arteries
Circulatory	5	Wurm	2004	P	13	Cerebrovascular stereolithographic biomodeling for aneurysm surgery
Respiratory	5	Booysen	2006	S	1	Anaesthetic mouthpiece development through QFD and customer interaction with functional prototypes
Skin	5	Chae	2015	P	1	3D-Printed haptic "Reverse" models for preoperative planning in soft tissue reconstruction: A case report
Skin	5	Wei	2016	T	2	3D-printed transparent facemasks in the treatment of facial hypertrophic scars of young children with burns.
Oral and Maxillofacial	5	Abe	1998	P	7	Model-based surgical planning and simulation of cranial base surgery
Oral and Maxillofacial	5	Abou-ElFetouh	2011	S	2	Computer-guided rapid-prototyped templates for segmental mandibular osteotomies: a preliminary report
Oral and Maxillofacial	5	Adolphs	2013	P	2	Craniomaxillofacial surgery planning based on 3D models derived from Cone-Beam CT data
Oral and Maxillofacial	5	Aita-Holmes	2015	T	1	Digital capture, design, and manufacturing of an extraoral device for a clarinet player with Bell's palsy
Oral and Maxillofacial	5	Aitasalo	2014	T	12	Craniofacial bone reconstruction with bioactive fiber-reinforced composite implant
Oral and Maxillofacial	5	Bai	2012	T	7	An Alternative Approach for Mandible Reconstruction
Oral and Maxillofacial	5	Bai	2010	S	1	CAD/CAM surface templates as an alternative to the intermediate wafer in orthognathic surgery
Oral and Maxillofacial	5	Bellini	2007	P	1	Bi-directional distraction in the treatment of micro-orbitism: A case report
Oral and Maxillofacial	5	Bibb	2006	T	1	Trial fitting of a removable partial denture framework made using computer-aided design and rapid prototyping techniques
Oral and Maxillofacial	5	Brie	2012	T	8	A new custom made bioceramic implant for the repair of large and complex craniofacial bone defects
Oral and Maxillofacial	5	Byun	2015	S	1	Endodontic Treatment of an Anomalous Anterior Tooth with the Aid of a 3-dimensional Printed Physical Tooth Model
Oral and Maxillofacial	5	Cao	2010	T	39	Application of EH Compound Artificial Bone Material Combined With Computerized Three-Dimensional Reconstruction in Craniomaxillofacial Surgery
Oral and Maxillofacial	5	Cassetta	2016	S	1	Accelerating orthodontic tooth movement: A new, minimally-invasive corticotomy technique using a 3D-printed surgical template.
Oral and Maxillofacial	5	Cesarano	2005	T	1	Customization of Load-Bearing Hydroxyapatite Lattice Scaffolds
Oral and Maxillofacial	5	Cho	2013	T	1	Mirror-image anterior crown fabrication with computer-aided design and rapid prototyping technology: A clinical report
Oral and Maxillofacial	5	Chow	2006	S	5	Zygomatic Implants—Protocol for Immediate Occlusal Loading: A Preliminary Report
Oral and Maxillofacial	5	Chua	1998	P	1	Rapid Prototyping Assisted Surgery Planning
Oral and Maxillofacial	5	Ciocca	2010a	T	1	Immediate facial rehabilitation in cancer patients using CAD–CAM and rapid prototyping technology: a pilot study
Oral and Maxillofacial	5	Ciocca	2010b	S	1	Rehabilitation of the nose using CAD-CAM and rapid prototyping technology after ablative surgery of squamous cell carcinoma: A pilot clinical report
Oral and Maxillofacial	5	Ciocca	2012	S	1	CAD/CAM guided secondary mandibular reconstruction of a discontinuity defect after ablative cancer surgery
Oral and Maxillofacial	5	Da Rosa	2004	P	1	Rapid Prototyping in Maxillofacial Surgery and Traumatology: Case Report
Oral and Maxillofacial	5	Elbashti	2016	T	1	The Role of Digitization in the Rapid Reproduction of an Obturator in a Frail Elderly Patient.
Oral and Maxillofacial	5	Fang	2013	S	2	Complex Facial Deformity Reconstruction With a Surgical Guide Incorporating a Built-in Occlusal Stent as the Positioning Reference
Oral and Maxillofacial	5	Feng	2010	P	1	Mirror imaging and preshaped titanium plates in the treatment of unilateral malar and zygomatic arch fractures
Oral and Maxillofacial	5	Fernandes	2007	P	1	Computer-aided surgery using 3D rendering of maxillofacial pathology and trauma
Oral and Maxillofacial	5	Figliuzzi	2012	T	1	A novel root analogue dental implant using CT scan and CAD/CAM: selective laser melting technology
Oral and Maxillofacial	5	Flugge	2013	S	1	Three-Dimensional Plotting and Printing of an Implant Drilling Guide: Simplifying Guided Implant Surgery
Oral and Maxillofacial	5	Goh	2009	T	31	Customised fabricated implants after previous failed cranioplasty
Oral and Maxillofacial	5	Gong	2012	P	7	Correction of maxillary deformity in infants with bilateral cleft lip and palate using computer-assisted design
Oral and Maxillofacial	5	Gopakumar	2004	T	1	RP in medicine: a case study in cranial reconstructive surgery
Oral and Maxillofacial	5	Hatamleh	2013a	T	1	Construction of an Implant-Retained Auricular Prosthesis with the Aid of Contemporary Digital Technologies: A Clinical Report
Oral and Maxillofacial	5	Hatamleh	2013b	T	4	Management of Extensive Frontal Cranioplasty Defects
Oral and Maxillofacial	5	He	2015	T	7	Novel Arch Bar Fabricated With a Computer-Aided Design and Three-Dimensional Printing: A Feasibility Study
Oral and Maxillofacial	5	Herlin	2011	S	1	Rapid prototyping in craniofacial surgery: Using a positioning guide after zygomatic osteotomy e A case report
Oral and Maxillofacial	5	Hierl	2013	S	3	CAD-CAM–Assisted Esthetic Facial Surgery
Oral and Maxillofacial	5	Holck	1999	P	2	Benefits of Stereolithography in Orbital Reconstruction
Oral and Maxillofacial	5	Hou	2011	P	7	Immediate Reconstruction of Bilateral Mandible Defects: Management Based on Computer-Aided Design/Computer-Aided Manufacturing Rapid Prototyping Technology in Combination With Vascularized Fibular Osteomyocutaneous Flap
Oral and Maxillofacial	5	Hu	2007	P	1	Reconstruction of a palatomaxillary defect with vascularized iliac bone combined with a superficial inferior epigastric artery flap and zygomatic implants as anchorage
Oral and Maxillofacial	5	Jang	2013	P	4	Autotransplantation of immature third molars using a computer-aided rapid prototyping model: a report of 4 cases
Oral and Maxillofacial	5	Juergens	2012	S	1	A Computer-Assisted Diagnostic and Treatment Concept to Increase Accuracy and Safety in the Extracranial Correction of Cranial Vault Asymmetries
Oral and Maxillofacial	5	Keightley	2010	S	1	Autotransplantation of an immature premolar, with the aid of cone beam CT and computeraided prototyping: a case report
Oral and Maxillofacial	5	Kfir	2013	P	1	The diagnosis and conservative treatment of a complex type 3 dens invaginatus using cone beam computed tomography (CBCT) and 3D plastic models
Oral and Maxillofacial	5	Kim	2007	S	1	Surgical positioning of orthodontic miniimplants with guides fabricated on models replicated with cone-beam computed tomography
Oral and Maxillofacial	5	Kim	2008	S	1	Clinical application of a stereolithographic surgical guide for simple positioning of orthodontic mini-implants
Oral and Maxillofacial	5	Kübler	1995	P	21	Repair of human skull defects using osteoinductive bone alloimplants
Oral and Maxillofacial	5	Kusek	2009	P	1	The use of laser technology (Er;Cr:YSGG) and stereolithography to aid in the placement of a subperiosteal implant: case study
Oral and Maxillofacial	5	Lee	2015	S	1	Keyhole System: A Computer-Assisted Designed and Computer-Assisted Manufactured Maxillomandibular Complex Repositioner in Orthognathic Surgery
Oral and Maxillofacial	5	Li	2009	P	1	Application of computer-aided three-dimensional skull model with rapid prototyping technique in repair of zygomatico-orbito-maxillary complex fracture
Oral and Maxillofacial	5	Li	2012	P	1	Preliminary Application of Virtual Simulation and Reposition Template for Zygomatico-orbitomaxillary Complex Fracture
Oral and Maxillofacial	5	Liu	2014c	S	1	Additive manufacturing and digital design assisted precise apicoectomy: a case study
Oral and Maxillofacial	5	Lucey	2010	P	1	Case report: Cone-beam CT imaging in the management of a double tooth
Oral and Maxillofacial	5	Matsuo	2010	T	2	Clinical application of a custom-made bioresorbable raw particulate hydroxyapatite/poly-L-lactide mesh tray for mandibular reconstruction
Oral and Maxillofacial	5	Mavili	2007	P	3	Use of Three-Dimensional Medical Modeling Methods for Precise Planning of Orthognathic Surgery
Oral and Maxillofacial	5	Mendez	2015	P	2	Customized ''In-Office'' Three-Dimensional Printing for Virtual Surgical Planning in Craniofacial Surgery
Oral and Maxillofacial	5	Oliveira	2010	P	1	Recessive dystrophic epidermolysis bullosa—oral rehabilitation using stereolithography and immediate endosseous implants
Oral and Maxillofacial	5	Olszewski	2010	P	1	Innovative procedure for computer-assisted genioplasty: three-dimensional cephalometry, rapid-prototyping model and surgical splint
Oral and Maxillofacial	5	Ozan	2012	S	1	Clinical Application of Stereolithographic Surgical Guide With a Handpiece Guidance Apparatus: A Case Report
Oral and Maxillofacial	5	Papaspyridakos	2015	S	1	Full Mouth Implant Rehabilitation with Staged Approach: 6-Year Clinical Follow-Up
Oral and Maxillofacial	5	Papaspyridakos	2010	S	1	Immediate Loading of the Maxilla with Prefabricated Interim Prosthesis Using Interactive Planning Software, and CAD/CAM Rehabilitation with Definitive Zirconia Prosthesis: 2-Year Clinical Follow-up
Oral and Maxillofacial	5	Papaspyridakos	2012b	S	1	Flapless CAD/CAM-guided surgery for staged transition from failing dentition to complete arch implant rehabilitation: a 3-year clinical report
Oral and Maxillofacial	5	Papaspyridakos	2008	S	1	Complete arch implant rehabilitation using subtractive rapid prototyping and porcelain fused to zirconia prosthesis: A clinical report
Oral and Maxillofacial	5	Park	2012	P	1	Autotransplantation with Simultaneous Sinus Floor Elevation
Oral and Maxillofacial	5	Park	2014	P	1	Optimizing Third Molar Autotransplantation: Applications of Reverse-Engineered Surgical Templates and Rapid Prototyping of Three-Dimensional Teeth
Oral and Maxillofacial	5	Rohner	2013	P	1	Importance of patient-specific intraoperative guides in complex maxillofacial reconstruction
Oral and Maxillofacial	5	Salmi	2013	T	1	A digital process for additive manufacturing of occlusal splints: a clinical pilot study
Oral and Maxillofacial	5	Sammartino	2004	S	1	Stereolithography in Oral Implantology A Comparison of Surgical Guides
Oral and Maxillofacial	5	Seitz	2016	T	1	A Combination of Various Technologies in the Fabrication of a Removable Partial Denture--A Case Study.
Oral and Maxillofacial	5	Shengwei	2014	T	7	Combined Use of an Anterolateral Thigh Flap and Rapid Prototype Modeling to Reconstruct Maxillary Oncologic Resections and Midface Defects
Oral and Maxillofacial	5	Shin	2015	S	1	A case report of a surgical guide fabricated via intraoral scanning-based implant planning and wax-based rapid prototyping
Oral and Maxillofacial	5	Singare	2007	P	2	Individually Prefabricated Prosthesis for Maxilla Reconstruction
Oral and Maxillofacial	5	Sultan	2011	S	3	Custom-Made, 3D, Intraoperative Surgical Guides for Nasal Reconstruction
Oral and Maxillofacial	5	Suzuki	2005	P	2	Application of a rapid-prototyped temporal bone model for surgical planning
Oral and Maxillofacial	5	Thomas	2013	S	4	Use of a titanium cutting guide to assist with raising and inset of a DCIA free flap
Oral and Maxillofacial	5	Toso	2005	P	16	Usefulness of Computed Tomography in pre-surgical evaluation of maxillo-facial pathology with rapid prototyping and surgical pre-planning by virtual reality
Oral and Maxillofacial	5	Turgut	2009	T	10	Use of Rapid Prototyping in Prosthetic Auricular Restoration
Oral and Maxillofacial	5	Verweij	2016	P	1	Autotransplantation of Premolars With a 3-Dimensional Printed Titanium Replica of the Donor Tooth Functioning as a Surgical Guide: Proof of Concept.
Oral and Maxillofacial	5	Watson	2014	T	1	Correction of Facial and Mandibular Asymmetry Using a Computer Aided Design/ Computer Aided Manufacturing Prefabricated Titanium Implant
Oral and Maxillofacial	5	Wiechmann	2008	T	1	Herbst appliance in lingual orthodontics
Oral and Maxillofacial	5	Wang	2013b	S	5	Application of Computer Techniques in Repair of Oblique Facial Clefts With Outer-Table Calvarial Bone Grafts
Oral and Maxillofacial	5	Wang	2015	P	1	Complex reconstruction of facial deformity and function after severe gunshot injury: one case report
Oral and Maxillofacial	5	Yoshioka	2010	T	1	Fabrication of an Orbital Prosthesis Using a Non-contact Three-Dimensional Digitizer and Rapid-Prototyping System
Oral and Maxillofacial	5	Zhen-yu	2010	P	1	Computer-assisted design and rapid prototyping technology for the underlay of hemifacial atrophy
Oral and Maxillofacial	5	Zhou	2010	T	6	Accurate Reconstruction of Discontinuous Mandible Using a Reverse Engineering/ Computer-Aided Design/Rapid Prototyping Technique: A Preliminary Clinical Study
Oral and Maxillofacial	5	Zhu	2011	P	15	Registration Strategy Using Occlusal Splint Based on Augmented Reality for Mandibular Angle Oblique Split Osteotomy
Musculoskeletal	5	Ai	2011	P	2	Transoral Atlantoaxial Reduction Plate Internal Fixation with Transoral Transpedicular or Articular Mass Screw of C2 for the Treatment of Irreducible Atlantoaxial Dislocation
Musculoskeletal	5	Bellanova	2013	S	4	Surgical Guides (Patient-Specific Instruments) for Pediatric Tibial Bone Sarcoma Resection and Allograft Reconstruction
Musculoskeletal	5	Brown	2002	S	8	Application of Computer-Generated Stereolithography and Interpositioning Template in Acetabular Fractures: A Report of Eight Cases
Musculoskeletal	5	Chen	2016b	T	1	Image-guided installation of 3D-printed patient-specific implant and its application in pelvic tumor resection and reconstruction surgery.
Musculoskeletal	5	D'Urso	1999d	P	5	Spinal biomodeling
Musculoskeletal	5	D'Urso	2005	P	20	Biomodeling as an Aid to Spinal Instrumentation
Musculoskeletal	5	Fan	2015	P	3	Implantation of customized 3-D printed titanium prosthesis in limb salvage surgery: a case series and review of the literature.
Musculoskeletal	5	Fang	2012	P	1	Guiding Templates For Pedicle Screws Insertion: A preliminary Study
Musculoskeletal	5	Hsu	2015	P	1	Patient-Specific 3-Dimensional Printed Titanium Truss Cage With Tibiotalocalcaneal Arthrodesis for Salvage of Persistent Distal Tibia Nonunion
Musculoskeletal	5	Hung	2008	P	1	Clinical Application of Rapid Prototype Model in Pediatric Proximal Femoral Corrective Osteotomy
Musculoskeletal	5	Li	2014	P	2	Composite artificial semi-knee joint system
Musculoskeletal	5	Lu	2009b	P	9	A novel computer-assisted drill guide template for placement of C2 laminar screws
Musculoskeletal	5	Minns	2003	P	1	The use of a reconstructed three-dimensional solid model from CT to aid the surgical management of a total knee arthroplasty: a case study
Musculoskeletal	5	Otsuki	2016	P	3	Utility of a custom screw insertion guide and a full-scale, color-coded 3D plaster model for guiding safe surgical exposure and screw insertion during spine revision surgery.
Musculoskeletal	5	Paiva	2007	P	1	Application of the stereolithography technique in complex spine surgery
Musculoskeletal	5	Papandrea	2014	P	1	New perspectives on treatment of both bone forearm malunions: three-dimensional (3D) modeling and printing
Musculoskeletal	5	Rahmati	2012	P	1	An improved methodology for design of custom-made hip prostheses to be fabricated using additive manufacturing technologies
Musculoskeletal	5	Singhal	2016	P	2	Improved Surgery Planning Using 3-D Printing: a Case Study.
Musculoskeletal	5	Tan	2015	P	4	A Novel Preoperative Planning Technique Using a Combination of CT Angiography and Three-Dimensional Printing for Complex Toe-to-Hand Reconstruction.
Musculoskeletal	5	Toyoda	2013	P	1	Novel Approach for the Effi cient Use of a Full-Scale, 3-Dimensional Model for Cervical Posterior Fixation
Musculoskeletal	5	Tricot	2012	P	3	3D-corrective osteotomy using surgical guides for posttraumatic distal humeral deformity
Musculoskeletal	5	Truscott	2007	P	1	Using RP to promote collaborative design of customised medical implants
Musculoskeletal	5	Yamazaki	2006	P	1	Surgical Simulation of Instrumented Posterior Occipitocervical Fusion in a Child With Congenital Skeletal Anomaly
External Devices	5	Buehler	2016	T	1	Investigating the Implications of 3D Printing in Special Education
External Devices	5	Eggbeer	2011	T	1	Computer-aided methods in bespoke breast prosthesis design and fabrication
External Devices	5	Faustini	2005	T	1	Design and Analysis of Orthogonally Compliant Features for Local Contact Pressure Relief in Transtibial Prostheses
External Devices	5	Hsu	2010	T	1	The development of a rapid prototyping prosthetic socket coated with a resin layer for transtibial amputees
External Devices	5	Mavroidis	2011	T	1	Patient specific ankle-foot orthoses using rapid prototyping
External Devices	5	Qiao	2016	T	3	A novel combination of computer-assisted reduction technique and three dimensional printed patient-specific external fixator for treatment of tibial fractures
External Devices	5	Rogers	2008	T	1	Case Report: Variably Compliant Transtibial Prosthetic Socket Fabricated Using Solid Freeform Fabrication
External Devices	5	Sengeh	2013	T	1	A Variable-Impedance Prosthetic Socket for a Transtibial Amputee Designed from Magnetic Resonance Imaging Data
External Devices	5	South	2010	T	1	Manufacture of Energy Storage and Return Prosthetic Feet Using Selective Laser Sintering
External Devices	5	Tay	2002	T	1	A CASD-CASM method for prosthetic socket fabrication using the FDM technology
External Devices	5	Yoshikawa	2015	T	1	Rehand: Realistic Electric Prosthetic Hand Created with a 3D Printer

PRISMA 2009 Checklist

Section/topic	#	Checklist item	Reported on page #
**TITLE**			
Title	1	Identify the report as a systematic review, meta-analysis, or both.	Title page
**ABSTRACT**			
Structured summary	2	Provide a structured summary including, as applicable: background; objectives; data sources; study eligibility criteria, participants, and interventions; study appraisal and synthesis methods; results; limitations; conclusions and implications of key findings; systematic review registration number.	1
**INTRODUCTION**			
Rationale	3	Describe the rationale for the review in the context of what is already known.	2
Objectives	4	Provide an explicit statement of questions being addressed with reference to participants, interventions, comparisons, outcomes, and study design (PICOS).	2
**METHODS**			
Protocol and registration	5	Indicate if a review protocol exists, if and where it can be accessed (e.g., Web address), and, if available, provide registration information including registration number.	2
Eligibility criteria	6	Specify study characteristics (e.g., PICOS, length of follow-up) and report characteristics (e.g., years considered, language, publication status) used as criteria for eligibility, giving rationale.	2, 3
Information sources	7	Describe all information sources (e.g., databases with dates of coverage, contact with study authors to identify additional studies) in the search and date last searched.	2
Search	8	Present full electronic search strategy for at least one database, including any limits used, such that it could be repeated.	Supplementary material
Study selection	9	State the process for selecting studies (i.e., screening, eligibility, included in systematic review, and, if applicable, included in the meta-analysis).	3
Data collection process	10	Describe method of data extraction from reports (e.g., piloted forms, independently, in duplicate) and any processes for obtaining and confirming data from investigators.	3, 4
Data items	11	List and define all variables for which data were sought (e.g., PICOS, funding sources) and any assumptions and simplifications made.	3, 4, Supplementary material
Risk of bias in individual studies	12	Describe methods used for assessing risk of bias of individual studies (including specification of whether this was done at the study or outcome level), and how this information is to be used in any data synthesis.	4
Summary measures	13	State the principal summary measures (e.g., risk ratio, difference in means).	4
Synthesis of results	14	Describe the methods of handling data and combining results of studies, if done, including measures of consistency (e.g., I^2) for each meta-analysis.	4

For peer review only

PRISMA 2009 Checklist

Section/topic	#	Checklist item	Reported on page #
Risk of bias across studies	15	Specify any assessment of risk of bias that may affect the cumulative evidence (e.g., publication bias, selective reporting within studies).	4, Table 3
Additional analyses	16	Describe methods of additional analyses (e.g., sensitivity or subgroup analyses, meta-regression), if done, indicating which were pre-specified.	N/A
**RESULTS**			
Study selection	17	Give numbers of studies screened, assessed for eligibility, and included in the review, with reasons for exclusions at each stage, ideally with a flow diagram.	4, Figure 1
Study characteristics	18	For each study, present characteristics for which data were extracted (e.g., study size, PICOS, follow-up period) and provide the citations.	6, 7, Table 2, Supplementary material
Risk of bias within studies	19	Present data on risk of bias of each study and, if available, any outcome level assessment (see item 12).	8, Table 3, Supplementary material
Results of individual studies	20	For all outcomes considered (benefits or harms), present, for each study: (a) simple summary data for each intervention group (b) effect estimates and confidence intervals, ideally with a forest plot.	Table 2, Supplementary material
Synthesis of results	21	Present results of each meta-analysis done, including confidence intervals and measures of consistency.	N/A
Risk of bias across studies	22	Present results of any assessment of risk of bias across studies (see Item 15).	N/A
Additional analysis	23	Give results of additional analyses, if done (e.g., sensitivity or subgroup analyses, meta-regression [see Item 16]).	N/A
**DISCUSSION**			
Summary of evidence	24	Summarize the main findings including the strength of evidence for each main outcome; consider their relevance to key groups (e.g., healthcare providers, users, and policy makers).	8, 9
Limitations	25	Discuss limitations at study and outcome level (e.g., risk of bias), and at review-level (e.g., incomplete retrieval of identified research, reporting bias).	10
Conclusions	26	Provide a general interpretation of the results in the context of other evidence, and implications for future research.	9, 10
**FUNDING**			
Funding	27	Describe sources of funding for the systematic review and other support (e.g., supply of data); role of funders for the systematic review.	10

From: Moher D, Liberati A, Tetzlaff J, Altman DG, The PRISMA Group (2009). Preferred Reporting Items for Systematic Reviews and Meta-Analyses: The PRISMA Statement. PLoS Med 6(6): e1000097. doi:10.1371/journal.pmed1000097

For more information, visit: www.prisma-statement.org

For peer review only

PRISMA 2009 Checklist

For peer review only
